# Total synthesis of landomycins Q and R and related core structures for exploration of the cytotoxicity and antibacterial properties†

**DOI:** 10.1039/d1ra01088c

**Published:** 2021-03-02

**Authors:** Yao-Hsuan Lai, Soumik Mondal, Hsin-Tzu Su, Sheng-Cih Huang, Mine-Hsine Wu, I.-Wen Huang, Tsai-Ling Yang Lauderdale, Jen-Shin Song, Kak-Shan Shia, Kwok-Kong Tony Mong

**Affiliations:** National Chiao Tung University Hsinchu 30010 Taiwan Republic of China; National Yang Ming Chiao Tung University Hsinchu 30010 Taiwan Republic of China tmong@nycu.edu.tw; National Health Research Institutes Miaoli County 35053 Taiwan Republic of China

## Abstract

Herein, we report the total synthesis of landomycins Q and R as well as the aglycone core, namely anhydrolandomycinone and a related core analogue. The synthesis features an acetate-assisted arylation method for construction of the hindered B-ring in the core component and a one-pot aromatization–deiodination–denbenzylation procedure to streamline the global functional and protecting group manuipulation. Subsequent cytotoxicity and antibacterial studies revealed that the landomycin R is a potential antibacterial agent against methicillin-resistant *Staphylococcus aureus*.

## Abstract 2

Herein, we report the total synthesis of landomycins Q and R as well as the aglycone core, namely anhydrolandomycinone and a related core analogue.

## Graphical abstract

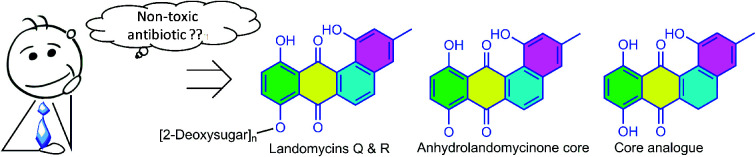

## Introduction

Over the past few decades, the emergence of multidrug-resistant nosocomial pathogens has become a worldwide issue that is of concern for scientists and clinical practitioners.^1,2^ Consequently, new antibiotics and antibacterial strategies are urgently needed.^3,4^ However, the development of a new antibiotic is a slow process that is associated with a high risk of failure. Compounds that exhibit antimicrobial capacity *in vitro* may be unable to combat the infection *in vivo* or may be highly toxic to the host cells.^5^

*Staphylococcus aureus* (*S. aureus*) is a class of Gram-positive bacteria that is present on the skin and mucous membranes as a part of normal flora.^6^ If by chance *S. aureus* bacteria enter the bloodstream, they may cause severe infections including meningitides, endocarditis, and urinary tract infections.^7^ Staphylococcal infections are common in community and hospital-acquired settings and treatment of these infections has been complicated because of the rising incidence of methicillin resistant *S. aureus* (MRSA) infections.^8^ Although vancomycin is the antibiotic of choice, it may cause nephrotoxicity at high doses.^9,10^ Therefore, a less or non-toxic antibiotic is desirable.^11–13^

**Fig. 1:**
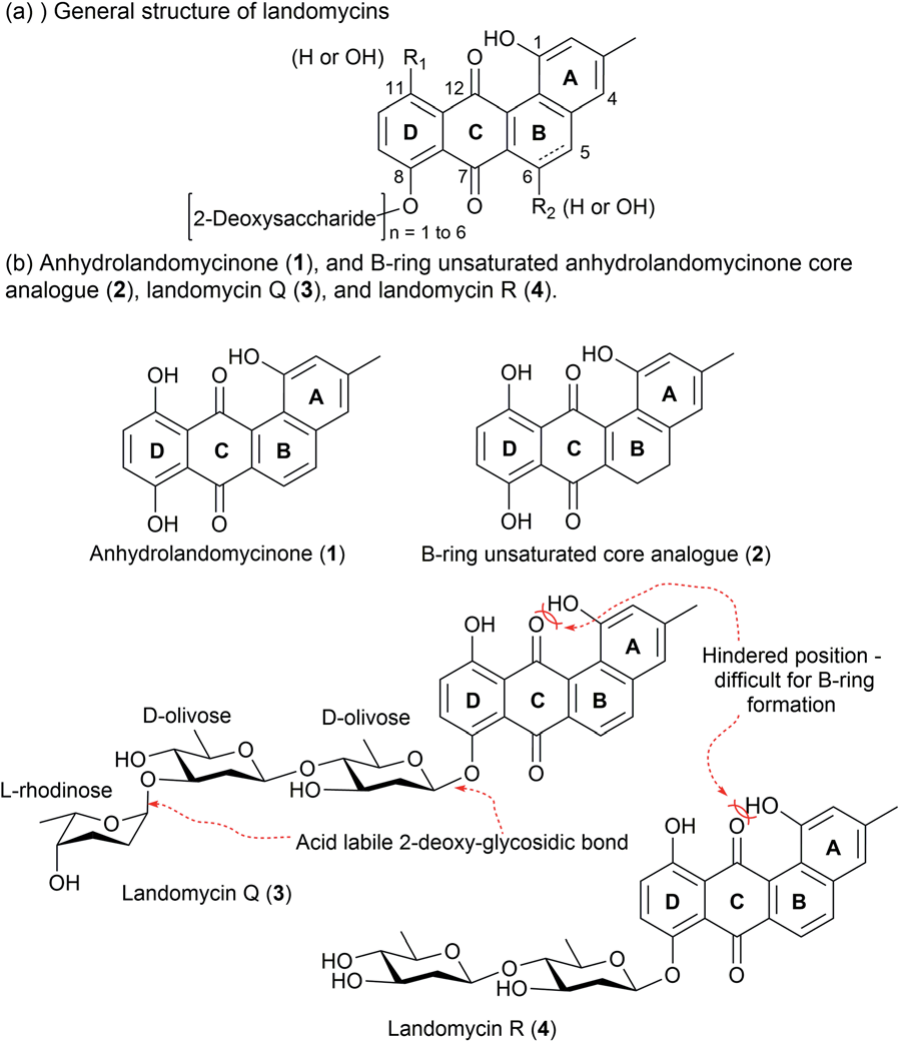
(a) General Structure of LAs. (b) Structures of anhydrolandomycinone aglycone core (1), and B-ring unsaturated core analogue (2), LA R (3) and LA Q (4).

The landomycins (LAs) are a class of angucyclines produced from *Streptomyces* species.^14^ The first report on LA described the isolation of LA A to D from *S. cyanogenus* S136.^15^ Through the use of the modern molecular biology techniques, the LA family has now grown to include more than 100 members.^16,17^ The general structure of LA compounds comprises a tetracyclic core, which is substituted with some hydroxyl groups and a 2-deoxysaccharide chain at the C8 position (Fig. 1a).^14–17^ Based on the B-ring structure and hydroxyl substitution pattern, four different core structures have been identified: tetrangulol, 5,6-anhydrolandomycinone, landomycinone, and 11-deoxy-landomycinone. The combinations of different core structures and 2-deoxysaccharide chains provide a rich pool of natural products for structure and activity relationship studies.

Previous studies of LAs have mainly focused on the anti-cancer activity, and their potential antibacterial property has received far less attention.^18^ Given that the demand for non-toxic antibiotics is on the rise, we sought to establish a versatile synthetic route to procure the anhydrolandomycinone core (1), the B-ring unsaturated core analogue (2), the LA Q (3), and LA R (4) for exploration of the cytotoxic and antibacterial properties (Fig. 1b).

## Results and discussion

### Synthesis of anhydrolandomycinone (1), B-ring unsaturated core analogue (2), LA Q (3), and LA R (4)

The total synthesis of LA A and LA D has been reported by Yu and Yang, but the synthesis of LA Q (3) and LA R (4) with a different core structure has not yet been realized.^19^ From structural perspective, the synthesis of this class of compounds is non-trivial and yet challenging. Currently available protocols toward their aglycone core structure and glycone component alone are inadequate for procurement of a complete LA scaffold.^20,21^ Some modifications are required for selective removal of protecting groups and subsequent coupling reactions; but such modifications may not be tolerable to the original protocols. Thereby, we envisaged some synthetic challenges to realize the structures of our targets. These include: (i) the construction of a highly hindered B-ring; (ii) stereocontrol of the formation of the 2-deoxy-β-glycosidic bonds; and (iii) the global deprotection in conditions tolerated by the rather fragile trideoxy- and dideoxy-glycosidic bonds (Fig. 1b).^22^

To tackle these challenges, new synthetic routes were designed, which was based on the modifications of literature protocols.^19–21^ To access anhydrolandomycinone core (1) and its B-ring unsaturated analogue (2), the D-ring and A-ring building blocks 5 and 6 were prepared from available hydroquinone 7 and dimethylanisole 8, respectively. Based on the building blocks 5 and 6, the C and B-rings of the core scaffold were constructed.

**Scheme 1:**
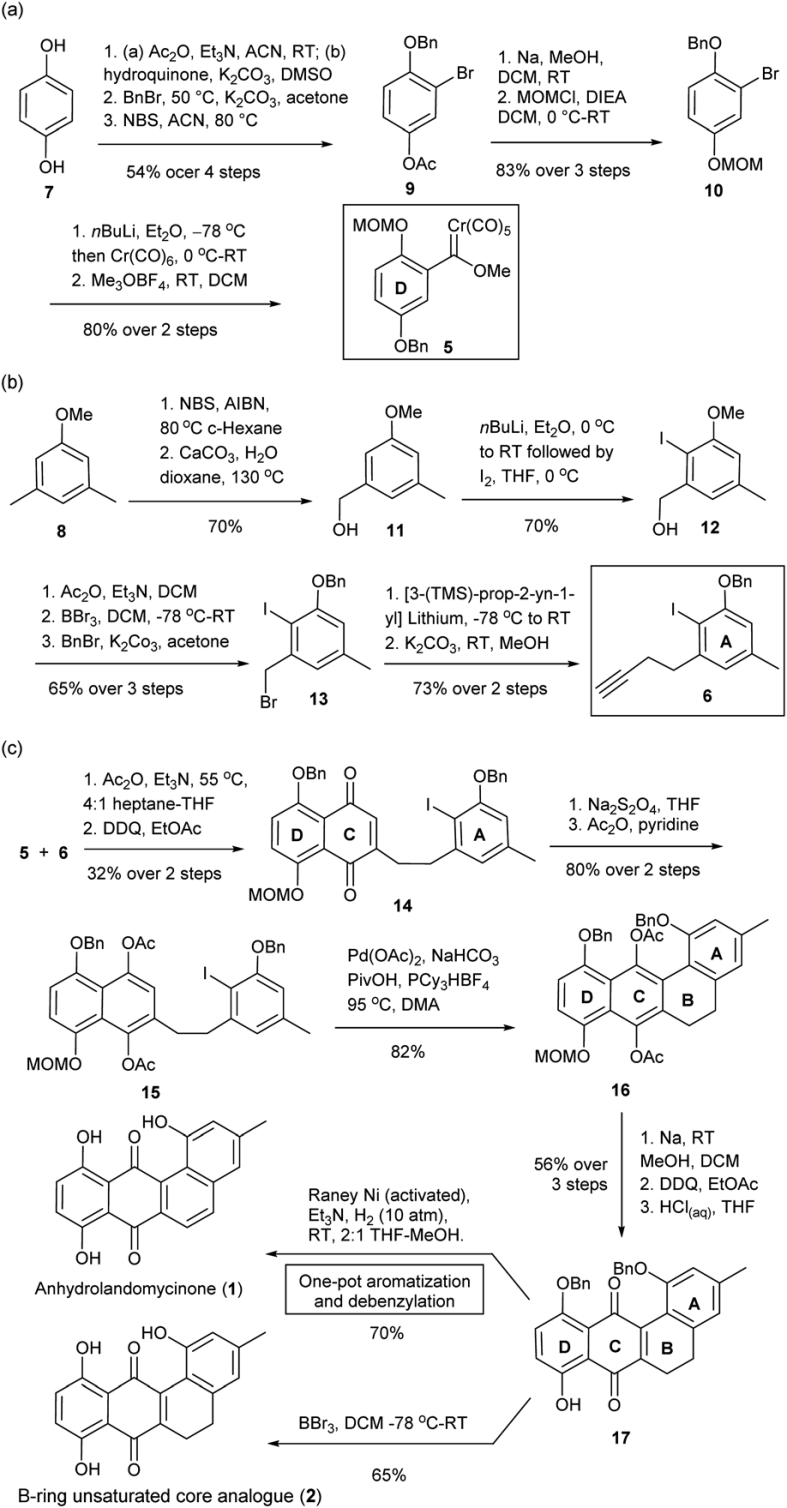
Preparation of (a) D-ring building block 5, (b) A-ring alkyne building block 6, (c) anhydrolandomycinone (1), and corresponding B-ring unsaturated core analogue (2).

In the preparation of the D-ring building block 5, regio-selective acetylation of hydroquinone 7 with Zhang's procedure^23^ followed by sequential benzylation and bromination gave the known 3-bromo-4-hydroxyphenyl acetate 9.^24^ Then, 9 was converted to 1-(benzyloxy)-2-bromo-4-(methoxy-methoxy)-benzene 10 through deacetylation and methoxymethyl ether (MOM) protection (Scheme 1a). Subsequent lithiation of 10 in couple with Meerwein's salt methylation completed the preparation of building block 5 in 37% yield over seven steps.^25^ Because of the poor stability of 5, it was freshly prepared prior to the Dötz benzannulation shown in Scheme 1c.

The preparation of the A-ring building block 6 started with readily available 3,5-dimethylaniosle 8 (ref. 20*b*) instead of the more expensive 3-bromo-5-methylphenol as was the case in our previous synthesis.^20*d*^ Thus, bromination of 3,5-dimethylaniosle 8 with Wohl–Ziegler's procedure^26^ followed by hydroxyl substitution afforded known (3-methoxy-5-methylphenyl)-methanol 11 (Scheme 1b).^20*b*^ Subsequent directed-*ortho* lithiation of 11 and regioselective iodination gave (2-iodo-3-methoxy-5-methylphenyl)methanol 12 as the sole isomer.^20*b*,27^ Acetylation of 12 followed by replacement of the methoxy group with benzyl ether protection provided 1-(benzyloxy)-3-(bromomethyl)-2-iodo-5-methylbenzene 13.^20*d*^ Finally, the substitution of 13 with *in situ* prepared [3-TMS-prop-2-yn-1-yl] lithium and desilylation completed the preparation of building block 6 in 23% yield over eight steps.

With the D- and A-ring building blocks 5 and 6 in hand, we embarked on the C-ring construction *via* Dötz benzannulation (Scheme 1c).^28^ The annulation was followed by 2,3-dichloro-5,6-dicyano-benzoquinone (DDQ) oxidation to give the A-ring tethered naphthalene-1,4-dione 14; which through Na_2_S_2_O_4_ reduction and acetylation was converted to the A-ring tethered naphthalene-1,4-diacetate 15. Subsequent acetate-assisted intramolecular directed arylation enabled construction of the B-ring in a highly congested environment.^29^ The desired 5,6-dihydrotetraphene-7,12-diyl-diacetate 16 was procured in a good 82% yield along with just a trace amount of the deiodination product of 15. Removal of the acetyl groups of 16 followed by DDQ oxidation and acidic cleavage of the MOM protecting group gave 5,6-dihydrotetraphene-7,12-dione 17. In subsequent hydrogenolytic debenzylation, the modified RANEY®Ni catalyzed hydrogenation conditions were applied; whereas, the RANEY®Ni catalyst was freshly activated with NaOH_(aq)_ and triethylamine (Et_3_N) was added as an acid scavenger.^30^ Under the new conditions, no reduction of the carbonyl groups at C-ring occurred, while the B-ring was aromatized to give desired anhydrolandomycinone (1) in a one-pot fashion. In previous hydrogenation conditions, the carbonyl groups at the C-ring were also reduced, which had to be recovered by the DDQ oxidation.^19,20*d*^ In addition to the synthesis of (1), removal of the benzyl protecting groups of 17 with BBr_3_ furnished the B-ring unsaturated core analogue (2).

**Scheme 2:**
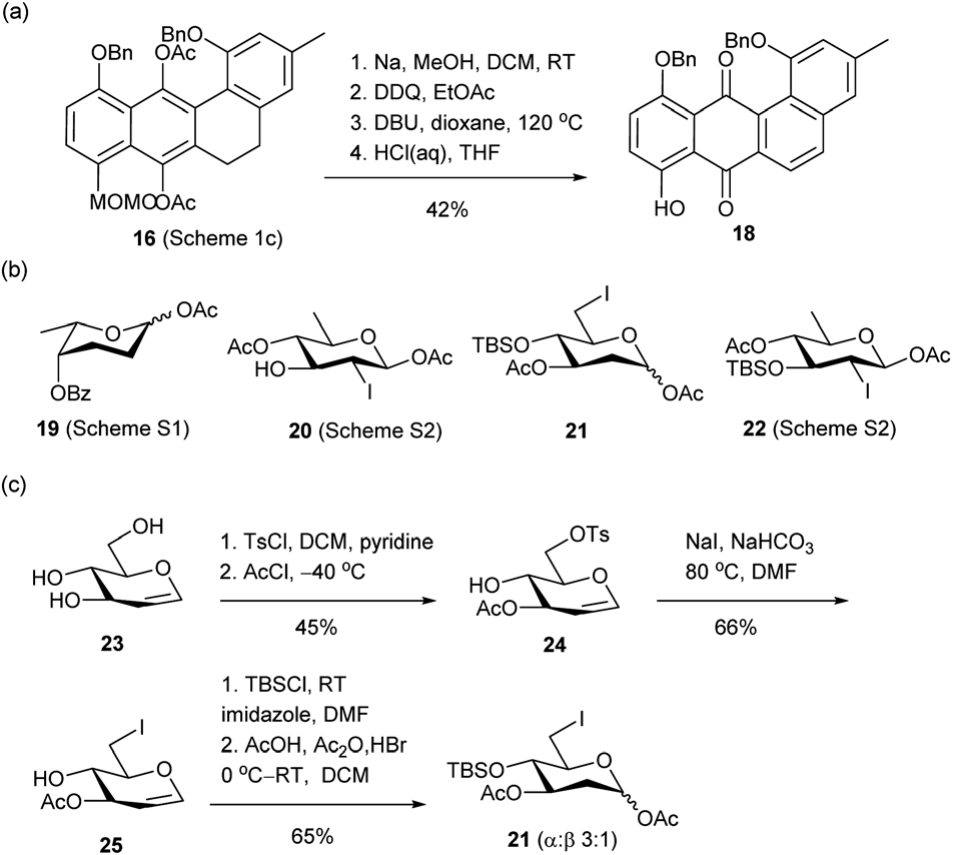
(a) Preparation of anhydrolandomycinone acceptor 18. (b) Structures of l-rhodinosyl acetate 19 and olivosyl acetates 20–22. (c) Preparation of d-olivosyl acetate 21.

For the synthesis of LA Q (3) and R (4), partially protected anhydrolandomycinone 18 was initially employed as the aglycone acceptor, which could be derived from advanced intermediate 16*via* four functional- and protecting-group manipulation steps (Scheme 2a). The 2-deoxytrisaccharide component of LA Q (3) was assembled from l-rhodinosyl acetates 19 (ref. 31) and, d-olivosyl acetates 20,^19*a*^ and 21, while the 2-deoxy-disaccharide component of LA R (4) was built from the d-olivosyl acetates 21 and 22 (ref. 19*a*) (Scheme 2b). l-Rhodinosyl acetate 19 was derived from l-rhamnose (Scheme S1†), and d-olivosyl acetates 20 and 22 were derived from known glucal 23 according to literature procedures (Scheme S2†).^32^ 6-Iodo-olivosyl acetate 21 was also prepared from 23*via* intermediates 24 and 25 in 20% overall yield (Scheme 2c).

**Scheme 3:**
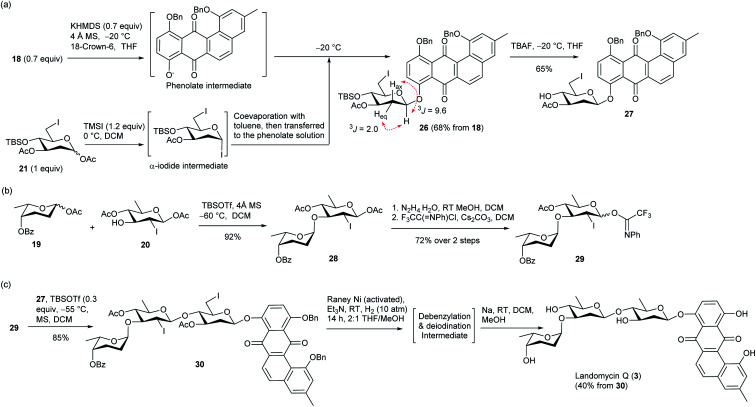
(a) Preparation of anhydrolandomycinonyl β-olivoside acceptor 27. (b) Preparation of disaccharide imidate donor 29. (c) Total synthesis of LA Q (3).

In the construction of LA Q (3), an anhydrolandomycinonyl β-olivoside acceptor was prepared, which was subsequently coupled with a non-reducing-end disaccharide donor. In the preparation of the β-olivoside acceptor, 6-iodo-olivosyl acetate 21 was converted to an α-olivosyl iodide by treatment with trimethylsilyl iodide (TMSI) (Scheme 3a).^33^ After formation of the iodide intermediate, the DCM and residual TMSI were removed and the crude intermediate was reacted with the phenolate nucleophile, which was prepared *in situ* by deprotonation of 18 with potassium hexamethyldisilazane (KHMDS). Under the S_N_2 glycosylation conditions, the desired anhydrolandomycinonyl β-olivoside 26 was obtained in a satisfactory 68% yield with perfect selectivity. Assignment of the configuration of the *O*-aryl 2-deoxy-β-glycosidic bond was supported by the ^3^*J*_H1-Hax_ coupling constant of 9.6 Hz. Notably, the above substitution occurred exclusively at C1 position despite the presence of the iodo substituent at the C6 position. Subsequent removal of the silyl protecting group afforded the desired β-olivoside acceptor 27 in 65% yield.

For the preparation of the disaccharide donor, olivosyl acetate acceptor 20 was coupled with rhodinosyl acetate donor 19 to give α-rhodinosyl-(1,3)-olivosyl acetate 28 (Scheme 3b). In the glycosylation, a low −60 °C temperature was applied to avoid the cleavage of the fragile 2,3,6-trideoxyglyosidic bond. Final replacement of the anomeric acetate group of 28 with *N*-phenyltrifluoroacetimidate completed the preparation of disaccharide imidate donor 29.

The availability of donor 29 set the stage for the assembly of the target (Scheme 3c). Glycosylation of acceptor 27 with donor 29 gave protected LA Q 30 in a high 85% yield with excellent β-selectivity. The selectivity of the glycosylation is attributed to the participatory effect of the 2-iodo substituent group.^34^ Subsequent one-pot deiodination and debenzylation of 30 could be achieved under the same RANEY®Ni hydrogenation conditions as shown in Scheme 1c. Hydrolysis of the acyl protecting groups of the deiodinated and debenzylated intermediate concluded the synthesis of LA Q (3) in 40% yield over two steps from 30. The identity and structure of 3 were confirmed with HRMS and NMR spectroscopy. The proton chemical shifts of (3) were in full agreement with the data given from an authentic sample (Table S1†).^16*c*^

**Scheme 4:**
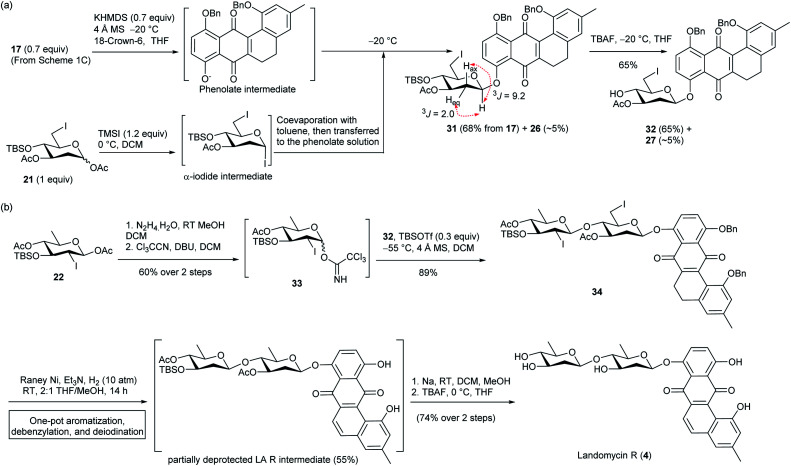
(a) Preparation of B-ring unsaturated anhydrolandomycinonyl core acceptor 32. (b) Total synthesis of LA R (4).

Inspired by the one-pot debenzylation and aromatization in Scheme 1c, and the one-pot debenzylation and deiodination in Scheme 3c, we envisaged a simpler synthetic route for LA R (4), which would invoke a one-pot three-step, *i.e.* aromatization–debenzylation–deiodination, procedure. With this aim, 5,6-dihydrotetraphene-7,12-dione 17 in Scheme 1c was coupled with 6-iodo olivosyl acetate 21 according to the above S_N_2-glycoslation procedure to give 5,6-dihydrotetraphene-7,12-dione β-olivoside 31 in 68% yield along with *ca.* 5% of the inseparable aromatization product 26 (Scheme 4a). The crude β-olivoside 31 was subjected to desilylation to give the 5,6-dihydrotetraphene-7,12-dionyl β-olivoside acceptor 32 along with a small amount of olivoside 27.

On the other hand, the non-reducing end olivosyl acetate 22 was converted to the trichloroacetimidate donor 33 by standard procedures (Scheme 4b). Subsequent glycosylation of β-olivoside acceptor 32 with donor 33 furnished 8-disaccharidyl-5,6-dihydrotetraphene-7,12-dione 34 in 89% yield, though a tiny amount of glycosylation product arising from contaminant olivoside 27 was also produced. Without separation, the crude β-glycoside 34 was subjected to the one-pot aromatization–debenzylation–deiodination procedure to afford a partially deprotected LA R intermediate in a satisfactory 55% yield. Final deprotection of the silyl and acetyl protecting groups concluded the total synthesis of LA R (4) in 74% yield over two steps.

### Exploration of the cytotoxic and antibacterial properties

Having acquired anhydrolandomycinone (1), the B-ring unsaturated core analogue (2), LA Q (3) and LA R (4), we evaluated their cytotoxicity with the [3-(dimethylthiazol-2-yl)-5-(3-carboxymethoxyphenyl)-2-(4-sulfo-phenyl)-2*H*-tetrazolium] (MTS) assay (Table 1).^35,36^ Anhydrolandomycinone (1) was found to be cytotoxic against the cancer cell lines NCI-H460 and SF-268 with IC_50_ values at 7.00 ± 0.70 and 8.57 ± 0.34 μM, respectively; but no appreciable cytoxicity was noted for normal cell line Detroit 551 at 10 μM (Entry 1). Intriguingly, such a toxicity pattern was reversed for the B-ring unsaturated core analogue (2) (Entry 2). At 10 μM concentration of glycosylated LA Q (3) and R (4), no appreciable cytotoxic effect was detected for the normal cell line (Entries 3 and 4). Taken together, the unsaturated B ring and/or the 2-deoxysaccharide chain appear to play a role in the cytotoxicity of the LA compounds.

**Table 1:** Inhibitory effects on normal/cancer cell proliferation by anhydrolandomycinone (1), B-ring unsaturated core analogue (2), LA Q (3), and LA R (4)^a^

Entry	Compound	IC_50_ (μM)		
		NCI-H460	SF-268	Detroit 551
1	Core (1)	7.00 ± 0.70	8.57 ± 0.34	>10.0
2	B-ring unsaturated core (2)	>10.0	>10.0	9.58 ± 0.66
3	LA Q (3)	>10.0	>10.0	>10.0
4	LA R (4)	>10.0	9.82 ± 0.08	>10.0

a Values represent the mean ± SD of three independent experiments.

To elucidate the extent of the cytotoxicity, we evaluated the survival rates of Detroit 551 cell line at 10 μM of the compounds, which were found to be 59.00% ± 3.26% for the core structure (1), 104.2% ± 20.67% for LA Q (3), and 93.56% ± 10.02% for LA R (4), implicating that the anhydrolandomycinone alone is more toxic than its glycosylated products.

After clarifying the cytotoxicity profiles of 1–4, we examined the antibacterial activity with MRSA 4N 216 and 7Y001 (Table 2).^37,38^ Among the compounds examined, only the disaccharide substituted LA R (4) exhibited inhibitory activity against MRSA 4N 216 and 7Y001 with the MIC values of 8 and 4 μg mL^−1^, respectively. Although, such a potency is inferior to that offered by vancomycin (VA), the non-toxic nature of LA R (4) renders it a potential candidate for further optimization.

**Table 2:** *In vitro* inhibitory assay with anhydrolandomycinone (1), B-ring unsaturated core analogue (2), LA Q (3), and LA R (4)^a^

MRSA strain	MIC (μg mL^−1^) of compound				
	Core (1)	Analogue (2)	LA Q (3)	LA R (4)	VA
4N216	>8	>8	>8	8	1
7Y001	>8	>8	>8	4	1

a Values are derived from two independent MIC experiments.

## Conclusion

In summary, a versatile strategy was established for the total synthesis of the anhydrolandomycinone core (1), the B-ring unsaturated anhydrolandomycinone analogue (2), landomycin Q (3), and landomycin R (4). The strategy employed the acetate-assisted intramolecular arylation for construction of the hindered B-ring of the anhydrolandomycin core and a one-pot aromatization, deiodination, and debenzylation procedure was established to simplify the end-stage functional group manipulations. In subsequent MTS study, the structure of the B-ring and the degree of glycosylation of the landomycins appear to play a role in the cytotoxicity. Further exploration of the antibacterial properties revealed the potential of landomycin R (4) for inhibition of MRSA 4N 216 and 7Y001.

## Conflicts of interest

There are no conflicts to declare.

## Supplementary Material

RA-011-D1RA01088C-s001
