# Supplementary material for: Total synthesis of landomycins Q and R and related core structures for exploration of the cytotoxicity and antibacterial properties
Source: RSC Adv. 2021 Mar 2;11(16):9426–32. doi: 10.1039/d1ra01088c (PMC8695357; doi:10.1039/d1ra01088c)

Electronic Supplementary Material (ESI) for RSC Advances.
This journal is © The Royal Society of Chemistry 2021

Supporting Information

Total Synthesis of Landomycins Q and R and Related Core Structures for Exploration of Cytotoxicity and Antibacterial Properties

Yao-Hsuan Lai,[‡,a] Soumik Mondal,[‡,a,b] Hsin-Tzu Su,[a,b] Sheng-Cih Huang,[a,b] Mine-Hsine Wu,[c] I-Wen Huang,[c] Tsai-Ling, Yang Lauderdale,[c] Jen-Shin Song,[c] Kak-Shan Shia,[c] Kwok-Kong Tony Mong*,[a,b]

[a] National Chiao Tung University, Hsinchu 30010, Taiwan, R.O.C. [b] National Yang Ming Chiao Tung University, Hsinchu 30010, Taiwan, R.O.C. [c] National Health Research Institutes, Maoli County 35053, Taiwan, R.O.C.

Contents

1. General experimental 6

Table S1: Comparison of ^{1}H Chemical Shifts of LA Q (3) with the Literature Values. 7

2. Experimental procedures 8

2.1 Synthesis of 4-(Benzyloxy)-3-bromophenyl Acetate 9 8

2.2 Synthesis of 1-(Benzyloxy)-2-bromo-4-(methoxymethoxy)benzene 10 9

2.3 Synthesis of [(2-Benzyloxy-5-methoxymethoxyphenyl)methoxymethylene]-pentacarbonyl-chromium 5 10

2.4 Synthesis of (3-Methoxy-5-methylphenyl)methanol 11 11

2.5 Synthesis of (2-Iodo-3-methoxy-5-methylphenyl)methanol 12 11

2.6 Synthesis of 2-Iodo-3-methoxy-5-methylbenzyl acetate 12A 12

2.7 Synthesis of 3-Bromo-2-iodo-5-methylphenol 12B 13

2.8 Synthesis of 1-(Benzyloxy)-3-(bromomethyl)-2-iodo-5-methylbenzene 13 13

2.9 Synthesis of 1-(Benzyloxy)-3-(but-3-yn-1-yl)-2-iodo-5-methylbenzene A ring alkyne 6 14

2.10 Synthesis of 5-Benzyloxy-2-(3-benzyloxy-2-iodo-5-methyl-phenethyl)-8-(methoxymethoxy)-naphthalene 14A and naphthalene-1,4-dione 14 15

2.11 Synthesis of 5-Benzyloxy-2-(3-benzyloxy-2-iodo-5-methyl-phenethyl)-8-(methoxymethoxy)naphthalene-1,4-diyl Diacetate 15 16

2.12 Synthesis of 1,11-Bis(benzyloxy)-8-(methoxymethoxy)-3-methyl-5,6-dihydrotetraphene-7,12-diyl Diacetate 16 17

2.13 Synthesis of 1,11-Bis(benzyloxy)-8-(methoxymethoxy)-3-methyl-5,6-dihydrotetraphene-7,12-dione 16A 18

2.14 Synthesis of 1,11-Bis(benzyloxy)-8-hydroxy-3-methyl-5,6-dihydrotetraphene-7,12-dione 17 .. 19

2.15 Synthesis of anhydrolandomycinone (1) 20

2.16 Synthesis of B-ring unsaturated core analogue (2) 20

2.17 Synthesis of 1,11-Bis(benzyloxy)-8-hydroxy-3-methyltetraphene-7,12-dione 18 21

Scheme S1. Preparation of 4-*O*-Benzoyl-2,3,6-trideoxy-L-rhodinose Acetate 19 22

2.18 Synthesis of 3,4-Di-O-acetyl-1,5-anhydro-2,6-dideoxy-L-arabino-hex-1-enol 19A 23

2.19 Synthesis of Methyl-4-*O*-acetyl-2,3,6-trideoxy-L-erythro-hex-2-enopyranoside 19B 24

2.20 Synthesis of Methyl-4-*O*-benzoyl-2,3,6-trideoxy-L-erythro-hex-2-enopyranoside 19C 24

2.21 Synthesis of Methyl-4-*O*-benzoyl-2,3,6-trideoxy-L-threo-hexopyranoside 36D 25

2.22 Synthesis of 1-*O*-Acetyl-4-*O*-benzoyl-2,3,6-trideoxy-L-rhodinose 19 26

Scheme S2. Synthesis of 1,4-Di-*O*-acetyl-3-*O*-*tert*-butyldimethylsilyl-2,6-dideoxy-2-iodo-*β*-D-glucopyranoside 22 and 1,4-Di-O-acetyl-2,6-dideoxy-2-iodo-*β*-D-glucopyranoside 20 27

2.23 Synthesis of 3-*O*-*tert*-Butyldimethylsilyl-6-iodo-D-glucal 23B 27

2.24 Synthesis of 4-*O*-Acetyl-3-*O*-*tert*-butyldimethylsilyl-6-deoxy-D-glucal 23C 28

2.25 Synthesis of 1,4-Di-*O*-acetyl--3-*O*-*tert*-butyldimethylsilyl-2,6-dideoxy-2-iodo-*β*-D-glucopyranoside 22 29

2.26 Synthesis of 1,4-Di-*O*-acetyl-2,6-dideoxy-2-iodo-*β*-D-glucopyranoside 20 30

2.27 Synthesis of 3-*O*-Acetyl-6-iodo-D-glucal 25 30

2.28 Synthesis of 3-*O*-Acetyl-6-iodo-4-*O*-*tert*-butyldimethylsilyl-D-glucal 25A 32

2.29 Synthesis of 3-*O*-Acetyl-6-iodo-4-*O*-*tert*-butyldimethylsilyl-2,6-dideoxy-D-glucopyranosyl Acetate 21 32

2.30 Synthesis of Anhydrolandomycinonyl *β*-Olivoside 26 33

2.31 Synthesis of Anhydrolandomycinonyl *β*-Olivoside Acceptor 27 34

2.32 Synthesis of α-Rhodinosyl-(1,3)-olivosyl Acetate 28 35

2.33 Synthesis of α-Rhodinosyl-(1,3)-olivosyl *N*-Phenyltrifluoroacetimidate 29 36

2.34 Assembly of Protected Landomycin Q 30 37

Activation Procedure for Raney Ni 38

2.35 Synthesis of Landomycin Q (3) 38

2.36 Synthesis of 5,6-Dihydrotetraphene-7,12-dione *β*-Olivoside 31 40

2.37 Synthesis of 5,6-Dihydrotetraphene-7,12-dione *β*-Olivoside Acceptor 32 41

2.38 Synthesis of Olivosyl Trichloroacetimidate Donor 33 41

2.39 Synthesis of 5,6-Dihydrotetraphene-7,12-dionyl Disaccharide 34 42

2.40 Synthesis of Landomycin R (4) 43

MTS Assay for Cell Viability 46

Table S2. Raw Data of Inhibitory Effects on Cell Proliferation by Anhydrolandomycinone (1), B-ring unsaturated core analogue (2), LA Q (3), and LA R (4). 47

Table S3. Inhibitory Effects on Normal or Cancer Cell Proliferation. 48

Minimum Inhibitory Concetnration (MIC) Determination 49

Table S4: Anhydrolandomycinone core (1), Core analogue (2), LA Q (3), LA R (4) and Vancomycin (VA) After Incubation with MRSA 4N216 for 20 and 24 h. 50

Table S5: Anhydrolandomycinone core (1), Core analogue (2), LA Q (3), LA R (4) and Vancomycin (VA) After Incubation with MRSA 7Y001 for 20 and 24 h 51

4. References 52

^{1}H NMR spectrum of 4-(Benzyloxy)-3-bromophenyl Acetate 9 54

^{1}H NMR spectrum of 1-(Benzyloxy)-2-bromo-4-(methoxymethoxy)benzene 10 56

^{1}H NMR spectrum of (2-Iodo-3-methoxy-5-methylphenyl)methanol 12 58

^{1}H NMR spectrum of 2-Iodo-3-methoxy-5-methylbenzyl Acetate 12A 60

^{1}H NMR spectrum of 3-Bromo-2-iodo-5-methylphenol 12B 62

^{1}H NMR spectrum of 1-(Benzyloxy)-3-(bromomethyl)-2-iodo-5-methylbenzene 13 64

^{1}H NMR spectrum of 1-(Benzyloxy)-3-(but-3-yn-1-yl)-2-iodo-5-methylbenzene 6 66

^{1}H NMR spectrum of 5-Benzyloxy-2-(3-benzyloxy-2-iodo-5-methylphenethyl)-8-(methoxymethoxy)naphthalene-1,4-dione 14A 68

^{1}H NMR spectrum of 5-Benzyloxy-2-(3-benzyloxy-2-iodo-5-methylphenethyl)-8-(methoxymethoxy)naphthalene-1,4-diyl diacetate 15 71

^{1}H NMR spectrum of 1,11-Bis(benzyloxy)-8-(methoxymethoxy)-3-methyl-5,6-dihydrotetraphene-7,12-diyl Diacetate 16 74

^{1}H NMR spectrum of 1,11-Bis(benzyloxy)-8-(methoxymethoxy)-3-methyl-5,6-dihydrotetraphene-7,12-dione 16A 77

^{1}H NMR spectrum of 1,11-Bis(benzyloxy)-8-hydroxy-3-methyl-5,6-dihydrotetraphene-7,12-dione 17 80

^{1}H NMR spectrum of anhydrolandomycinone (1) 83

^{1}H NMR spectrum of B-ring unsaturated core analogue (2) 85

^{1}H NMR spectrum of 1,11-Bis(benzyloxy)-8-hydroxy-3-methyltetraphene-7,12-dione 18 87

^{1}H NMR spectrum of 3,4-Di-*O*-acetyl-1,5-anhydro-2,6-dideoxy-L-*arabino*-hex-1-enol 36A........ 89

^{1}H NMR spectrum of Methyl 4-*O*-Acetyl-2,3,6-trideoxy-L-*erythro*-hex-2-enopyranoside 36B 90

^{1}H NMR spectrum of Methyl 4-*O*-Benzoyl-2,3,6-trideoxy-L-*erythro*-hex-2-enopyranoside 36C 91

^{1}H NMR spectrum of Methyl 4-*O*-Benzoyl-2,3,6-trideoxy-α-L-*threo*-hexopyranoside 36D........ 92

^{1}H NMR spectrum of 1-*O*-acetyl-4-*O*-benzoyl-2,3,6-trideoxy-α-L-rhodinose 19........ 93

^{1}H NMR spectrum of 3-*O*-*tert*-Butyldimethylsilyl-6-iodo-D-glucal 23B 94

^{1}H NMR spectrum of 4-*O*-Acetyl-3-*O*-*tert*-butyldimethylsilyl-6-deoxy-D-glucal 23C 96

^{1}H NMR spectrum of 1,4-Di-*O*-acetyl--3-*O*-*tert*-butyldimethylsilyl-2,6-dideoxy-2-iodo-*β*-D-glucopyranoside 22 100

^{1}H NMR spectrum of 1,4-Di-*O*-acetyl--3-*O*-*tert*-butyldimethylsilyl-2,6-dideoxy-2-iodo-*α*-D-mannopyranoside 23D........ 103

^{1}H NMR spectrum of 1,4-Di-*O*-acetyl-2,6-dideoxy-2-iodo-β-D-glucopyranoside 20 104

^{1}H NMR spectrum of 3-*O*-Acetyl-6-iodo-D-glucal 25........ 108

^{1}H NMR spectrum of 3-*O*-Acetyl-6-iodo-4-*O*-*tert*-butyldimethylsilyl-D-glucal 25A 111

^{1}H NMR spectrum of 3-*O*-Acetyl-6-iodo-4-*O*-*tert*-butyldimethylsilyl-2,6-dideoxy-D-glucopyranosyl acetate 21 113

^{1}H NMR spectrum of Anhydrolandomycinonyl *β*-Olivoside 26 117

^{1}H NMR spectrum of Anhydrolandomycinonyl *β*-Olivoside Acceptor 27 121

^{1}H NMR spectrum of *α*-Rhodinosyl-(1,3)-Olivosyl Acetate 28 125

^{1}H NMR spectrum of protected LA Q 30 129

^{1}H NMR spectrum of landomycin Q (3) 133

^{1}H NMR spectrum of 5,6-Dihydrotetraphene-7,12-dione *β*-olivoside 31 137

^{1}H NMR spectrum of 5,6-Dihydrotetraphene-7,12-dione *β*-Olivoside Acceptor 32 141

^{1}H NMR spectrum of Protected 8-Disaccharidyl-5,6-dihydrotetraphene-7,12-dione 34 145

^{1}H NMR spectrum of Partially Deprotected LA R intermediate 34A 149

^{1}H NMR spectrum of Landomycin R (4) 150

1. General experimental

All the moisture sensitive reactions were performed in oven- or flame-dried flasks under the nitrogen or argon atmosphere. Reagents were purchased from Acros®, Alfa Aeser®, Sigma Aldrich® and some local chemical companies and used directly without purification. All solvents were dried under conventional way before using. Aluminum plates (60 F254) coated with silica-gel obtained from Merck were used for thin layer chromatography (TLC). Compounds were visualized under ultraviolet (UV) light and also by staining with phosphomolybdic acid (PMA) solution followed by heating on a hot plate. Silica-gel (100-200 mesh) was used for flash column chromatography. NMR spectra were recorded on Varian UI (400 MHz) or Varian UI (600 MHz) spectrometers in CDCl3 or Acetone-d_6 or DMSO-d_6 or Benzene-d_6 at ambient temperature. High resolution mass spectra were recorded on Joel Accurate Mass Q-Tof GCX instrument for electrospray ionization (ESI) and are reported as a ratio of mass to charge (m/z) in Daltons. Optical rotations were measured at 25 ℃ with a AA-65 automatic polarimeter using a quartz cell with 2 mL capacity and a 0.5 cm path length. Concentrations (c) are given in g/100 mL.

Table S1: Comparison of ^{1}H Chemical Shifts of LA Q (3) with the Literature Values.

^{1}H	δ_H (ppm) of LA Q (3) synthesized in present study (400 MHz, $CDCl_3$)	δ_H (ppm) of authentic LA Q isolated from *Streptomyces* (500 MHz, $CDCl_3$)[1]
**1-OH**	10.64, s	10.62, s
**2**	7.17, s	7.16, d (*J* = 1.8 Hz)
**3-CH_3**	2.50, s	2.48, s
**4**	7.27, s	7.27, d (*J* = 1.8 Hz)
**5**	8.14, d (*J* = 8.7 Hz)	8.13, d (*J* = 8.7 Hz)
**6**	8.26, d (*J* = 8.6 Hz)	8.25, d (*J* = 8.6 Hz)
**9**	7.55, d (*J* = 9.3 Hz)	7.53, d (*J* = 9.3 Hz)
**10**	7.28, d (*J* = 9.2 Hz)	7.26, d (*J* = 9.3 Hz)
**11-OH**	12.26, s	12.23, s
**H1′**	5.10, dd (*J* = 9.6, 2.0 Hz)	5.08, dd (*J* = 9.5, 2.0 Hz)
**H2′**	1.97, m; 2.82, ddd (*J* = 12.1, 4.4, 1.6 Hz)	2.00, ddd (*J* = 12.7, 12.1, 8.4 Hz); 2.80, ddd (*J* = 12.7, 5.1, 2.0 Hz)
**H3′**	3.76, ddd (*J* = 12.4, 8.4, 5.2 Hz)	3.75,ddd (*J* = 12.0, 8.5, 5.0 Hz)
**OH′**	4.72, s	4.68, br
**H4′**	3.13, dd (*J* = 8.8, 8.4 Hz)	3.12, dd (*J* = 8.4, 8.4 Hz)
**H5′**	3.41, m	3.39, m
**H6′**	1.30, d (*J* = 6.1 Hz)	1.28, d (*J* = 6.2 Hz)
**H1″**	4.54, dd (*J* = 9.2, 1.6 Hz)	4.52, dd (*J* = 9.8, 2.0 Hz)
**H2″**	1.68, m; 2.25, ddd (*J* = 12.8, 4.8, 1.2 Hz)	1.66, ddd (*J* = 12.0, 12.0, 8.0 Hz); 2.24, ddd (*J* = 12.0, 5.0, 1.5 Hz)
**H3″**	3.54, ddd (*J* = 12.0, 8.4, 4.8 Hz)	3.52, ddd (*J* = 12.0, 8.5, 4.8 Hz)
**H4″**	3.13, dd (*J* = 8.8, 8.4 Hz)	3.12, dd (*J* = 8.4, 8.4 Hz)
**OH″**	4.39, s	4.32, br
**H5″**	3.41, m	3.39, m
**H6″**	1.41, d (*J* = 6.1 Hz)	1.39, d (*J* = 6.2 Hz)
**H1‴**	4.96, s	4.94, br
**H2‴**	1.56, m；2.00, m	1.56, m；2.00, m
**H3‴**	1.69, m；2.01, m	1.67, m；2.00, m
**H4‴**	3.65, br	3.63, br
**OH‴**	Not observed.	Not observed.
**H5‴**	4.15, q (*J* = 6.2 Hz)	4.13, q (*J* = 6.7 Hz)
**H6‴**	1.23, d (*J* = 6.8 Hz)	1.21, d (*J* = 6.6 Hz)

2. Experimental procedures

2.1 Synthesis of 4-(Benzyloxy)-3-bromophenyl Acetate 9

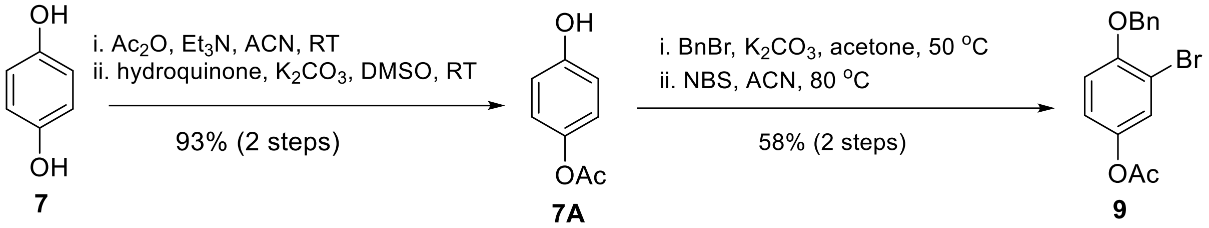

To a solution of hydroquinone **7** (9.9 g, 90.00 mmol) in CH_3CN (90 mL), Ac_2O (21.25 mL, 225.00 mmol) and Et_3N (37.40 mL, 270 mmol) were added at 0 ℃. The reaction mixture was stirred at room temperature for 2 h. After completion of the reaction, the solvent was reduced to ~ 45 mL by a rotary evaporator. Then the concentrated mixture was diluted with EtOAc (150 mL) and washed with 5% aqueous HCl (50 mL × 2), water (50 mL), and brine (50 mL). The organic layer was dried over $MgSO_4$ and filtered. The filtrate was concentrated under *vacuo* to give the crude acetylation product for the next reaction.

To a solution of crude diacetylated product in DMSO (90 mL) above were additional hydroquinone **7** (9.9 g, 90.00 mmol) and K_2CO_3 (12.42 g, 90.00 mmol). The reaction mixture was stirred at RT for 14 h, then diluted with EtOAc (200 mL), washed with water (200 mL × 3), and brine (200 mL). The organic layer was dried over $MgSO_4$ and filtered. The filtrate was concentrated under *vacuo* for flash column chromatography purification (Elution: 17% EtOAc in hexanes) to afford **7A** (25.39 g, 93% over two steps) as a white solid, which is a known compound reported by Zhang *et al.*[2] Analytical data for **7A**: R_f = 0.5 (50% EtOAc in hexanes); ^{1}H NMR (400 MHz, $CDCl_3$): 6.86 (d, J = 8.9 Hz, 2H, H2 and H6), 6.69 (d, J = 9.0 Hz, 2H, H3 and H5), 2.24 (s, 3H, CH_3CO); ^{13}C (100 MHz, $CDCl_3$): 171.1 (C=O), 153.6 (C1), 143.5 (C4), 122.1 (C3 and C5), 115.9 (C2 and C6), 20.9 ($\underline{C}H_3CO$).

To a solution of **7A** (11.82 g, 77.7 mmol) in anhydrous acetone (156 mL) were added benzyl bromide (11.10 mL, 93.32 mmol) and K_2CO_3 (16.10 g, 116.65 mmol). The reaction mixture was stirred at 50 ℃ for 8 h. After completion of the reaction, the reaction mixture was filtered and the filtrate was concentrated under reduced pressure. The residue was dissolved in in DCM (150 mL) and washed with saturated NH_4Cl (50 mL), water (50 mL), and brine (50 mL). The organic

layer was dried over anhydrous $MgSO_4$ and concentrated under reduced pressure. The residue was triturated with hexanes (250 mL) and the product was collected after filtration. This benzyl compound was used in the next reaction without further purification.

To a solution of the benzyl compound (11.19 g, 46.24 mmol) in CH_3CN (93 mL) was added NBS (9.05 g, 50.86 mmol) at RT. The reaction mixture was stirred at 80 ℃ for 16 h. Then the solvent was reduced to 45 mL under reduced pressure. The concentrated mixture was diluted with EtOAc (100 mL) and washed with saturated $NaHCO_3$ (50 mL), water (50 mL), and brine (50 mL). The organic layer was dried over anhydrous $MgSO_4$ and concentrated under reduced pressure for column chromatographic purification (DCM/Hexanes = 1:4) to afford **9** (14.38 g, 58% over two steps) as a white solid.[2] Analytical data for **9**: R_f = 0.25 (50% DCM in hexanes); ^{1}H NMR (400 MHz, $CDCl_3$) δ: 7.47 (d, *J* = 7.6 Hz, 2H), 7.39 (t, *J* = 7.6 Hz, 2H), 7.36-7.29 (m, 2H), 6.98 (dd, *J* = 8.8, 2.4 Hz, 1H, H5), 6.91 (d, *J* = 8.8 Hz, 1H, H6), 5.14 (s, 2H, benzyl CH_2), 2.27 (s, 3H, CH_3CO); ^{13}C NMR (100 MHz, $CDCl_3$) δ: 169.4 (C=O), 153.0 (C4), 144.4 (C1), 136.3, 128.6, 128.0, 127.0, 126.6 (C2), 121.2 (C6), 113.9 (C3), 112.4 (C5), 71.3 (benzyl CH_2), 21.0 ($\mathit{C}H_3CO$); HRMS (ESI): calcd for $C_{15}H_{13}BrNaO_3^+$ $[M + Na]^+$ 342.9940, found *m/z* 342.9942.

2.2 Synthesis of 1-(Benzyloxy)-2-bromo-4-(methoxymethoxy)benzene 10

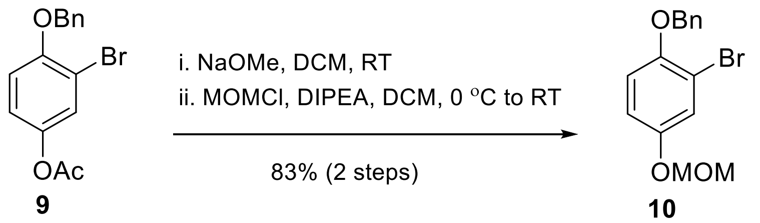

To a solution of bromide **9** (14.38 g, 44.77 mmol) in DCM (90 mL)/MeOH (18.20 mL), sodium metal (929.7 mg, 40.44 mmol) was added at RT and the reaction mixture was stirred for 1 h at the same temperature. After completion of the reaction, 5% aqueous HCl (15 mL) was added to quench the reaction. The reaction mixture was diluted with DCM (45 mL) and washed with satd. NH_4Cl (100 mL), water (100 mL), and brine (100 mL). The organic layer was dried over $MgSO_4$ and concentrated by a rotary evaporator to obtain a crude deacetylation product which was used for the MOM protection.

To a solution of the above crude product in DCM (90 mL), *N*,*N*-diisopropylethylamine (DIEA) (23.50 mL, 134.82 mmol) followed by chloromethyl methyl ether (MOMCl) (6.80 mL, 89.88 mmol) were added slowly at 0 °C. The reaction mixture was stirred at room temperature for 2 h. After completion of the reaction, the solution was quenched by saturated NH_4Cl. Then diluted with DCM (45 mL) and washed with saturated NH_4Cl (50 × 2), water (50 mL), and brine (50 mL). The organic layer was dried over anhydrous $MgSO_4$ and concentrated under reduced pressure for flash column chromatographic purification (EtOAc/Hexanes = 1:7) to afford **10** (11.98 g, 83% over two steps) as a pale-yellow oily liquid. Analytical data for **10**: R_f = 0.35 (17% EtOAc in hexanes); ^{1}H NMR (400 MHz, $CDCl_3$) δ: 7.47 (d, *J* = 7.6 Hz, 2H), 7.41-7.36 (m, 2H), 7.35-7.29 (m, 2H), 6.93 (dd, *J* = 9.2, 2.8 Hz, 1H, H5), 6.86 (d, *J* = 9.2 Hz, 1H, H6), 5.10 (s, 4H), 3.47 (s, 3H); ^{13}C NMR (100 MHz, $CDCl_3$) δ: 151.9 (C4), 150.3 (C1), 136.7, 128.5, 127.9, 127.1, 121.8 (C3), 116.2 (C5), 115.1 (C2), 112.9 (C6), 95.2, 71.7 (benzyl CH_2), 56.0; HRMS (ESI): calcd for $C_{15}H_{15}BrNaO_3^+$ $[M + Na]^+$ 345.0097, found *m/z* 345.0098.

2.3 Synthesis of [(2-Benzyloxy-5-methoxymethoxyphenyl)methoxymethylene]-pentacarbonyl-chromium 5

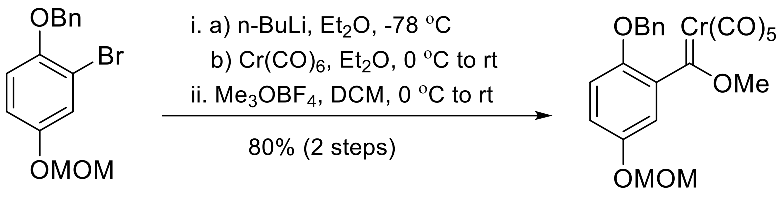

10 **5**

To a solution of bromide **10** (11.57g, 35.79 mmol) in dry Et_2O (90 mL), *n*-BuLi solution (1.6 M in hexanes, 24.70 mL, 39.52 mmol) was added dropwise at –78 °C under N_2. After stirred for 0.5 h, it was cannulated to a solution of $Cr(CO)_6$ (9.49 g, 43.12 mmol) in anhydrous Et_2O (90 mL) at 0 °C. The solution color turned from pale-yellow to brownish-yellow and was stirred at room temoerature for 3 h. Then, the solution was concentrated under reduced pressure and the residue was dissolved in DCM (240 mL) then cooled to 0 °C. Trimethyloxonium tetrafluoroborate (Me_3OBF_4) (7.98 g, 53.90 mmol) was added to the solution and the reaction mixture was stirred at room temperature for 1 h. After completion of the reaction, the solution was concentrated under

reduced pressure and purified by flash column chromatography (EtOAc/Hexanes = 1:7) to afford **5** (13.74 g, 80% over two steps) as a dark red oily liquid. Compound **5** is unstable at room temperature and so used for the next step without characterization.

2.4 Synthesis of (3-Methoxy-5-methylphenyl)methanol 11

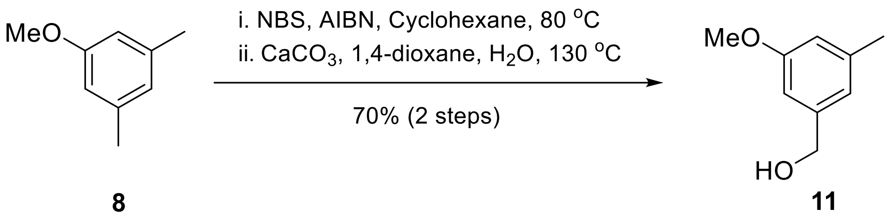

To a solution of **8** (20.82 g, 152.87 mmol) in cyclohexane (250 mL), NBS (29.79 g, 167.40 mmol) and AIBN (11.00 g, 66.96 mmol) were added.[4] The reaction mixture was purged with N_2 for 3 times, and then stirred at 80 ℃ for 8 h. After completion of the reaction, the mixture was filtered over celite and the filtrate was concentrated under reduced pressure to give the crude bromide compound which was used in the next step without further purification.

To a solution of the above bromide intermediate in 1,4-dioxane (250 mL)/water (150 mL), $CaCO_{3\,(s)}$ (15.30 g, 152.87 mmol) was added at room temperature. The reaction was stirred at 130 ℃ for 14 h. After completion of the reaction, the solution was filtered over celite and the filtrate was concentrated under reduced pressure. The obtain residue was dissolved in EtOAc (200 mL) and washed with water (100 mL) and brine (100 mL). The organic layer was dried over $MgSO_4$ and concentrated for flash column chromatographic purification (EtOAc/Hexanes = 1:4) to afford known **11** (16.22 g, 70% over two steps) as a pale yellow liquid.[4,5]

2.5 Synthesis of (2-Iodo-3-methoxy-5-methylphenyl)methanol 12

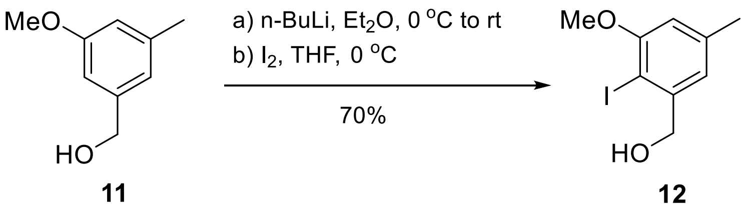

To a solution of **11** (11.38 g, 74.77 mmol) in Et_2O (250 mL), *n*-BuLi (1.6 M solution in hexanes, 93.60 mL, 149.5 mmol) was added at 0 °C under N_2. After being stirred at room temperature for 4 h, anhydrous THF (62 mL) was added to the solution and the mixture was stirred for additional 1 h, followed by the slow addition of I_2 solution (20.90 g, 82.36 mmol in 62 mL THF) at 0 °C. After being stirred at 0 °C for 1 h, the saturated $Na_2S_2O_3$ (150 mL) was poured to the reaction mixture. The organic phase was separated and washed with water (50 mL) and brine (150 mL), dried over anhydrous $MgSO_4$, and concentrated under reduced pressure. The crude product was purified by crystallization to afford known **12** (14.6 g, 70%) as a white solid.[5] Analytical data for **12**: R_f = 0.25 (25% EtOAc in hexanes); ^{1}H NMR (400 MHz, $CDCl_3$) δ: 6.90 (d, J = 0.8 Hz, 1H, H6), 6.58 (d, J = 0.8 Hz, 1H, H4), 4.64 (s, 2H, CH_2), 3.86 (s, 3H, OCH_3), 2.33 (s, 3H, CH_3 at C5); ^{13}C NMR (100 MHz, $CDCl_3$) δ: 157.8 (C3), 144.0 (C1), 139.7 (C5), 121.9 (C6), 111.2 (C4), 85.4 (C2), 69.6 (CH_2), 56.5 (OCH_3), 21.4 (CH_3).

2.6 Synthesis of 2-Iodo-3-methoxy-5-methylbenzyl acetate 12A

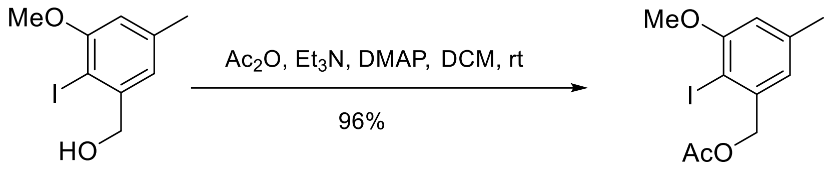

12 **12A**

To a solution of **12** (25.50 g, 91.43 mmol) in DCM (183 mL), Et_3N (38.35 mL, 275.18 mmol), DMAP (1.12 g, 9.17 mmol) and Ac_2O (11.27 mL, 119.24 mmol) were added at 0 °C. After stirring for 1 h, the reaction mixture was diluted with DCM (100 mL) and washed with 1 N aqueous HCl (150 mL), water (100 mL), and brine (50 mL). The organic layer was dried over anhydrous $MgSO_4$ and concentrated under reduced pressure for flash column chromatographic purification (13% EtOAc/Hexanes) to afford known **12A** (27.24 g, 92.7%) as a white solid.[5] Analytical data for **12A**: R_f = 0.43 (25% EtOAc in hexanes); ^{1}H NMR (400 MHz, $CDCl_3$) δ: 6.81 (s, 1H, H6), 6.60 (d, J = 0.8 Hz, 1H, H4), 5.11 (s, 2H, CH_2), 3.85 (s, 3H, OCH_3), 2.33 (s, 3H, CH_3), 2.13 (s, 3H, CH_3CO); ^{13}C NMR (100 MHz, $CDCl_3$) δ: 170.4 (CO), 157.9 (C3), 139.44 (C1), 139.41 (C5), 122.5 (C6), 111.5 (C4), 86.4 (C2), 70.2 (CH_2), 56.4 (OCH_3), 21.3 (CH_3), 20.8 ($\mathit{C}H_3CO$).

2.7 Synthesis of 3-Bromo-2-iodo-5-methylphenol 12B

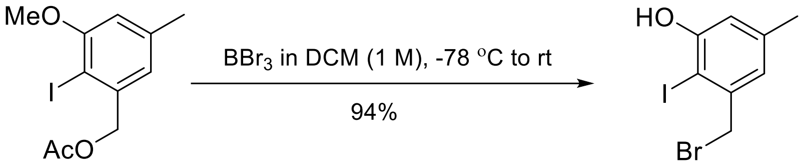

12A **12B**

To a solution of **12A** (7.22 g, 22.55 mmol) in anhydrous DCM (186 mL), a solution of BBr_3 in DCM (1.0 M, 46.58 mL, 46.58 mmol) was added slowly at −78 °C under N_2. The reaction mixture was stirred at –78 °C for 0.5 h, and then the reaction temperature was raised to room temperature and further stirred for 2 h. After completion of the reaction, saturated $NaHCO_3$ (250 mL) was added to quench the reaction. The mixture was diluted with DCM (75 mL) and washed with saturated $NaHCO_3$ (100 mL × 2), water (100 mL), and brine (50 mL). The organic layer was dried over anhydrous $MgSO_4$ and concentrated under reduced pressure for flash column chromatographic purification (Elution: 17% EtOAc in hexanes) to afford known **12B** (6.93 g, 94%) as a white solid.[5] Analytical data for **12B**: R_f = 0.38 (25% EtOAc in hexanes); ^{1}H NMR (400 MHz, $CDCl_3$) δ: 6.87 (d, *J* = 1.2 Hz, 1H, H4), 6.74 (s, *J* = 1.6 Hz, 1H, H6), 5.56 (s, 1H, OH at C1), 5.54 (s, 2H, CH_2), 2.27 (s 3H, CH_3 at C5); ^{13}C NMR (100 MHz, $CDCl_3$) δ: 155.3 (C1), 140.5 (C3), 140.4 (C5), 123.9 (C4), 115.8 (C6), 88.0 (C2), 39.2 (CH_2), 20.8 (CH_3 at C5).

2.8 Synthesis of 1-(Benzyloxy)-3-(bromomethyl)-2-iodo-5-methylbenzene 13

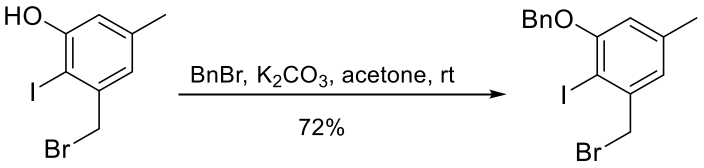

12B **13**

To a solution of **12B** (8.41 g, 25.72 mmol) in acetone (104 mL), K_2CO_3 (14.31 g, 103.52 mmol) and bromide (15.36 mL, 129.38 mmol) were added. The reaction mixture was stirred at room temperature for 6 h. After completion of the reaction, the reaction mixture was filtrated and the filtrate was concentrated under reduced pressure. The residue was dissolved in EtOAc (200 mL) and washed with water and brine. The organic layer was dried over anhydrous $MgSO_4$ and

concentrated under reduced pressure for flash column chromatographic purification (DCM/Hexanes = 1:19 gradient to 1:9) to afford known **13** (7.72 g, 72%) as a white solid.[5] Analytical data for **13**: R_f = 0.38 (20% DCM in hexanes); ^{1}H NMR (400 MHz, $CDCl_3$) δ: 7.52 – 7.23 (m, 5H), 6.93 (d, *J* = 0.8 Hz, 1H, H4), 6.57 (d, *J* = 1.2 Hz, 1H, H6), 5.08 (s, 2H, benzyl CH_2), 4.61 (s, 2H, CH_2), 2.27 (s, 3H, CH_3 at C5); ^{13}C NMR (100 MHz, $CDCl_3$) δ: 157.6 (C1), 141.5 (C3), 139.8 (C5), 136.4, 128.6, 127.9, 127.0, 124.0 (C4), 113.4 (C6), 89.3 (C2), 71.1, 39.6, 21.3 (CH_3 at C5).

2.9 Synthesis of 1-(Benzyloxy)-3-(but-3-yn-1-yl)-2-iodo-5-methylbenzene A ring alkyne 6

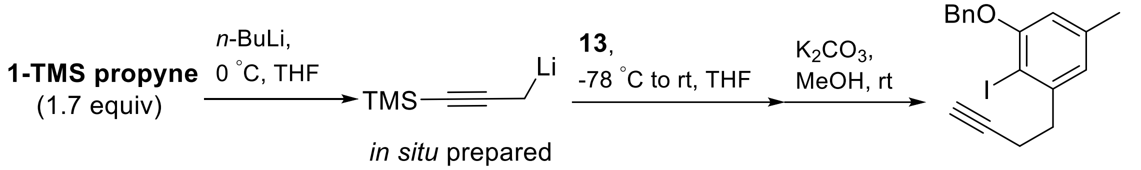

A ring alkyne (6)

(74% for two steps from **13**)

To a solution of TMS-propyne (4.70 mL, 31.74 mmol) in dry THF (53 mL), *n*-BuLi (1.6 M solution in hexanes, 17.49 mL, 27.98 mmol) was added slowly at 0 °C under N_2. The reaction mixture was stirred for 0.5 h, and transferred to a solution of **13** (7.74 g, 18.56 mmol) in dry THF (27 mL) at -78 °C. The resultant reaction mixture was stirred at –78 °C for 1 h, then the temperature was raised to room temperature and further stirred for 1 h. After completion of the reaction, 5% aqueous HCl (30 mL) was added to quench the reaction. The mixture was diluted with Et_2O (100 mL) and washed with water (100 mL), and brine (100 mL). The organic layer was dried over $MgSO_4$ and concentrated under reduced pressure to obtain the crude TMS-protected alkyne (TLC: R_f = 0.21, 20% DCM in hexanes), which was used in next step without column purification.

To a solution of above TMS-protected alkyne in MeOH (93 mL), K_2CO_3 (3.09 g, 22.38 mmol) was added at room temperature and the reaction mixture was stirred for 5 h at same temperature. The reaction was filtrated and the filtrate was evaporated under reduced pressure to remove methanol. The residue was dissolved in EtOAc (300 mL) and washed with water (100 mL) and brine (50 mL), dried over $MgSO_4$, and concentrated under reduced pressure. The crude product was purified by flash column chromatography (DCM/Hexanes = 1:19) to afford known A-ring

substituted alkyne **6** (5.18 g, 74%) as a pale yellow liquid.[4] Analytical data for **6**: R_f = 0.30 (10% DCM in hexanes); ^{1}H NMR (400 MHz, $CDCl_3$) δ: 7.49 (d, *J* = 7.2 Hz, 2H), 7.36 (t, *J* = 7.2 Hz, 2H), 7.29 (d, *J* = 7.2 Hz, 1H), 6.73 (s, 1H, H4), 6.52 (s, 1H, H6), 5.06 (s, 2H, benzyl CH_2), 2.97 (t, *J* = 7.6 Hz, 2H, CH_2), 2.47 (td, *J* = 7.6, 2.8 Hz, 2H, CH_2), 2.27 (s, 3H, CH_3 at C5), 1.97 (t, *J* = 2.8 Hz, 1H, CH of alkyne); ^{13}C NMR (100 MHz, $CDCl_3$) δ: 157.1 (C1), 144.2 (C3), 139.0 (C5), 136.6, 128.4, 127.7, 126.9, 123.4 (C4), 111.6 (C6), 89.1 (C2), 83.4, 70.9, 69.0, 39.9, 21.2, 19.1; HRMS (ESI): calcd for $C_{18}H_{18}IO^+$ $[M + H]^+$ 377.0397, found *m*/*z* 377.0396.

2.10 Synthesis of 5-Benzyloxy-2-(3-benzyloxy-2-iodo-5-methyl-phenethyl)-8-(methoxymethoxy)-naphthalene 14A and naphthalene-1,4-dione 14

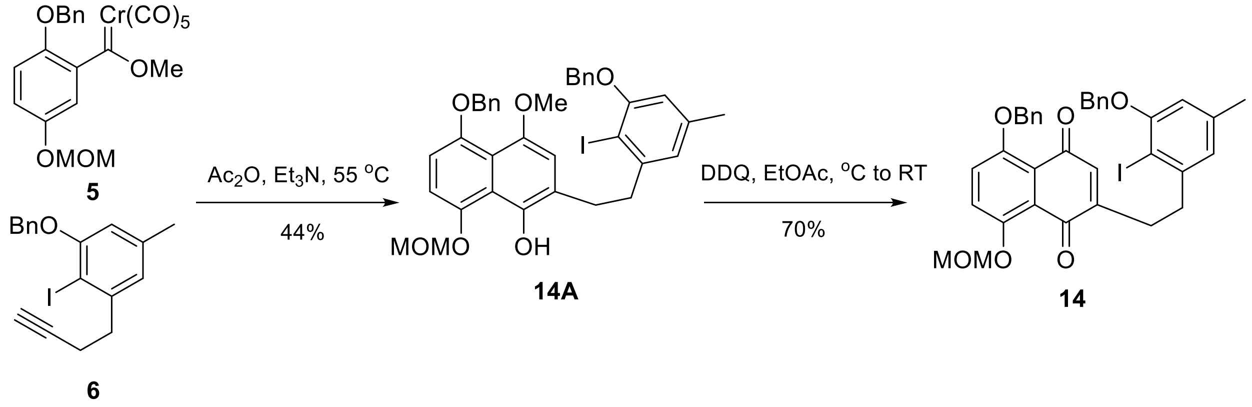

Pentacarbonylchromium **5** (12.82 g, 26.8 mmol) and alkyne **6** (8.41 g, 22.35 mmol) were dissolved in heptane (179 mL)/THF (45 mL), then Ac_2O (3.17 mL, 33.53 mmol) and Et_3N (4.64 mL, 33.53 mmol) were added to it at room temperature. The reaction mixture was purged with N_2 for 3 times and the mixture was stirred at 55 °C for 12 h. After completion of the reaction, the solution was concentrated and purified by flash column chromatography (EtOAc/hexanes = 9:1) to afford the naphthalene intermediate **14A** (6.75 g, 44%) as a yellow liquid. Due to the poor stability of **14A**, it was taken to subsequent oxidation without NMR characterization.

The above naphthalene **14A** was dissolved in EtOAc (195 mL) and DDQ (4.43 g, 19.55 mmol) was added at 0 °C. The reaction temperature was raised to room temperature. After stirring for 1 h at room temperature, saturated $NaHCO_3$ (150 mL) was poured into the reaction mixture. The organic phase was separated and washed with water and brine, dried over anhydrous $MgSO_4$,

and concentrated under reduced pressure. The crude product was purified by flash column chromatography (EtOAc/hexanes = 4:1) to afford naphthalene-1,4-dione **14** (4.58 g, 70%) as an orange amorphous solid. Analytical data for **14**: R_f = 0.23 (25% EtOAc in hexanes); ^{1}H NMR (400 MHz, $CDCl_3$) δ: 7.58-7.50 (m, 4H), 7.47-7.38 (m, 5H), 7.35-7.27 (m, 3H), 6.75 (d, *J* = 1.1 Hz, 1H, H6′), 6.66 (s, 1H, H3), 6.54 (d, *J* = 1.3 Hz, 1H, H4′), 5.27 (s, 2H, 5.24 (s, 2H), 5.12 (s, 2H), 3.56 (s, 3H), 3.02 (dd, *J* = 9.3, 6.5 Hz, 2H), 2.80 (dd, *J* = 9.5, 6.2 Hz, 2H), 2.26 (s, 3H, CH_3 at C5′); ^{13}C NMR (100 MHz, $CDCl_3$) δ: 184.9 (C=O), 184.4 (C=O), 157.2 (C5′), 153.4 (C5), 151.4 (C8), 149.4 (C2), 144.9 (C1′), 139.2 (C3′), 136.7, 136.3, 135.5 (C3), 128.6, 128.5, 127.9, 127.8, 127.0, 126.9, 125.2, 123.3 (C6′), 123.1, 122.0, 121.9, 111.5 (C4′), 96.2, 89.5 (C2′), 71.6, 71.0, 56.6, 39.4 (CH_2), 30.0 (CH_2), 21.2 (CH_3 at C5′); HRMS (ESI): calcd for $C_{15}H_{32}IO_6^+$ $[M + H]^+$ 675.1244, found *m*/*z* 675.1232.

2.11 Synthesis of 5-Benzyloxy-2-(3-benzyloxy-2-iodo-5-methyl-phenethyl)-8-(methoxymethoxy)naphthalene-1,4-diyl Diacetate 15

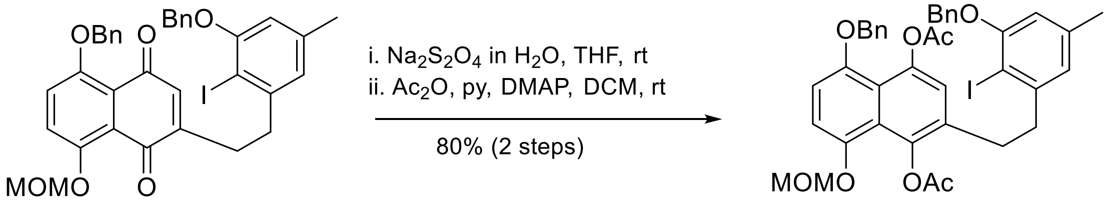

14 **15**

To a solution of naphthalene-1,4-dione **14** (1.83 g, 2.71 mmol) in THF (27 mL), aqueous $Na_2S_2O_4$ (1.89 g, 10.85 mmol in 27 mL water) was added at room temperature. After stirring at RT for 2 h, the reaction mixture was diluted with EtOAc (100 mL) and washed with water (50 mL × 2) and brine (60 mL), dried over $MgSO_4$, and concentrated under reduced pressure to afford the crude diol which was used for the next step without further purification.

To a solution of the above diol intermediate in DCM (27 mL), pyridine (13.5 mL, 168.02 mmol), Ac_2O (2.05 mL, 21.68 mmol) and DMAP (0.17 g, 1.36 mmol) were added at 0 °C. After stirring at room temperature for 3 h, the reaction mixture was diluted with DCM (20 mL) and washed with 5% aqueous HCl, saturated NH_4Cl, water and brine. The organic layer was separated, dried over anhydrous $MgSO_4$ and concentrated under reduced pressure. The crude product was

purified by flash column chromatography (EtOAc/Hexanes = 1:3) to afford **15** (1.65 g, 80% over two steps) as an oily yellow liquid. Analytical data for **15**: R_f = 0.47 (50% EtOAc in hexanes); ^{1}H NMR (400 MHz, $CDCl_3$) δ: 7.55 (d, *J* = 7.1 Hz, 2H), 7.50-7.45 (m, 2H), 7.44-7.32 (m, 6H), 7.04 (d, *J* = 8.8 Hz, 1H, H7), 7.03 (s, 1H, H3), 6.79 (d, *J* = 8.8 Hz, 1H, H6), 6.67 (d, *J* = 1.0 Hz, 1H, H6′), 6.55 (d, *J* = 1.2 Hz, 1H, H4′), 5.17 (d, *J* = 4.8 Hz, 2H, CH_2 in MOM), 5.14 (s, 2H, benzyl CH_2), 5.04 (d, *J* = 4.3 Hz, 2H, benzyl CH_2), 3.53 (s, 3H, CH_3 in MOM), 3.10-3.00 (m, 2H), 2.94-2.84 (m, 2H), 2.41 (s, 3H, CH_3CO), 2.27 (s, 3H, CH_3CO), 1.66 (s, 3H, CH_3 at C5′); ^{13}C NMR (100 MHz, $CDCl_3$) δ: 170.3 (C=O), 169.8 (C=O), 157.2 (C3′), 149.9 (C1′), 146.3 (C4), 145.6 (C5), 144.3 (C8), 141.4 (C5′), 139.3, 136.7, 136.5, 131.5, 128.8, 128.7, 128.5, 128.4, 127.8, 127.0, 123.4 (C6′), 122.4, 121.6 (C3), 120.7, 112.5 (C7), 111.4 (C4′), 107.3 (C6), 96.4 (CH_2 in MOM), 89.1 (C2′), 71.7 (benzyl CH_2), 71.0 (benzyl CH_2), 56.2 (CH_3 in MOM), 41.5, 30.8, 21.23 ($\mathit{C}H_3CO$), 21.17 ($\mathit{C}H_3CO$), 20.3 (CH_3 at C5′); HRMS (ESI): calcd for $C_{39}H_{41}INO_8^+$ $[M + NH_4]^+$ 778.1877, found *m*/*z* 778.1851.

2.12 Synthesis of 1,11-Bis(benzyloxy)-8-(methoxymethoxy)-3-methyl-5,6-dihydrotetraphene-7,12-diyl Diacetate 16

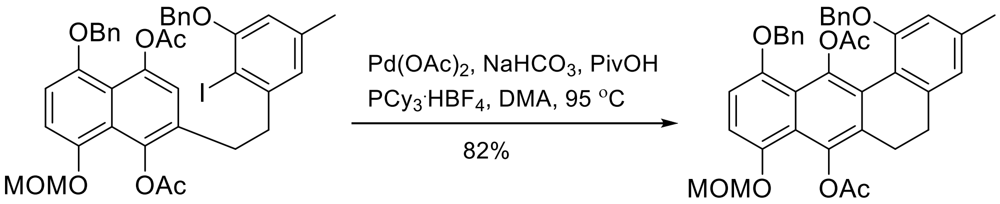

15 **16**

The flame dried glass pressure reactor was charged with naphthalene 1,4-diacetate **15** (0.81g, 1.06 mmol) and solid $NaHCO_3$ (0.54 g, 6.40 mmol), then anhydrous *N*,*N*-dimethylacetamide (36 mL) was added to the mixture. To the solution of **15**, a premix solution of $Pd(OAc)_2$ (0.12 g, 0.53 mmol), $PCy_3 \cdot HBF_4$ (0.39 g, 1.07 mmol) and PivOH (0.24 mL, 2.13 mmol) in DMA (12 mL) was added dropwise. After stirring at 95 °C for 18 h, the reaction mixture was diluted with EtOAc (100 mL) and washed with water (50 mL × 2) and brine (50 mL), dried over $MgSO_4$, and concentrated under reduced pressure. The obtained crude product was purified by flash column chromatography (EtOAc/Hexanes = 1:4) to afford **16** (0.55 g, 82%) as an oily yellow

liquid. **16** exists as a pair of atropisomers. Analytical data for **16**: R_f = 0.27 (33% EtOAc in hexanes); ^{1}H NMR (400 MHz, $CDCl_3$) δ: 7.51 (d, *J* = 7.4 Hz, 2H), 7.35-7.26 (m, 8H), 6.98 (d, *J* = 8.6 Hz, 1H), 6.78-6.71 (m, 3H), 5.23-5.03 (m, 6H), 3.54 (s, 3H, CH_3 MOM), 3.16-3.08 (m, 1H, CH_2 at C6), 2.75-2.59 (m, 2H, CH_2 at C5), 2.40 (s, 3H, CH_3CO), 2.34 (s, 3H, CH_3CO), 2.32-2.26 (m, 1H, CH_2 at C6), 1.52 (s, 3H, CH_3 at C3); ^{13}C NMR (100 MHz, $CDCl_3$) δ: 169.9 (C=O), 169.6 (C=O), 156.0, 149.9, 146.6, 142.6, 142.1, 141.0, 139.0, 138.8, 137.6, 136.8, 131.5, 128.43, 128.35, 128.31, 128.1, 128.0, 127.4, 126.9, 125.0, 121.1, 120.8, 118.3, 112.0, 111.3, 108.4, 107.4, 95.9 (CH_2 in MOM), 72.0 (benzyl CH_2), 70.2 (benzyl CH_2), 56.1 (CH_3 in MOM), 30.0 (C5), 23.6 (C6), 21.6 ($\mathit{C}H_3CO$), 20.9 ($\mathit{C}H_3CO$), 20.7 (CH_3 at C3); HRMS (ESI): calcd for $C_{39}H_{40}NO_8^+$ $[M + NH_4]^+$ 650.2754, found *m*/*z* 650.2740.

2.13 Synthesis of 1,11-Bis(benzyloxy)-8-(methoxymethoxy)-3-methyl-5,6-dihydrotetraphene-7,12-dione 16A

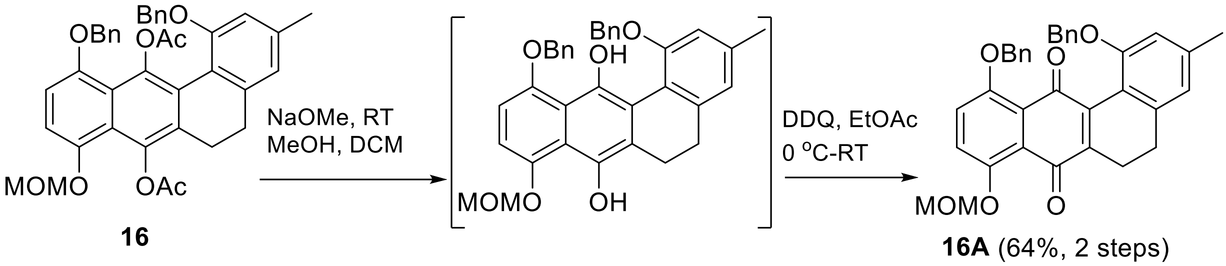

To a solution of **16** (0.82 g, 1.30 mmol) in DCM (44 mL) was added a solution of NaOMe (280.9 mg, 5.2 mmol) in MeOH (22 mL). After stirring at RT for 6 h, the reaction was quenched with saturated NH_4Cl (5 mL), then diluted with DCM (60 mL) and washed with water (50 mL) and brine (50 mL), dried over anhydrous $MgSO_4$, and concentrated under reduced pressure to give a crude hydroquinone intermediate.

To a solution of the above hydroquinone intermediate in EtOAc (26 mL), DDQ (0.32 g, 1.43 mmol) was added at 0 °C. After stirring at room temperature for 1 h, saturated $NaHCO_3$ (5 mL) was poured into the reaction mixture. EtOAc (100 mL) was added and the organic layer was washed with water (50 mL) and brine (30 mL), dried over anhydrous $MgSO_4$, and concentrated for flash column chromatographic purification (EtOAc/Hexanes = 1:9) to afford **16A** (0.45 g, 64%

over two steps) as an orange amorphous solid. Analytical data for **16A**: R_f = 0.42 (EtOAc/Hexanes = 1:2); ^{1}H NMR (400 MHz, $CDCl_3$) δ: 7.45 (d, *J* = 3.5 Hz, 2H), 7.37 (d, *J* = 9.3 Hz, 1H), 7.29-7.16 (m, 9H), 6.73 (s, 1H, H4), 6.63 (s, 1H, H2), 5.26 (s, 2H), 5.08 (s, 2H, benzyl CH_2), 5.04 (s, 2H, benzyl CH_2), 3.56 (s, 3H), 2.74 (s, 4H, CH_2 at C5 and C6), 2.30 (s, 3H, CH_3 at C3); ^{13}C NMR (100 MHz, $CDCl_3$) δ: 183.8 (C=O), 183.0 (C=O), 156.4 (C1), 152.0, 150.6, 144.0, 141.9, 141.0, 140.7, 137.4, 136.5, 128.3, 128.2, 127.6, 127.2, 126.9, 126.6, 124.7, 123.7, 123.2, 121.4 (C4), 120.8, 117.8, 113.0 (C2), 96.2 (CH_2), 71.4 (benzyl CH_2), 71.0 (benzyl CH_2), 56.5, 28.4 (C5), 21.9 (C6), 19.5 (CH_3 at C3); HRMS (ESI): calcd for $C_{35}H_{31}O_6^+$ $[M + H]^+$ 547.2121, found *m/z* 547.2115.

2.14 Synthesis of 1,11-Bis(benzyloxy)-8-hydroxy-3-methyl-5,6-dihydrotetraphene-7,12-dione 17

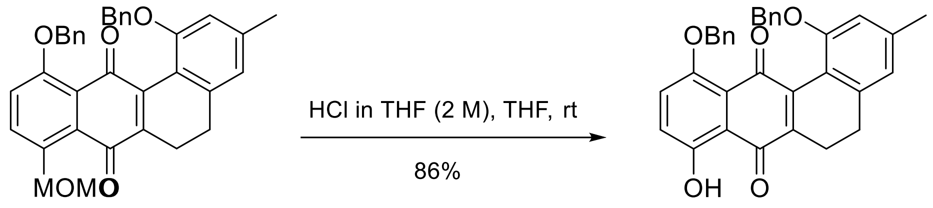

16A **17**

To a solution of **16A** (0.51 g, 0.93 mmol) in THF (94 mL), 8 mL 33% aqueous HCl was added and the reaction mixture was stirred for 7 h at room temperature. After completion of the reaction, the reaction mixture was diluted with EtOAc (250 mL) and washed with saturated $NaHCO_3$ (50 mL× 3), brine (50 mL) and water. The organic layer was separated, dried over anhydrous $MgSO_4$ and concentrated under reduced pressure. The obtained crude product was purified by flash column chromatography (EtOAc/Hexanes = 1:4) to afford **17** (0.40 g, 86%) as an orange amorphous solid. Analytical data for **17**: R_f = 0.5 (50% EtOAc in hexanes); ^{1}H NMR (400 MHz, $CDCl_3$) δ: 12.39 (s, 1H, OH at C8), 7.46-7.41 (m, 2H), 7.31-7.27 (m, 2H), 7.23-7.15 (m, 7H), 7.09 (d, *J* = 9.3 Hz, 1H, H10), 6.71 (s, 1H, H4), 6.67 (s, 1H, H2), 5.07 (s, 2H, benzyl CH_2), 5.04 (s, 2H, benzyl CH_2), 2.72 (s, 4H, CH_2 at C5 and C6), 2.30 (s, 3H, CH_3 at C3); ^{13}C NMR (100 MHz, $CDCl_3$) δ: 188.5 (C=O), 182.6 (C=O), 156.7 (C8), 156.1, 151.1, 146.5, 142.5, 141.5, 139.9, 137.1, 136.6, 128.3, 128.2, 127.6, 127.3, 127.0, 126.8, 125.2, 124.2 (C10), 121.7, 121.4 (C4),

117.9, 115.2, 113.0 (C2), 72.0 (benzyl CH_2), 71.0 (benzyl CH_2), 28.2 (C5), 21.9 (CH_3 at C3), 19.7 (C6); HRMS (ESI): calcd for $C_{33}H_{27}O_5^+$ $[M + H]^+$ 503.1858, found *m/z* 503.1863.

2.15 Synthesis of anhydrolandomycinone (1)

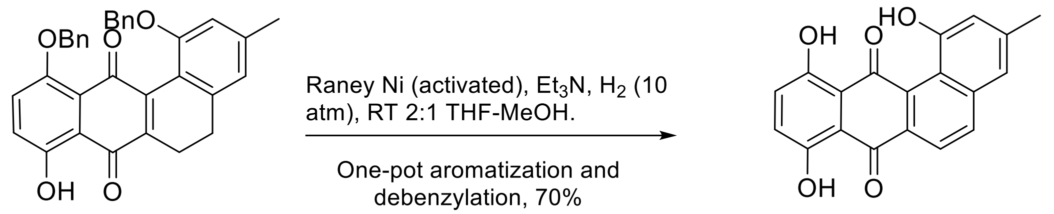

17 Anhydrolandomycinone (**1**)

To a solution of **17** (94.5 mg, 0.19 mmol) in the MeOH (3 mL)/THF (6 mL), activated Raney Ni (1.0 g) and Et_3N (0.16 mL, 1.14 mmol) were added. After stirring under H_2 (10 atm) for 17 h, the reaction mixture was filtered through celite. The filtrate was concentrated under reduced pressure for flash column chromatographic purification (EtOAc/DCM/hexanes = 0.2:0.8:1.5) to afford desired **anhydrolandomycinone (1)** (42.0 mg, 70%).[4] ^{1}H NMR (400 MHz, CDCl3) δ: 12.99 (s, 1H, O*H*), 12.49 (s, 1H, O*H*), 11.12 (s, 1H, O*H*), 8.33 (d, *J* = 8.4 Hz, 1H, Ar*H* at B ring), 8.14 (d, *J* = 8.8 Hz, 1H, Ar*H* at B ring), 7.34 (m, 2H, Ar*H* at D ring), 7.28 (s, 1H, Ar*H* at A ring), 7.17 (s, 1H, Ar*H* at A ring), 2.50 (s, 3H, C*H*$_3$ at C3 of A ring); ^{13}C NMR (100 MHz, $CDCl_3$) δ: 190.9 (C=O), 186.2 (C=O), 159.0 (C1), 158.2 (C11), 155.1 (C8), 142.0, 139.2, 138.3, 135.7, 131.6, 130.6, 130.4, 121.9, 121.7, 120.8, 120.1, 113.7, 111.3, 21.3 (*C*H$_3$ at C3). The ^{1}H and ^{13}C spectroscopic data are in full agreement with the reported data.[1]

2.16 Synthesis of B-ring unsaturated core analogue (2)

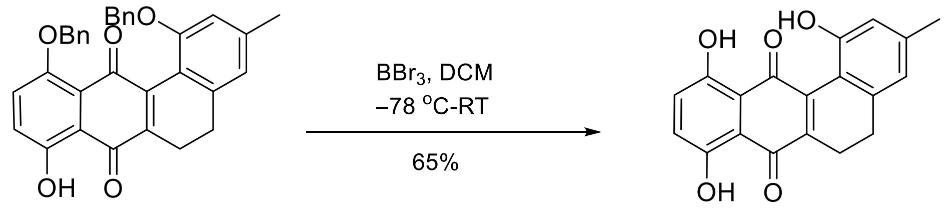

17 B-ring unsaturated core analogue (**2**)

To a stirred solution of **17** (20 mg, 0.04 mmol) in DCM (6 mL) was added BBr_3 (1 M solution in DCM, 0.1 mL, 0.1 mmol) at -78 °C. After stirring at -78 °C for 3 h and 30 mins at RT,

the reaction was diluted with DCM (50 mL) and quenched with water (2 mL). The organic layer was successively washed with water (25 mL), brine (25 mL) and dried over Mg_2SO_4. The crude residue was purified by flash column chromatography (EtOAc/DCM/hexanes = 0.2:0.8:1.5) to provide compound **2** (8.3 mg, 65%) as a brown solid. Analytical data for **2**: ^{1}H NMR (400 MHz, $CDCl_3$) δ: 12.98 (s, 1H), 12.76 (s, 1H), 8.89 (s, 1H), 7.32 (d, *J* = 9.6 Hz, Ar-H at D-ring), 7.27 (d, *J* = 9.6 Hz, Ar-*H* at D-ring), 6.80 (s, 1H, Ar-*H* at A-ring), 6.69 (s, 1H, Ar-*H* at A-ring), 2.84-2.80 (m, 2H, C*H*$_2$ at B-ring), 2.72-2.69 (m, 2H, C*H*$_2$ at B-ring); ^{13}C NMR (100 MHz, $CDCl_3$) δ: 187.0, 183.9, 161.3, 160.1, 155.1, 148.9, 143.2, 141.4, 140.7, 131.6, 130.5, 121.7, 119.8, 115.5, 112.1, 110.9, 77.2, 28.4, 21.7, 21.2. HRMS (ESI): calcd for $C_{19}H_{15}O_5^+$ $[M + H]^+$ 323.0919, found *m*/*z* 323.0914.

2.17 Synthesis of 1,11-Bis(benzyloxy)-8-hydroxy-3-methyltetraphene-7,12-dione 18

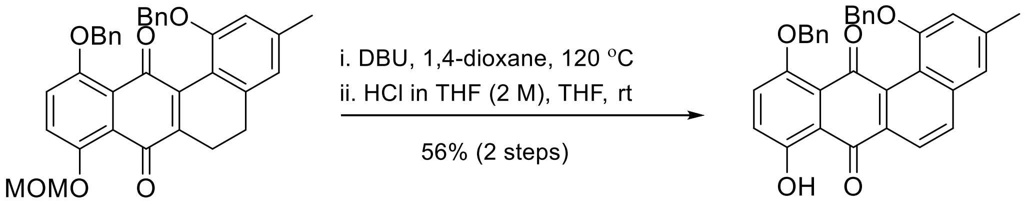

16A (from **16**, please refer 2.13) **18**

To a thick glass pressure reactor (150 mL) were charged with compound **16A** (0.42 g, 0.77 mmol), 1,4-dioxane (38 mL) and DBU (1.70 mL, 11.39 mmol) at room temperature. After stirring at 120 °C for 16 h, the reaction was cooled to room temperature. Then, the reaction mixture was diluted with EtOAc (100 mL) and neutralized (pH = 7.0) by the addition of 0.5 N HCl. The mixture was washed with water (50 mL × 2) and brine (50 mL). The organic layer was separated, dried over anhydrous Na_2SO_4 and concentrated under reduced pressure. The crude product thus obtained was used for the next reaction without purification.

To a solution of the above compound in THF (76 mL), 6.5 mL 33% aqueous HCl was added (Final [HCl] is ~ 1 N) at room temperature. After stirring at room temperature for 8 h, the reaction mixture was diluted with EtOAc (100 mL) and washed with saturated $NaHCO_3$ (100 mL× 2), water (50 mL) and brine (50 mL). The organic layer was separated, dried over anhydrous $MgSO_4$, and concentrated under reduced pressure. The obtained crude product was purified by

flash column chromatography (EtOAc/Hexanes = 1:9) to afford **18** (0.23 g, 56% over two steps) as an orange amorphous solid. Analytical data for **18**: R_f = 0.35 (20% EtOAc in hexanes); ^{1}H NMR (400 MHz, $CDCl_3$) δ: 12.22 (s, 1H, OH at C8), 8.17 (d, *J* = 8.6 Hz, 1H, H5), 7.88 (d, *J* = 8.6 Hz, 1H, H6), 7.47 (d, *J* = 6.9 Hz, 2H), 7.42 (d, *J* = 7.1 Hz, 2H), 7.33 –7.27 (m, 3H), 7.25-7.22 (m, 2H), 7.17-7.08 (m, 4H), 6.78 (d, *J* = 1.0 Hz, 1H, H2), 5.18 (s, 2H, benzyl CH_2), 5.11 (s, 2H, benzyl CH_2), 2.42 (s, 3H, CH_3 at C3); ^{13}C NMR (100 MHz, $CDCl_3$) δ: 188.2 (C=O), 185.7 (C=O), 156.4 (C8), 156.1, 150.0, 141.0, 139.2, 138.6, 137.2, 136.6, 131.8, 131.4, 128.5, 128.3, 127.7, 127.5, 127.2, 126.5, 125.3, 125.0, 123.2, 121.6, 120.2, 119.6, 116.0, 112.7, 72.2 (benzyl CH_2), 71.1 (benzyl CH_2), 22.2 (CH_3 at C3); HRMS (ESI): calcd for $C_{33}H_{25}O_5^+$ $[M + H]^+$ 501.1702, found *m/z* 501.1704.

Scheme S1. Preparation of 4-*O*-Benzoyl-2,3,6-trideoxy-L-rhodinose Acetate 19

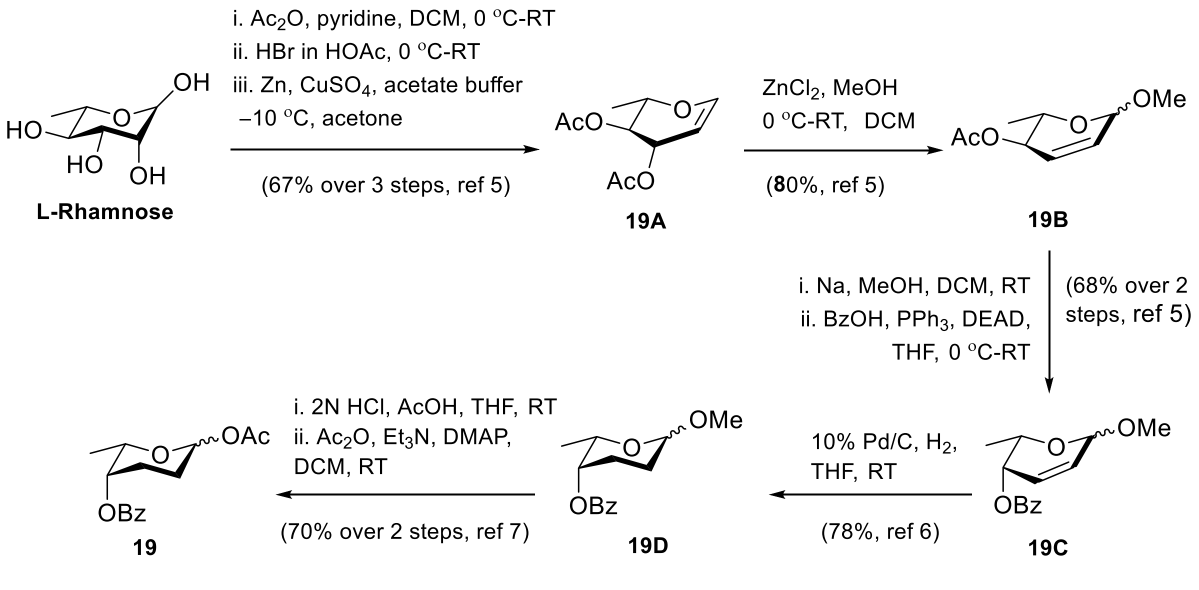

2.18 Synthesis of 3,4-Di-O-acetyl-1,5-anhydro-2,6-dideoxy-L-arabino-hex-1-enol 19A

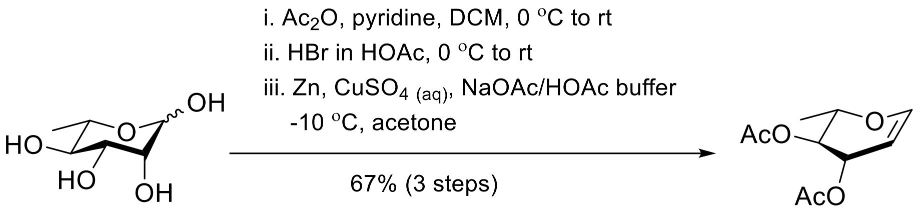

19A

To a solution of L-rhamnose (20.00 g, 121.95 mmol) in DCM (61 mL) and pyridine (61 mL), was added Ac_2O (57.59 mL, 609.76 mmol) at 0 ℃. After stirring at room temperature for 5 h, the reaction mixture was diluted with DCM (200 mL) and quenched with 5% aqueous HCl, washed with water (100 mL), brine (100 mL), dried over $MgSO_4$, and filtered. The filtrate was concentrated to give the crude tetraacetate which was used in the next reaction without further purification.

To a solution of the above tetra-acetate compound in DCM (244 mL), a solution of HBr in acetic acid (33 wt%, 88 mL, 487.80 mmol) was added at 0 ℃. After stirring at room temperature for 2 h, the reaction mixture was quenched with ice cool saturated NaOH under ice bath, then diluted with DCM (50 mL). The DCM solution was washed with water (100 mL), brine (100 mL), dried over $MgSO_4$, and filtered. The filtrate was concentrated to give the crude glycal, which used in the next step directly.

Sodium acetate/HOAc buffer solution (122 mL) was prepared by adding NaOAc (50 g, 609.75 mmol) to HOAc (40.65 mL, 707.31 mmol). To this buffer solution, activated zinc powder (39.60 g, 609.75 mmol) and $CuSO_4$ solution (0.5 M in water, 24 mL) were added at -10 ℃. The solution was stirred until the blue color of the solution and bubbling disappeared. Then a solution of the above obtained glycal in acetone (244 mL) was added at -10 ℃ and the reaction mixture was stirred for 2 h at the same temperature. Then, it was filtered over celite, the filtrate was concentrated under reduced pressure. The residual syrup was diluted with EtOAc (200 mL) and washed with saturated NH_4Cl (100 mL × 2), brine (100 mL), dried over $MgSO_4$, and filtered. The filtrate was concentrated for flash column chromatographic purification (EtOAc/Hexanes = 1:7) to afford **19A** (26.9 g, 67% over 3 steps) as a colorless liquid.[6] Analytical data for **19A**: R_f = 0.58

(50% EtOAc in hexanes); ^{1}H NMR (400 MHz, $CDCl_3$) δ: 6.42 (dd, J = 6.1, 1.4 Hz, 1H, H1), 5.33 (dddd, J = 6.1, 3.0, 1.4, 0.5 Hz, 1H), 5.02 (dd, J = 8.2, 6.1 Hz, 1H), 4.77 (dd, J = 6.1, 3.0 Hz, 1H), 4.10 (qd, J = 7.3, 3.8 Hz, 1H), 2.07 (s, 3H, CH_3CO), 2.03 (s, 3H, CH_3CO), 1.30 (d, J = 6.6 Hz, 3H, H6).

2.19 Synthesis of Methyl-4-*O*-acetyl-2,3,6-trideoxy-L-erythro-hex-2-enopyranoside 19B

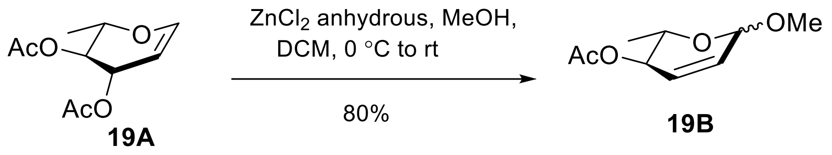

To a solution of **19A** (20.88 g, 97.56 mmol) in DCM (195 mL), MeOH (5.90 mL, 146.34 mmol) and $ZnCl_2$ (3.92 g, 29.27 mmol) were added at 0 ℃. After stirring at room temperature for 3 h, the reaction was quenched with saturated $NaHCO_3$. The organic layer was diluted with DCM (50 mL) and washed with saturated $NaHCO_3$ (50 mL), water (50 mL), brine (50 mL), dried over $MgSO_4$ and filtered. The filtrate was concentrated for flash column chromatographic purification (EtOAc/Hexanes = 1:9) to afford **19B** (14.54 g, 80%) as a α/β-anomeric mixture.[6] Analytical data for α-anomer **19B**: R_f = 0.45 (20% EtOAc in hexanes); ^{1}H NMR (400 MHz, $CDCl_3$) δ: 5.86-5.76 (m, 2H, H2 and H3), 5.06-5.02 (m, 1H, H1), 4.84 (s, 1H, H4), 3.93 (dq, J = 9.2, 6.3 Hz, 1H, H5), 3.42 (s, 3H, OCH_3), 2.07 (s, 3H, CH_3CO), 1.22 (d, J = 6.3 Hz, 3H, H6).

2.20 Synthesis of Methyl-4-*O*-benzoyl-2,3,6-trideoxy-L-erythro-hex-2-enopyranoside 19C

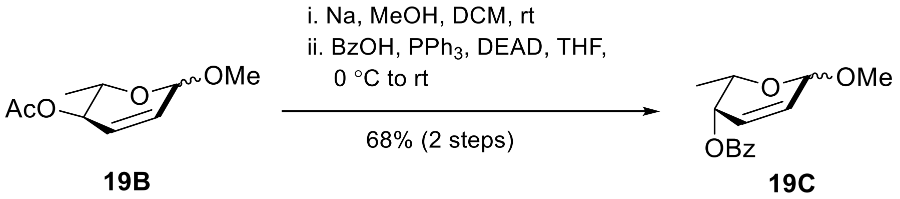

To a solution of **19B** (5.25 g, 28.23 mmol) in DCM (28 mL)/MeOH (11 mL), sodium was added. After stirring at room temperature for 2 h, the reaction mixture was quenched with saturated NH_4Cl, then the mixture was diluted with DCM (100 mL) and washed with saturated NH_4Cl (50 mL × 2), water (50 mL), brine (50 mL), dried over $MgSO_4$, and filtered. The filtrate was concentrated to give the crude product for the next reaction.

To a solution of the above obtain compound in THF (56 mL), benzoic acid (6.90 g, 56.46 mmol), PPh_3 (14.80 g, 56.46 mmol) and a solution of DEAD (9.80 g, 56.46 mmol, in THF 56 mL) were added at 0 °C. After stirring at room temperature for 3 h, the solvent was removed under reduced pressure and the resulting residue was suspended in Et_2O and stirred for 1 h. The suspension was filtrated and the filtrate was concentrated under reduced pressure for flash column chromatographic purification (EtOAc/Hexanes = 1:19) to afford **19C** (4.75 g, 68% over two steps) as a α/β-anomeric mixture.[6] Analytical data for *α*-anomer **19C**: R_f = 0.45 (20% EtOAc in hexanes); ^1H NMR (400 MHz, $CDCl_3$) δ: 8.08 (dd, *J* = 8.3, 1.2 Hz, 2H), 7.56 (td, *J* = 7.6, 1.2 Hz, 1H), 7.44 (t, *J* = 8.0 Hz, 2H), 6.20 (dd, *J* = 10.0, 5.4 Hz, 1H, H2), 6.06 (dd, *J* = 10.0, 3.1 Hz, 1H, H3), 5.15 (dd, *J* = 5.4, 2.5 Hz, 1H, H1), 4.98 (d, *J* = 3.0 Hz, 1H, H4), 4.33 (qd, *J* = 6.6, 2.5 Hz, 1H, H5), 3.47 (s, 3H, OCH_3), 1.32 (d, *J* = 6.6 Hz, 3H, H6).

2.21 Synthesis of Methyl-4-*O*-benzoyl-2,3,6-trideoxy-L-threo-hexopyranoside 36D

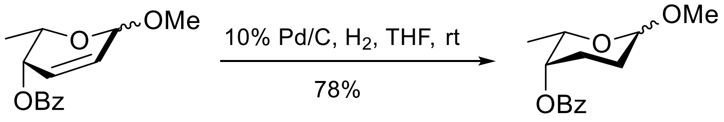

19C **19D**

To a solution of **19C** (2.68 g, 10.81 mmol) in THF (22 mL), Pd (10% on charcoal, 0.13 g) was added under nitrogen atmosphere. Then the H_2 gas was purged into the reaction apparatus. After stirring at room temperature for 12 h, it was filtered by celite. The filtrate was concentrated under reduced pressure for flash column chromatographic purification (EtOAc/Hexanes = 1:19) to afford **19D** (2.10 g, 78%) as a α/β-anomeric mixture.[7] Analytical data for α-anomer **36D**: R_f = 0.35 (10% EtOAc in hexanes); ^1H NMR (400 MHz, $CDCl_3$) δ: 8.11 (dd, *J* = 8.4, 1.6 Hz, 2H), 7.56 (td, *J* = 7.1, 1.6 Hz, 1H), 7.45 (t, *J* = 8.4 Hz, 2H), 5.05 (s, 1H, H1), 4.80 (d, *J* = 3.1 Hz, 1H, H4), 4.09 (qd, *J* = 6.4, 1.2 Hz, 1H, H5), 3.40 (s, 3H, OCH_3), 2.15 (tdd, *J* = 13.7, 4.3, 2.8 Hz, 1H), 2.01 (tt, *J* = 13.6, 4.0 Hz, 1H), 1.94-1.87 (m, 1H), 1.63-1.57 (m, 1H), 1.19 (d, *J* = 6.6 Hz, 3H, H6).

2.22 Synthesis of 1-*O*-Acetyl-4-*O*-benzoyl-2,3,6-trideoxy-L-rhodinose 19

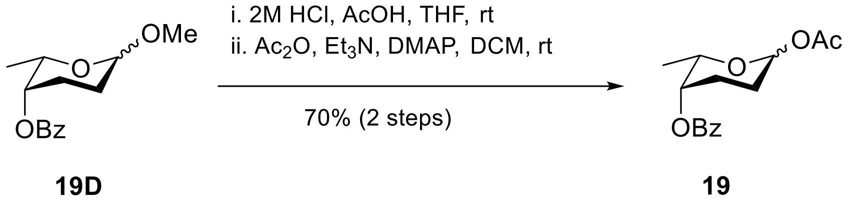

To a solution of **19D** (1.49 g, 5.96 mmol) in THF (6 mL), AcOH (6 mL) in 2 M aqueous HCl (6 mL) was added at 0 °C. After stirring at room temperature for 2 h, the reaction mixture was quenched with saturated $NaHCO_3$, then the mixture was diluted with EtOAc (50 mL) and washed with water (50 mL), brine (50 mL), dried over $MgSO_4$, and filtered. The filtrate was concentrated to give the crude hemiacetal compound which was used for the next step without further purification.

To a solution of the above hemiacetal compound in DCM (30 mL), Et_3N (1.32 mL, 9.54 mmol), Ac_2O (0.73 mL, 7.75 mmol) and DMAP (0.15 g, 1.19 mmol) were added at 0 °C. After stirring at room temperature for 1 h, the reaction mixture was diluted with DCM (15 mL) and washed with saturated NH_4Cl (50 mL × 2), water (50 mL), brine (50 mL), dried over $MgSO_4$, and filtered. The filtrate was concentrated for flash column chromatographic purification (EtOAc/Hexanes = 1:9) to afford **19** (1.16 g, 70% over two steps) a α/β-anomeric mixture.[8] Analytical data for α-anomer **19**: R_f = 0.25 (16% EtOAc in hexanes); ^{1}H NMR (400 MHz, $CDCl_3$) δ: 8.10 (t, *J* = 8.0 Hz, 2H), 7.58 (t, *J* = 7.4 Hz, 1H), 7.45 (t, *J* = 7.6 Hz, 2H), 6.24 (s, 1H, H1), 5.11 (s, 1H, H4), 4.21 (q, *J* = 6.4 Hz, 1H, H5), 2.18-2.10 (m, 5H), 2.06-1.99 (m, 1H), 1.69-1.63 (m, 1H), 1.19 (d, *J* = 6.4 Hz, 3H, H6).

Scheme S2. Synthesis of 1,4-Di-*O*-acetyl-3-*O*-*tert*-butyldimethylsilyl-2,6-dideoxy-2-iodo-*β*-D-glucopyranoside 22 and 1,4-Di-O-acetyl-2,6-dideoxy-2-iodo-*β*-D-glucopyranoside 20

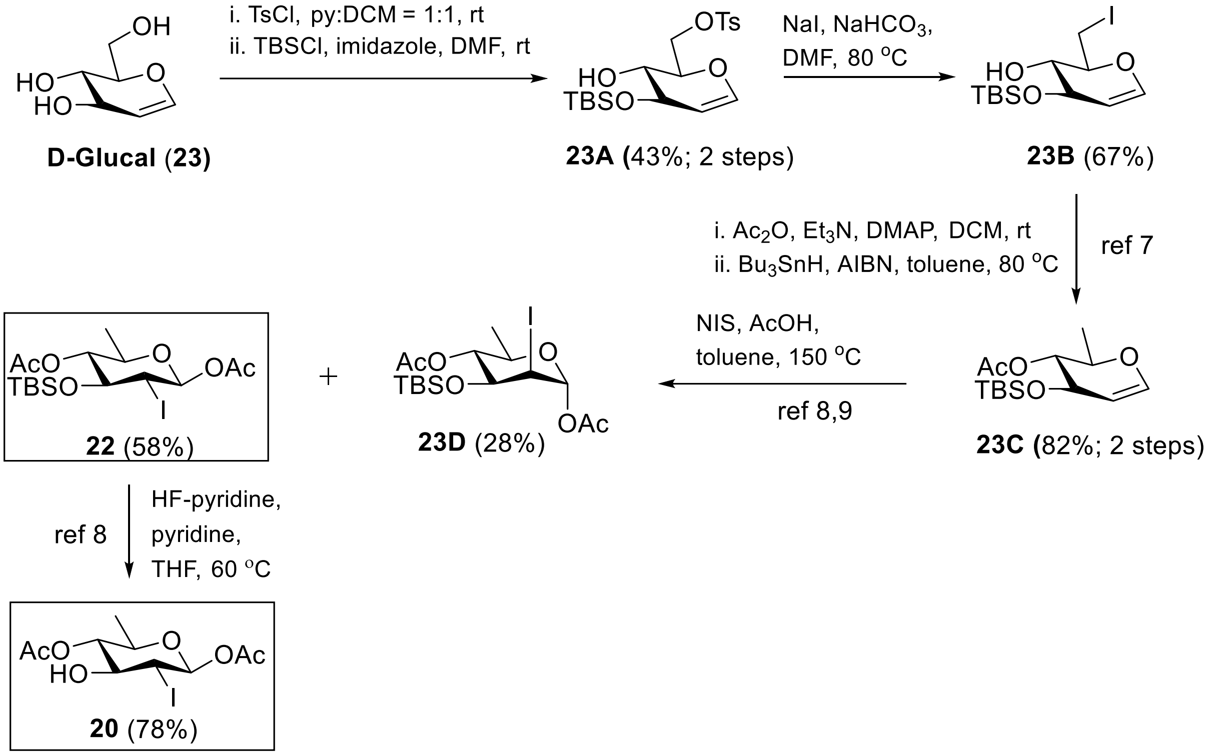

2.23 Synthesis of 3-*O*-*tert*-Butyldimethylsilyl-6-iodo-D-glucal 23B

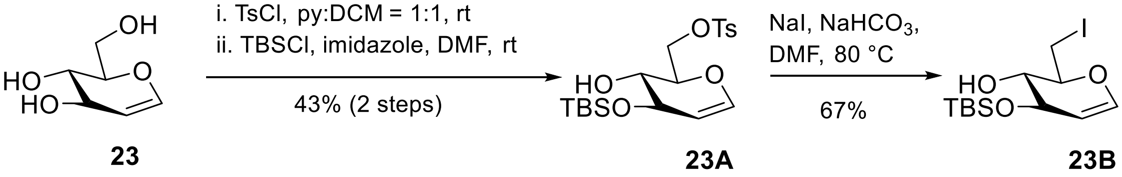

To a solution of D-glucal **23**[9] (3.83 g, 26.23 mmol) in DCM (13 mL), pyridine (13 mL) and TsCl (5.98 g, 31.48 mmol) were added at 0 ℃. After stirring at room temperature for 1 h, the reaction mixture was diluted with DCM (15 mL), washed with water (30 mL), dried over $MgSO_4$, and filtered. The filtrate was concentrated to give the crude tosyl compound which was used in the next step directly.

To a solution of the above obtained tosyl compound in DMF (26 mL), imidazole (3.92 g, 57.71 mmol) and TBSCl (4.33 g, 28.86 mmol) were added. After stirring at room temperature for 1.5 h, the reaction mixture was quenched with 5% aqueous HCl, then the reaction mixture was diluted with EtOAc (90 mL). The organic layer was separated and washed with water (100 mL × 3), brine (100 mL), dried over $MgSO_4$, and filtered. The filtrate was concentrated under reduced pressure for flash column chromatographic purification (EtOAc/Hexanes = 1:6) to afford known **23A** (4.05 g, 43% over two steps) as a colorless liquid.[10]

To a solution of **23A** (2.92 g, 8.07 mmol) in DMF (27 mL), $NaHCO_{3\,(s)}$ (2.03 g, 24.20 mmol) and NaI (6.05 g, 40.30 mmol) were added. After stirring at 80 °C for 16 h, water (50 mL) was poured into the reaction mixture followed by dilution with DCM (100 mL). The organic layer was separated and washed with water (50 mL × 4), brine (50 mL), dried over $MgSO_4$ and concentrated for flash column chromatographic purification (EtOAc/Hexanes = 1:13) to afford **23B** (2.0 g, 67%) as a colorless liquid. Analytical data for **23B**: R_f = 0.57 (20% EtOAc in hexanes); ^1H NMR (400 MHz, $CDCl_3$) δ: 6.29 (d, *J* = 6.0 Hz, 1H, H1), 4.67 (dd, *J* = 6.1, 2.0 Hz, 1H), 4.24 (d, *J* = 6.0 Hz, 1H), 3.73 – 3.63 (m, 2H), 3.59 – 3.48 (m, 2H), 2.31 (d, *J* = 4.1 Hz, 1H), 0.90 (s, 9H, CH_3 of TBS), 0.11 (s, 6H, CH_3 of TBS); ^{13}C NMR (100 MHz, $CDCl_3$) δ: 143.0 (C1), 103.7 (C2), 76.1, 72.9, 69.6, 25.8, 18.1, 6.0 (C6), -4.4, -4.5. HRMS (ESI): calcd for $C_{12}H_{23}INaO_3Si^+$ $[M + Na]^+$ 393.0359, found *m*/*z* 393.0355.

2.24 Synthesis of 4-*O*-Acetyl-3-*O*-*tert*-butyldimethylsilyl-6-deoxy-D-glucal 23C

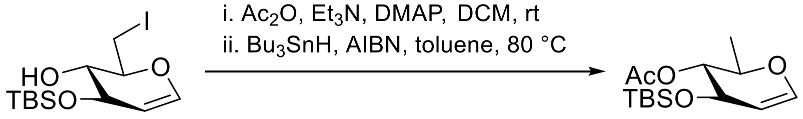

23B

23C (82%; 2 steps)

To a solution of **23B** (2.68 g, 7.24 mmol) in DCM (36 mL), Et_3N (1.60 mL, 11.58 mmol), Ac_2O (0.89 mL, 9.41 mmol) and DMAP (0.18 g, 1.45 mmol) were added at 0 °C. After stirring at room temperature for 1.5 h, the reaction mixture was diluted with DCM (20 mL) and washed with saturated NH_4Cl (50 mL × 2), water (50 mL) and brine (50 mL). The organic layer was separated,

dried over $MgSO_4$ and concentrated under reduced pressure to obtain the crude acetate, which was used in the next reaction without further purification.

To a solution of the above obtained acetate in toluene (72 mL), Bu_3SnH (7.68 mL, 28.96 mmol) and AIBN (0.36 g, 2.17 mmol) were added, then the reaction mixture was purged with N_2 (3 times). The deoxygenated mixture was stirred at 80 °C for 2 h, then the reaction mixture was cooled down to room temperature. The reaction was concentrated for flash column chromatographic purification (DCM/Hexanes = 1:4) to afford **23C** (1.70 g, 82% over two steps) as a colorless liquid.[11] Analytical data for **23C**: R_f = 0.33 (50% DCM in hexanes); ^{1}H NMR (400 MHz, $CDCl_3$) δ: 6.27 (dd, *J* = 6.2, 1.2 Hz, 1H, H1), 4.88 (dd, *J* = 8.2, 6.2 Hz, 1H, H4), 4.64 (dd, *J* = 6.2, 2.8 Hz, 1H, H2), 4.23 (ddd, *J* = 6.0, 2.3, 1.4 Hz, 1H, H3), 3.98 (dd, *J* = 8.0, 6.7 Hz, 1H, H5), 2.06 (s, 3H, CH_3CO), 1.26 (d, *J* = 6.6 Hz, 3H, H6), 0.85 (s, 9H, CH_3 of TBS), 0.05 (s, 3H, CH_3 of TBS), 0.04 (s, 3H, CH_3 of TBS); ^{13}C NMR (100 MHz, $CDCl_3$) δ: 169.7, 143.4 (C1), 103.2 (C2), 74.9 (C4), 72.5 (C5), 66.7 (C3), 25.5, 21.0, 17.8, 16.7 (C6), -4.6, -5.0.

2.25 Synthesis of 1,4-Di-*O*-acetyl--3-*O*-*tert*-butyldimethylsilyl-2,6-dideoxy-2-iodo-*β*-D-glucopyranoside 22

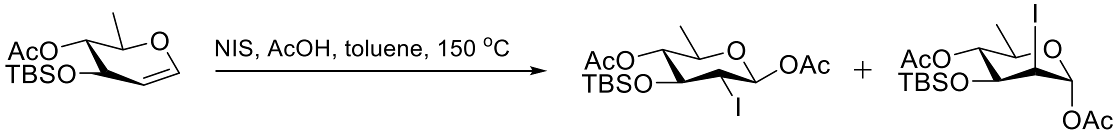

23C **22 (58%)** **23D (28%)**

To a solution of **39** (2.00 g, 6.98 mmol) in toluene (70 mL), AcOH (1.6 mL, 27.93 mmol) and NIS (4.71 g, 20.95 mmol) were added and the reaction was refluxed for 15 min. Then diluted with EtOAc (100 mL) and washed with saturated $Na_2S_2O_3$ (200 mL × 2) and brine (200 mL). the organic layer was separated, dried over $MgSO_4$, and concentrated under reduced pressure for flash column chromatographic purification (EtOAc/Hexanes = 1:19) to afford **22** (1.90 g, 58%) and **23D** (0.92 g, 28%) as a pale yellow liquid.[10] Analytical data for **22**: R_f = 0.50 (20% EtOAc in hexanes); ^{1}H NMR (400 MHz, $CDCl_3$) δ: 5.81 (d, *J* = 9.5 Hz, 1H, H1), 4.70 (dd, *J* = 9.7, 8.5 Hz, 1H, H4), 3.95 (dd, *J* = 10.0, 8.5 Hz, 1H, H3), 3.86 (t, *J* = 9.7 Hz, 1H, H2), 3.55 (ddd, *J* = 9.7, 6.4, 3.3 Hz,

1H, H5), 2.12 (s, 3H, CH_3CO), 2.08 (s, 3H, CH_3CO), 1.16 (d, J = 6.2 Hz, 3H, H6), 0.88 (s, 9H), 0.26 (s, 3H), 0.03 (s, 3H); ^{13}C NMR (100 MHz, $CDCl_3$) δ: 169.7, 168.8, 94.0 (C1), 76.5 (C4), 76.1 (C5), 71.2 (C3), 33.7 (C2), 26.1, 21.5, 20.8, 18.2, 17.5 (C6), -3.0, -3.5; Analytical data for **23D**: R_f = 0.45 (20% EtOAc in hexanes); ^1H NMR (400 MHz, $CDCl_3$) δ: 6.34 (d, J = 1.5 Hz, 1H, H1), 5.03 (t, J = 9.2 Hz, 1H, H4), 4.25 (dd, J = 4.2, 1.6 Hz, 1H, H2), 3.91 (dq, J = 9.6, 6.3 Hz, 1H, H5), 3.26 (dd, J = 8.8, 4.3 Hz, 1H, H3), 2.12 (s, 3H, CH_3CO), 2.07 (s, 3H, CH_3CO), 1.21 (d, J = 6.3 Hz, 3H, H6), 0.90 (s, 9H, CH_3 of TBS), 0.09 (s, 3H, CH_3 of TBS), 0.07 (s, 3H, CH_3 of TBS).

2.26 Synthesis of 1,4-Di-*O*-acetyl-2,6-dideoxy-2-iodo-*β*-D-glucopyranoside 20

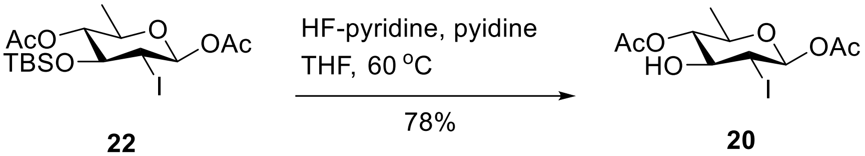

22 **20**

A PTFE flask was charged with **22** (1.36 g, 2.88 mmol), then THF (36 mL), pyridine (26 mL), HF-pyridine (4.08 mL, 28.80 mmol) were added. After stirring at 60 °C for 8 h, the reaction mixture was diluted with EtOAc (120 mL). The organic layer was washed with saturated $NaHCO_3$ (150 mL), water (50 mL), brine (50 mL), dried over $MgSO_4$, and filtered. The filtrate was concentrated under reduced pressure for flash column chromatographic purification (EtOAc/Hexanes = 1:3) to afford **20** (0.81 g, 78%) as a white solid.[10] Analytical data for **20**: R_f = 0.47 (50% EtOAc in hexanes); ^1H NMR (400 MHz, $CDCl_3$) δ: 5.80 (d, J = 9.3 Hz, 1H, H1), 4.65 (t, J = 9.3 Hz, 1H, H4), 3.91 (t, J = 9.6 Hz, 1H, H2), 3.81 (t, J = 8.8 Hz, 1H, H3), 3.65 (dq, J = 12.3, 6.1 Hz, 1H, H5), 3.10 (s, 1H, OH at C3), 2.13 (s, 3H, CH_3CO), 2.10 (s, 3H, CH_3CO), 1.21 (d, J = 6.2 Hz, 3H, H6); ^{13}C NMR (100 MHz, $CDCl_3$) δ: 170.7, 168.9, 93.7 (C1), 75.9 (C3), 75.3 (C4), 71.1 (C5), 33.1 (C2), 20.9, 20.7, 17.2 (C6).

2.27 Synthesis of 3-*O*-Acetyl-6-iodo-D-glucal 25

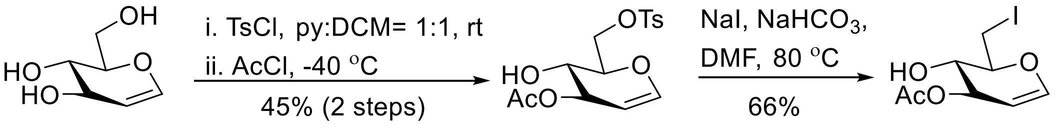

23 **24** **25**

To a solution of D-glucal **23** (4.74 g, 32.43 mmol) in DCM (32 mL), pyridine (32 mL) and TsCl (9.28 g, 48.65 mmol) were added at 0 °C. After stirring at room temperature for 1 h, the solvents were evaporated under reduced pressure and the residue was dissolved in DCM (120 mL) and washed with water (60 mL), and brine (50 mL). The organic layer was separated, dried over anhydrous $MgSO_4$, and concentrated under reduced pressure to afford the crude tosyl compound which was used for the next reaction without further purification.

To a solution of the above obtained tosyl compound in DCM (65 mL)/pyridine (20 mL), a solution of AcCl (1.38 mL, 19.46 mmol in 6 mL DCM) was added at -40 °C and the reaction mixture was stirred for 2 h at -40 °C. The reaction mixture was diluted with DCM (120 mL) and washed with aqueous $CuSO_4$ (50 mL × 4), water (50 mL), brine (50 mL), dried over anhydrous $MgSO_4$, and concentrated under reduced pressure. The crude product was purified by flash column chromatography (EtOAc/Hexanes = 1:4) to afford the acyl **24** (4.03 g, 45% over two steps) as a colorless liquid. This acyl **24** compound was used for the next reaction without further characterization.[12]

To a solution of **24** (2.53 g, 7.38 mmol) in DMF (30 mL), $NaHCO_3$ (2.29 g, 27.26 mmol) and NaI (6.81 g, 45.45 mmol) were added at room temperature. After stirring at 80 °C for 15 h, the mixture was cooled to room temperature, diluted with DCM (150 mL) and washed successively with water (50 mL × 4) and brine (50 mL). The organic layer was separated, dried over anhydrous $MgSO_4$ and concentrated under reduced pressure. The crude product was purified by flash column chromatography (EtOAc/Hexanes = 1:6) to afford **25** (1.79 g, 66%) as a colorless liquid. Analytical data for **25**: R_f = 0.35 (33% EtOAc in hexanes); ^{1}H NMR (400 MHz, $CDCl_3$) δ: 6.47 (dd, *J* = 6.1, 1.5 Hz, 1H, H1), 5.27 (ddd, *J* = 6.6, 2.4, 1.7 Hz, 1H, H3), 4.74 (dd, *J* = 6.1, 2.5 Hz, 1H, H2), 3.85 (ddd, *J* = 8.4, 6.6, 3.2 Hz, 1H, H4), 3.62-3.53 (m, 3H, H5 and H6), 3.45 (d, *J* = 3.3 Hz, 1H, OH at C4), 2.14 (s, 3H, CH_3CO); ^{13}C NMR (100 MHz, $CDCl_3$) δ: 172.9 (C=O), 146.1 (C1), 99.2 (C2), 76.1 (C5), 73.3 (C4), 71.1 (C3), 21.2 ($\mathit{C}H_3CO$), 5.8 (C6); HRMS (ESI): calcd for $C_8H_{10}IO_4^+$ [M + H]$^+$ 296.9618, found *m*/*z* 296.9611.

2.28 Synthesis of 3-*O*-Acetyl-6-iodo-4-*O*-*tert*-butyldimethylsilyl-D-glucal 25A

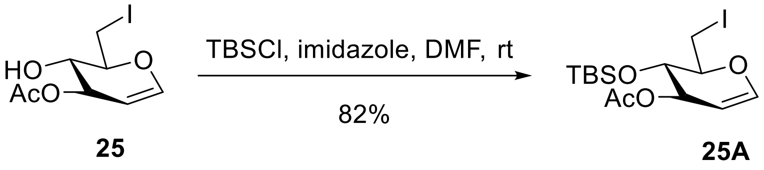

To a solution of **25** (1.94 g, 6.51 mmol) in DMF (13 mL), imidazole (2.22 g, 32.60 mmol) and TBSCl (2.45 g, 16.28 mmol) were added at room temperature and the reaction mixture was stirred for 10 h at the same temperature. The reaction mixture was diluted with EtOAc (100 mL) and washed with water (45 mL × 3) and brine (45 mL). The organic layer was separated, dried over anhydrous $MgSO_4$, and concentrated under reduced pressure. The obtained crude product was purified by flash column chromatography (EtOAc/Hexanes = 1:9) to afford **25A** (2.19 g, 82%) as a colorless liquid. Analytical data for **25A**: R_f = 0.60 (20% EtOAc in hexanes); ^{1}H NMR (400 MHz, $CDCl_3$) δ: 6.39(d, *J* = 5.9 Hz, 1H, H1), 5.15 (d, *J* = 5.4 Hz, 1H, H3), 4.77 (d, *J* = 3.5 Hz, 1H, H2), 3.96 (t, *J* = 7.4 Hz, 1H, H4), 3.56-3.51 (m, 2H, H6), 3.47-3.42 (m, 1H, H5), 2.08 (s, 3H, CH_3CO), 0.87 (s, 9H, CH_3 of TBS × 3), 0.20 (s, 3H, CH_3 of TBS), 0.11 (s, 3H, CH_3 of TBS); ^{13}C NMR (100 MHz, $CDCl_3$) δ: 170.6 (C=O), 145.1 (C1), 99.5 (C2), 76.4 (C5), 72.7 (C4), 70.5 (C3), 25.7, 21.3 ($\mathit{C}H_3CO$), 18.1 [$\mathit{C}(CH_3)_3$], 6.6 (C6), -4.4 (CH_3 of TBS), -4.5 (CH_3 of TBS); HRMS (ESI): calcd for $C_{14}H_{25}INaO_4Si^+$ $[M + Na]^+$ 435.0464, found *m*/*z* 435.0466.

2.29 Synthesis of 3-*O*-Acetyl-6-iodo-4-*O*-*tert*-butyldimethylsilyl-2,6-dideoxy-D-glucopyranosyl Acetate 21

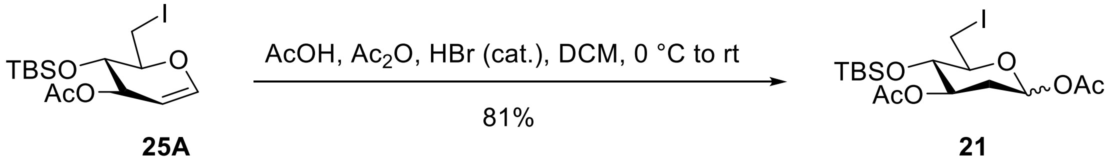

To a solution of **25A** (1.33 g, 3.23 mmol) in DCM (6.46 mL), AcOH (1.85 mL, 32.25 mmol) and Ac_2O (3.04 mL, 32.25 mmol) were added at 0 °C. After that, HBr (45% v/v in AcOH, 46.7 µL, 0.26 mmol) was added slowly into the reaction mixture at the same temperature. Reaction temperature was raised to room temperature and stirred for 10 h. NaOAc (0.4 g) was added to quench the reaction. The reaction mixture was diluted with DCM (50 mL) and washed with 5%

aqueous NaOH (30 mL × 2), water (30 mL) and brine (30 mL), dried over anhydrous $MgSO_4$, and concentrated under reduced pressure. The crude product was purified by flash column chromatography (EtOAc/Hexanes = 1:16) to afford **21** (1.11 g, 81%) as a 3:1 α/β anomeric mixture. Analytical data for *α*-anomer **21**: R_f = 0.30 (11% EtOAc in hexanes); ^{1}H NMR (400 MHz, $CDCl_3$) δ: 6.22-6.19 (m, 1H, H1), 5.06 (ddt, *J* = 11.1, 8.6, 5.5 Hz,1H, H3), 3.67-3.61 (m, 1H, H4), 3.55-3.47 (m, 1H, H6), 3.42-3.33 (m, 2H, H5 and H6), 2.34-2.29 (m, 1H, H2), 2.13 (s, 3H, CH_3CO), 2.08 (s, 3H, CH_3CO), 1.79-1.70 (m, 1H, H2), 0.88 (s, 9H, CH_3 of TBS), 0.20 (s, 3H, CH_3 of TBS), 0.13 (s, 3H, CH_3 of TBS); ^{13}C NMR (100 MHz, $CDCl_3$) δ: 169.9 (C=O), 169.1 (C=O), 90.8 (C1), 73.1 (C4), 72.3 (C5), 71.6 (C3), 33.6 (C2), 25.7 , 21.3 ($\mathit{C}H_3CO$), 21.0 ($\mathit{C}H_3CO$), 18.1 [$\mathit{C}(CH_3)_3$], 8.9 (C6), -4.2 (CH_3 of TBS), -4.3 (CH_3 of TBS); HRMS (ESI): calcd for $C_{16}H_{29}INaO_6Si^+$ $[M + Na]^+$ 495.0676, found *m*/*z* 495.0669.

2.30 Synthesis of Anhydrolandomycinonyl *β*-Olivoside 26

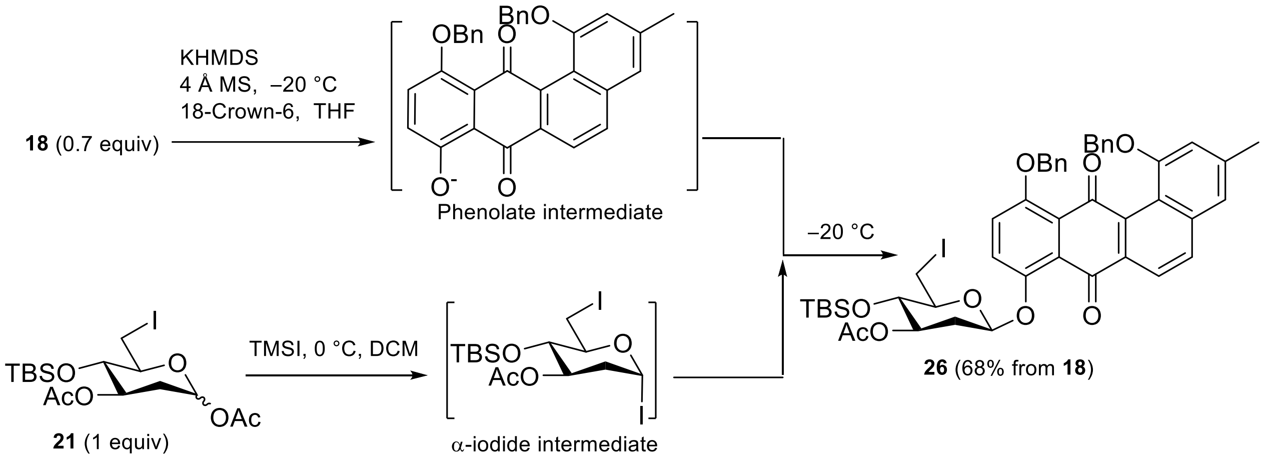

To a solution of donor **21** (310.20 mg, 0.66 mmol) in DCM (7 mL), TMSI (124.53 *μ*L, 0.88 mmol) was added at 0 °C. After stirring at 0 °C for 0.5 h, the solvent was evaporated under reduced pressure. The residue was coevaporated with toluene (× 3) at temperature ≤ 35 °C under N_2 atmosphere to afford the glycosyl iodide, which was further dried under *vacuo* for about 2 min.

To a separate two-necked flask was charged with landomycinone acceptor **18** (255.3 mg, 0.51 mmol), 18-crown-6-ether (134.80 mg, 0.51 mmol) and 4Å MS (776 mg). The mixture was dried under high reduced pressure for 1 h, then THF (5 mL) was added, and the mixture was stirred

at room temperature for 20 minutes. After that, the reaction mixture was cooled at -20 °C and 0.7 M of KHMDS in toluene (0.73 mL, 0.51 mmol) was added slowly. After stirring for 10 minutes at -20 °C, a solution of the glycosyl donor **21** in THF (5 mL) was added slowly. After stirring at -20 °C for 1.5 h, the reaction mixture was filtrated and the filtrate was diluted with EtOAc (50 mL) and washed with saturated $Na_2S_2O_3$ (30 mL), brine (30 mL), dried over anhydrous $MgSO_4$, and concentrated under reduced pressure. The crude product was purified by flash column chromatography (Elution: EtOAc/DCM/hexanes = 0.4/3/6.6) to afford **26** (311.02 mg, 68%) as a yellow amorphous solid. Analytical data for **26**: R_f = 0.26 (EtOAc : DCM : hexanes = 0.6 : 3 : 6.4); $[\alpha]_D^{25}$ = –9.88 (*c* 0.005, $CDCl_3$); ^{1}H NMR (400 MHz, $CDCl_3$) δ: 8.09 (d, *J* = 8.5 Hz, 1H, H6), 7.85 (d, *J* = 8.7 Hz, 1H, H5), 7.56 (d, *J* = 9.2 Hz, 1H), 7.43 (d, *J* = 7.1 Hz, 4H), 7.32-7.20 (m, 5H), 7.15-7.11 (m, 1H), 7.08-7.03 (m, 2H), 6.71 (d, *J* = 1.0 Hz, 1H, H2), 5.18-5.07 (m, 5H, H1′ and benzyl CH_2), 4.92 (ddd, *J* = 11.5, 8.6, 5.3 Hz, 1H, H3′), 3.64-3.57 (m, 2H, H4′ and H6′), 3.31-3.19 (m, 2H, H5′ and H6′), 2.91 (ddd, *J* = 12.5, 5.2, 2.1 Hz, 1H, H2′), 2.38 (s, 3H, CH_3 at C3), 2.12 (s, 3H, CH_3CO), 1.96 (ddd, *J* = 16.2, 10.9, 7.3 Hz, 1H, H2′), 0.87 (s, 9H, CH_3 of TBS), 0.17 (s, 3H, CH_3 of TBS), 0.12 (s, 3H, CH_3 of TBS); ^{13}C NMR (100 MHz, $CDCl_3$) δ: 186.5 (C=O), 182.1 (C=O), 170.0 (C=O), 155.6, 152.2, 149.5, 140.2, 137.8, 137.7, 137.4, 136.3, 132.6, 131.5 (C5), 128.4, 128.3, 127.7, 127.3, 127.1, 126.6, 126.4, 124.9, 122.1, 120.1 (C6), 119.9, 119.0, 112.4 (C2), 99.5 (C1′), 75.7 (C5′), 73.4 (C3′), 73.2 (C4′), 71.2 (benzyl CH_2), 70.8 (benzyl CH_2), 35.9, (C2′) 25.7 (CH_3 of TBS), 22.1 (CH_3 at C3), 21.4 ($\mathit{C}H_3CO$), 18.0 [$\mathit{C}(CH_3)_3$], 6.9 (C6′), –4.1 (CH_3 of TBS), –4.3 (CH_3 of TBS); HRMS (ESI): calcd for $C_{47}H_{50}IO_9Si^+$ $[M + H]^+$ 913.2269, found *m/z* 913.2273.

2.31 Synthesis of Anhydrolandomycinonyl *β*-Olivoside Acceptor 27

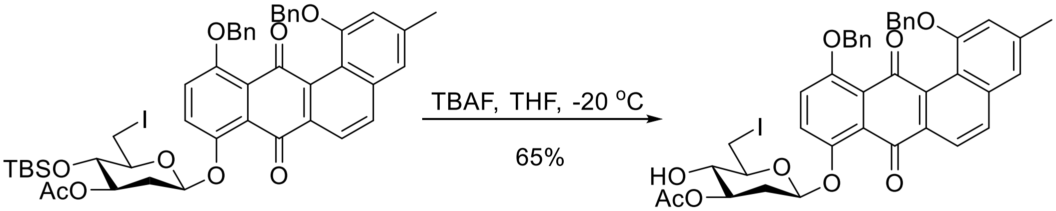

26 **27**

To a solution of **26** (121.41 mg, 0.13 mmol) in THF (2.6 mL), TBAF (1 M in THF, 0.16 mL, 0.16 mmol) was added at -20 °C. After stirring at –20 °C for 1 h, the reaction mixture was

diluted with EtOAc (50 mL) and washed with saturated NH_4Cl (20 mL), water (20 mL), brine (20 mL), dried over anhydrous $MgSO_4$ and concentrated under reduced pressure. The crude product was purified by flash column chromatography (EtOAc/DCM/hexanes = 0.4:1:1) to afford **27** (70.0 mg, 65%) as a yellow amorphous solid. Analytical data for **27**: R_f = 0.33 (EtOAc/DCM/ hexanes = 0.4:1:1, run for three times); $[\alpha]_D^{25}$ = -8.23 (*c* 0.005, $CDCl_3$); ^{1}H NMR (400 MHz, $CDCl_3$) δ: 8.07 (d, *J* = 8.6 Hz, 1H, H6), 7.85 (d, *J* = 8.6 Hz, 1H, H5), 7.58 (d, *J* = 7.4 Hz, 1H), 7.43 (d, *J* = 7.4 Hz, 4H), 7.33-7.20 (m, 5H), 7.13 (t, *J* = 7.3 Hz, 1H), 7.06 (d, *J* = 7.4 Hz, 2H), 6.71 (s, 1H, H2), 5.17-5.05 (m, 5H, H1′ and benzyl CH_2), 4.98 (ddd, *J* = 11.8, 9.0, 5.3 Hz, 1H, H3′), 3.65 (dd, *J* = 10.6, 2.2 Hz, 1H, H6′), 3.51(td, *J* = 9.0, 3.7 Hz, 1H, H4′), 3.34 (dd, *J* = 10.5, 7.8 Hz, 1H, H6′), 3.23 (td, *J* = 9.2, 2.0 Hz, 1H, H5′), 2.94-2.86 (m, 2H, OH at C4′ and H2′), 2.39 (s, 3H, CH_3 at C3), 2.16 (s, 3H, CH_3CO), 2.08 (dt, *J* = 12.1, 9.8 Hz, 1H, H2′) ; ^{13}C NMR (100 MHz, $CDCl_3$) δ: 186.4 (C=O), 182.3 (C=O), 172.1 (C=O), 155.6, 152.4, 149.3, 140.4, 137.82, 137.79, 137.4, 136.3, 132.5, 131.5 (C5), 128.4, 128.3, 127.7, 127.5, 127.4, 127.2, 127.1, 126.4, 125.2, 121.9 (C6), 120.1, 119.9, 119.0, 112.4 (C2), 100.1 (C1′), 75.5 (C5′), 73.8 (C3′), 73.7 (C4′), 71.2 (benzyl CH_2), 70.8 (benzyl CH_2), 35.8 (C2′), 22.1 (CH_3 at C3), 21.1 ($\underline{C}H_3CO$), 6.1 (C6′); HRMS (ESI): calcd for $C_{41}H_{35}INaO_9^+$ $[M + Na]^+$ 821.1223, found *m/z* 821.1233.

2.32 Synthesis of α-Rhodinosyl-(1,3)-olivosyl Acetate 28

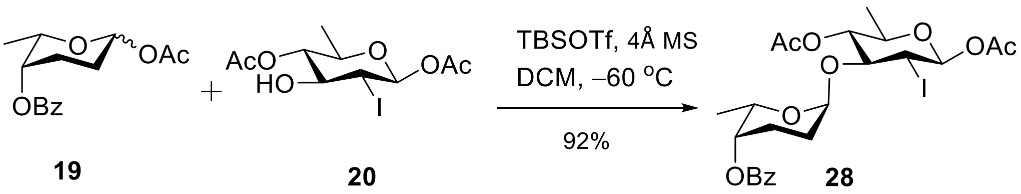

To a solution of acceptor **20** (356.90 mg, 1.00 mmol) and donor **19** (446.00 mg, 1.60 mmol) in anhydrous DCM (20 mL), 4Å MS (1.5 g) was added at room temperature and the mixture was stirred for 20 minutes at the same temperature. The reaction mixture was cooled to -60 °C and TBSOTf (46 μL, 0.20 mmol) was added dropwise. After stirring at -60 °C for 1.5 h, the reaction mixture was quenched with Et_3N and then filtrated. The filtrate was concentrated under reduced pressure and the crude product was purified by flash column chromatography (EtOAc/Hexanes = 1:7) to afford **28** (530.0 mg, 92%) as a colorless liquid. Analytical data for **28**: R_f = 0.25 (20%

EtOAc in hexanes); ^{1}H NMR (400 MHz, $CDCl_3$) δ: 8.09 (d, *J* = 7.3 Hz, 2H), 7.56 (t, *J* = 7.4 Hz, 1H), 7.44 (t, *J* = 7.7 Hz, 2H), 5.83 (d, *J* = 9.2 Hz, 1H, H1), 5.13 (s, 1H, H1′), 5.02 (s, 1H, H4′), 4.81 (t, *J* = 9.2 Hz, 1H, H4), 4.15-4.06 (m, 1H, H5′), 3.99-3.84 (m, 2H, H2 and H3), 3.60 (dq, *J* = 9.7, 6.2 Hz, 1H, H5), 2.15 (s, 3H, CH_3CO), 2.11 (s, 3H, CH_3CO), 2.06-1.87 (m, 4H, H2′ and H3′), 1.21 (d, *J* = 6.2 Hz, 3H, H6), 1.12 (d, *J* = 6.5 Hz, 3H, H6′); ^{13}C NMR (100 MHz, $CDCl_3$) δ: 170.0 (C=O), 168.9 (C=O), 166.1, 133.0, 130.4, 129.6, 128.4, 100.2 (C1′), 94.0 (C1), 84.1 (C3), 74.8 (C4), 71.3 (C5), 70.0 (C4′), 66.8 (C5′), 30.8 (C2), 25.1 (C2′), 22.5 (C3′), 21.4 (*C*H_3CO), 20.8 (*C*H_3CO), 17.3 (C6), 17.0 (C6′); HRMS (ESI): calcd for $C_{23}H_{29}INaO_9^+$ $[M + Na]^+$ 599.0754, found *m*/*z* 599.0749.

2.33 Synthesis of α-Rhodinosyl-(1,3)-olivosyl *N*-Phenyltrifluoroacetimidate 29

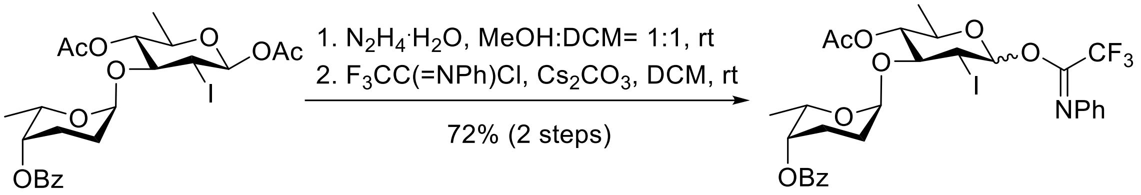

28 **29**

To a solution of **28** (530 mg, 0.92 mmol) in MeOH (5 mL)/DCM (5 mL), $N_2H_4 \cdot H_2O$ (0.18 mL) was added at room temperature. After stirring at room temperature for 2 h, the reaction mixture was diluted with DCM (100 mL) and washed with water (20 mL), dried over anhydrous $MgSO_4$, and concentrated under reduced pressure. The crude product was purified by flash column chromatography (EtOAc/Hexanes = 1:4) to afford a hemiacetal intermediate which was dissolved in DCM (6 mL), then Cs_2CO_3 (779.36 mg, 2.39 mmol) and *N*-phenyl-2,2,2-trifluoroacetimidoyl chloride (301.15 µL, 1.89 mmol) were added to it at 0 °C. After stirring at room temperature for 2 h, the reaction mixture was filtered over celite. The filtrate was concentrated under reduced pressure and the crude product was purified by flash column chromatography (pH = 7.0, EtOAc/Hexanes = 1:13) to afford **29** (466.82 mg, 72% over two steps) as a mixture of α/β-isomer. **As c**ompound **29** was an unstable donor, it was used for the glycosylation immediately without characterization.

2.34 Assembly of Protected Landomycin Q 30

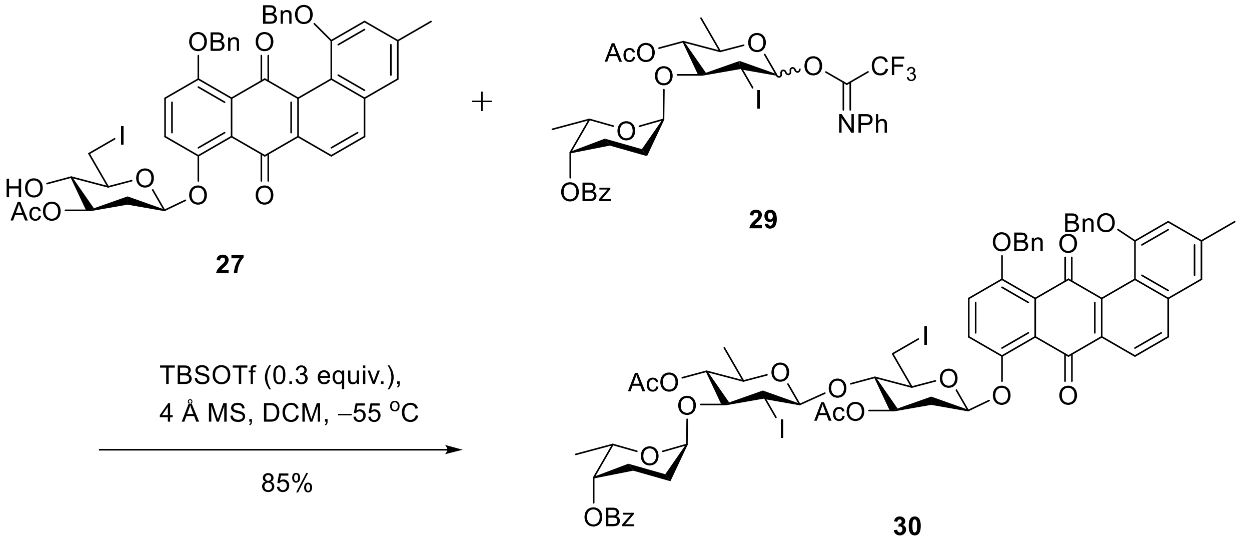

A flame dried two-necked round bottom flask was charged with acceptor **27** (78.4 mg, 0.09 mmol), trifluoroacetimidate donor **29** (109.40 mg, 0.16 mmol) and 4Å MS (550 mg). The mixture was dried under reduced pressure for 1 h. DCM (5.5 mL) was added to it and stirred for 20 minutes at room temperature. Then, the reaction mixture was cooled at -55 °C and TBSOTf (7.6 μL, 0.03 mmol) was added slowly. After stirring for 7 h at -55 °C, the reaction was quenched with Et_3N and filtrated to remove 4Å MS. The filtrate was concentrated under reduced pressure and the crude product was purified by flash column chromatography (EtOAc/DCM/hexanes = 0.2:0.8:1) to afford **30** (109.20 mg, 85%) as a yellow amorphous solid. Analytical data for **30** : R_f = 0.28 (EtOAc/DCM/hexanes = 0.2:0.8:1); $[\alpha]_D^{25}$ = –14.71 (*c* 0.005, $CDCl_3$); ^{1}H NMR (600 MHz, $CDCl_3$) δ: 8.12-8.06 (m, 3H), 7.86 (d, *J* = 8.6 Hz, 1H), 7.59-7.53 (m, 2H), 7.48-7.41 (m, 6H), 7.30 (t, *J* = 7.4 Hz, 1H), 7.25 (d, *J* = 7.6 Hz, 1H), 7.24-7.20 (m, 2H), 7.13 (t, *J* = 7.3 Hz, 1H), 7.07 (t, *J* = 7.4 Hz, 2H), 6.71 (s, 1H, H2), 5.25-5.20 (m, 1H, H3′), 5.17-5.02 (m, 7H, H1′, H1‴, H4‴, and benzyl CH_2), 4.81-4.75 (m, 2H, H1″ and H4″), 4.15-4.10 (m, 1H, H5‴), 3.88-3.80 (m, 3H, H6′ and H2″), 3.74 (t, *J* = 8.7 Hz, 1H, H4′), 3.59 (dd, *J* = 10.5, 6.8 Hz, 1H), 3.48-3.42 (m, 1H, H5″), 3.39 (dd, *J* = 10.7, 4.3 Hz, 1H), 2.86 (dd, *J* = 12.1, 4.5 Hz, 1H, H2′), 2.39 (s, 3H, CH_3 at C3), 2.12 (s, 6H, CH_3CO), 2.09-1.89 (m, 5H, H2′, H2‴, and H3‴), 1.24 (d, *J* = 6.0 Hz, 3H, H6″), 1.12 (d, *J* = 6.4 Hz, 3H, H6‴); ^{13}C NMR (150 MHz, $CDCl_3$) δ: 186.4 (C=O), 182.1 (C=O), 170.0 (C=O), 169.7

(C=O), 166.1 (C=O), 155.7, 152.3, 149.4, 140.3, 137.8, 137.7, 137.4, 136.3, 133.0, 132.6, 131.5, 130.4, 129.6, 128.4, 128.35, 128.32, 127.7, 127.36, 127.34, 127.1, 126.9, 126.4, 125.1, 122.0, 120.1, 119.9, 119.0, 112.4 (C2), 101.6 (C1″), 100.3 (C1‴), 99.6 (C1′), 84.5, 78.9 (C4′), 75.2 (C4″), 74.2, 71.2 (benzyl CH_2), 70.8 (benzyl CH_2), 70.4 (C5″), 70.1 (C4‴), 69.9 (C3′), 66.8 (C5‴), 35.7 (C2′), 33.8 (C2″), 25.2, 22.5, 22.1 (CH_3 at C3), 21.39 (*C*H_3CO), 21.34 (*C*H_3CO), 17.5 (C6″), 17.0 (C6‴), 7.6 (C6′); HRMS (ESI): calcd for $C_{62}H_{61}I_2O_{16}^+$ $[M + H]^+$ 1315.2049, found *m/z* 1315.2075.

Activation Procedure for Raney Ni

Raney Ni (1 g) was added to 2 M aqueous NaOH (30 mL) solution and the suspension was stirred at 80 °C for 1 h. The solvent was decanted and the residue was again dissolved in 2 M aqueous NaOH (30 mL) and stirred at 80 °C for another 1 h. Then the solvent was decanted and the residue was washed with double-distilled H_2O (until pH ~ 7.0) and finally washed with MeOH (10 mL × 2) to remove the water.[13]

2.35 Synthesis of Landomycin Q (3)

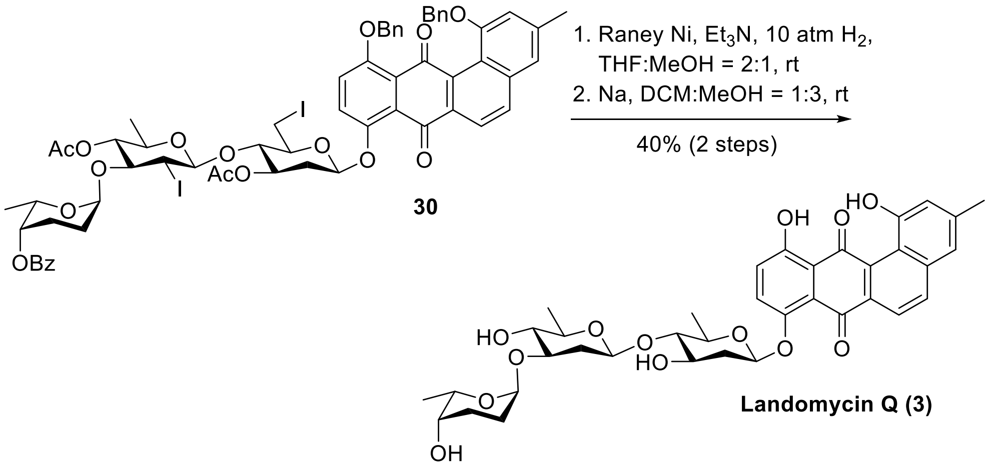

To a solution of **30** (75.40 mg, 0.057 mmol) in the MeOH (2 mL)/THF (4 mL), NaOH-activated Raney Ni (1 g) and Et_3N (48 μL, 0.34 mmol) were added. After stirring under H_2 (10 atm) for 14 h, the reaction mixture was filtered over celite. The filtrate was concentrated under reduced pressure to obtain the debenzylated compound which was used for the next step without further purification.

To a DCM (2 mL)/MeOH (6 mL) solution of the debenzylated and deiodinated product above, catalytic amount of sodium was added. After stirring at room temperature for 24 h, the reaction mixture was neutralized with 5% aqueous HCl (until pH = 7.0), then diluted with DCM (100 mL) and washed with water (20 mL), brine (20 mL), dried over anhydrous $MgSO_4$ and concentrated under reduced pressure. The crude product was purified by flash column chromatography (MeOH/EtOAc/hexanes = 0.04:3:1 gradient to 0.08:3:1) to afford **Landomycin Q (3)** (16.1 mg, 40%) as a red solid. The $CDCl_3$ solution of **3** has a deep red color that rendered the determination of the optical rotation difficult. Analytical data for **Landomycin Q (3)**: R_f = 0.17 (75% EtOAc in hexanes); ^1H NMR (400 MHz, $CDCl_3$) δ: 12.26 (s, 1H, OH at C11), 10.64 (s, 1H, OH at C1), 8.26 (d, *J* = 8.6 Hz, 1H, H6), 8.14 (d, *J* = 8.7 Hz, 1H, H5), 7.55 (d, *J* = 9.3 Hz, 1H, H9), 7.28 (d, *J* = 9.2 Hz, 1H, H10), 7.27 (s, 1H, H4), 7.17 (s, 1H, H2), 5.10 (dd, *J* = 9.6, 2.0 Hz, 1H, H1′), 4.96 (s, 1H, H1‴), 4.72 (s, 1H, OH at C3′), 4.54 (dd, *J* = 9.2, 1.6 Hz, 1H, H1″), 4.39 (s, 1H, OH at C4″), 4.15 (q, *J* = 6.2 Hz, 1H, H5‴), 3.76 (ddd, *J* = 12.4, 8.4, 5.2, 1H, H3′), 3.65 (br, 1H, H4‴), 3.54 (ddd, *J* = 12.0, 8.4, 4.8, 1H, H3″), 3.45 – 3.37 (m, 2H, H5′ and H5″), 3.13 (dd, *J* = 8.8, 8.4 Hz, 2H, H4′ and H4″), 2.82 (ddd, *J* = 12.1, 4.4, 1.6 Hz, 1H, H2′), 2.50 (s, 3H, CH_3 at C3), 2.25 (ddd, *J* = 12.8, 4.8, 1.2 Hz, 1H, H2″), 2.07 – 1.92 (m, 3H, H2′, H2‴, and H3‴), 1.80-1.55 (m, 3H, H2″, H2‴ and H3‴), 1.41 (d, *J* = 6.1 Hz, 3H, H6″), 1.30 (d, *J* = 6.1 Hz, 3H, H6′), 1.23 (d, *J* = 6.8 Hz, 3H, H6‴); ^{13}C NMR (100 MHz, $CDCl_3$) δ: 193.1 (C12), 181.5 (C7), 159.2 (C8), 154.5 (C1), 150.5 (C3), 141.3 (C4a), 138.5 (C5), 138.4 (C11a), 136.9 (C6a), 132.3 (C10), 130.2 (C12a), 126.3 (C9), 122.6 (C11), 121.7 (C6), 120.6 (C7a), 120.5 (C4), 119.1 (C2), 116.7 (C12b), 100.8 (C1″), 99.9 (C1′), 97.5 (C1‴), 88.0 (C4′), 80.4 (C3″), 75.2 (C4″), 72.3 (C5′), 70.8 (C5″), 69.3 (C3′), 67.7 (C5‴), 67.1 (C4‴), 37.6 (C2′), 37.0 (C2″), 25.4 (C3‴), 24.1 (C2‴), 21.2 (CH_3 at C3), 17.8 (C6′ and C6″), 17.0 (C6‴); HRMS (ESI): calcd for $C_{37}H_{42}NaO_{13}^+$ $[M + Na]^+$ 717.2523, found *m/z* 717.2514.

2.36 Synthesis of 5,6-Dihydrotetraphene-7,12-dione *β*-Olivoside 31

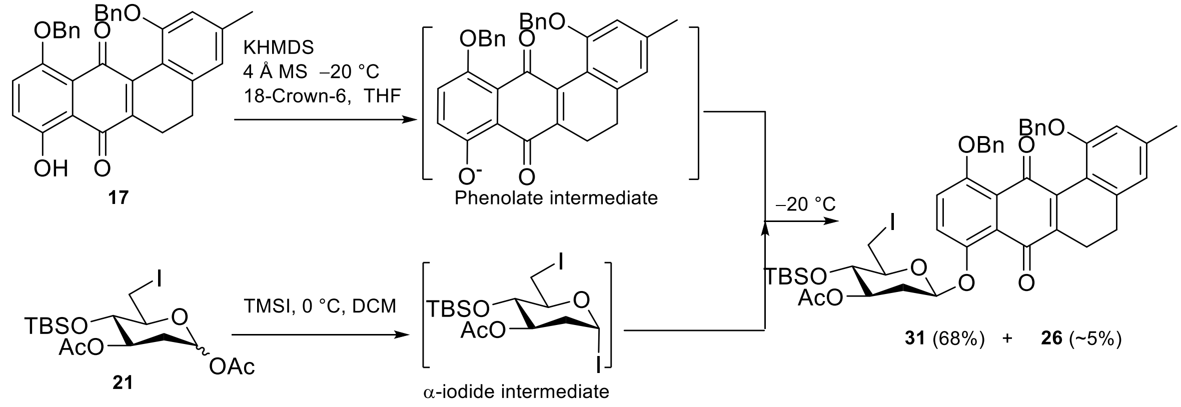

Same procedure as for preparation **26** except that 5,6-dihydrotetraphene-7,12-dione acceptor **17** (221.41 mg, 0.44 mmol) was employed. The residue was concentrated for flash column chromatography purification (EtOAc/DCM/hexanes = 0.4:3:6.6) to afford **31** (273.55 mg, 68%) as a yellow amorphous solid along with small amont of compound **26** (~5%). Analytical data for **32**: R_f = 0.25 (EtOAc/DCM/hexanes = 0.6:3:6.4); $[\alpha]_D^{25}$ = -9.50 (*c* 0.005, $CDCl_3$); ^{1}H NMR (600 MHz, $CDCl_3$) δ: 7.56 (d, *J* = 9.2 Hz, 1H), 7.46-7.42 (m, 2H), 7.24-7.15 (m, 9H), 6.70 (s, 1H), 6.61 (s, 1H, H2), 5.12-5.04 (m, 3H, H1′ and benzyl CH_2), 5.01 (s, 2H, benzyl CH_2), 4.90 (ddd, *J* = 11.5, 8.6, 5.3 Hz, 1H, H3′), 3.63-3.55 (m, 2H, H4′ and H6′), 3.29-3.24 (m, 1H, H6′), 3.18 (td, *J* = 8.4, 2.4 Hz, 1H, H5′), 2.88 (ddd, *J* = 12.5, 5.1, 2.0 Hz, 1H, H2′), 2.83 (br, 1H, H6), 2.74 – 2.69 (m, 2H, H5), 2.53 (br, 1H, H6), 2.28 (s, 3H, CH_3 at C3), 2.10 (s, 3H, CH_3CO), 1.92 (ddd, *J* = 11.9, 10.7, 7.3 Hz, 1H, H2′), 0.87 (s, 9H, CH_3 of TBS), 0.16 (s, 3H, CH_3 of TBS), 0.11 (s, 3H, CH_3 of TBS); ^{13}C NMR (150 MHz, $CDCl_3$) δ: 183.6 (C=O), 182.5 (C=O), 169.9 (C=O), 156.4, 153.1, 149.4, 144.2, 141.9, 141.0, 140.6, 137.4, 136.4, 128.3, 128.1, 127.7, 127.6, 127.2, 126.9, 126.5, 124.23, 124.21, 121.3, 120.2, 117.8, 113.0 (C2), 99.6 (C1′), 75.6 (C5′), 73.3 (C3′), 73.2 (C4′), 71.2 (benzyl CH_2), 71.0 (benzyl CH_2), 35.9 (C2′), 28.4 (C5), 25.7 (CH_3 of TBS), 21.8 (CH_3 at C3), 21.3 ($\mathit{C}H_3CO$), 19.5 (C6), 18.0 [$\mathit{C}(CH_3)_3$], 6.9 (C6′), -4.2 (CH_3 of TBS), -4.3 (CH_3 of TBS); HRMS (ESI): calcd for $C_{47}H_{51}INaO_9Si^+$ $[M + Na]^+$ 937.2245, found *m*/*z* 937.2229.

2.37 Synthesis of 5,6-Dihydrotetraphene-7,12-dione *β*-Olivoside Acceptor 32

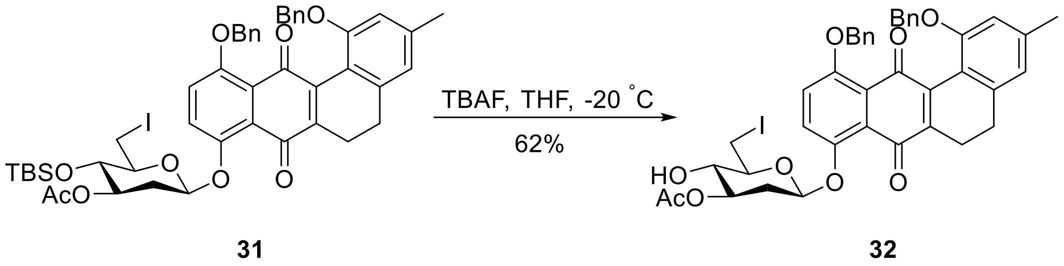

Same procedure as for preparation **27** except that *β*-olivoside acceptor **31** (273.52 mg, 0.30 mmol) was used as the starting substrate. The residue was concentrated for flash column chromatographic purification (Elution: EtOAc/DCM/hexanes = 0.4:1:1) to afford **32** (147.80 mg, 62%) as a yellow amorphous solid. Analytical data for **32**: R_f = 0.30 (EtOAc/DCM/hexanes = 0.4:1:1 run for three times); $[\alpha]_D^{25}$ = -7.98 (*c* 0.005, $CDCl_3$); ^1H NMR (400 MHz, $CDCl_3$) δ: 7.57 (d, *J* = 9.2 Hz, 1H), 7.46-7.41 (m, 2H), 7.25-7.15 (m, 9H), 6.70 (s, 1H), 6.62 (s, 1H, H2), 5.13-5.00 (m, 5H, H1′ and benzyl CH_2), 4.94 (ddd, *J* = 11.8, 9.0, 5.3 Hz, 1H, H3′), 3.62 (dd, *J* = 10.6, 2.3 Hz, 1H, H6′), 3.48 (td, *J* = 9.0, 3.6 Hz, 1H, H4′), 3.32 (dd, *J* = 10.6, 7.8 Hz, 1H, H6′), 3.20 (td, *J* = 8.0, 2.4 Hz, 1H, H5′), 2.98 (s, 1H, OH at C4′), 2.89 (br, 1H, H6), 2.84 (ddd, *J* = 12.5, 5.2, 1.9 Hz, 1H, H2′), 2.72 (dd, *J* = 10.2, 6.0 Hz, 2H, H5), 2.56-2.41 (br, 1H, H6), 2.28 (s, 3H, CH_3 at C3), 2.13 (s, 3H, CH_3CO), 2.08-1.98 (m, 1H, H2′); ^{13}C NMR (100 MHz, $CDCl_3$) δ: 183.6 (C=O), 182.8 (C=O), 171.9 (C=O), 156.4, 153.4, 149.3, 144.4, 142.0, 141.0, 140.5, 137.4, 136.3, 128.6, 128.3, 128.2, 127.6, 127.2, 126.9, 126.6, 124.4, 124.1, 121.4, 120.1, 117.8, 113.1 (C2), 100.1 (C1′), 75.4 (C5′), 73.7 (C3′), 73.6 (C4′), 71.2 (benzyl CH_2), 71.0 (benzyl CH_2), 35.8 (C2′), 28.3 (C5), 21.9 (CH_3 at C3), 21.0 ($\underline{C}H_3CO$), 19.5 (C6), 6.1 (C6′); HRMS (ESI): calcd for $C_{41}H_{37}INaO_9^+$ $[M + Na]^+$ 823.1380, found *m*/*z* 823.1386.

2.38 Synthesis of Olivosyl Trichloroacetimidate Donor 33

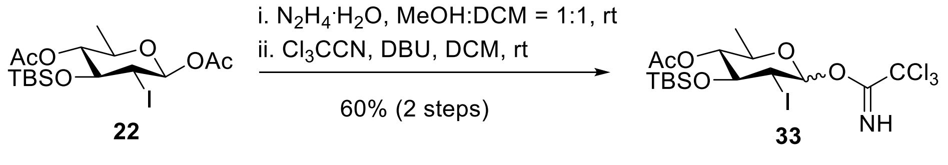

To a solution of non-reducing end olivosyl acetate **22** (348 mg, 0.74 mmol) in MeOH (4 mL)/DCM (4 mL), $N_2H_4 \cdot H_2O$ (74 μL) was added at room temperature. After stirring at room temperature for 3 h, the reaction mixture was diluted with DCM (100 mL) and washed with water (30 mL), dried over anhydrous $MgSO_4$, and concentrated under reduced pressure. The crude product was purified by flash column chromatography (EtOAc/Hexanes = 1:4) to afford hemiacetal as a colorless liquid.

To a solution of the above obtained hemiacetal in DCM (9 mL), DBU (66.40 μL, 0.44 mmol) and trichloroacetonitrile (0.74 mL, 7.4 mmol) were added at 0 °C. After stirring at room temperature for 8 h, the solvent was evaporated and the crude product was purified by flash column chromatography (pH = 7.0, Et_3N/EtOAc/hexanes = 0.1:1:19) to afford **33** (252.13 mg, 60% over two steps) as a mixture of α/β-isomer. **33** was unstable, thus it was used for the glycosylation immediately after purification.

2.39 Synthesis of 5,6-Dihydrotetraphene-7,12-dionyl Disaccharide 34

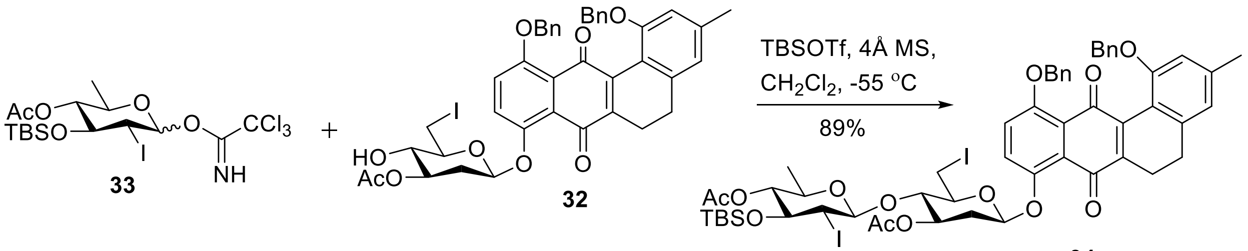

A flame dried two-necked round bottom flask was charged with acceptor **32** (69.90 mg, 0.088 mmol), olivosyl trichloroacetimidate donor **33** (70.42 mg, 0.12 mmol) and 4Å MS (437.5 mg). The mixture was dried under reduced pressure for 1 h. DCM (4.4 mL) was added to it and stirred for 20 minutes at room temperature. The mixture was cooled to –55 °C, then TBSOTf (6.04 μL, 0.026 mmol) was added slowly. After stirring for 2 h at –55 °C, the reaction mixture was quenched with Et_3N and the solution was filtered to remove 4Å MS. The filtrate was concentrated under reduced pressure for flash column chromatographic purification (EtOAc/DCM/ hexanes = 0.2:0.8:2) to afford **34** (94.70 mg, 89%) as a yellow amorphous solid. Analytical data for **34**: R_f = 0.43 (EtOAc/DCM/Hexane = 0.2:0.8:1); $[\alpha]_D^{25}$ = + 8.42 (*c* 0.005, $CDCl_3$); ^{1}H NMR (400 MHz, $CDCl_3$) δ: 8.08 (d, *J* = 8.5 Hz, 1H, H6), 7.85 (d, *J* = 8.6 Hz, 1H, H5), 7.54 (d, *J* = 9.2 Hz, 1H), 7.43

(d, *J* = 7.2 Hz, 4H), 7.30 (t, *J* = 7.2 Hz, 2H), 7.25-7.19 (m, 3H), 7.13 (d, *J* = 7.1 Hz, 1H), 7.07 (t, *J* = 7.3 Hz, 2H), 6.71 (s, 1H, H2), 5.25-5.17 (m, 1H, H3′), 5.16-5.05 (m, 5H, H1′ and benzyl CH_2), 4.76 (d, *J* = 9.0 Hz, 1H, H1″), 4.67 (t, *J* = 9.0 Hz, 1H, H4″), 3.97-3.86 (m, 2H, H3″ and H6′), 3.73 (dd, *J* = 18.8, 9.3 Hz, 2H, H2″ and H4′), 3.57 (dd, *J* = 10.4, 7.0 Hz, 1H, H6′), 3.44-3.35 (m, 2H, H5′ and H5″), 2.85 (dd, *J* = 12.3, 4.4 Hz, 1H, H2′), 2.39 (s, 3H, CH_3 at C3), 2.11 (s, 3H, CH_3CO), 2.10 (s, 3H, CH_3CO), 2.07-2.01 (m, 1H, H2′), 1.20 (d, *J* = 6.1 Hz, 3H, H6″), 0.89 (s, 9H, CH_3 of TBS), 0.27 (s, 3H, CH_3 of TBS), 0.04 (s, 3H, CH_3 of TBS); ^{13}C NMR (100 MHz, $CDCl_3$) δ: 186.4 (C=O), 182.1 (C=O), 169.8 (C=O), 169.7 (C=O), 155.7, 152.3, 149.4, 140.3, 137.8, 137.7, 137.4, 136.3, 132.6, 131.5 (C5), 128.4, 128.3, 127.7, 127.3, 127.1, 126.8, 126.4, 125.1, 122.0 (C6), 120.1, 120.0, 119.0, 112.4 (C2), 101.7 (C1″), 99.6 (C1′), 78.8 (C4′), 76.7 (C3″), 76.6 (C4″), 74.4 (C5′), 71.2 (benzyl CH_2), 70.8 (benzyl CH_2), 70.4 (C5″), 70.0 (C3′), 36.7 (C2″), 35.7 (C2′), 26.1 (CH_3 of TBS), 22.1 (CH_3 at C3), 21.6 (*C*H_3CO), 21.4 (*C*H_3CO), 18.2 [*C*$(CH_3)_3$], 17.7 (C6″), 7.7 (C6′), -3.0 (CH_3 of TBS), -3.5 (CH_3 of TBS) ; HRMS (ESI): calcd for $C_{55}H_{61}I_2O_{13}Si^+$ $[M + H]^+$ 1211.1971, found *m*/*z* 1211.1990.

2.40 Synthesis of Landomycin R (4)

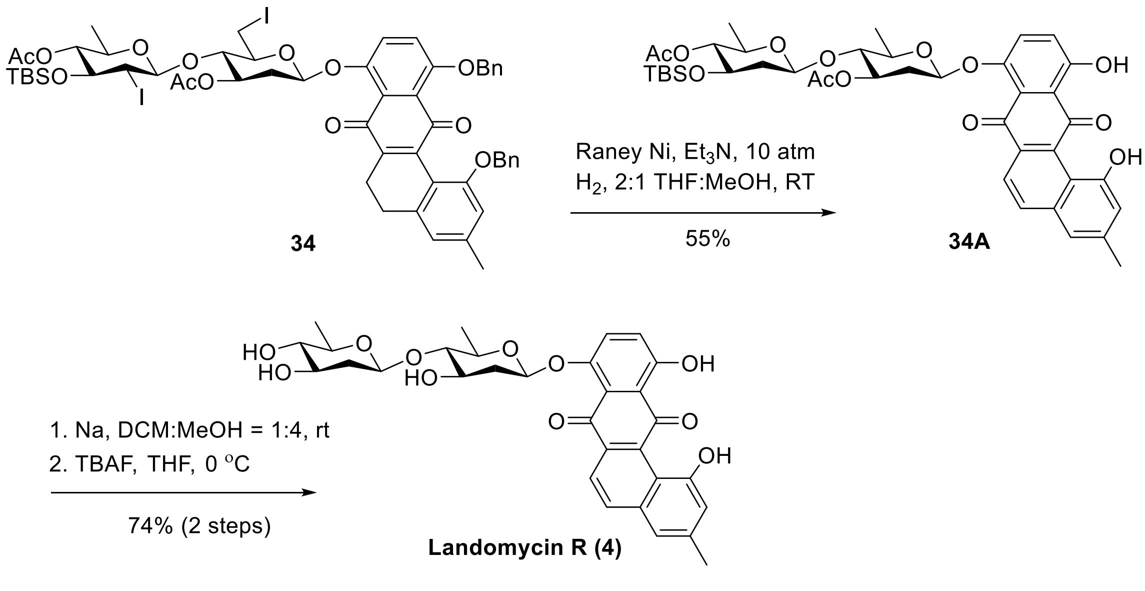

To a solution of **34** (70.50 mg, 0.058 mmol) in the MeOH (2 mL)/THF (4 mL), activated Raney Ni (1.0 g) and Et_3N (48.0 μL, 0.349 mmol) were added. After stirring under H_2 (10 atm) for

17 h, the reaction mixture was filtered through celite. The filtrate was concentrated under reduced pressure for flash column chromatographic purification (EtOAc/DCM/hexanes = 0.2:0.8:1.5) to afford **34A** (25.3 mg, 55%) as a dark red solid. Analytical data for **34A**: R_f = 0.25 (EtOAc/DCM/hexanes = 0.2:0.8:1.5); ^{1}H NMR (400 MHz, $CDCl_3$) δ: 12.26 (s, 1H, OH at C11), 10.61 (s, 1H, OH at C1), 8.25 (d, *J* = 8.6 Hz, 1H, H6), 8.15 (d, *J* = 8.7 Hz, 1H, H5), 7.55 (d, *J* = 9.3 Hz, 1H), 7.31-7.28 (m, 2H), 7.18 (s, 1H), 5.17-5.07 (m, 2H), 4.59 (t, *J* = 9.2 Hz, 1H), 4.52 (d, *J* = 8.2 Hz, 1H), 3.71 (ddd, *J* = 11.6, 8.9, 5.3 Hz, 1H), 3.51-3.40 (m, 2H), 3.33 (dq, *J* = 12.4, 6.3 Hz, 1H), 2.79 (ddd, *J* = 12.5, 5.3, 1.9 Hz, 1H), 2.51 (s, 3H, CH_3), 2.12-2.03 (m, 8H), 1.73-1.64 (m, 1H), 1.34 (d, *J* = 5.7 Hz, 3H), 1.21 (d, *J* = 6.2 Hz, 3H), 0.84 (s, 9H), 0.05 (s, 3H), 0.03 (s, 3H).

To a DCM (3 mL)/MeOH (12 mL) solution of **34A** (43.20 mg, 0.055 mmol), catalytic amount sodium was added. After stirring at room temperature for 24 h, the reaction mixture was neutralized with 5% aqueous HCl (until pH = 7.0). DCM (100 mL) was added to the reaction mixture and the organic layer was washed with water (30 mL), brine (30 mL), dried over anhydrous $MgSO_4$ and concentrated under reduced pressure. The crude product was dissolved in THF (5.5 mL) and TBAF (0.5 M in THF, 0.17 mL, 0.083 mmol) was added at 0 °C. After stirring at 0 °C for 0.5 h, the reaction mixture was diluted with DCM (50 mL). The organic layer was washed with saturated NH_4Cl (20 mL), water (20 mL), brine (20 mL), dried over anhydrous $MgSO_4$ and concentrated under reduced pressure. The crude product was purified by flash column chromatography (IPA/DCM/hexanes = 0.15:5:1) to afford the title compound **Landomycin R** (**4**) (23.60 mg, 74% over two steps) as a red solid. Similar to LA Q (**3**), the optical rotation [α] of **4** was not determined. Analytical data for **Landomycin R** (**4**): R_f = 0.33 (IPA/DCM/hexanes = 0.2:5:1); ^{1}H NMR (600 MHz, 10% CD_3OD in $CDCl_3$) δ: 8.23 (d, *J* = 8.5 Hz, 1H, H6), 8.16 (d, *J* = 8.5 Hz, 1H, H5), 7.57 (d, *J* = 9.3 Hz, 1H, H9), 7.33-7.29 (m, 2H, H4 and H10), 7.14 (s, 1H, H2), 5.14 (d, *J* = 8.7 Hz, 1H, H1′), 4.59 (d, *J* = 9.7 Hz, 1H, H1″), 3.79-3.74 (m, 1H, H3′), 3.60-3.55 (m, 1H, H3″), 3.45 (dt, *J* = 14.0, 5.6 Hz, 1H, H5′), 3.40 (dt, *J* = 12.2, 6.3 Hz, 1H, H5″), 3.14 (t, *J* = 8.7 Hz, 1H, H4′), 3.04 (t, *J* = 9.0 Hz, 1H, H4″), 2.79 (dd, *J* = 12.0, 4.6 Hz, 1H, H2′), 2.50 (s, 3H, CH_3 at C3), 2.25 (dd, *J* = 12.0, 3.9 Hz, 1H, H2″), 1.96 (dd, *J* = 22.1, 11.8 Hz, 1H, H2′), 1.64 (dd, *J* = 22.5, 12.1 Hz, 1H, H2″), 1.38 (d, *J* = 6.1 Hz, 3H, H6″), 1.33 (d, *J* = 6.0 Hz, 3H, H6′); ^{13}C NMR (150 MHz, 10% CD_3OD in $CDCl_3$) δ: 192.6 (C12), 181.4 (C7), 158.5 (C8), 153.9 (C1), 150.2

(C3), 141.1 (C4a), 138.3 (C5), 138.0 (C11a), 136.5 (C6a), 131.3 (C10), 130.0 (C12a), 126.1 (C9), 122.2 (C11), 121.5 (C6), 120.0 (C7a), 119.9 (C4), 118.6 (C2), 116.4 (C12b), 100.6 (C1″), 99.3 (C1′), 87.2 (C4′), 76.3 (C4″), 72.1 (C5″), 70.54 (C5′), 70.46 (C3″), 69.0 (C3′), 38.4 (C2″), 37.2 (C2′), 29.3 (CH_3 at C3), 17.3 (C6′), 17.0 (C6″); HRMS (ESI): calcd for $C_{31}H_{31}O_{11}^+$ $[M + H]^+$ 579.1866, found *m*/*z* 579.1984.

MTS Assay for Cell Viability

Cultured Detroit 551, NCI-H460, and SF-268 cells were seeded on 96-well plates at a density of 2500, 2500, and 7500 cells/well, respectively. Test compounds were added 24 hours after cell seeding. MTS assay was performed 3 days after drug treatment to determine the viability of cells. The MTS assay (CellTiter96 AQ_{ueous} Non-Radioactive Cell Proliferation Assay, Promega) is a homogeneous colorimetric method based on the ability of viable cells to convert a soluble tetrazolium salt (MTS) to a colored formazan product.[14] The quantity of formazan product, as measured by 490 nm absorbance, is directly proportional to the number of viable cells in culture. The detection reagent is composed of solutions of MTS (3-(4,5-dimethylthiazol-2-yl)-5-(-3-carboxymethoxyphenyl)-2-(4-sulfophenyl)-2H-tetrazolium, inner salt) and PMS (phenazine methosulfate). PMS is an electron transfer coupling reagent that facilitates cellular reduction of MTS into a colored soluble formazan. At the end of the 72 h incubation period with the test chemicals, the cells were then incubated with 20 μL of MTS/PMS mixtures (MTS/PMS ratio: 20:1) in each well of 96-well plates for 90 minutes at 37 °C in the humidified incubator with 5% CO_2 to allow viable cells to convert tetrazolium salt (MTS) into formazan. The absorbance/optical density (OD) was recorded at 490 nm using a Perkin Elmer Victor2 plate reader (Table S2).

Table S2. Raw Data of Inhibitory Effects on Cell Proliferation by Anhydrolandomycinone (1), B-ring unsaturated core analogue (2), LA Q (3), and LA R (4).

NCI-H460	2,500 cells/well					Plate 01 RAW DATA							
1	2	3	4	5	6	7	8	9	10	11	12		
nM	1nM	10nM	100nM	1000nM	10000nM	1nM	10nM	100nM	1000nM	10000nM	0	Compound	IC50
1.443	1.65	1.66	1.62	1.55	0.19	1.68	1.62	1.63	1.67	0.20	1.435	Core (1)	7.00
0.070	1.63	1.62	1.62	1.55	0.70	1.65	1.59	1.62	1.61	0.81	0.070	Analogue (2)	>10.0 uM
Maximune	1.439926113	Minimune	0.07059872										

NCI-H460	2,500 cells/well					Plate 02 RAW DATA							
1	2	3	4	5	6	7	8	9	10	11	12		
nM	1250nM	2500nM	5000nM	10000nM	20000nM	1250nM	2500nM	5000nM	10000nM	20000nM	0	Compound	IC50
0.071	1.6	1.6	1.6	1.6	1.5	1.7	1.6	1.7	1.6	1.6	0.072	LA Q (3)	>10.0 uM
1.435	1.69	1.68	1.69	1.65	1.57	1.71	1.70	1.72	1.67	1.58	1.474	LA R (4)	>10.0 uM
1.452	1.68	1.71	1.68	0.97	0.72	1.71	1.75	1.62	1.03	0.76	1.553	Vx680	3.40
1.504	1.69	1.69	1.71	1.66	1.74	1.70	1.74	1.70	1.70	1.72	1.518	0	#DIV/0!
1.548	1.68	1.67	1.77	1.61	1.69	1.69	1.73	1.69	1.72	1.72	1.520	0	#DIV/0!
1.566	1.65	1.70	1.70	1.64	1.69	1.70	1.74	1.69	1.71	1.69	1.530	0	#DIV/0!
1.455	1.68	1.69	1.69	1.61	1.66	1.65	1.70	1.64	1.70	1.66	1.508	0	#DIV/0!
0.070	1.67	1.64	1.66	1.61	1.51	1.66	1.67	1.62	1.70	1.48	0.073	0	#DIV/0!
Maximune	1.49	Minimune	0.07										

SF-268						Plate 05 RAW DATA							
1	2	3	4	5	6	7	8	9	10	11	12		
nM	1,250 nM	2,500 nM	5,000 nM	10,000 nM	20,000 nM	1,250 nM	2,500 nM	5,000 nM	10,000 nM	20,000 nM	0	Compound	IC50
0.769	0.905	0.868	0.888	0.978	0.337	0.866	0.865	0.881	0.967	0.343	0.808	Core (1)	8.573
0.070	0.856	0.829	0.842	0.812	0.458	0.842	0.865	0.841	0.868	0.645	0.077	Analogue (2)	>10.0 uM
Maximune	0.797	Minimune	0.071										

SF-268						Plate 06 RAW DATA							
1	2	3	4	5	6	7	8	9	10	11	12		
nM	1,250 nM	2,500 nM	5,000 nM	10,000 nM	20,000 nM	1,250 nM	2,500 nM	5,000 nM	10,000 nM	20,000 nM	0	Compound	IC50
0.071	0.861	0.836	0.842	0.972	0.896	0.848	0.846	0.885	0.987	0.969	0.074	LA Q (3)	>10.0 uM
0.823	0.860	0.874	0.907	1.006	0.400	0.896	0.886	0.911	1.046	0.412	0.821	LA R (4)	9.823
0.805	0.860	0.869	0.804	0.755	0.572	0.901	0.907	0.767	0.735	0.607	0.854	Vx680	>10.0 uM
0.778	0.882	0.933	0.942	0.898	0.901	0.921	0.892	0.901	0.900	0.856	0.825	0.000	#DIV/0!
0.775	0.878	0.869	0.885	0.863	0.883	0.899	0.891	0.867	0.890	0.881	0.800	0.000	#DIV/0!
0.787	0.913	0.875	0.879	0.872	0.852	0.898	0.876	0.870	0.888	0.884	0.806	0.000	#DIV/0!
0.794	0.891	0.880	0.883	0.841	0.859	0.879	0.876	0.837	0.882	0.883	0.813	0.000	#DIV/0!
0.072	0.859	0.823	0.869	0.837	0.815	0.858	0.852	0.865	0.860	0.831	0.073	0.000	#DIV/0!
Maximune	0.818	Minimune	0.073										

Cont'd Table S2.

Detroit551		Plate 07 RAW DATA A											
1	2	3	4	5	6	7	8	9	10	11	12		
nM	1,250 nM	2,500 nM	5,000 nM	10,000 nM	20,000 nM	1,250 nM	2,500 nM	5,000 nM	10,000 nM	20,000 nM	0	**Compound**	IC50
0.293	0.357	0.312	0.346	0.238	0.228	0.323	0.313	0.294	0.262	0.212	0.279	Core (**1**)	>10.0 uM
0.073	0.316	0.297	0.291	0.311	0.172	0.314	0.324	0.311	0.290	0.214	0.076	Analogue (**2**)	9.584
Maximune	0.321	Minimune	0.075										

Detroit551		Plate 08 RAW DATA											
1	2	3	4	5	6	7	8	9	10	11	12		
nM	1,250 nM	2,500 nM	5,000 nM	10,000 nM	20,000 nM	1,250 nM	2,500 nM	5,000 nM	10,000 nM	20,000 nM	0	**Compound**	IC50
0.068	0.241	0.308	0.315	0.286	0.270	0.320	0.307	0.317	0.285	0.324	0.072	LA Q (**3**)	>10.0 uM
0.281	0.225	0.301	0.348	0.310	0.240	0.318	0.342	0.302	0.286	0.281	0.274	LA R (**4**)	>10.0 uM
0.327	0.203	0.346	0.331	0.325	0.181	0.335	0.348	0.348	0.302	0.169	0.272	Vx680	>10.0 uM
0.244	0.267	0.336	0.329	0.325	0.327	0.350	0.331	0.340	0.281	0.389	0.243	0.000	#DIV/0!
0.293	0.348	0.262	0.319	0.337	0.351	0.354	0.333	0.334	0.262	0.375	0.297	0.000	#DIV/0!
0.294	0.319	0.332	0.300	0.308	0.318	0.326	0.305	0.310	0.295	0.367	0.255	0.000	#DIV/0!
0.264	0.322	0.327	0.307	0.313	0.319	0.298	0.306	0.321	0.318	0.362	0.267	0.000	#DIV/0!
0.069	0.354	0.353	0.341	0.286	0.340	0.343	0.359	0.338	0.292	0.378	0.071	0.000	#DIV/0!
Maximune	0.274	Minimune	0.070										

Table S3. Inhibitory Effects on Normal or Cancer Cell Proliferation.

	NCI-H460		SF-268		Detroit 551	
	survival rate at 10 μM (%)	IC_{50} (μM)	survival rate at 10 μM (%)	IC_{50} (μM)	survival rate at 10 μM (%)	IC_{50} (μM)
Core **1**	9.33 ± 0.42	7.00 ± 0.70	37.04 ± 0.41	8.57 ± 0.34	59.00 ± 3.26	>10.00
Analogue **2**	50.08 ± 4.06	>10.00	66.12 ± 12.89	>10.00	47.99 ± 8.58	9.58 ± 0.66
LA Q **3**	106.18 ± 0.16	>10.00	44.79 ± 0.80	9.82 ± 0.08	93.56 ± 10.02	>10.00
LA R **4**	107.35 ± 1.33	>10.00	82.14 ± 38.14	>10.00	104.2 ± 20.67	>10.00

Values represent the mean ± SD of three independent experiments.

Minimum Inhibitory Concetnration (MIC) Determination

The MIC of each compound was determined using broth microdilution method following the guidelines of the Clinical and Laboratory Standards Institute for antimicrobial susceptibility testing.[15] Briefly, on the day of testing, the compound was serial diluted to 2× final test concentrations (0.5 – 8.0 μg/mL) in cation-adjusted Mueller-Hinton broth (CAMHB) containing 0.2% DMSO, and 50 µL of the diluted compound was dispensed into a 96-well plate. Using colonies from an overnight sheep blood agar plate, the test organism (MRSA N216 and Y001) was suspended in dd-H_2O to achieve a turbidity equivalent to 0.5 McFarland standard (approximately ~1-1.5 x 10^8 CFU/mL). The organism suspension was then diluted 1:100 in CAMHB to use as inoculum. Fifty µL of the inoculum was dispensed into wells containing the test compound. Vancomycin was tested as a reference in CAMHB without DMSO on the same 96-well plate. The 96-well plate was then sealed and incubated at 35 ^{o}C ambient air overnight and read at 20 and 24 h after incubation. The MIC was the lowest concentration of compound that inhibited visible growth of the test organism (Tables S4 and S5).

Table S4: Anhydrolandomycinone core (1), Core analogue (2), LA Q (3), LA R (4) and Vancomycin (VA) After Incubation with MRSA 4N216 for 20 and 24 h.

Compound, incubation time	Compound conc. (µg/mL)						Compound conc. (µg/mL)					
	**DMSO**	**0.5**	**1**	**2**	**4**	**8**	**DMSO**	**0.5**	**1**	**2**	**4**	**8**
	1	2	3	4	5	6	7	8	9	10	11	12
Core (**1**), 20 h												
Analogue (**2**), 20 h												
LA R (**4**), 20 h												
LA Q (**3**), 20 h												
VA, 20 h												
Core (**1**), 24 h												
Analogue (**2**), 24 h												
LA R (**4**), 24 h												
LA Q (**3**), 24 h												
VA, 24 h												

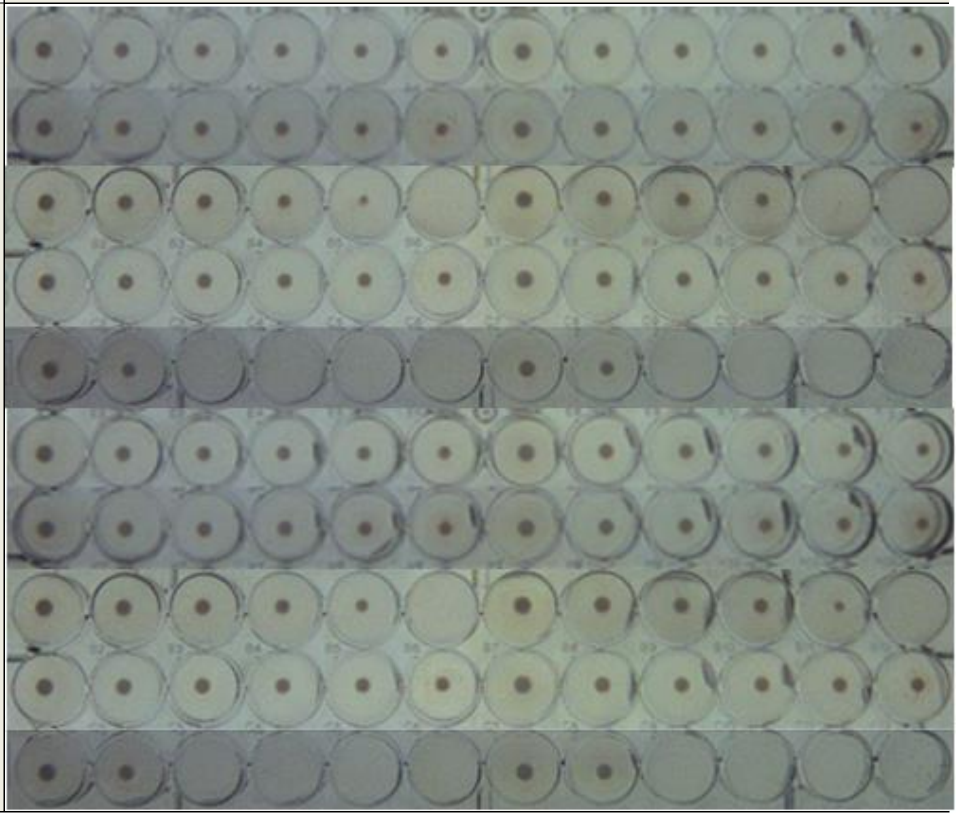

Table S5: Anhydrolandomycinone core (1), Core analogue (2), LA Q (3), LA R (4) and Vancomycin (VA) After Incubation with MRSA 7Y001 for 20 and 24 h

**Compound, incubation time**	**Compound conc. (µg/mL)**						**Compound conc. (µg/mL)**					
	**DMSO**	**0.5**	**1**	**2**	**4**	**8**	**DMSO**	**0.5**	**1**	**2**	**4**	**8**
	1	2	3	4	5	6	7	8	9	10	11	12
Core (**1**), 20 h	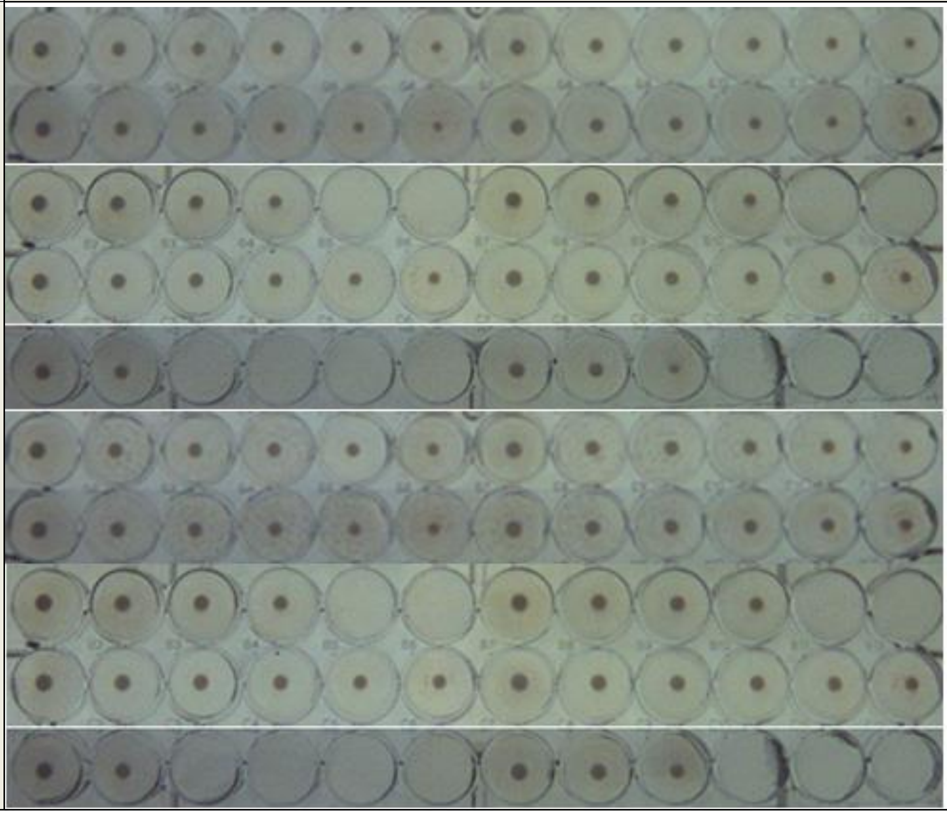											
Analogue (**2**), 20 h												
LA R (**4**), 20 h												
LA Q (**3**), 20 h												
VA, 20 h												
Core (**1**), 24 h												
Analogue (**2**), 24 h												
LA R (**4**), 24 h												
LA Q (**3**), 24 h												
VA, 24 h												

4. References

1. K. A. Shaaban, S. Srinivasan, R. Kumar, C. Damodaran and J. Rohr, Landomycins P-W, Cytotoxic Angucyclines from *Streptomyces cyanogenus* S136, *J. Nat. Rep*., 2011, **74**, 2-11.
2. For D-ring intermediate **9**: F. Falchi, S. M. Bertozzi, G. Ottonello, G. F. Ruda, G. Colombano, C. Fiorelli, C. Martucci, R. Bertorelli, R. Scarpelli, A. Cavalli, T. Bandiera and A. Armirotti, A New Kernal Based, Partial Least Squares, QSRR Model for UPLC Retention Time Prediction: A Useful Tool for Metabolite Identification, *Anal. Chem*., 2016, **88**, 9510–9517.
3. J.-C. Liu, J.-J Fu, W.-L. Li, Y. Zou, Z.-J. Huang, J.-Y. Xu, S.-X. Peng and Y.-H. Zhang, Utilization of the inherent nucleophile for regioselective O-acylation of polyphenols via an intermolecular cooperative transesterification, *Tetrahedron Lett.*, 2016, **72**, 4103-4110.
4. S. Yamaguchi, H. Tanaka, R. Yamada, S. Kawauchi, and T. Takahashi, Synthesis of a landomycinone skeleton via Masamune-Bergmann cyclization, *RSC Adv.*, 2014, **4**, 32241-32248.
5. C.-J. Sie, V. Patteti, Y.-R. Yang and T. K.-K. Mong, A general strategy for diverse syntheses of anhydrolandomycinone, tetrangulol, and landomycinone, *Chem. Commun*., 2018, **54**, 1885-1888.
6. For intermediates **19A**, **19B**, and **19C**: T. B. Durham and W. R. Roush, Stereoselective synthesis of functionalized precursors of the CDEF and CDE 2,6-dideoxy-tetra- and trisaccharide units of durhamycins A and B, *Org. Lett.*, 2003, **5**, 1875-1878.
7. For intermediate **19D**: K. Tatsuta, K. Akimoto, M. Annaka, Y. Ohno and M. Kinoshita, Enantiodivergent total syntheses of nanaomycins and their enantiomers, kalafungins, *Bull. Chem. Soc. Jpn.*, 1985, **58**, 1699-1706.
8. For rhodinose acetate **19**: O. Calin, R. Pragani and P. H. Seeberger, *De novo* Synthesis of L-Colitose and L-Rhodinose Building Blocks, *J. Org. Chem.* 2012, **77**, 870-877.
9. For glucal substrate **23**: W. Roth, W. Pigman, D-Glucal and the Glycals. D-Glucal and 6-Deoxy-L-Dlucal, *Methods in Carbohydr. Chem*., 1963, 405-408.
10. For olivosyl acetate building blocks **20**, **22**, **23D**, and intermediate **23A**: X. Yang, B. Fu and B. Yu, Total synthesis of landomycin A, a potent antitumor angucycline antibiotic. *J. Am. Chem. Soc*., 2011, **133**, 12433-12435.
11. For glucal intermediate **23C**: S. Kopper, D. Springer and J. Thiem, Unusual Formation of a β-Linked Disaccharide in a NIS Glycosylation, *J. Carbohydr. Chem.*, 1994, **13**, 1065-1070.
12. For intermediate **24**: D. Crich, T. Ritchie, Silyl Migration and Formation of an Anhydro Derivative on Attempted Benzylation of 3-O-*tert*-Butyldimethylsilyl-6-*O*-Tosyl-D-Glucal, *Carbohydr. Res*. 1990, **197**, 324-326.
13. H. R. Billica and H. Adkins, Catalyst, Raney Nickel, W-6, *Org. Synth.*, 1949, **29**, 24 and references cited therein.
14. MTS Assay: (a) C. J. Goodwin, S. J. Holt, S. Downes and N. J. Marshall, Microculture Tetrazolium Assays: a Comparison between Two New Tetrazolium Salts, XTT and MTS, *J. Immunol. Method*, 1995, **179**, 95-103. (b) Promega, CellTiter96 AQueous Non-Radioactive Cell Proliferation Assay: Technical Bulletin No 169, Promega Corporation, Madison, USA, 2001.
15. MIC assay: (a) CLSI. Methods for dilution antimicrobial susceptibility tests for bacteria that grow aerobically. 11th ed. CLSI standard M07. Wayne, PA: Clinical and Laboratory Standards Institute;

2018. (b) F.-J. Chen, T.-L. Lauderdale, C.-H. Lee, Y.-C. Hsu, I.-W. Huang, P.-C. Hsu and C.-S. Yang, Effect of a point mutation in mprF on susceptibility to daptomycin, vancomycin, and oxacillin in an MRSA clinical strain, *Front Microbiol*., 2018, **9**, 1086

^{1}H NMR spectrum of 4-(Benzyloxy)-3-bromophenyl Acetate **9**

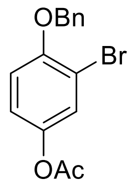

9

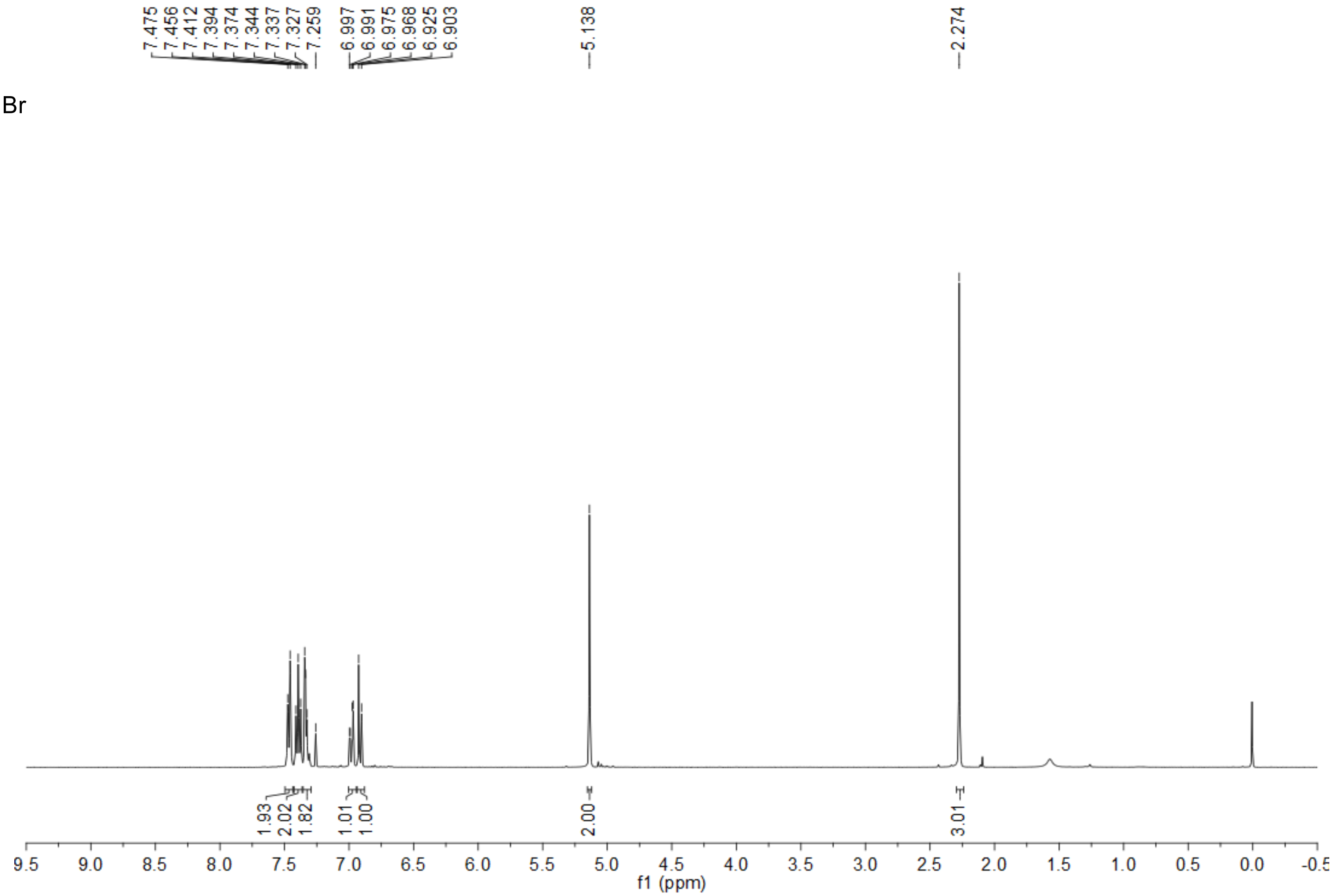

^{13}C NMR spectrum of 4-(Benzyloxy)-3-bromophenyl Acetate **9**

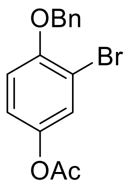

9

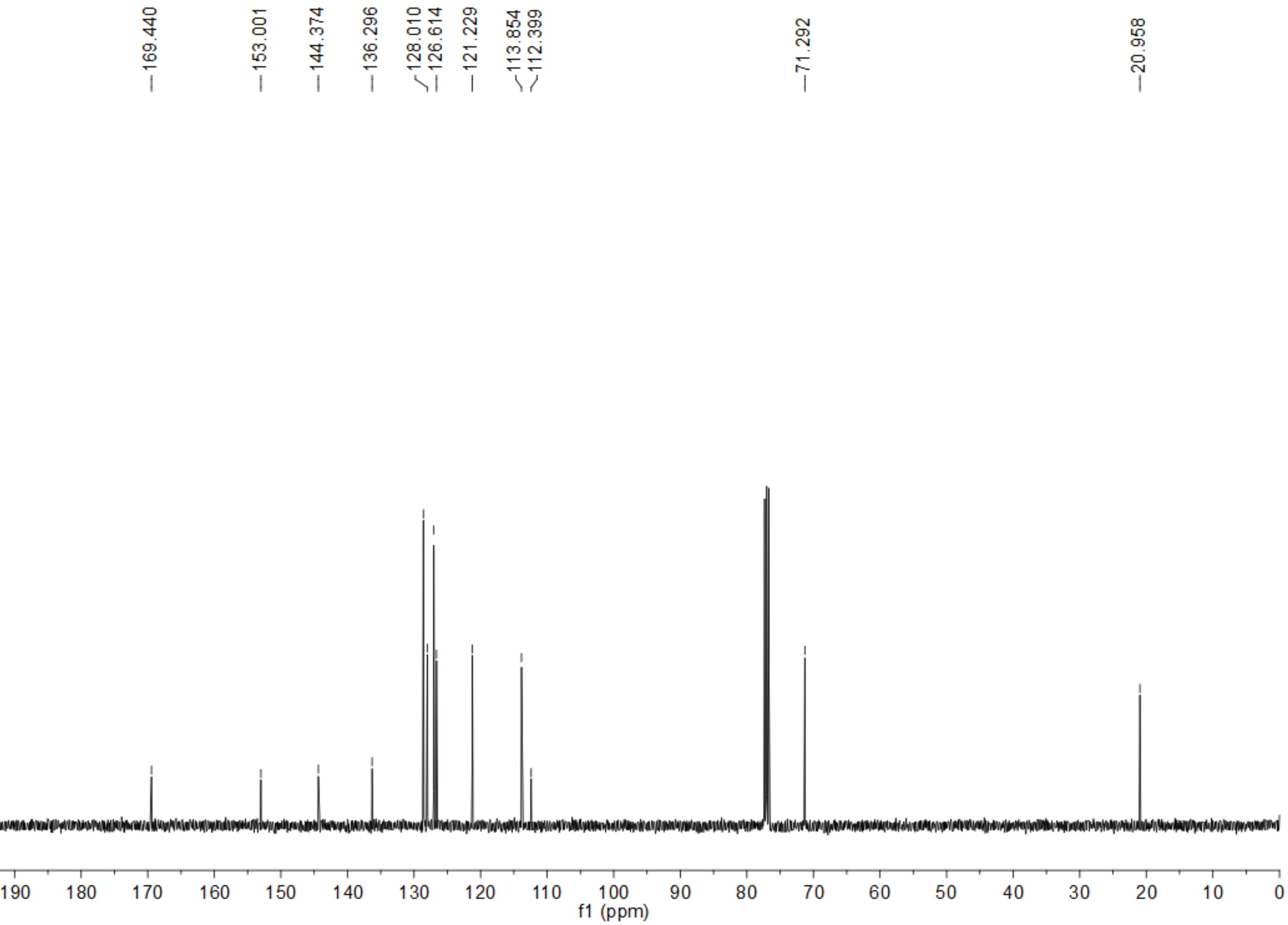

^{1}H NMR spectrum of 1-(Benzyloxy)-2-bromo-4-(methoxymethoxy)benzene **10**

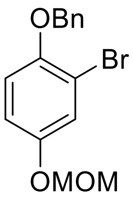

10

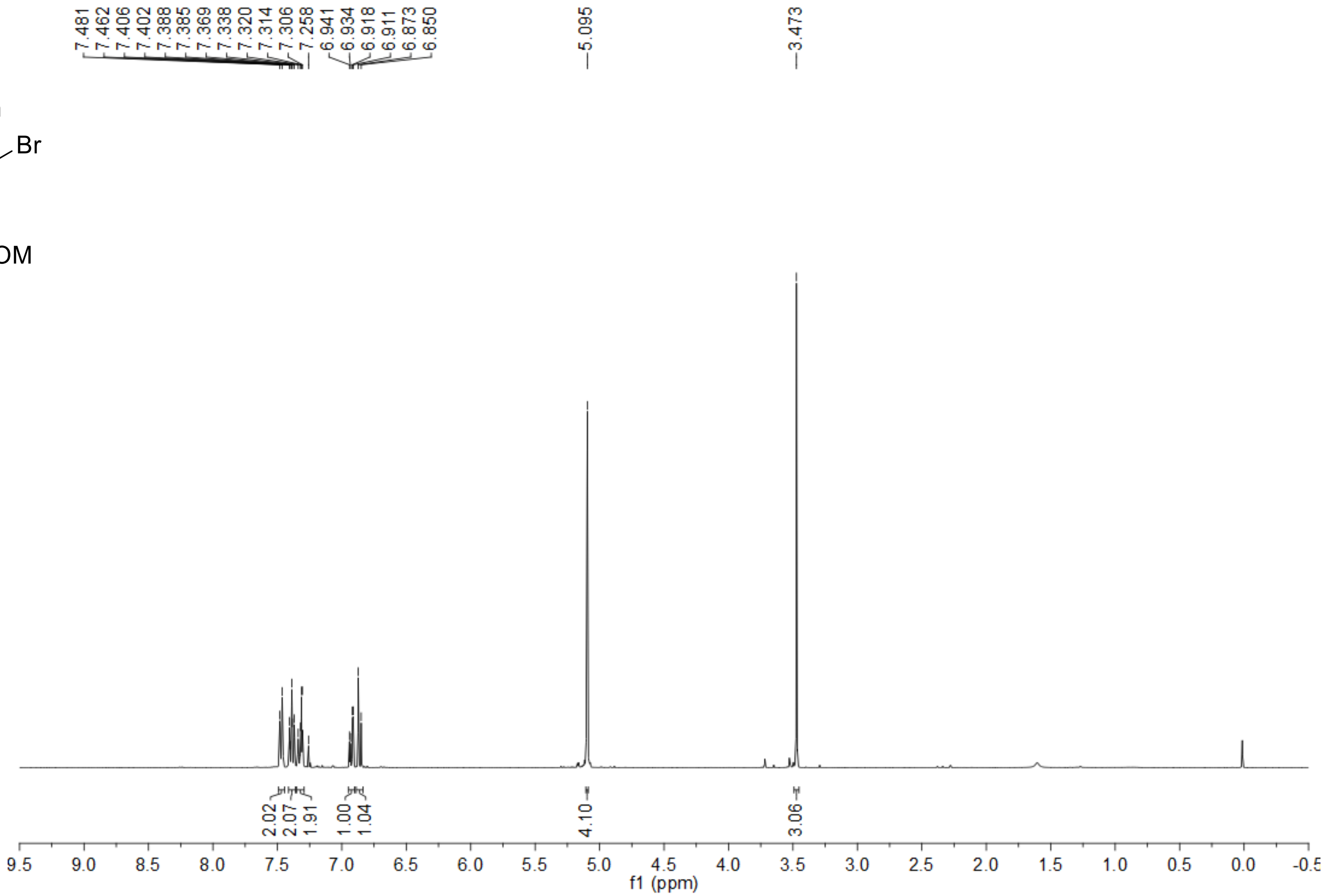

^{13}C NMR spectrum of 1-(Benzyloxy)-2-bromo-4-(methoxymethoxy)benzene **10**

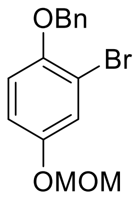

10

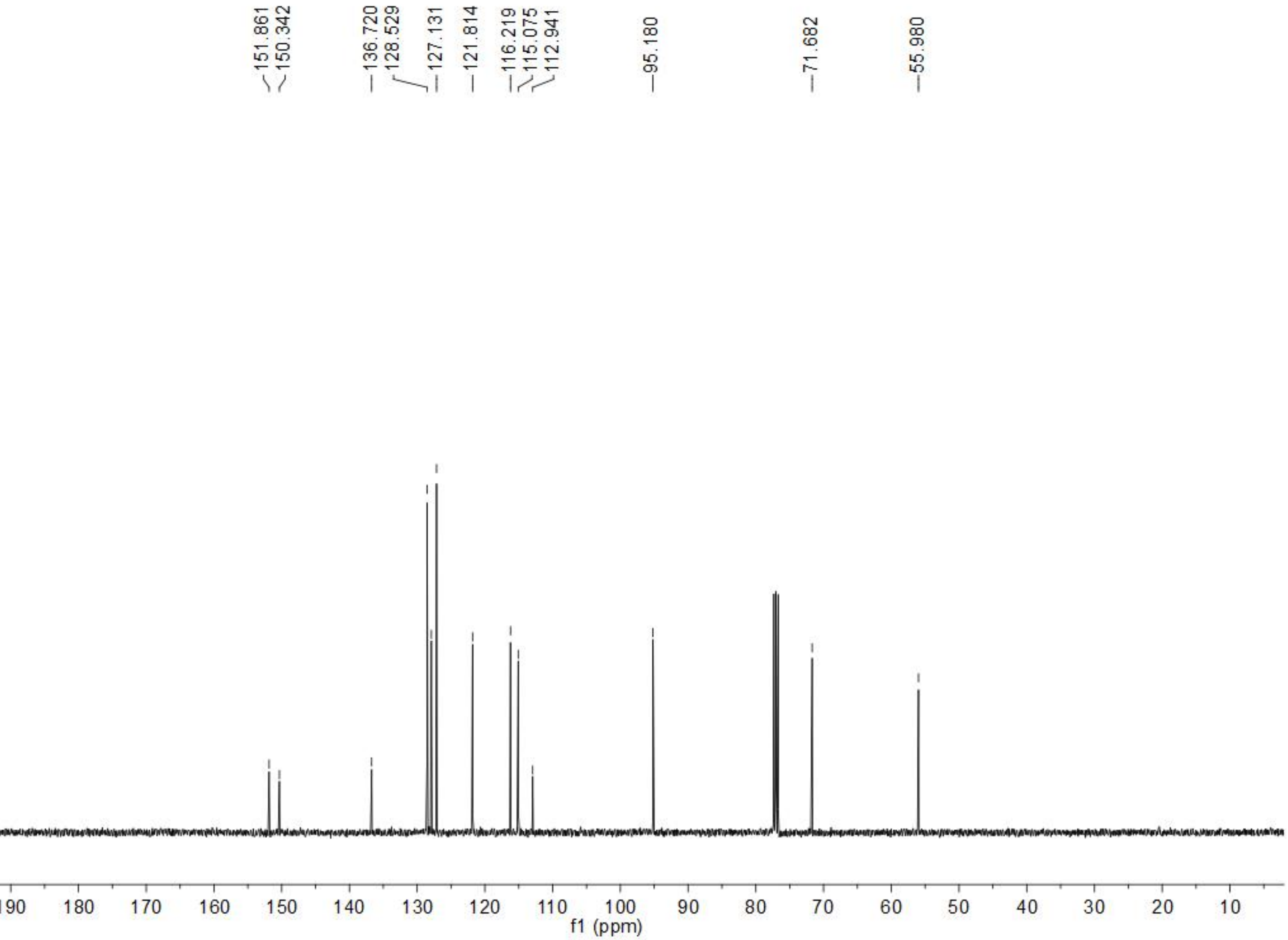

^{1}H NMR spectrum of (2-Iodo-3-methoxy-5-methylphenyl)methanol **12**

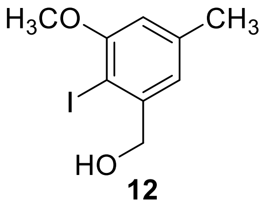

12

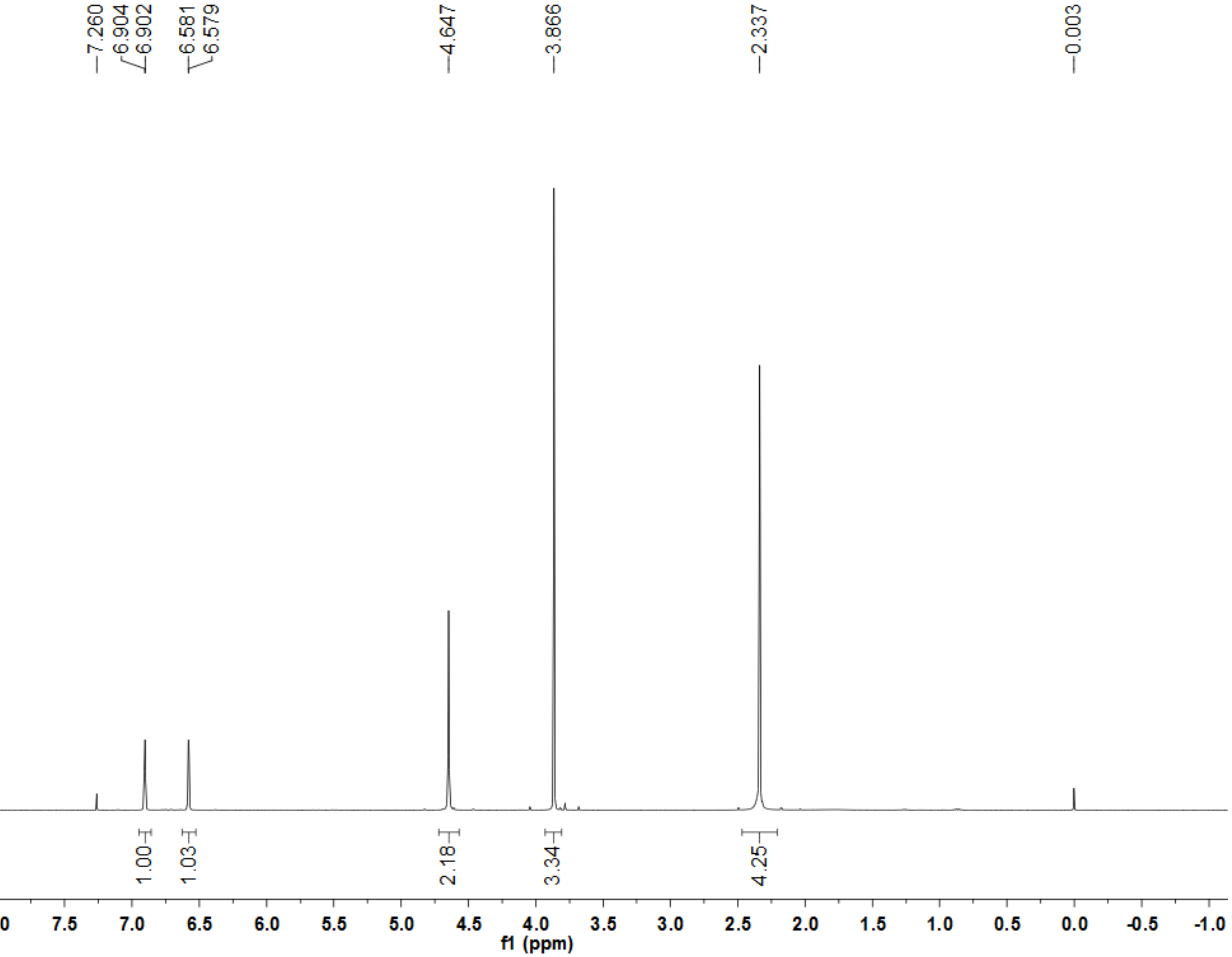

^{13}C NMR spectrum of (2-Iodo-3-methoxy-5-methylphenyl)methanol **12**

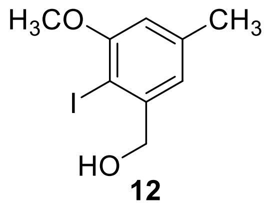

12

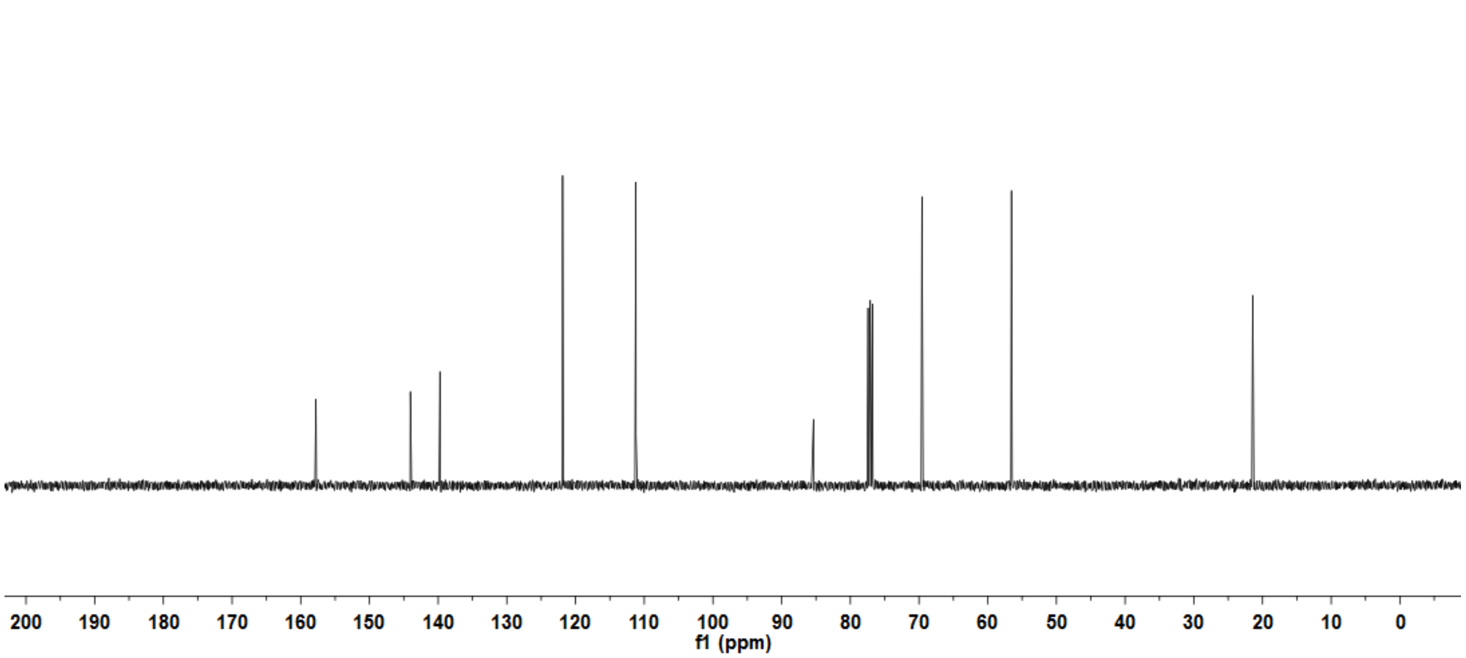

^{1}H NMR spectrum of 2-Iodo-3-methoxy-5-methylbenzyl Acetate **12A**

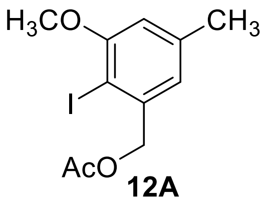

12A

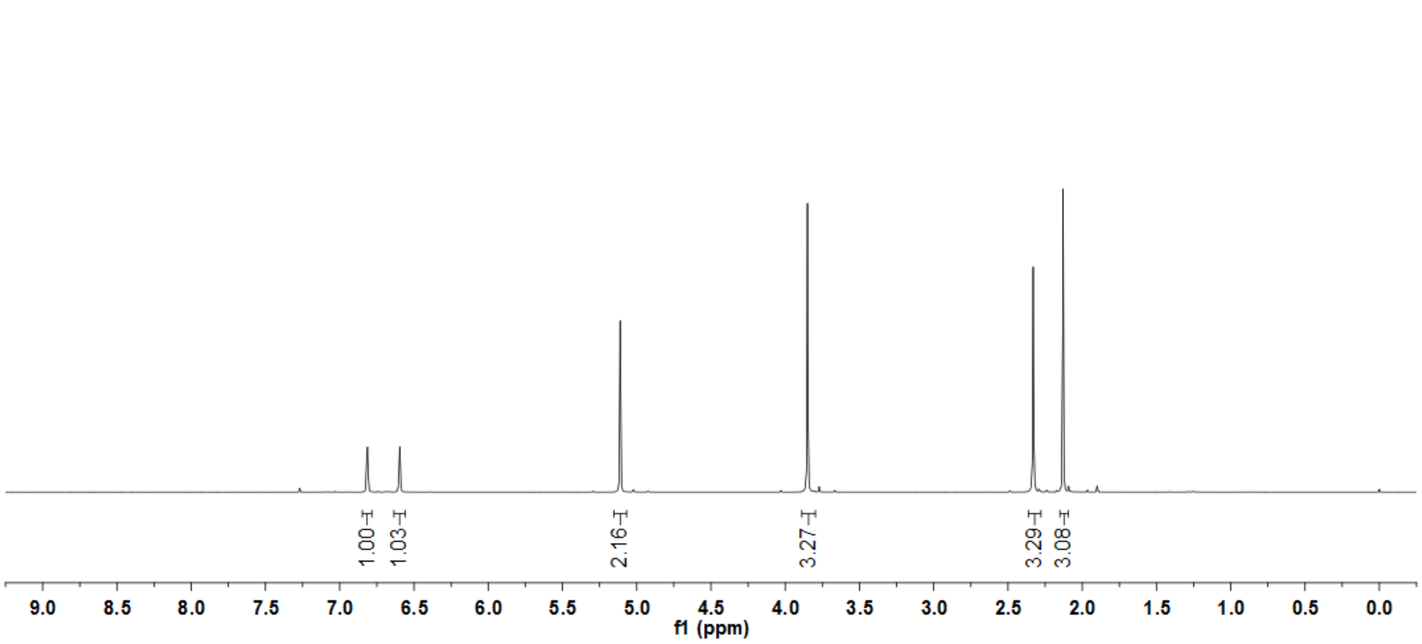

^{13}C NMR spectrum of 2-Iodo-3-methoxy-5-methylbenzyl Acetate **12A**

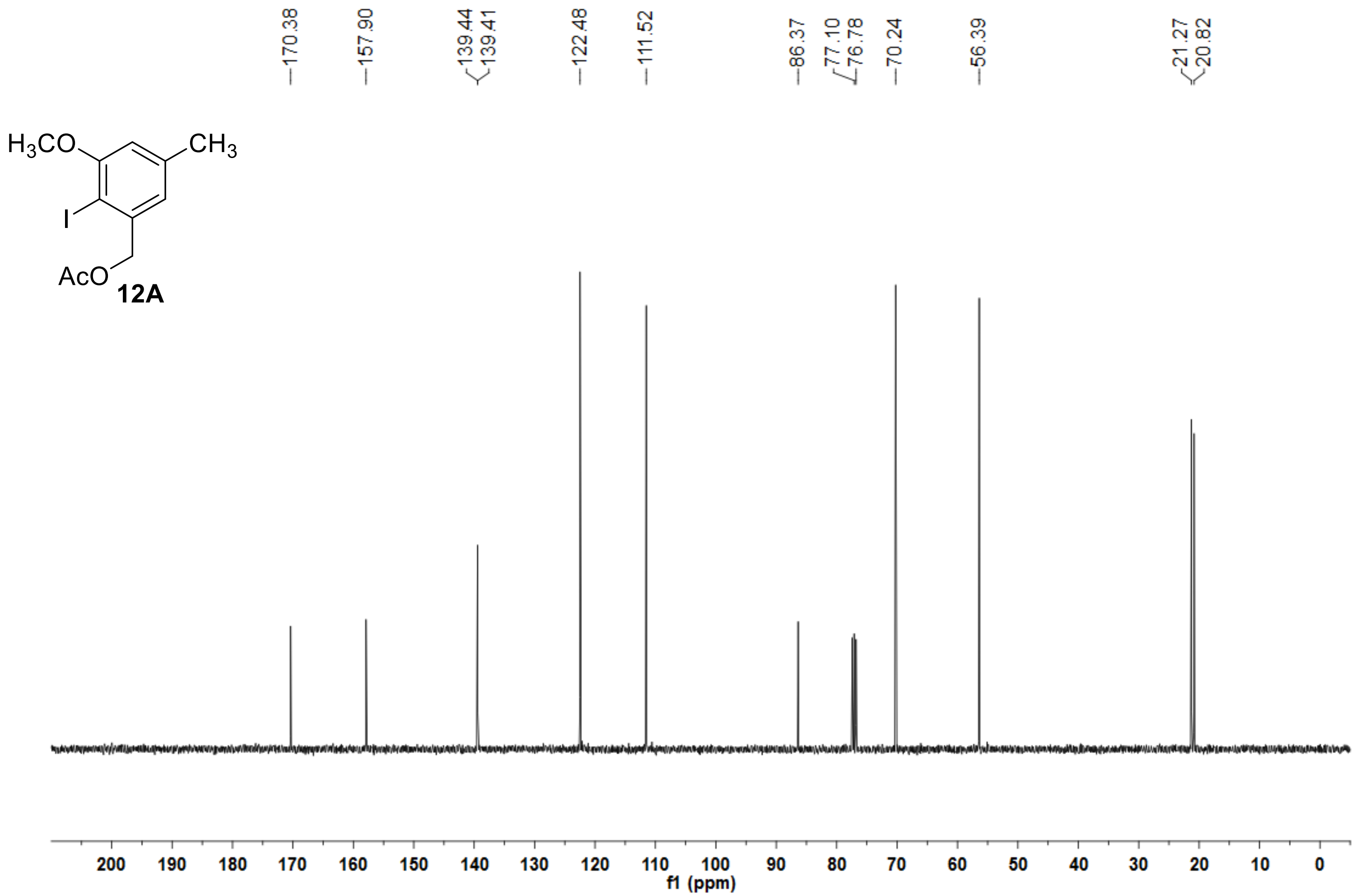

^{1}H NMR spectrum of 3-Bromo-2-iodo-5-methylphenol **12B**

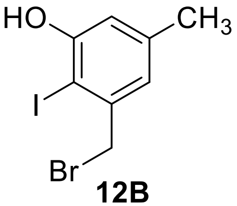

12B

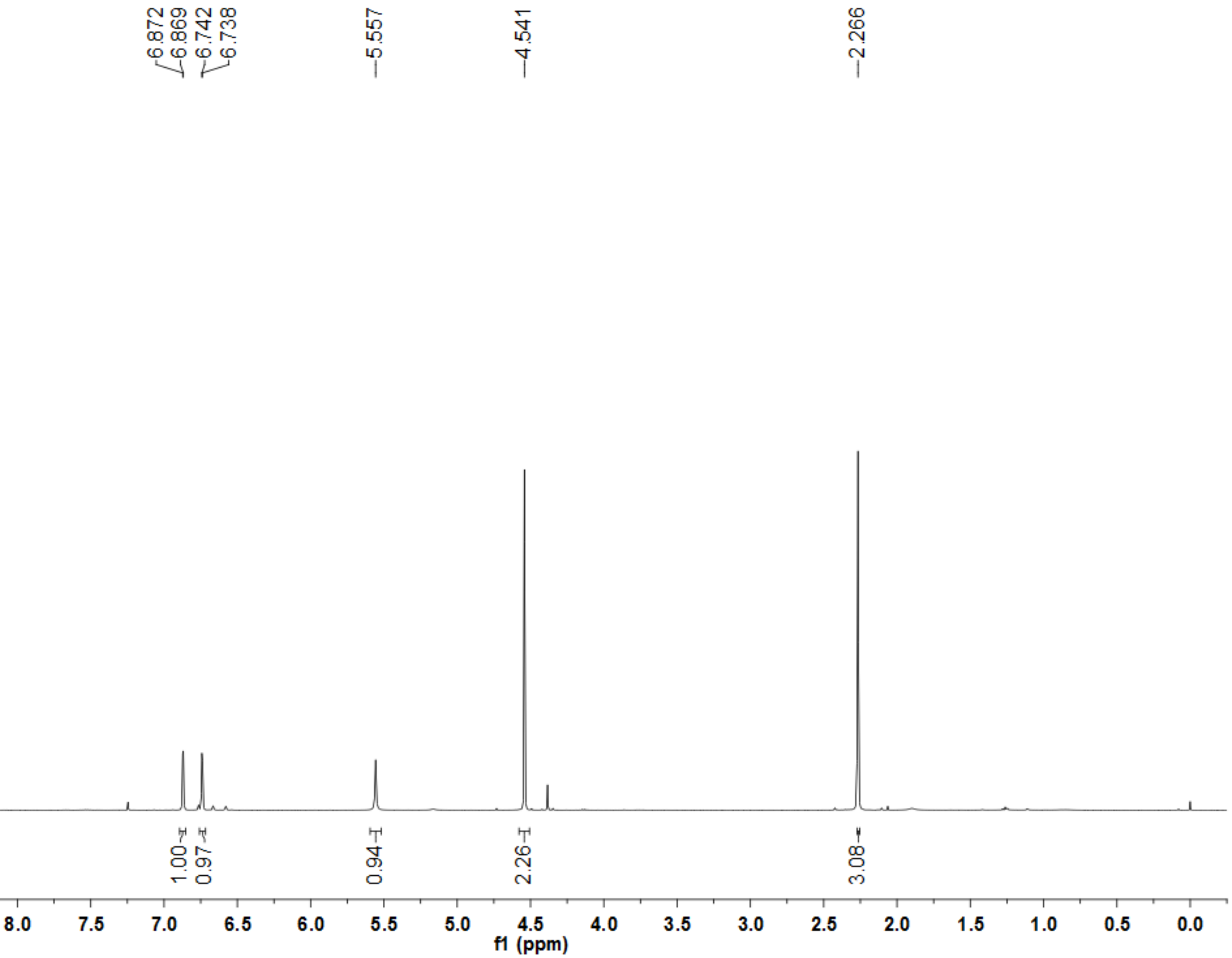

^{13}C NMR spectrum of 3-Bromo-2-iodo-5-methylphenol **12B**

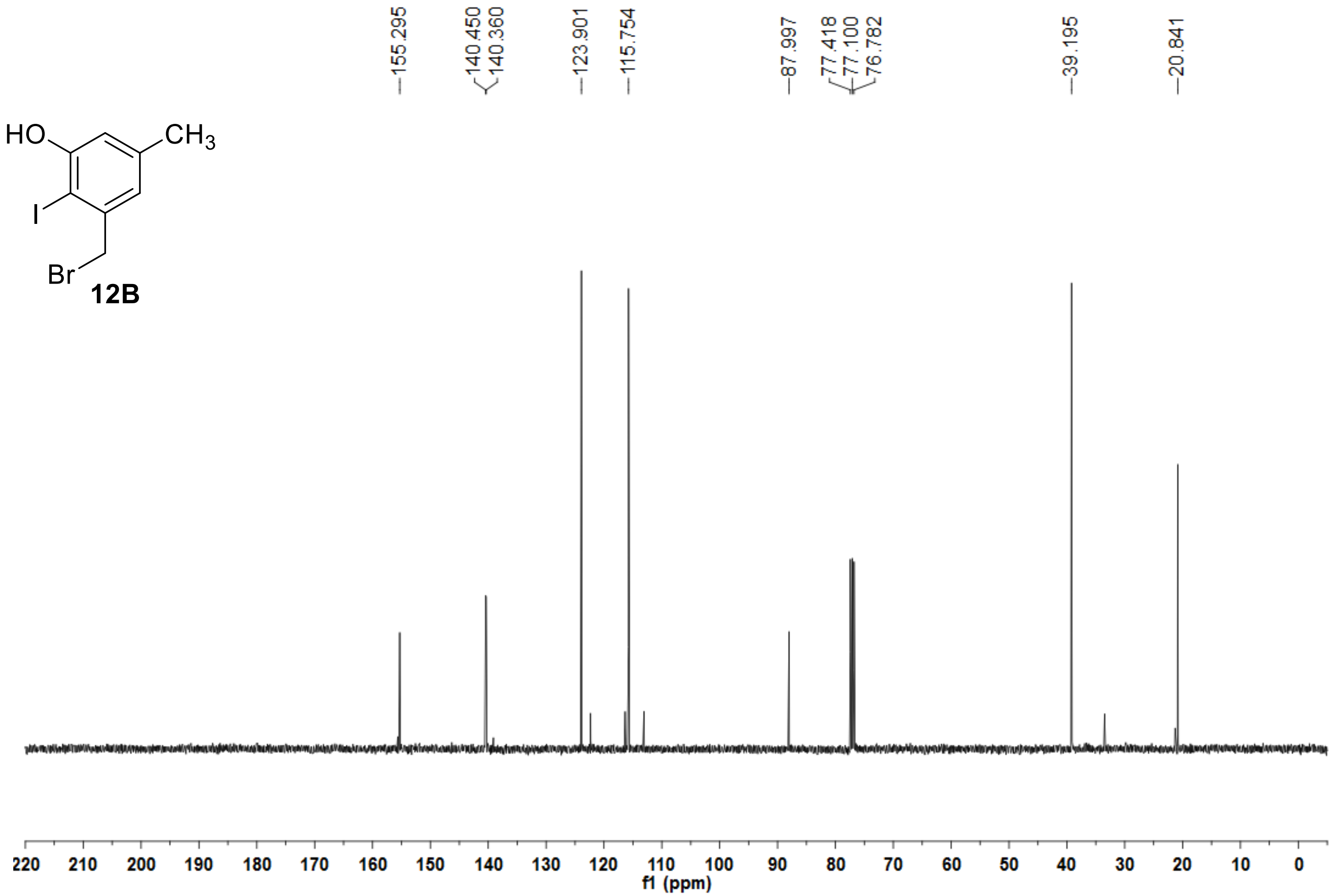

^{1}H NMR spectrum of 1-(Benzyloxy)-3-(bromomethyl)-2-iodo-5-methylbenzene **13**

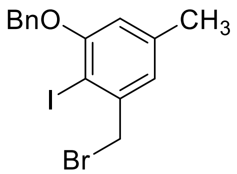

13

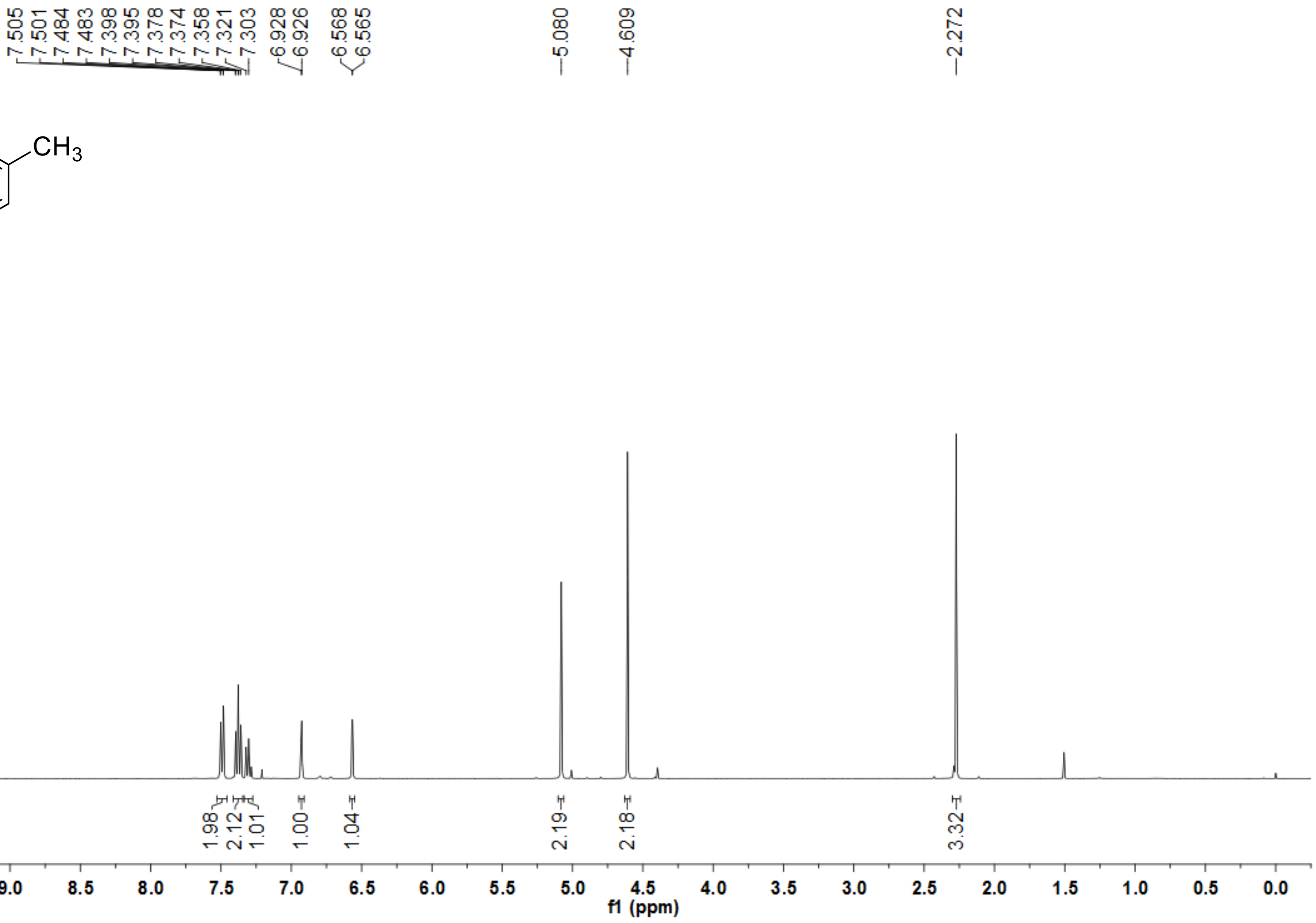

^{13}C NMR spectrum of 1-(Benzyloxy)-3-(bromomethyl)-2-iodo-5-methylbenzene **13**

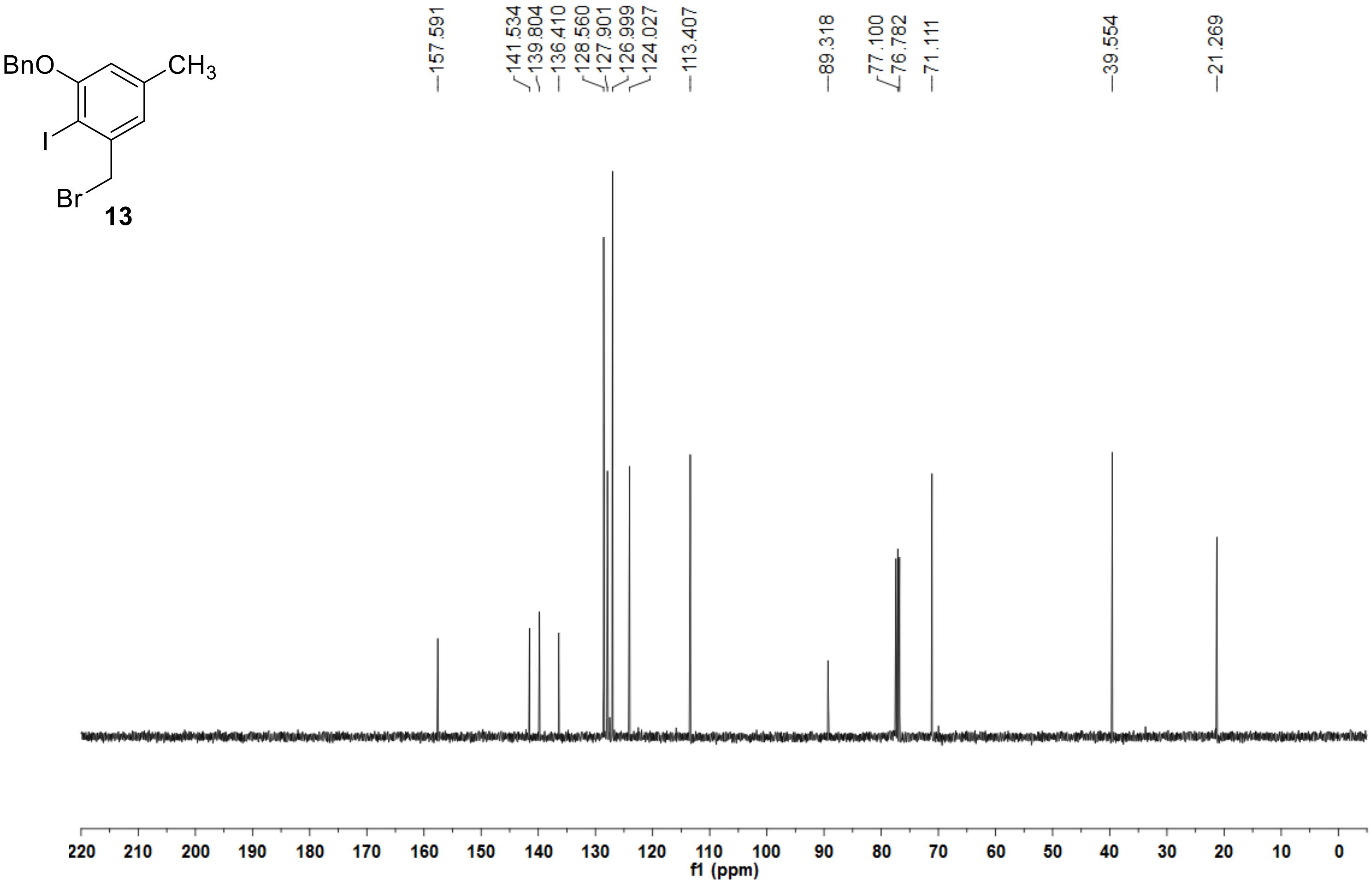

^{1}H NMR spectrum of 1-(Benzyloxy)-3-(but-3-yn-1-yl)-2-iodo-5-methylbenzene **6**

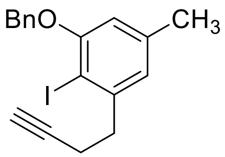

6

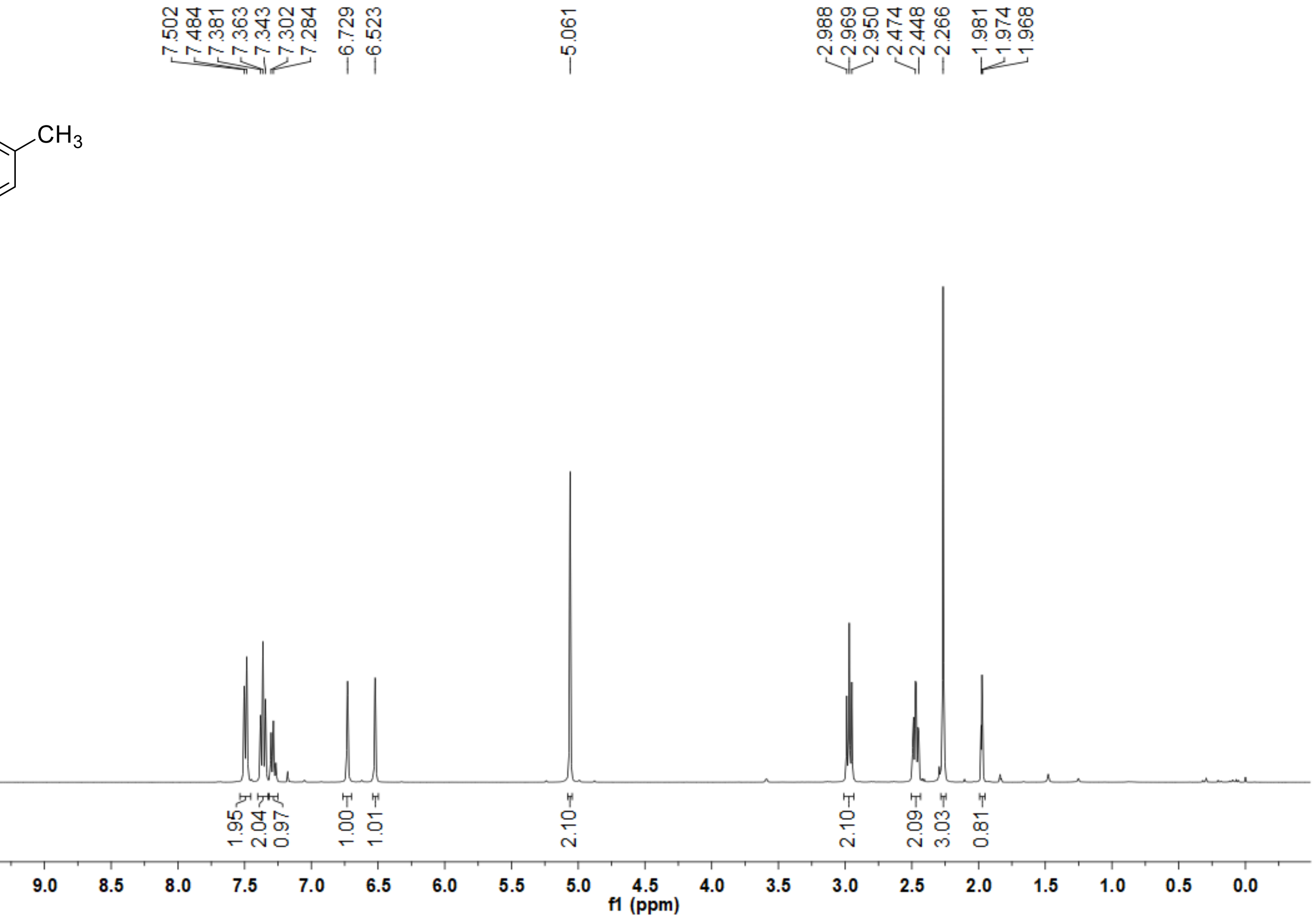

^{13}C NMR spectrum of 1-(Benzyloxy)-3-(but-3-yn-1-yl)-2-iodo-5-methylbenzene **6**

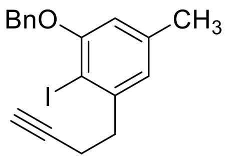

6

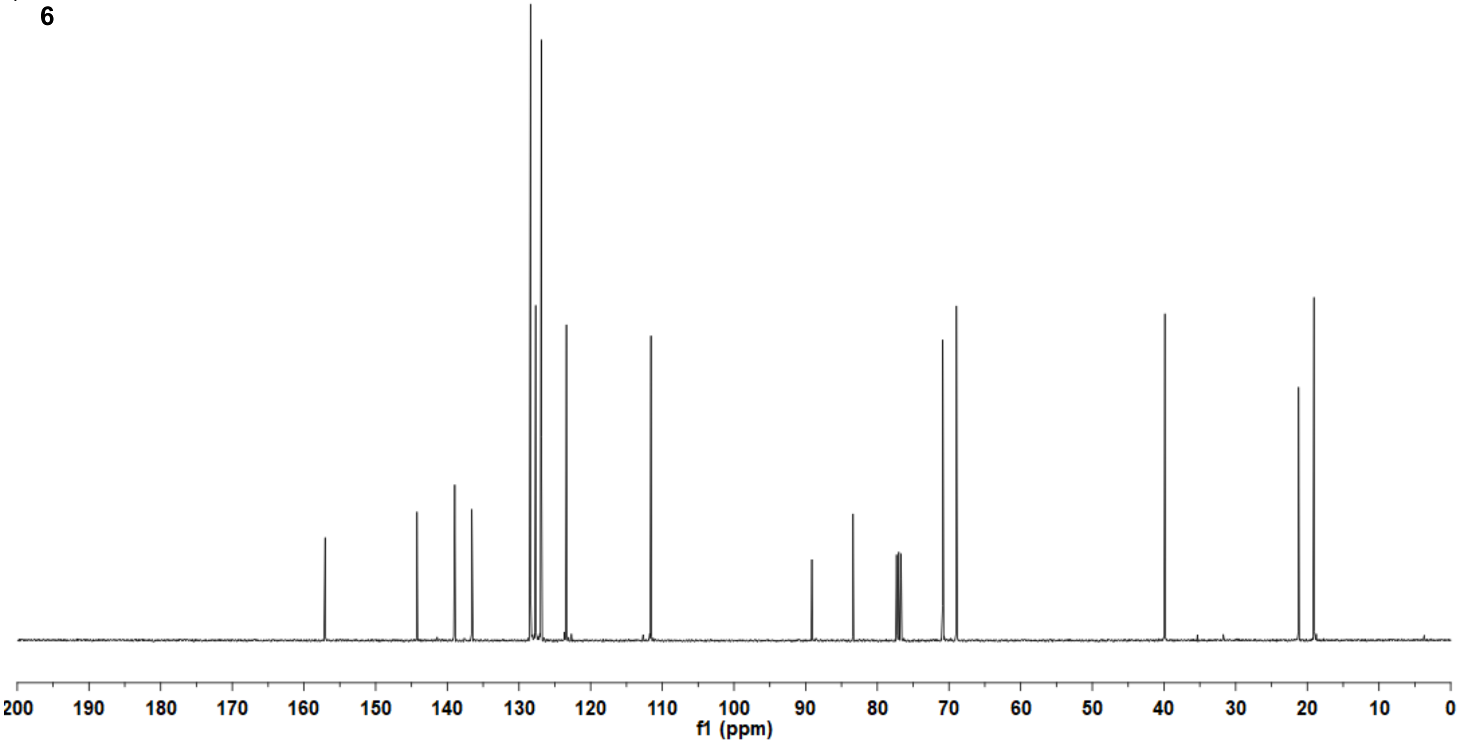

^{1}H NMR spectrum of 5-Benzyloxy-2-(3-benzyloxy-2-iodo-5-methylphenethyl)-8-(methoxymethoxy)naphthalene-1,4-dione **14A**

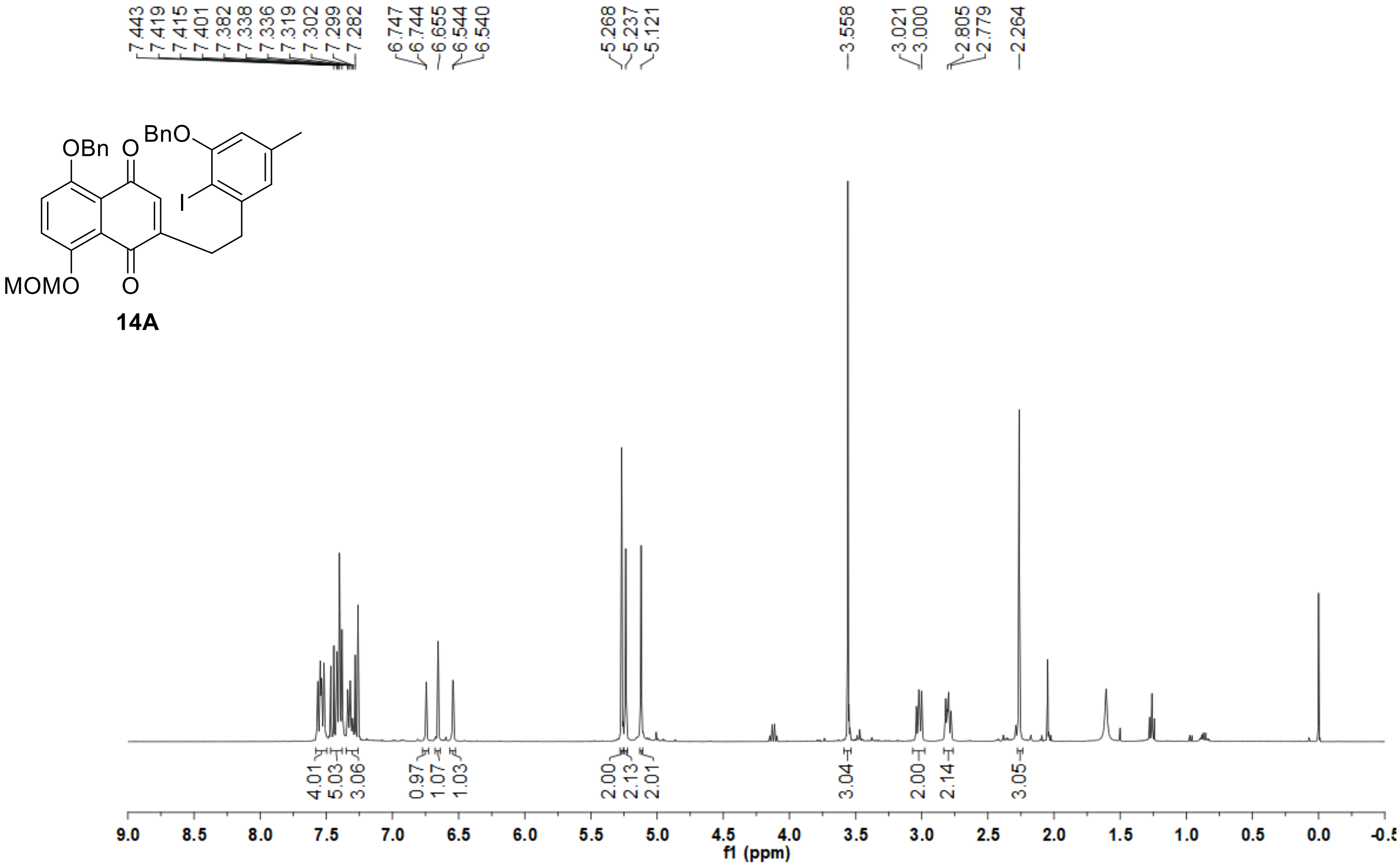

^{13}C NMR spectrum of 5-Benzyloxy-2-(3-benzyloxy-2-iodo-5-methylphenethyl)-8-(methoxymethoxy)naphthalene-1,4-dione **14A**

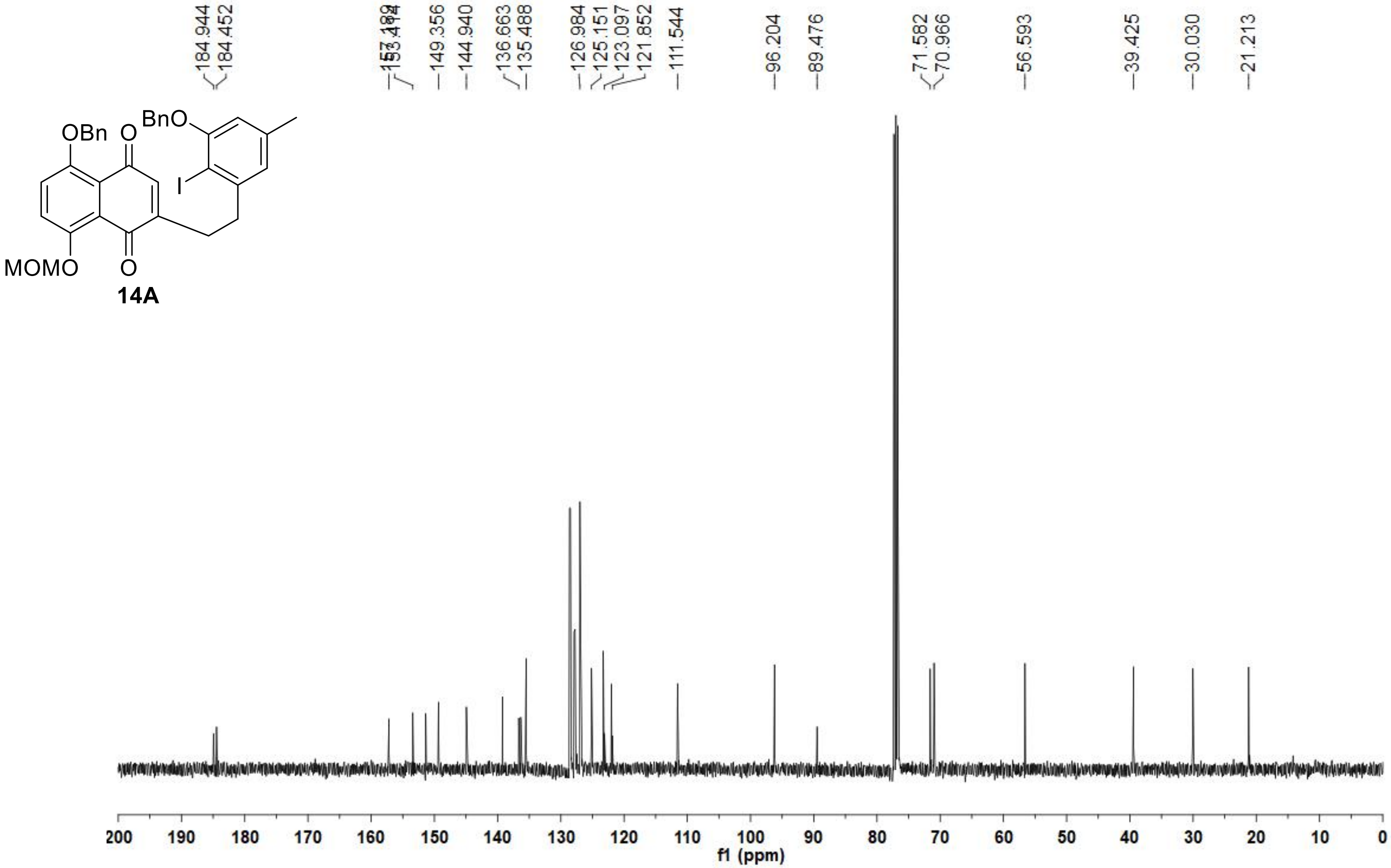

HSQC NMR spectrum of 5-Benzyloxy-2-(3-benzyloxy-2-iodo-5-methylphenethyl)-8-(methoxymethoxy)naphthalene-1,4-dione **14A**

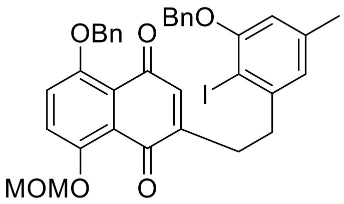

14A

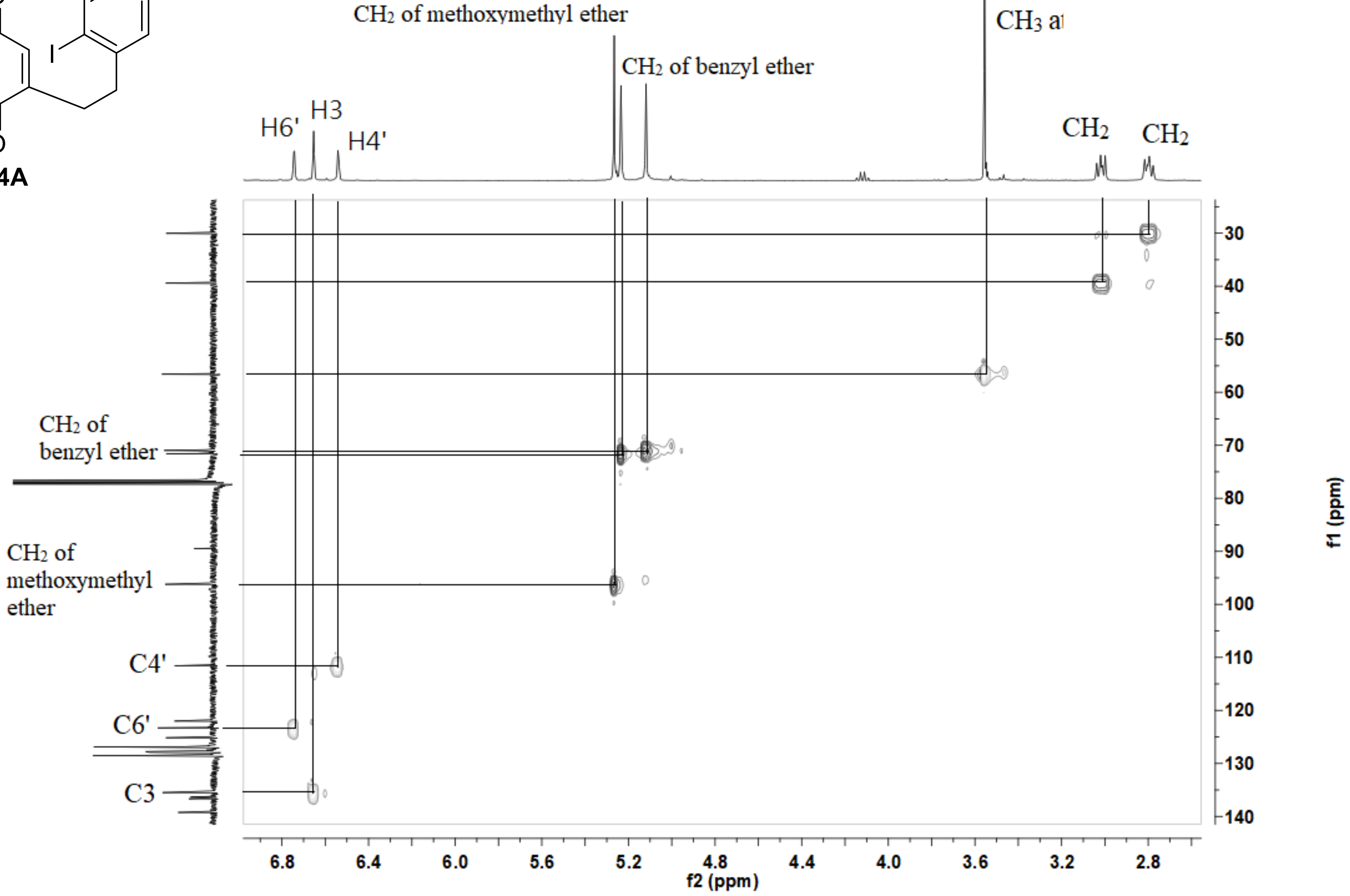

^{1}H NMR spectrum of 5-Benzyloxy-2-(3-benzyloxy-2-iodo-5-methylphenethyl)-8-(methoxymethoxy)naphthalene-1,4-diyl diacetate **15**

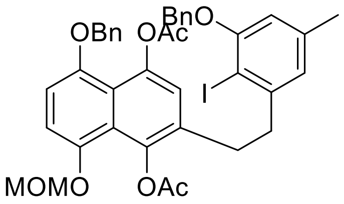

15

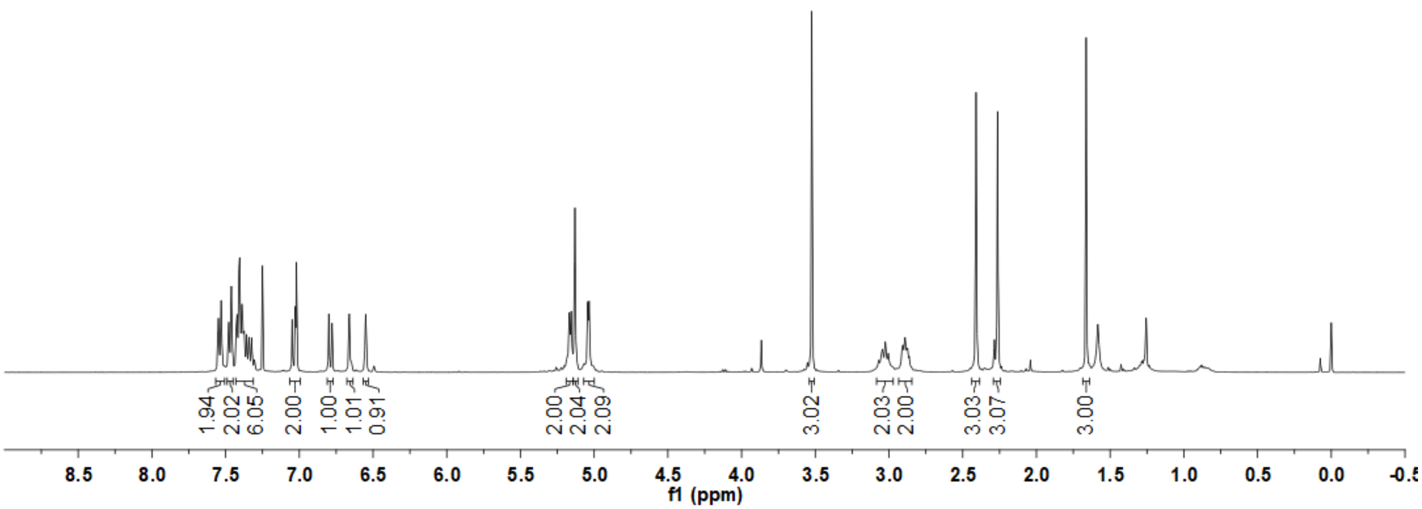

^{13}C NMR spectrum of 5-Benzyloxy-2-(3-benzyloxy-2-iodo-5-methylphenethyl)-8-(methoxymethoxy)naphthalene-1,4-diyl Diacetate **15**

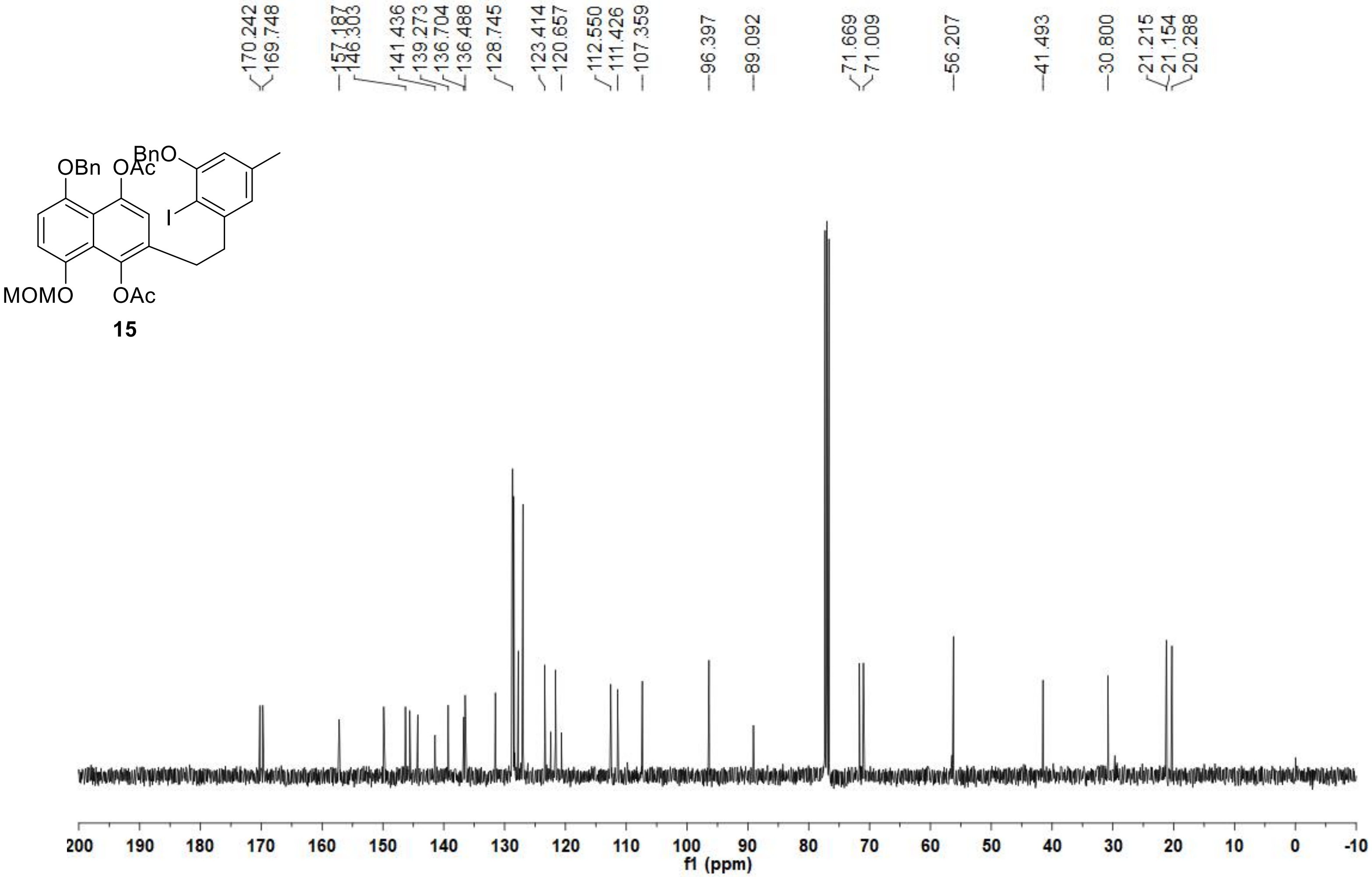

HSQC NMR spectrum of 5-Benzyloxy-2-(3-benzyloxy-2-iodo-5-methylphenethyl)-8-(methoxymethoxy)naphthalene-1,4-diyl Diacetate **15**

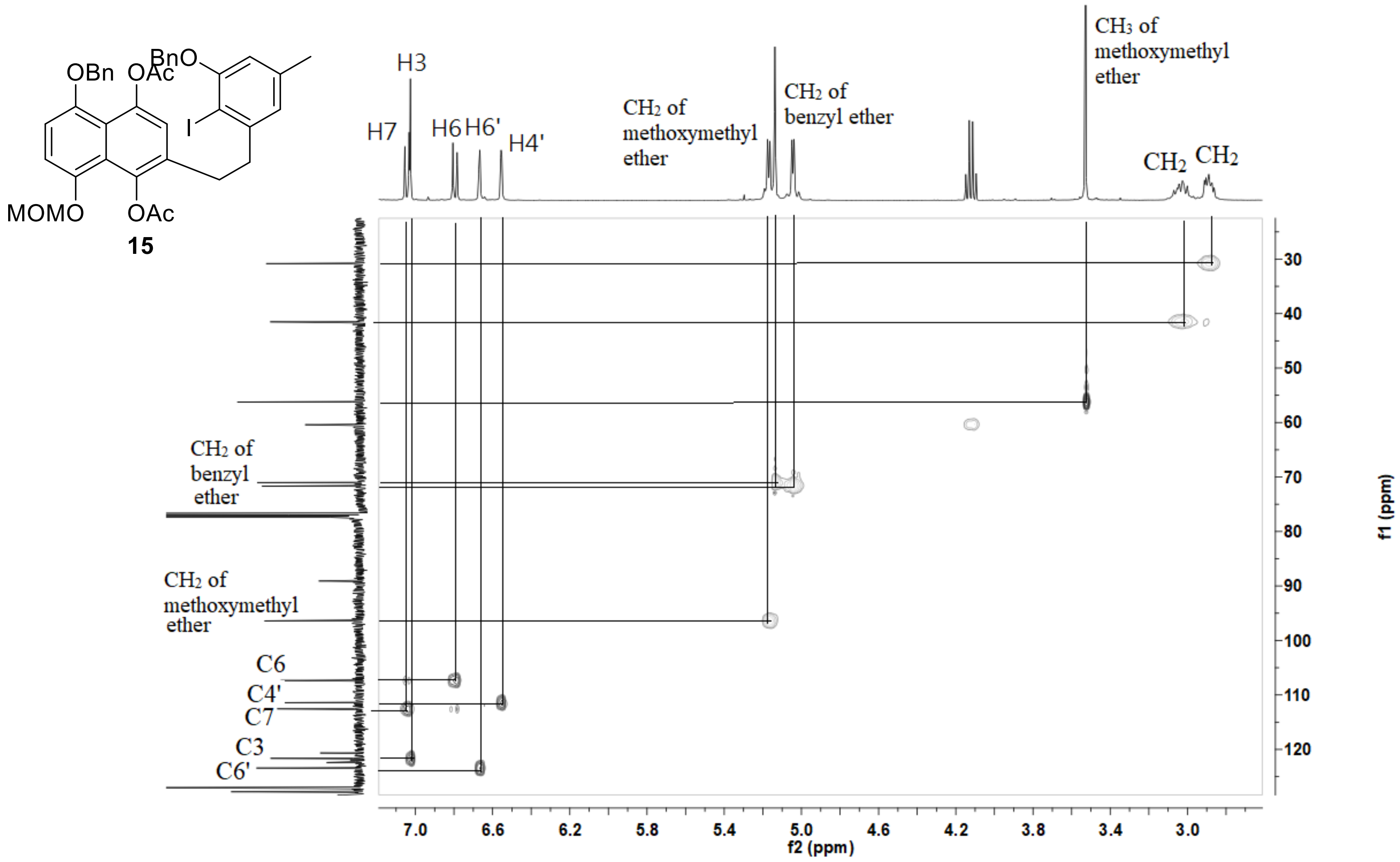

^{1}H NMR spectrum of 1,11-Bis(benzyloxy)-8-(methoxymethoxy)-3-methyl-5,6-dihydrotetraphene-7,12-diyl Diacetate **16**

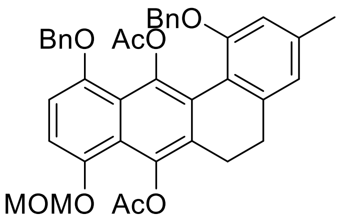

16

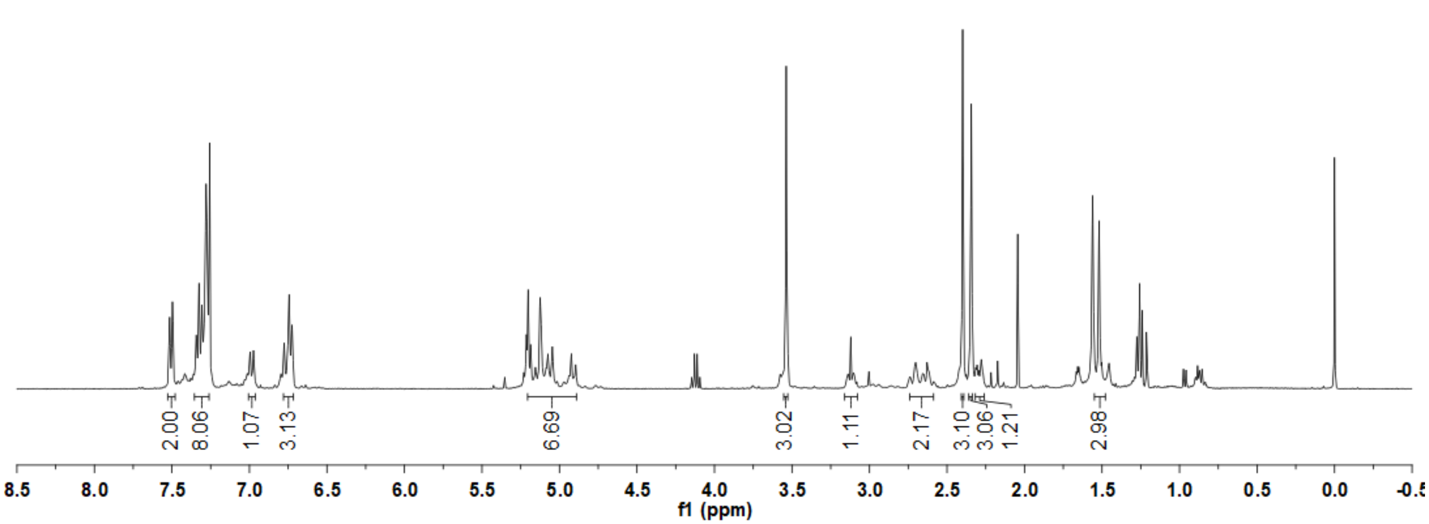

^{13}C NMR spectrum of 1,11-Bis(benzyloxy)-8-(methoxymethoxy)-3-methyl-5,6-dihydrotetraphene-7,12-diyl Diacetate **16**

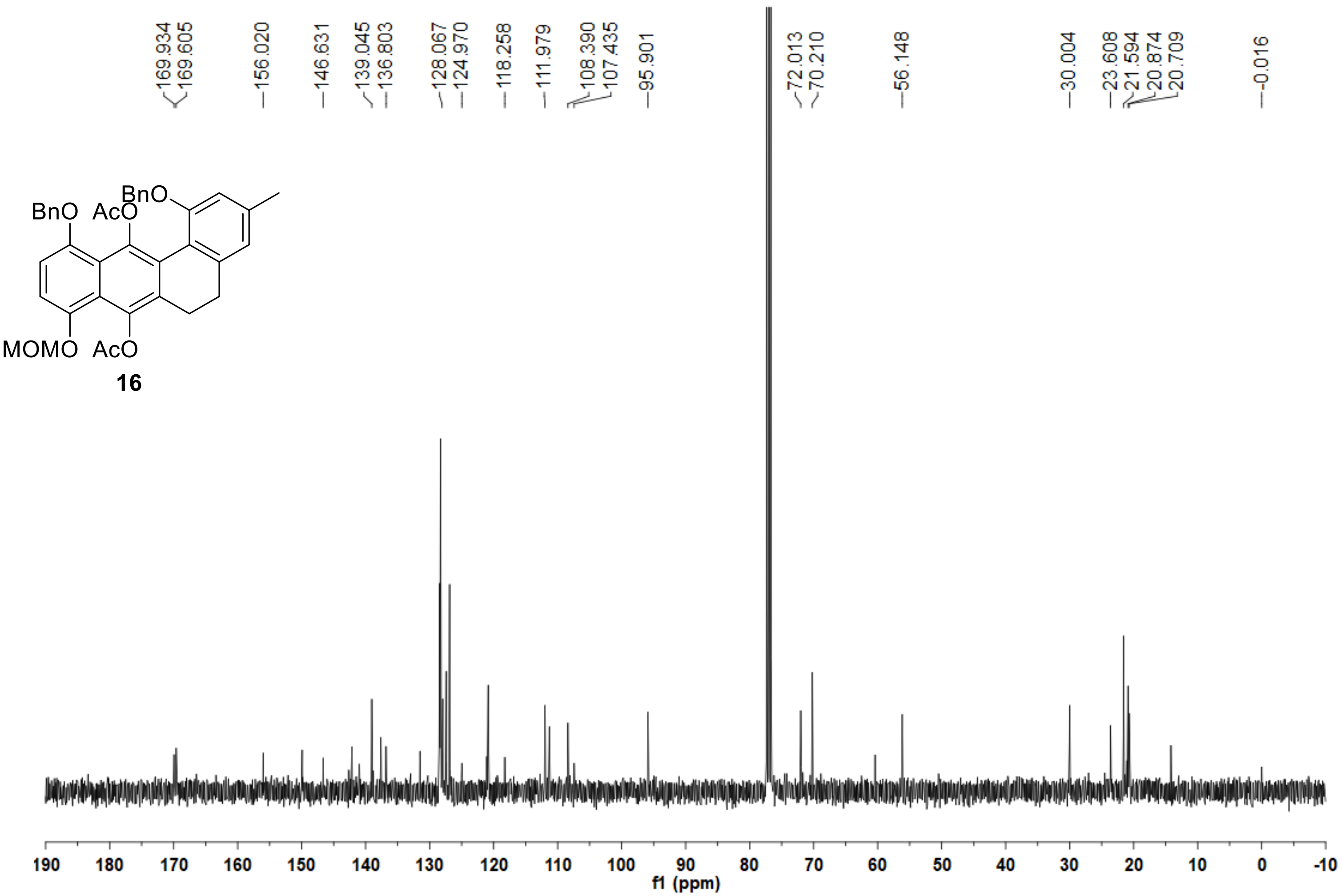

16

HSQC NMR spectrum of 1,11-Bis(benzyloxy)-8-(methoxymethoxy)-3-methyl-5,6-dihydrotetraphene-7,12-diyl Diacetate **16**

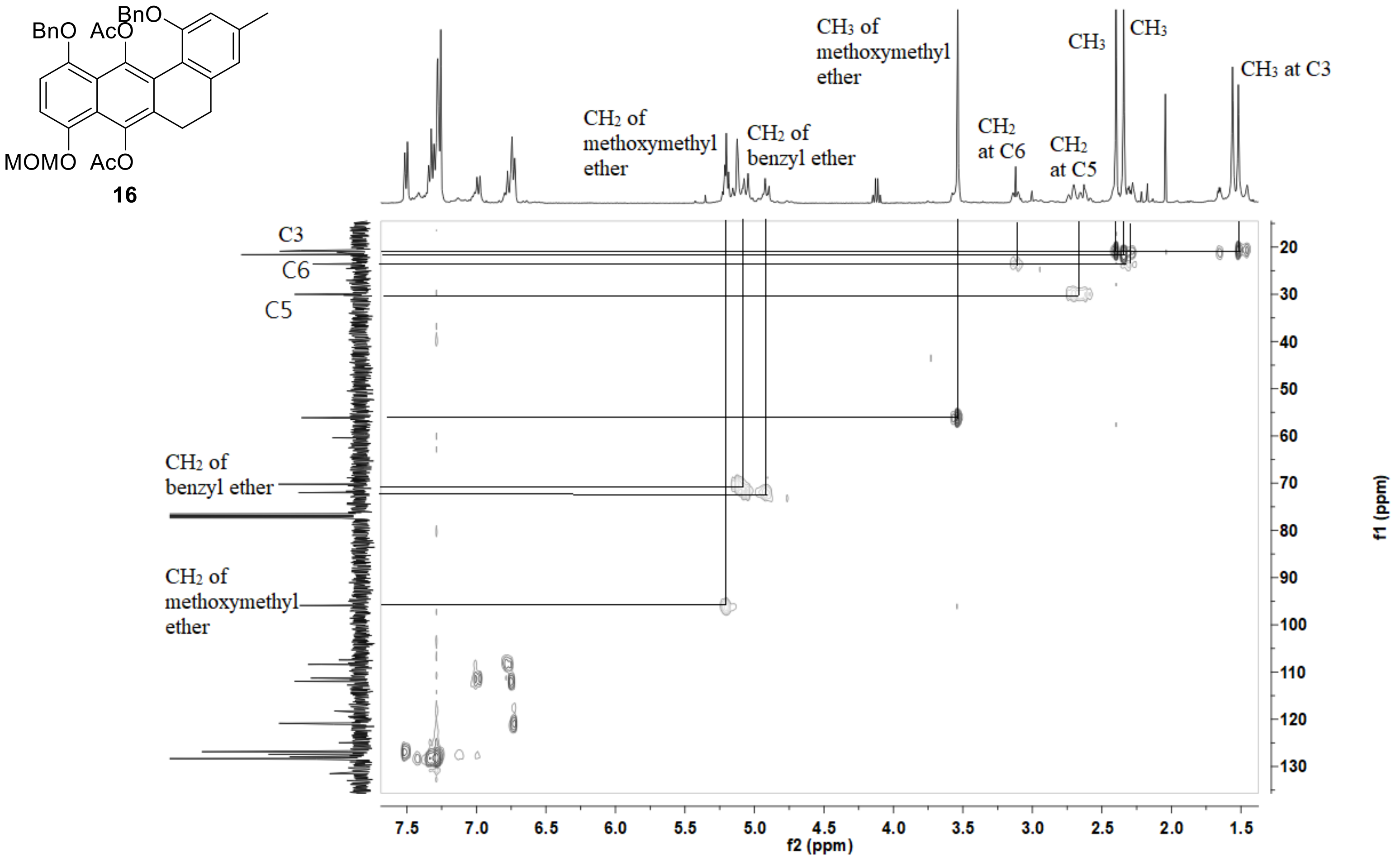

^{1}H NMR spectrum of 1,11-Bis(benzyloxy)-8-(methoxymethoxy)-3-methyl-5,6-dihydrotetraphene-7,12-dione **16A**

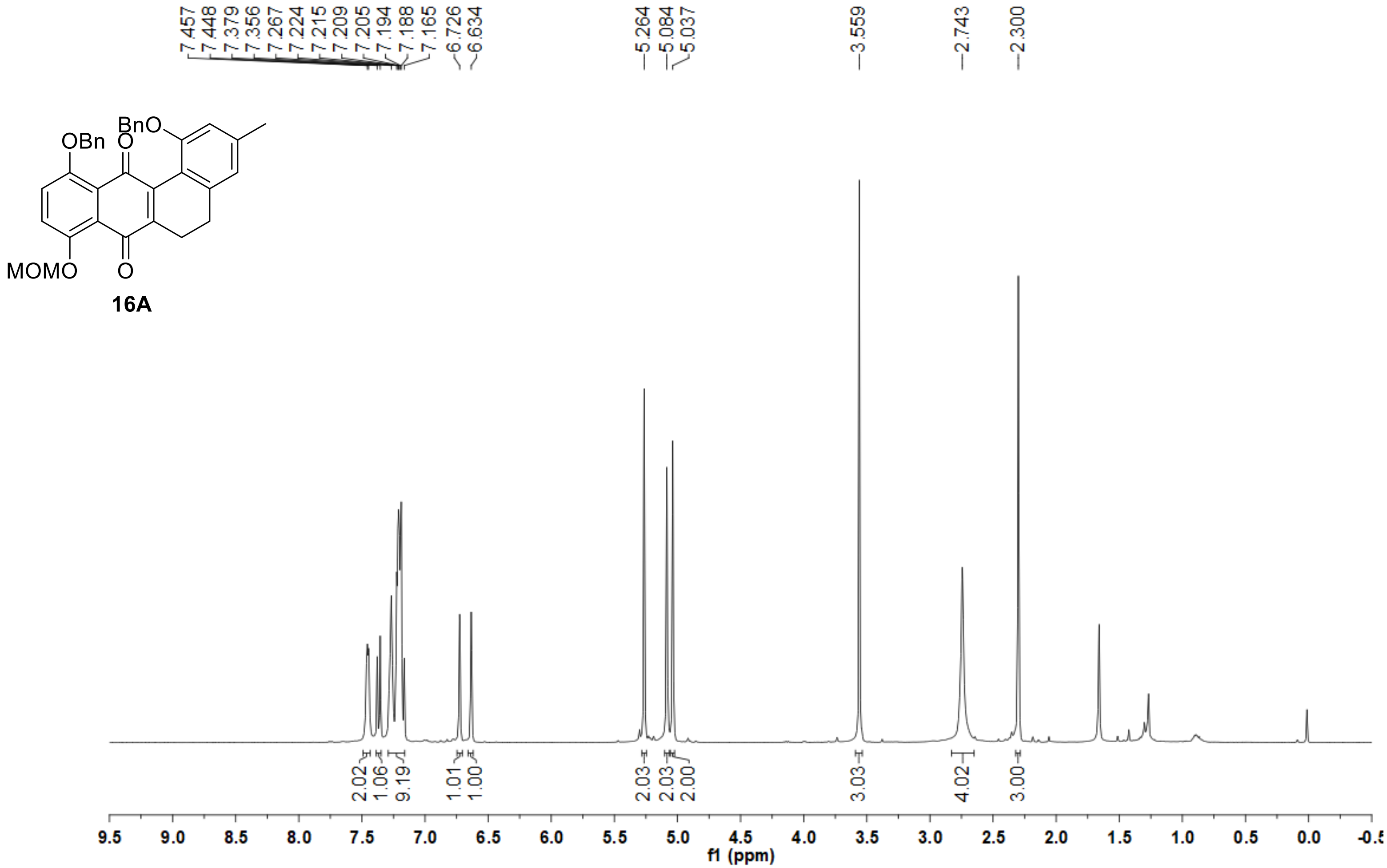

^{13}C NMR spectrum of 1,11-Bis(benzyloxy)-8-(methoxymethoxy)-3-methyl-5,6-dihydrotetraphene-7,12-dione **16A**

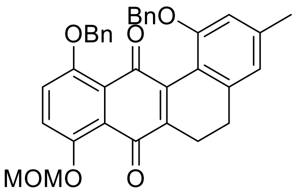

16A

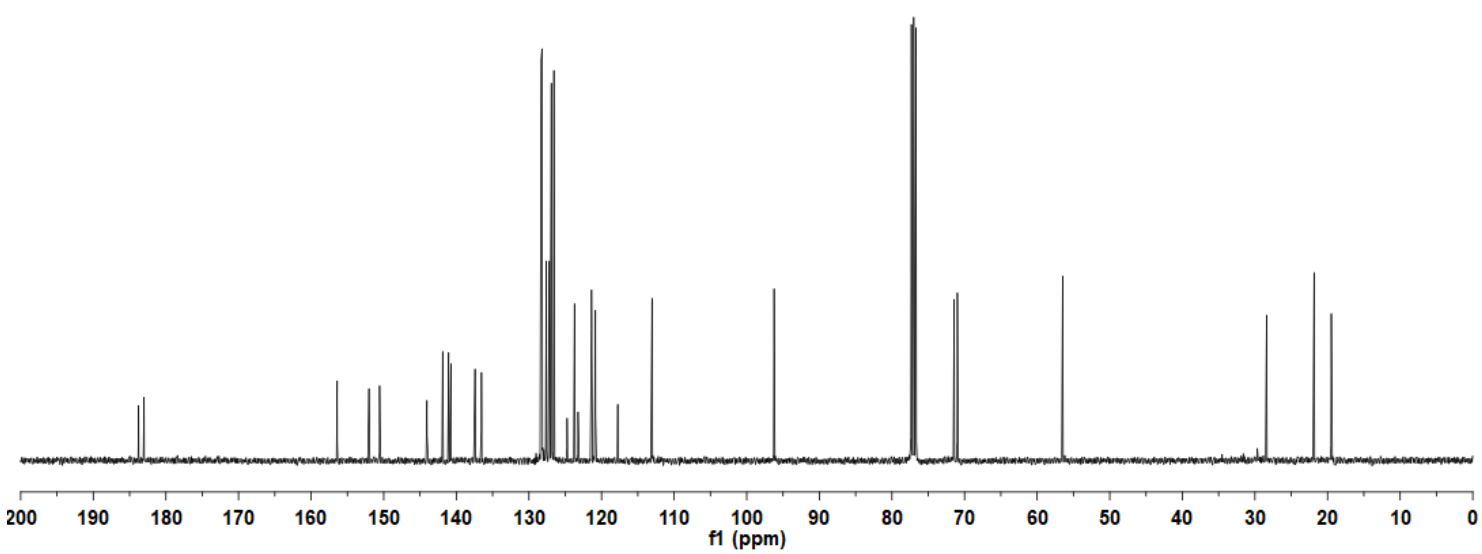

HSQC NMR spectrum of 1,11-Bis(benzyloxy)-8-(methoxymethoxy)-3-methyl-5,6-dihydrotetraphene-7,12-dione **16A**

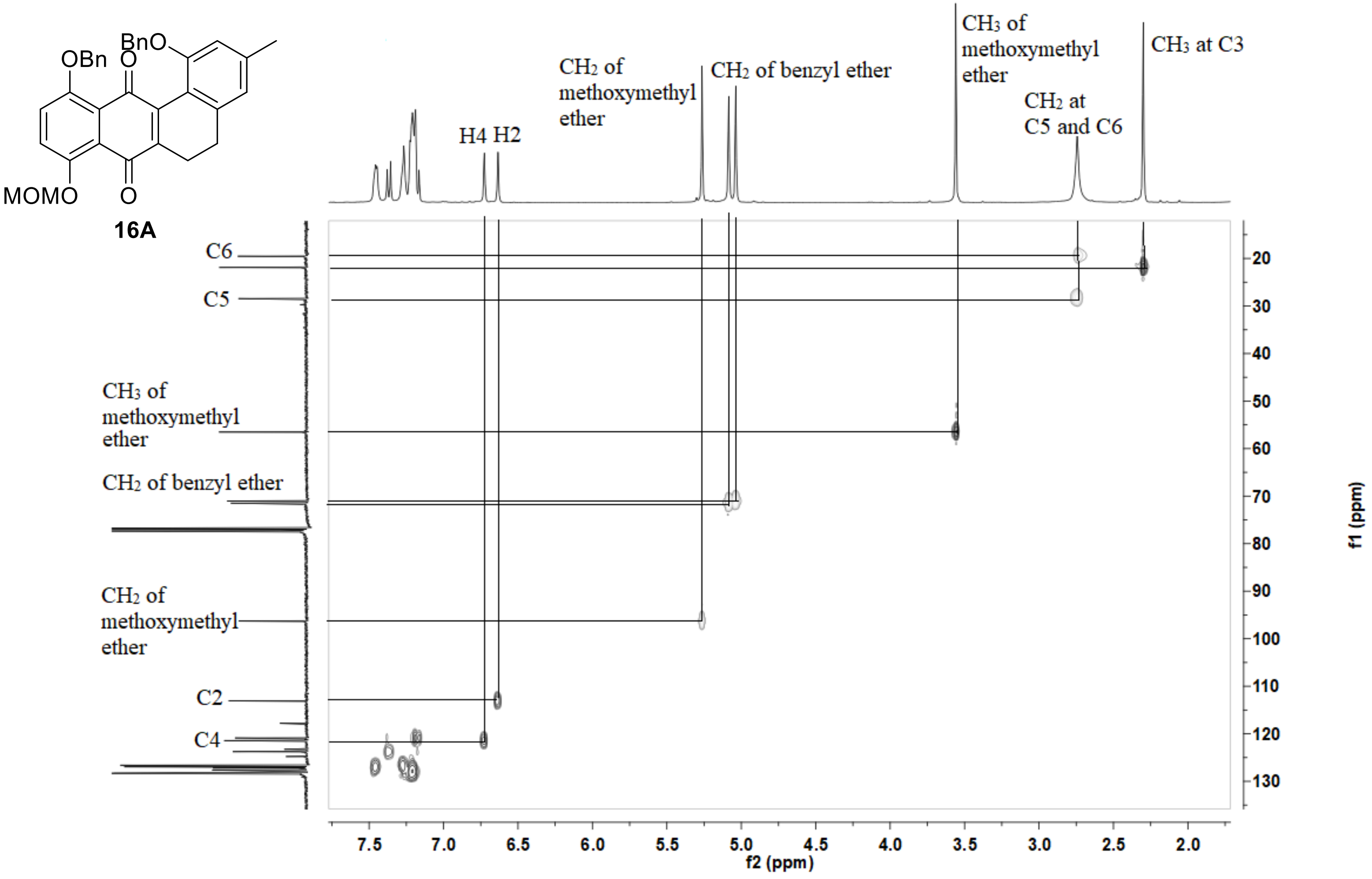

^{1}H NMR spectrum of 1,11-Bis(benzyloxy)-8-hydroxy-3-methyl-5,6-dihydrotetraphene-7,12-dione **17**

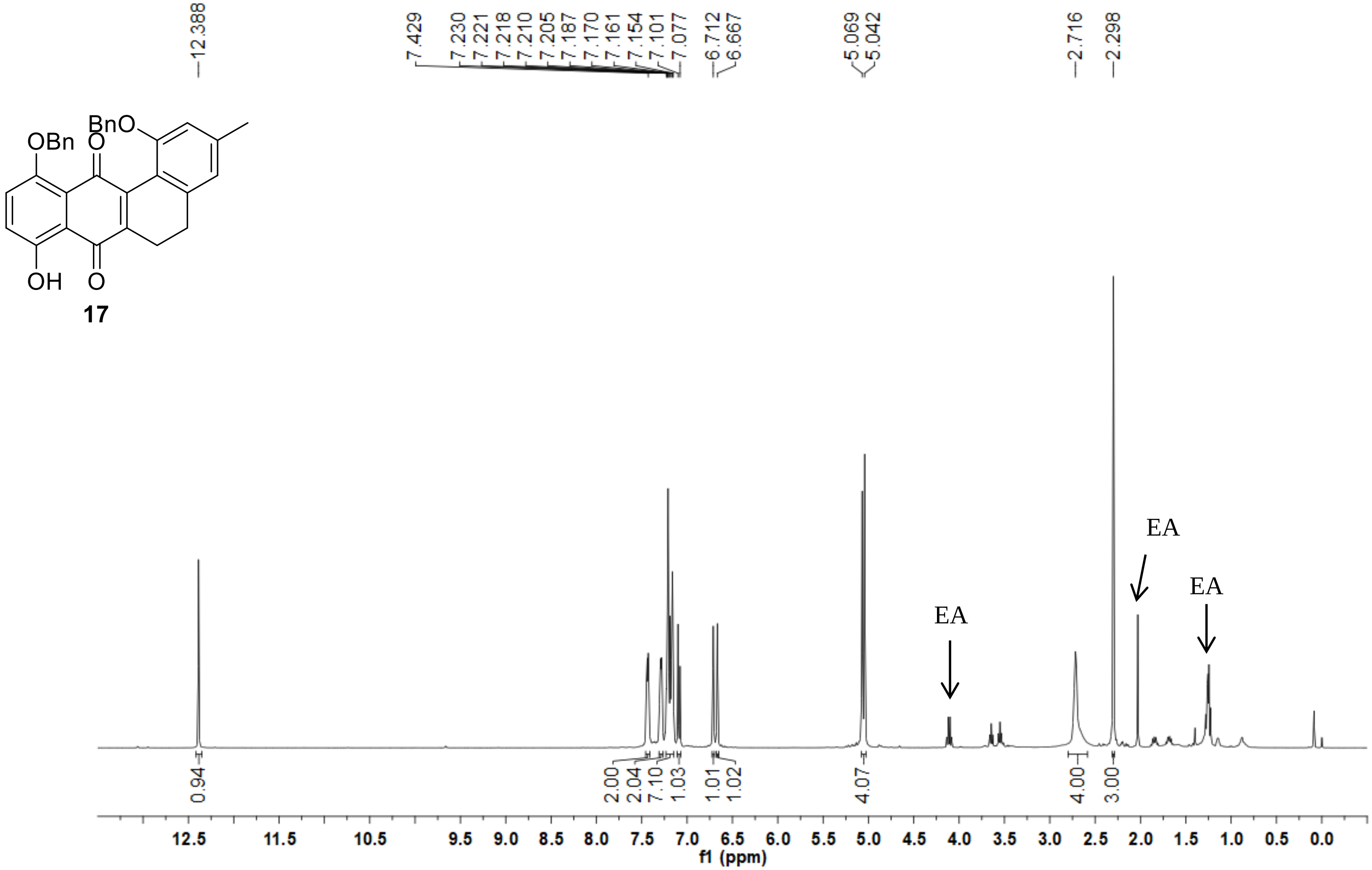

^{13}C NMR spectrum of 1,11-Bis(benzyloxy)-8-hydroxy-3-methyl-5,6-dihydrotetraphene-7,12-dione **17**

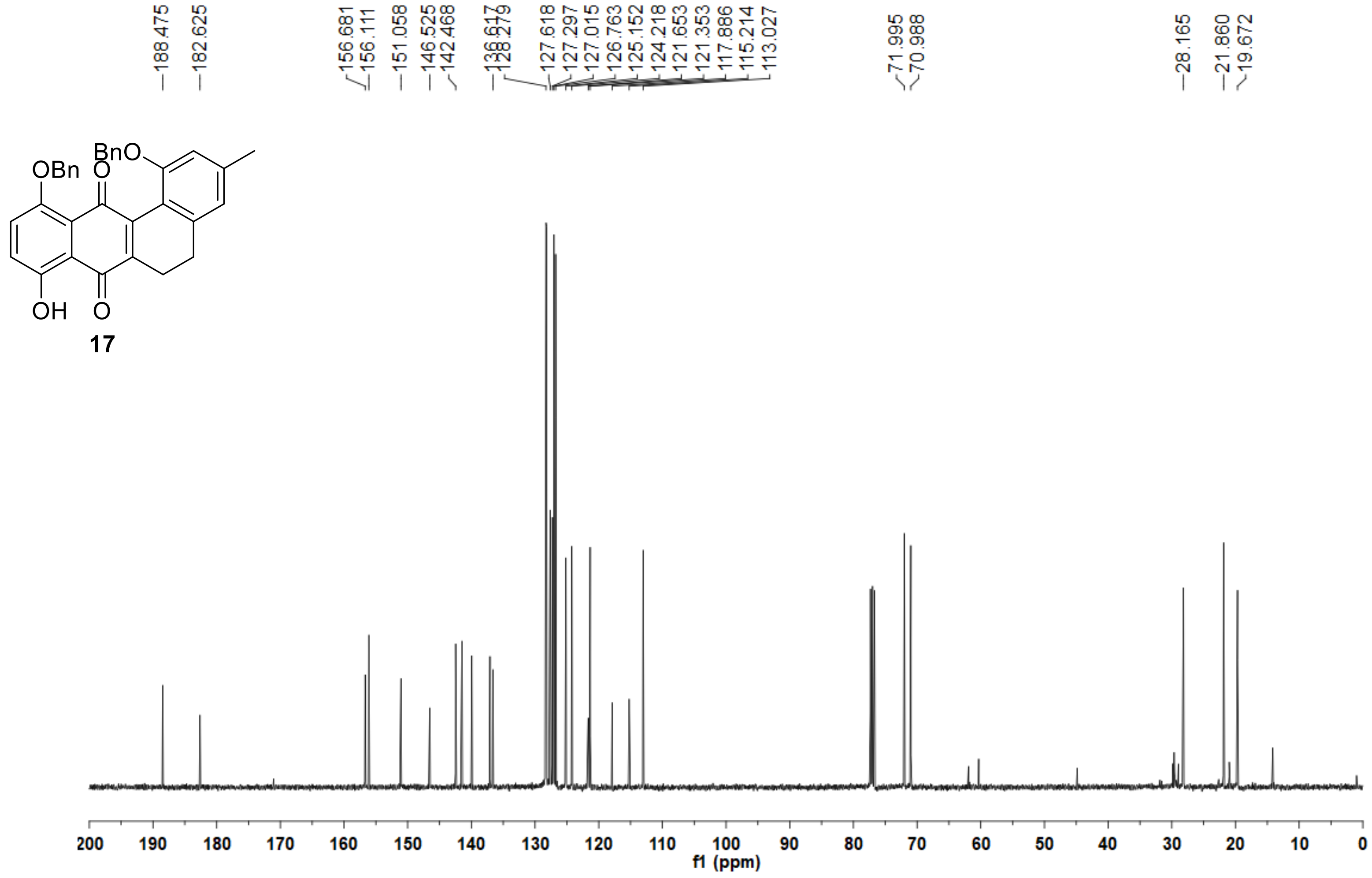

HSQC NMR spectrum of 1,11-Bis(benzyloxy)-8-hydroxy-3-methyl-5,6-dihydrotetraphene-7,12-dione **17**

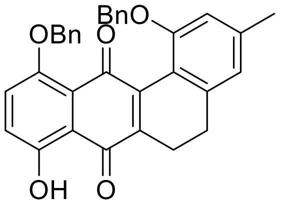

17

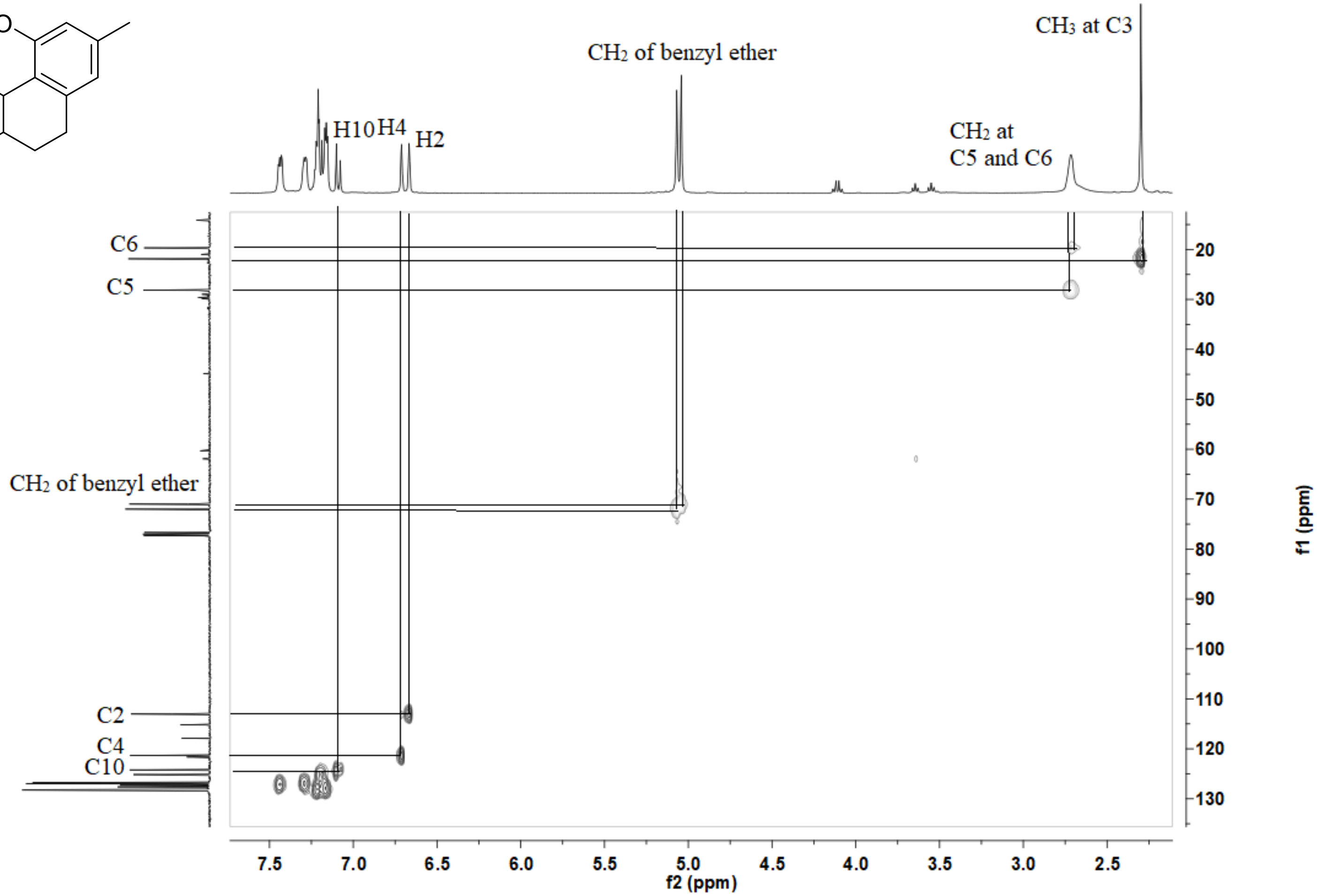

^{1}H NMR spectrum of anhydrolandomycinone (**1**)

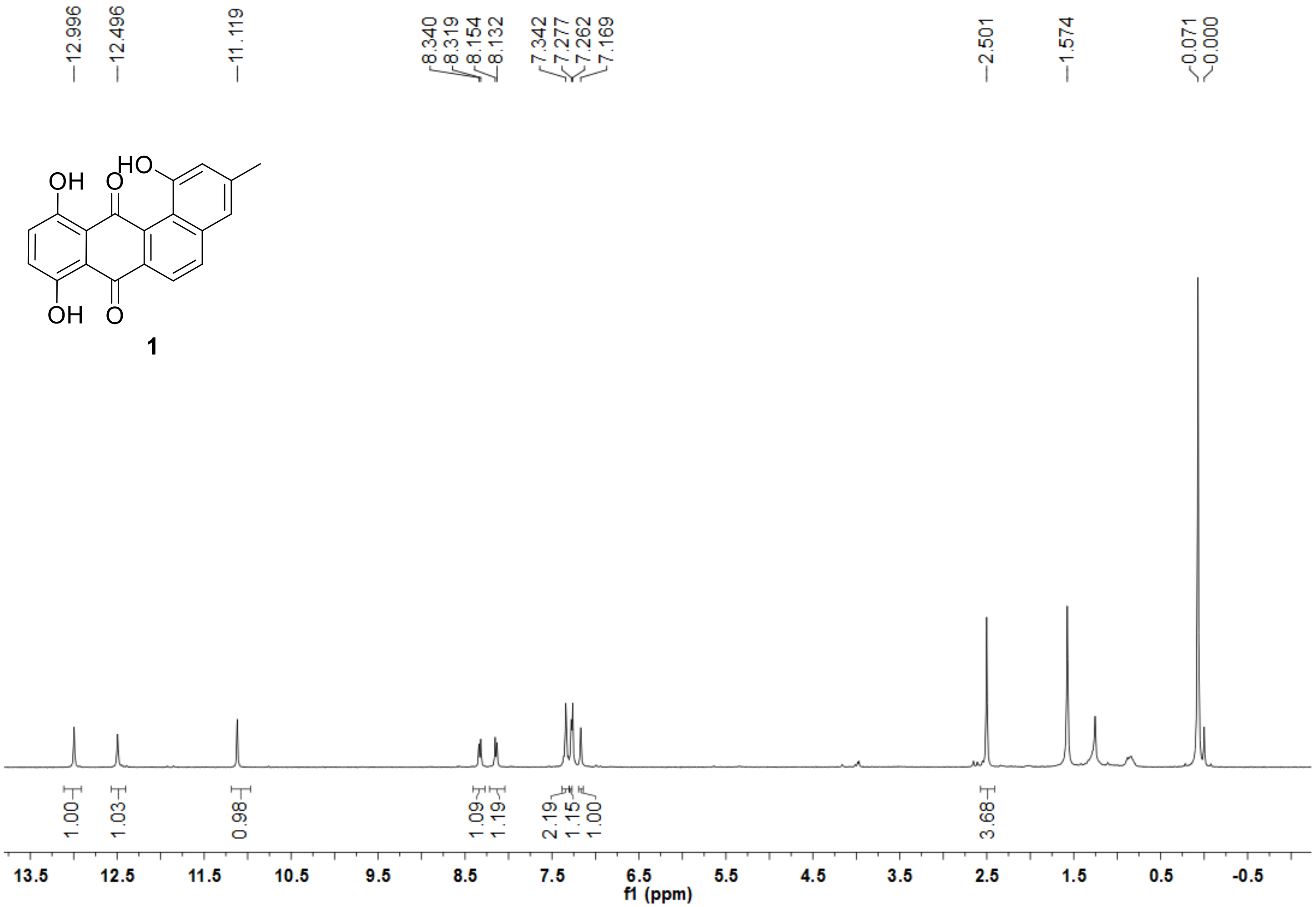

^{13}C NMR spectrum of anhydrolandomycinone (**1**)

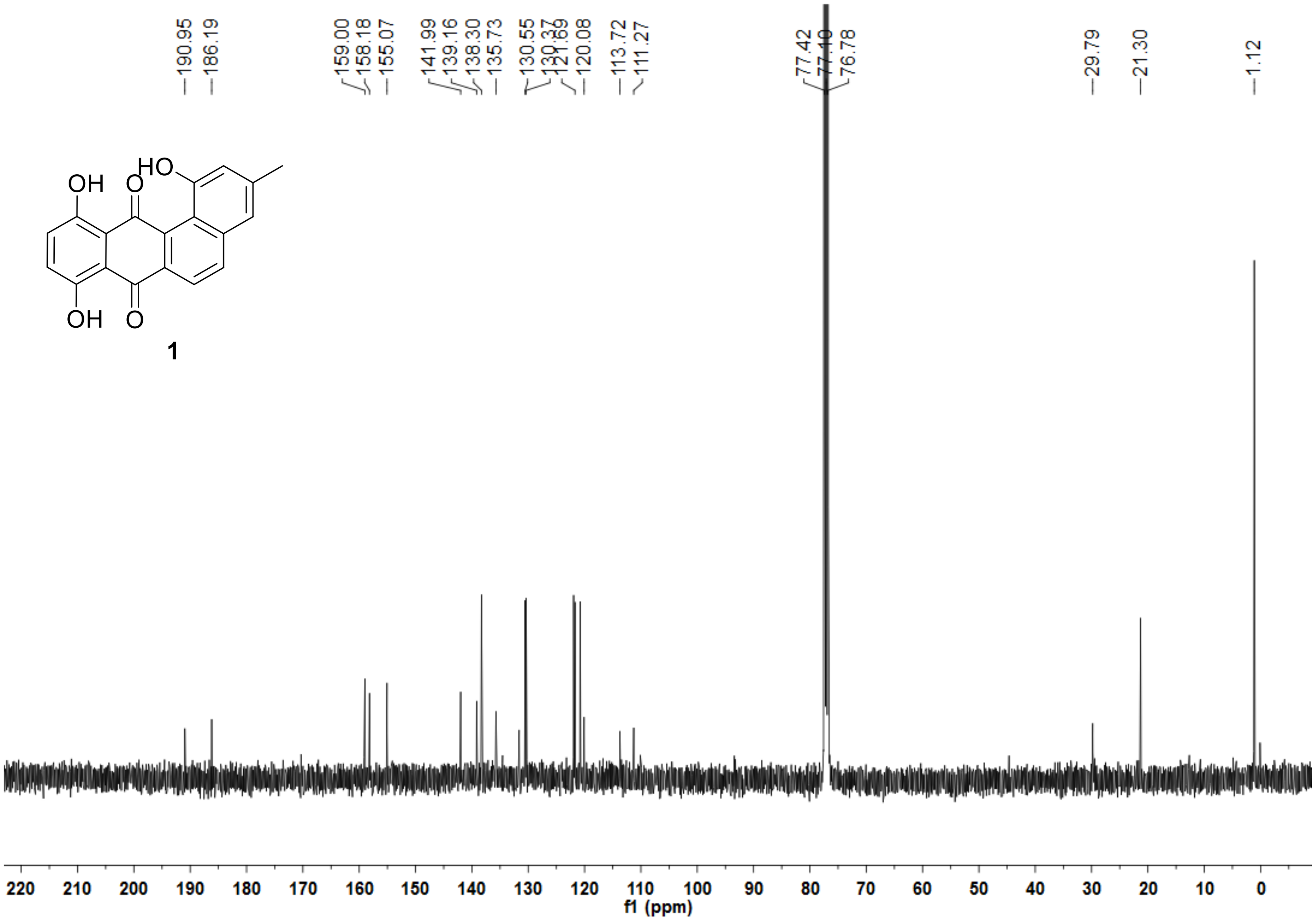

^{1}H NMR spectrum of B-ring unsaturated core analogue (**2**)

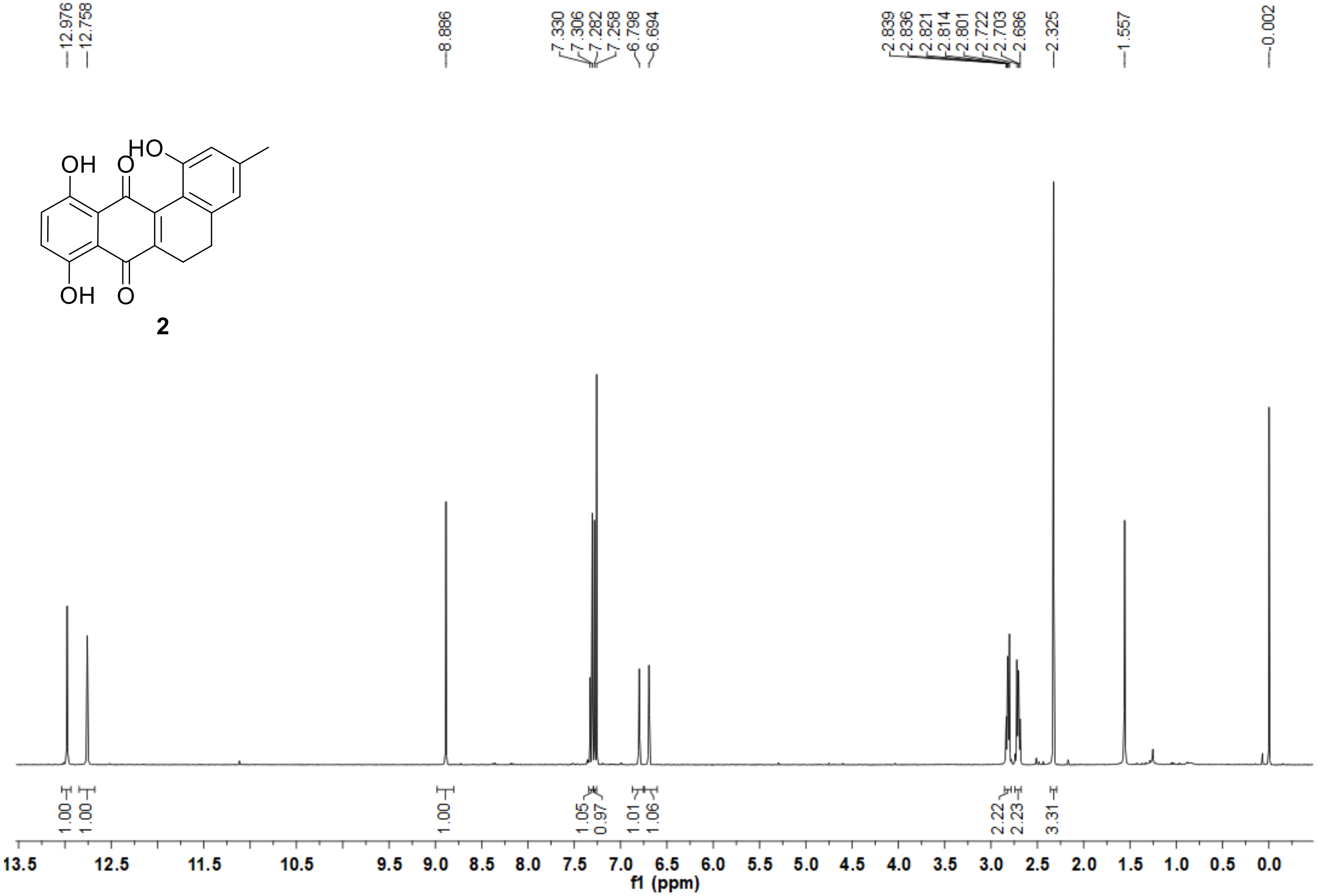

^{13}C NMR spectrum of B-ring unsaturated core analogue (**2**)

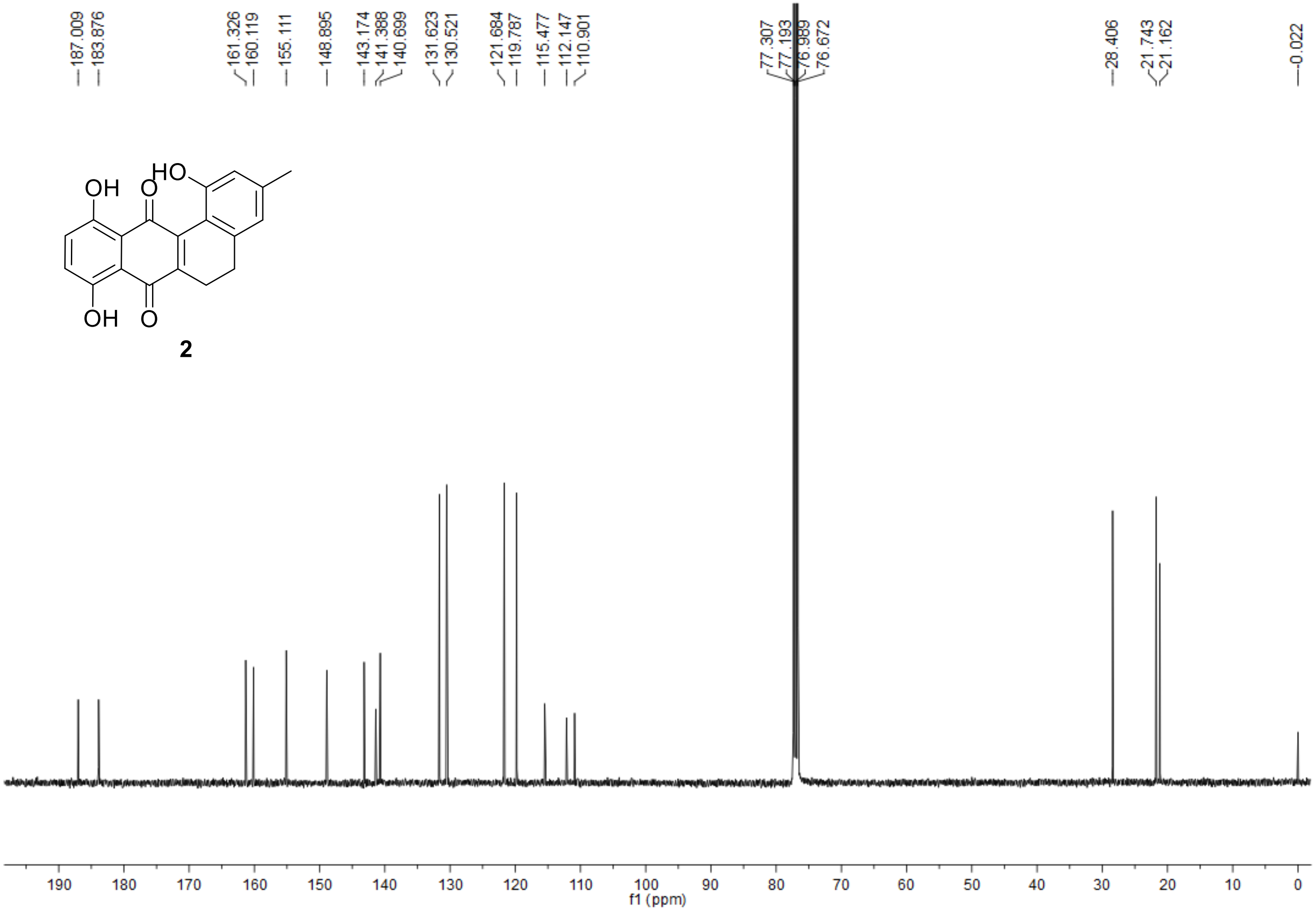

^{1}H NMR spectrum of 1,11-Bis(benzyloxy)-8-hydroxy-3-methyltetraphene-7,12-dione **18**

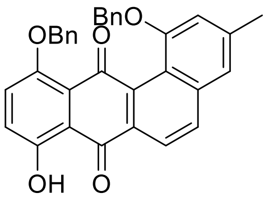

18

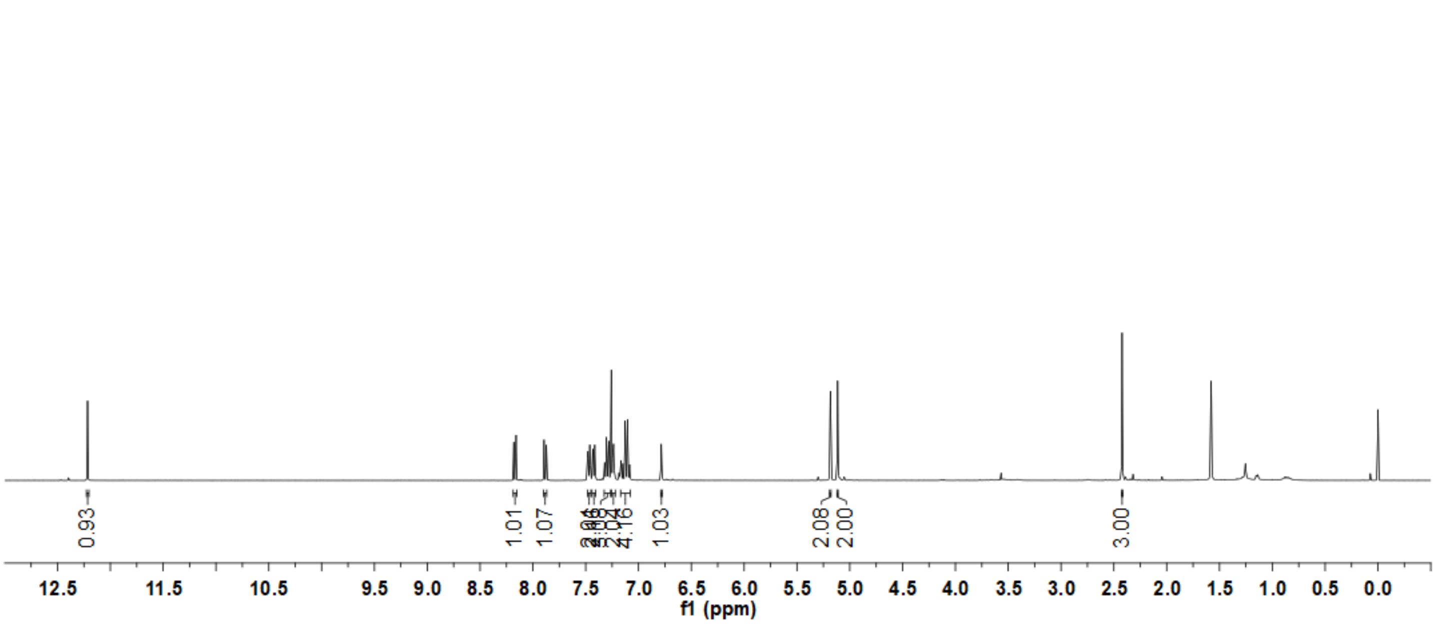

^{13}C NMR spectrum of 1,11-Bis(benzyloxy)-8-hydroxy-3-methyltetraphene-7,12-dione **18**

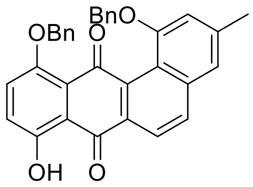

18

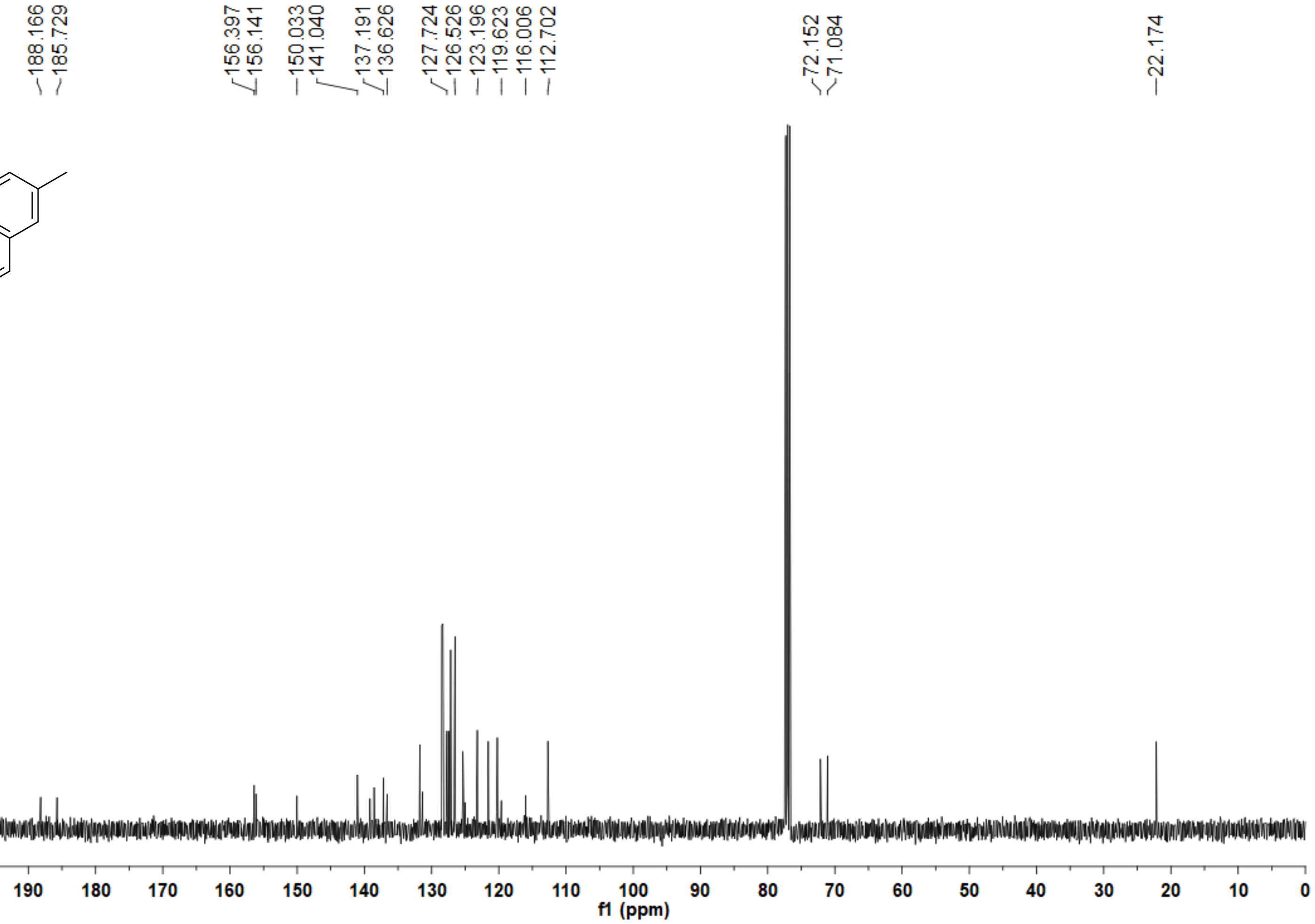

^{1}H NMR spectrum of 3,4-Di-*O*-acetyl-1,5-anhydro-2,6-dideoxy-L-*arabino*-hex-1-enol **36A**

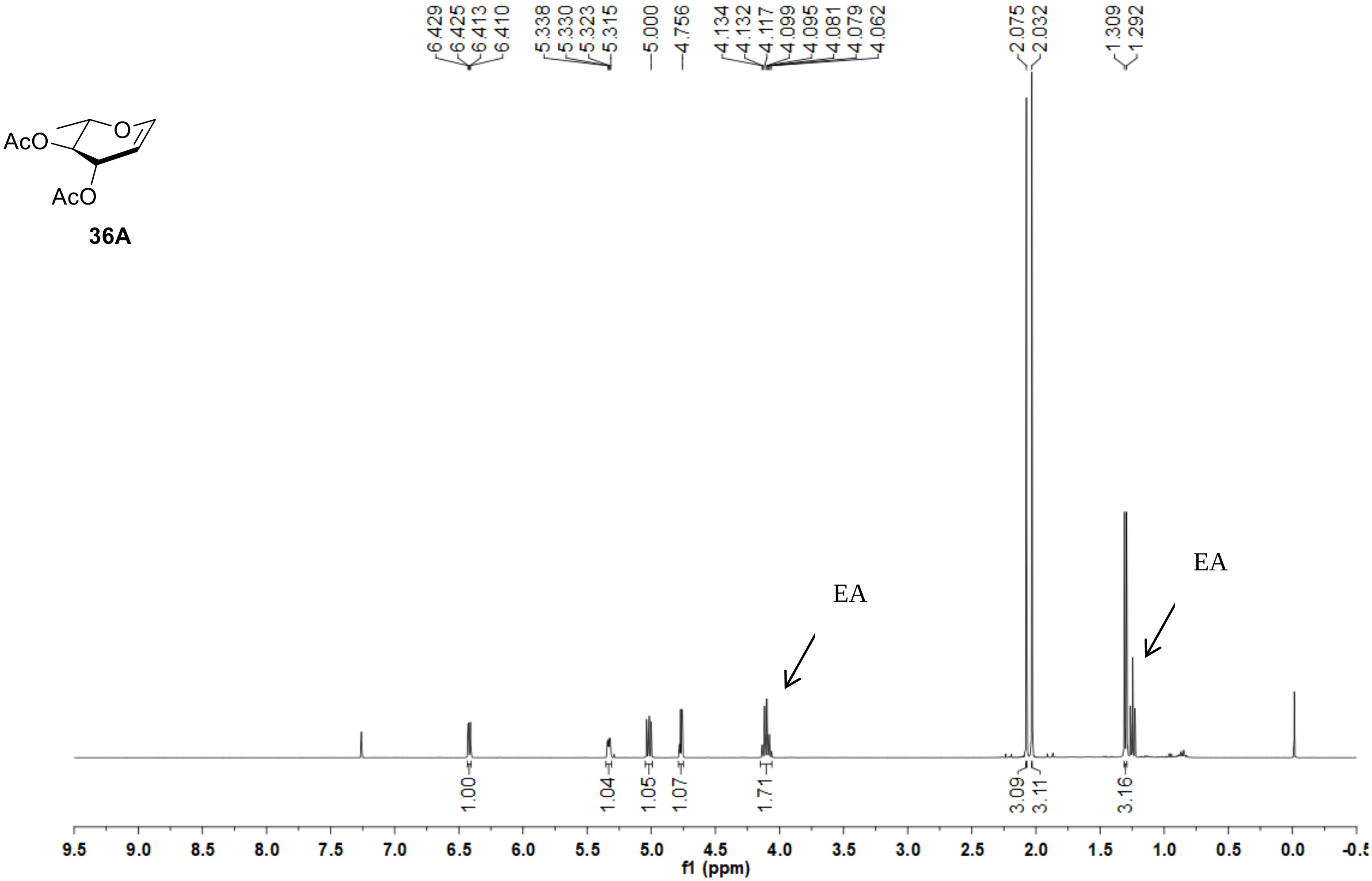

36A

^{1}H NMR spectrum of Methyl 4-*O*-Acetyl-2,3,6-trideoxy-L-*erythro*-hex-2-enopyranoside **36B**

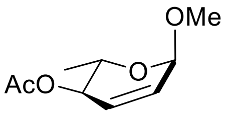

36B

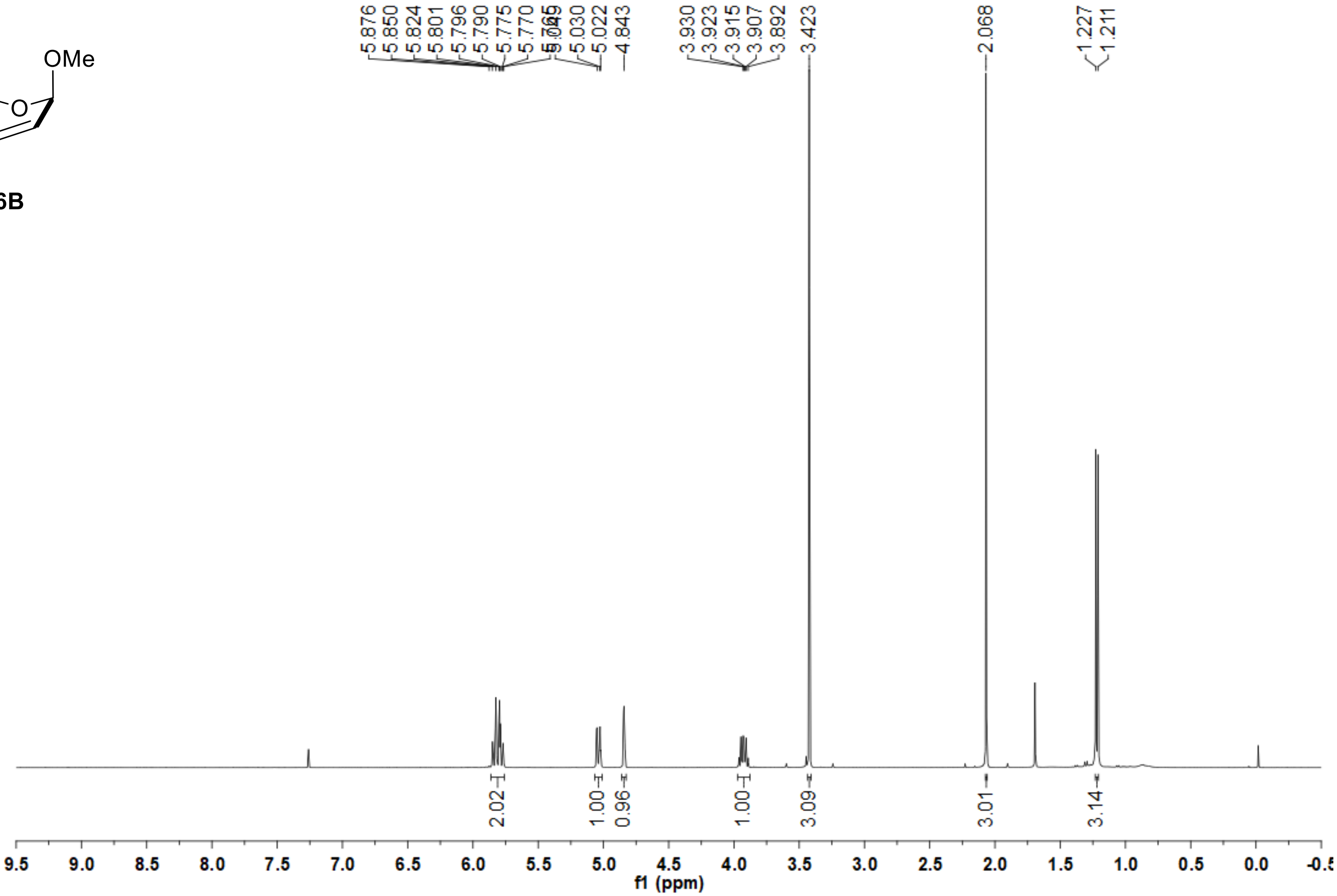

^{1}H NMR spectrum of Methyl 4-*O*-Benzoyl-2,3,6-trideoxy-L-*erythro*-hex-2-enopyranoside **36C**

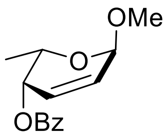

36C

(contain some β-anomer)

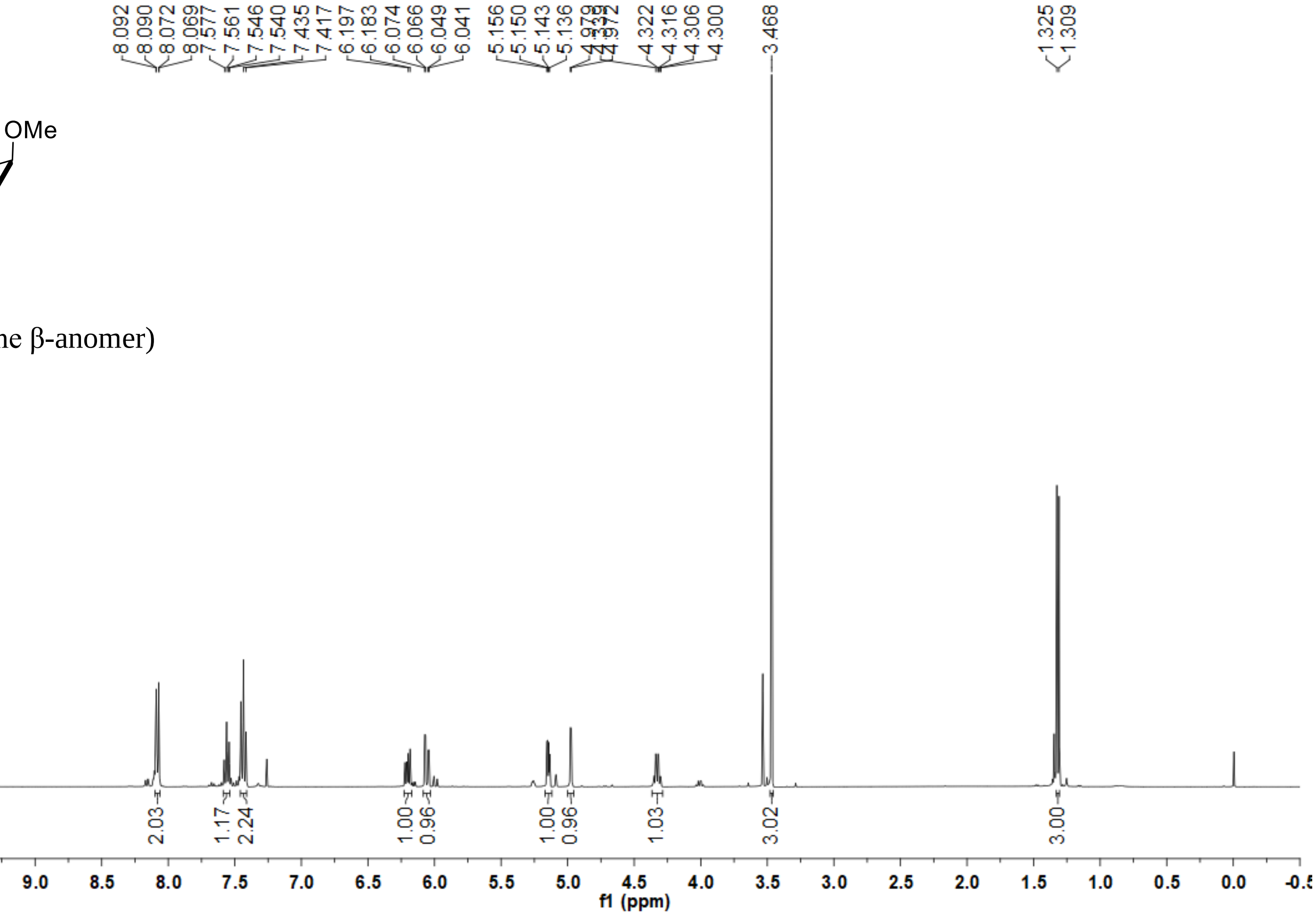

^{1}H NMR spectrum of Methyl 4-*O*-Benzoyl-2,3,6-trideoxy-α-L-*threo*-hexopyranoside **36D**

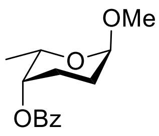

36D

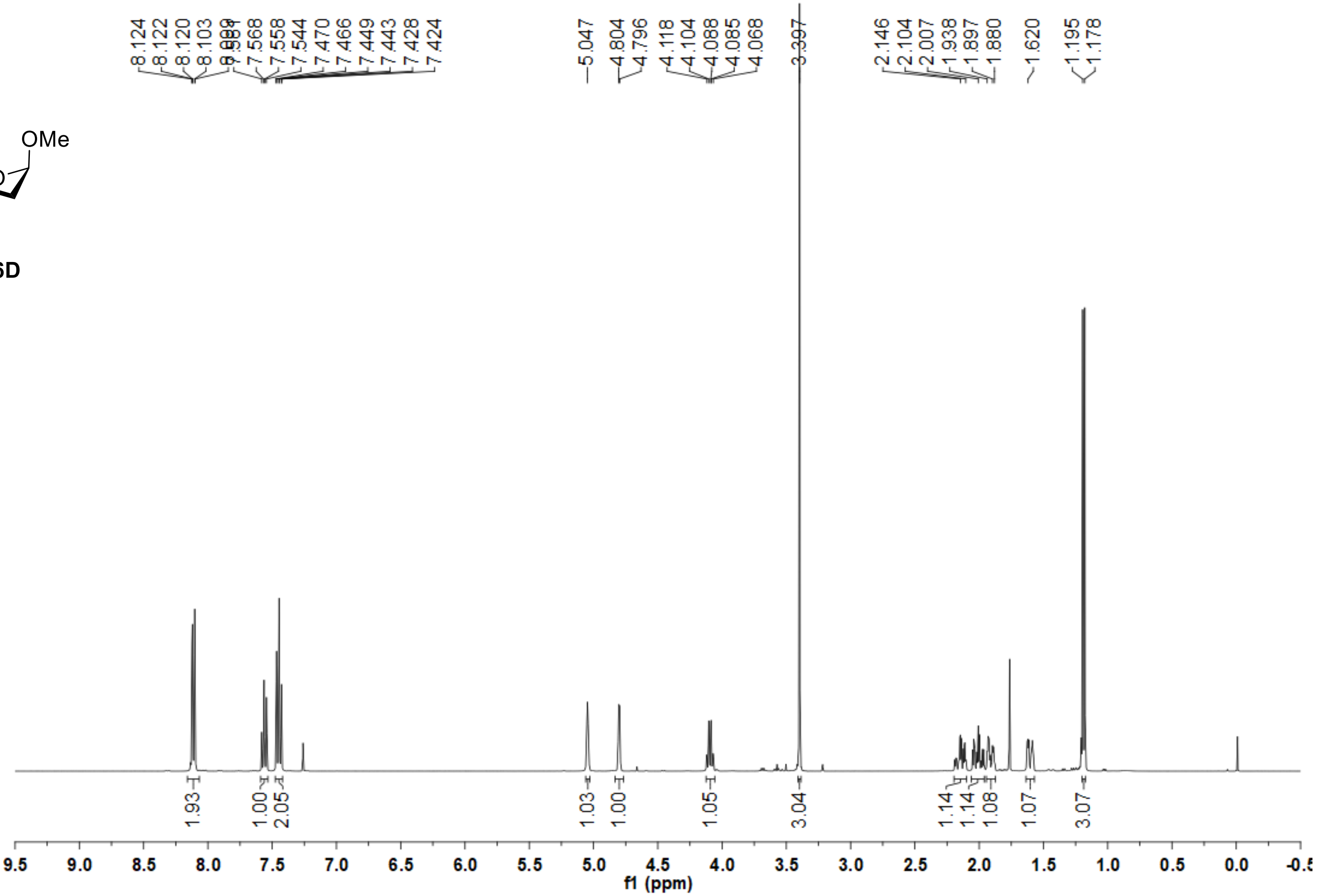

^{1}H NMR spectrum of 1-*O*-acetyl-4-*O*-benzoyl-2,3,6-trideoxy-α-L-rhodinose **19**

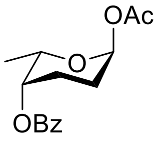

19

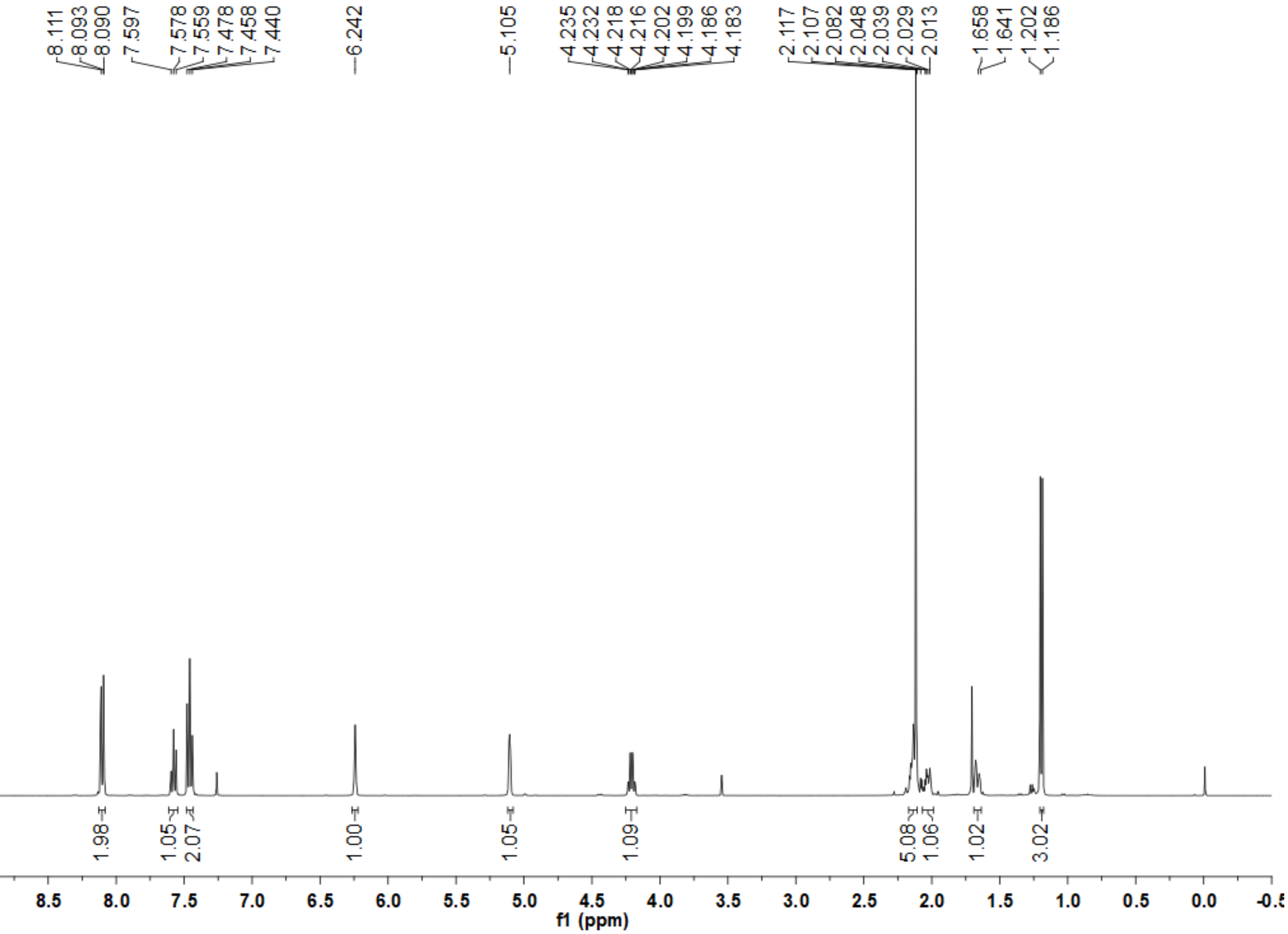

^{1}H NMR spectrum of 3-*O*-*tert*-Butyldimethylsilyl-6-iodo-D-glucal **23B**

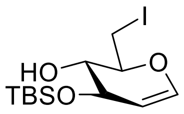

23B

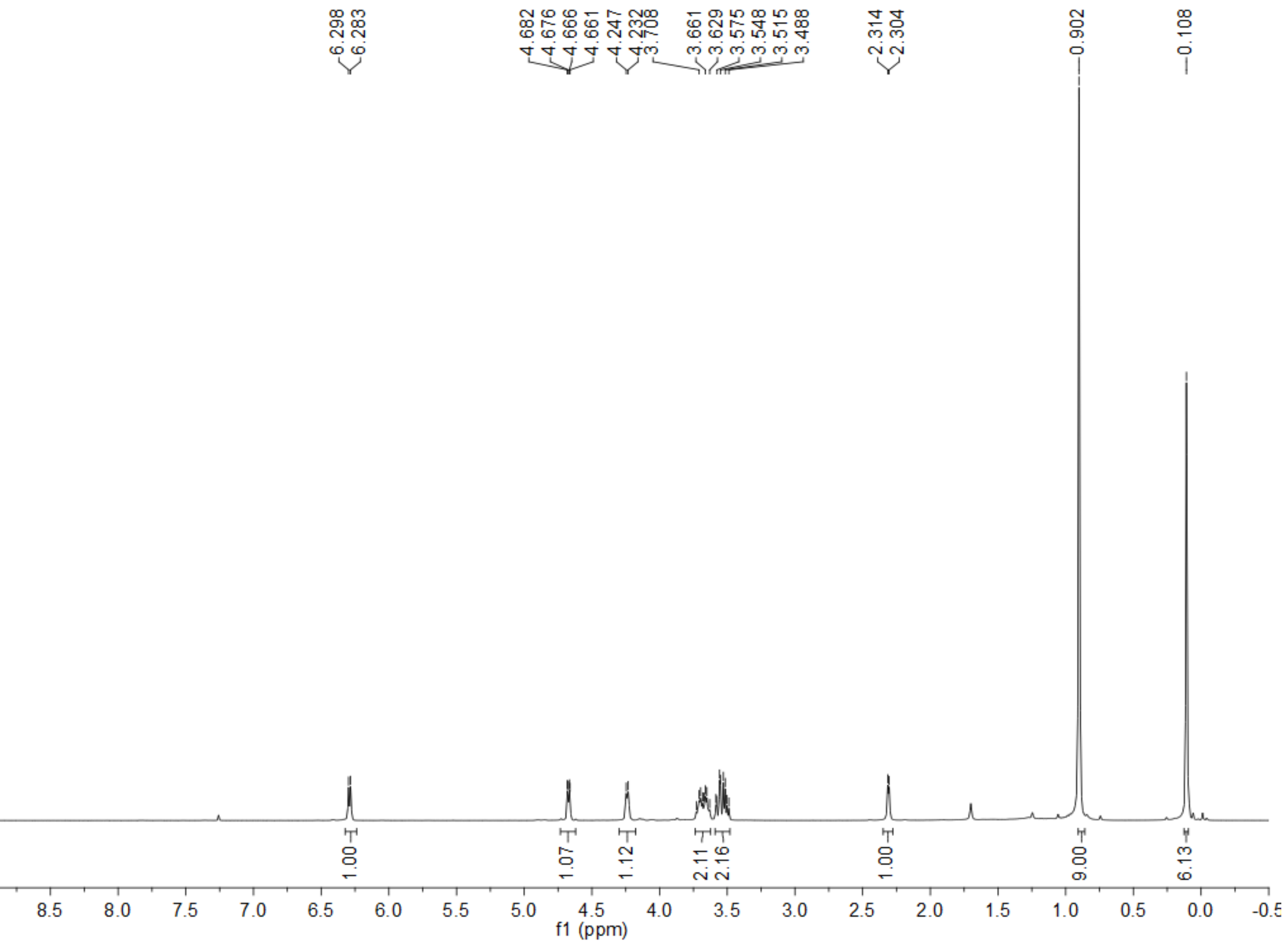

^{13}C NMR spectrum of 3-*O*-*tert*-Butyldimethylsilyl-6-iodo-D-glucal **23B**

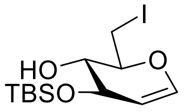

23B

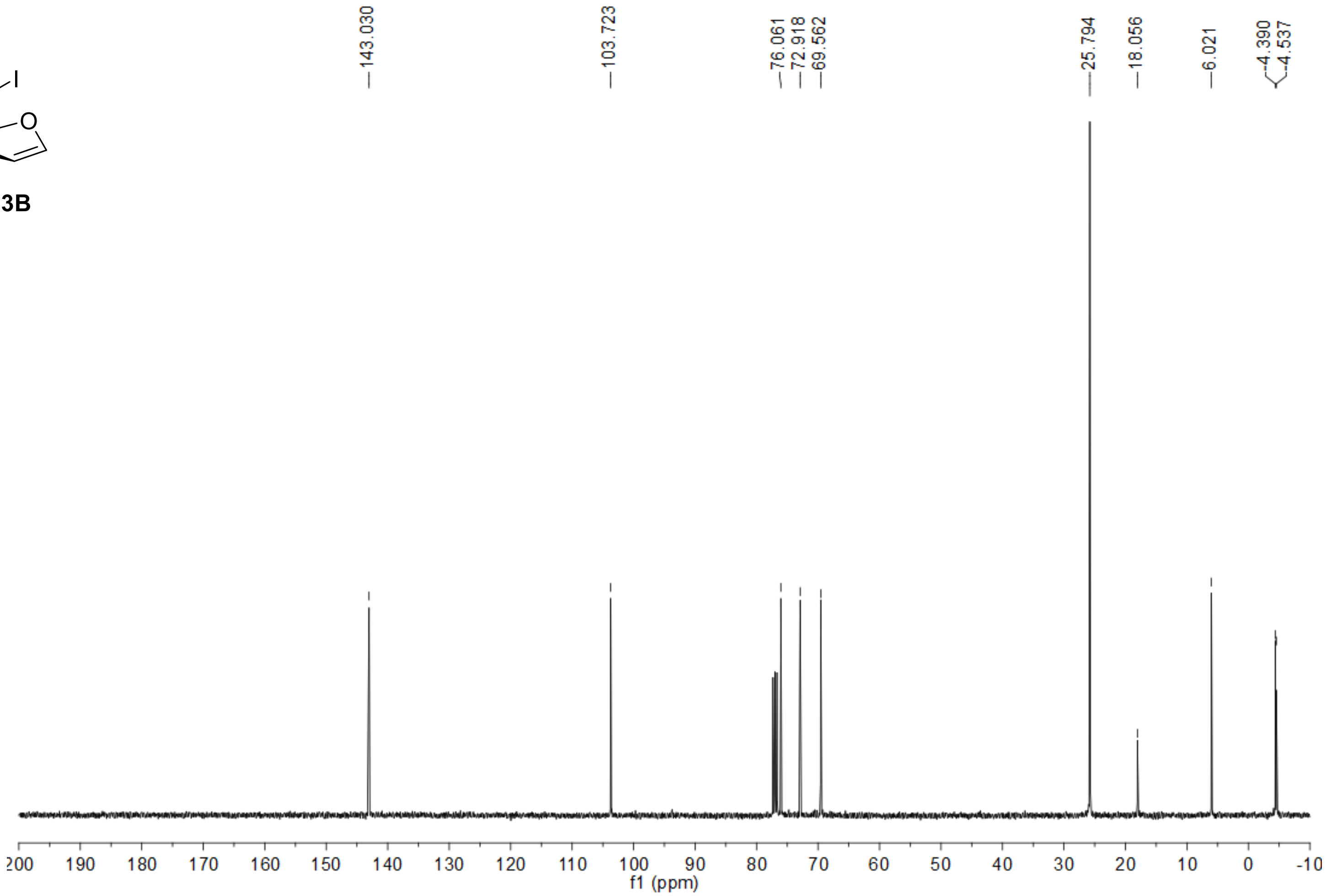

^{1}H NMR spectrum of 4-*O*-Acetyl-3-*O*-*tert*-butyldimethylsilyl-6-deoxy-D-glucal **23C**

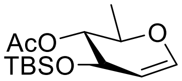

23C

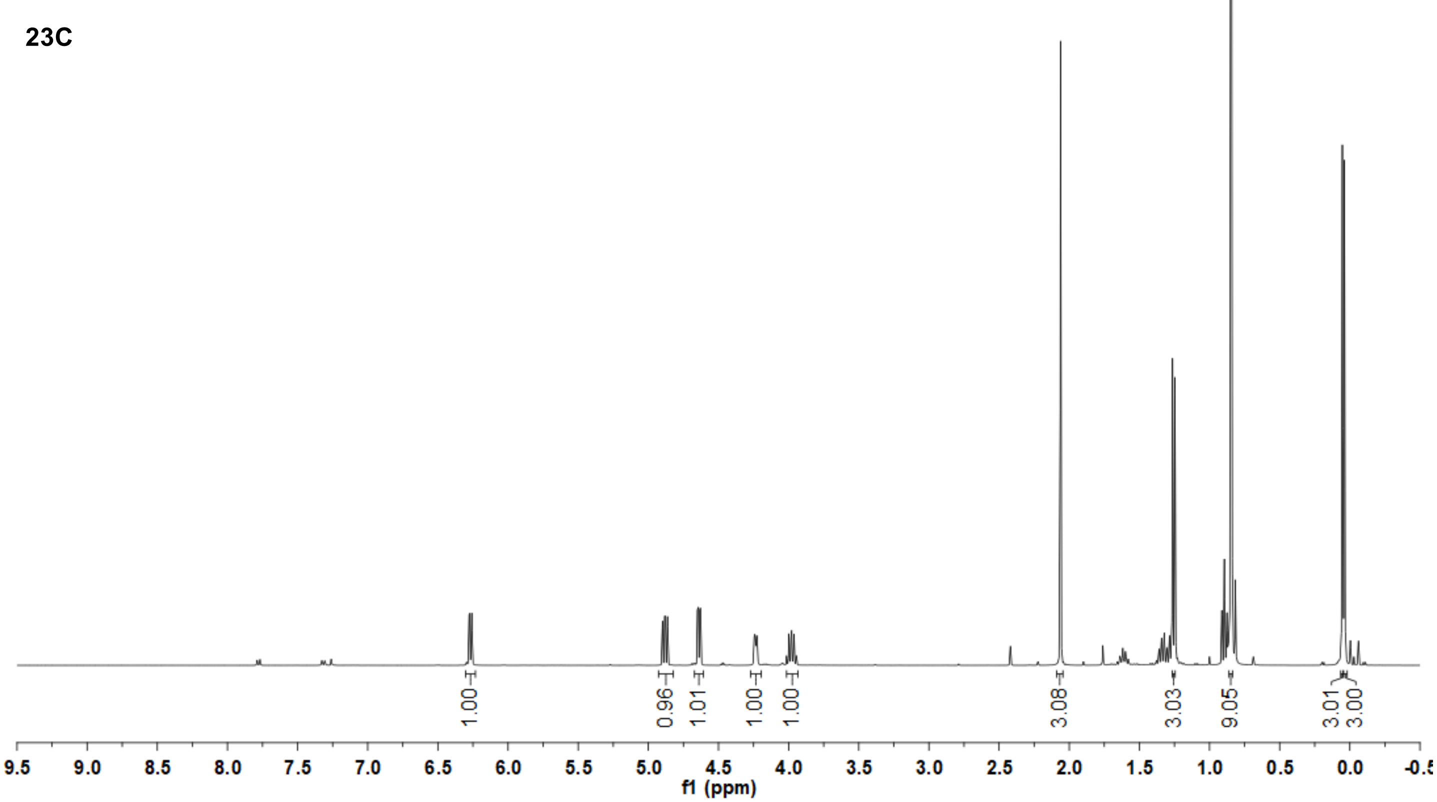

^{13}C NMR spectrum of 4-*O*-Acetyl-3-*O*-*tert*-butyldimethylsilyl-6-deoxy-D-glucal **23C**

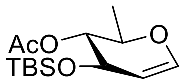

23C

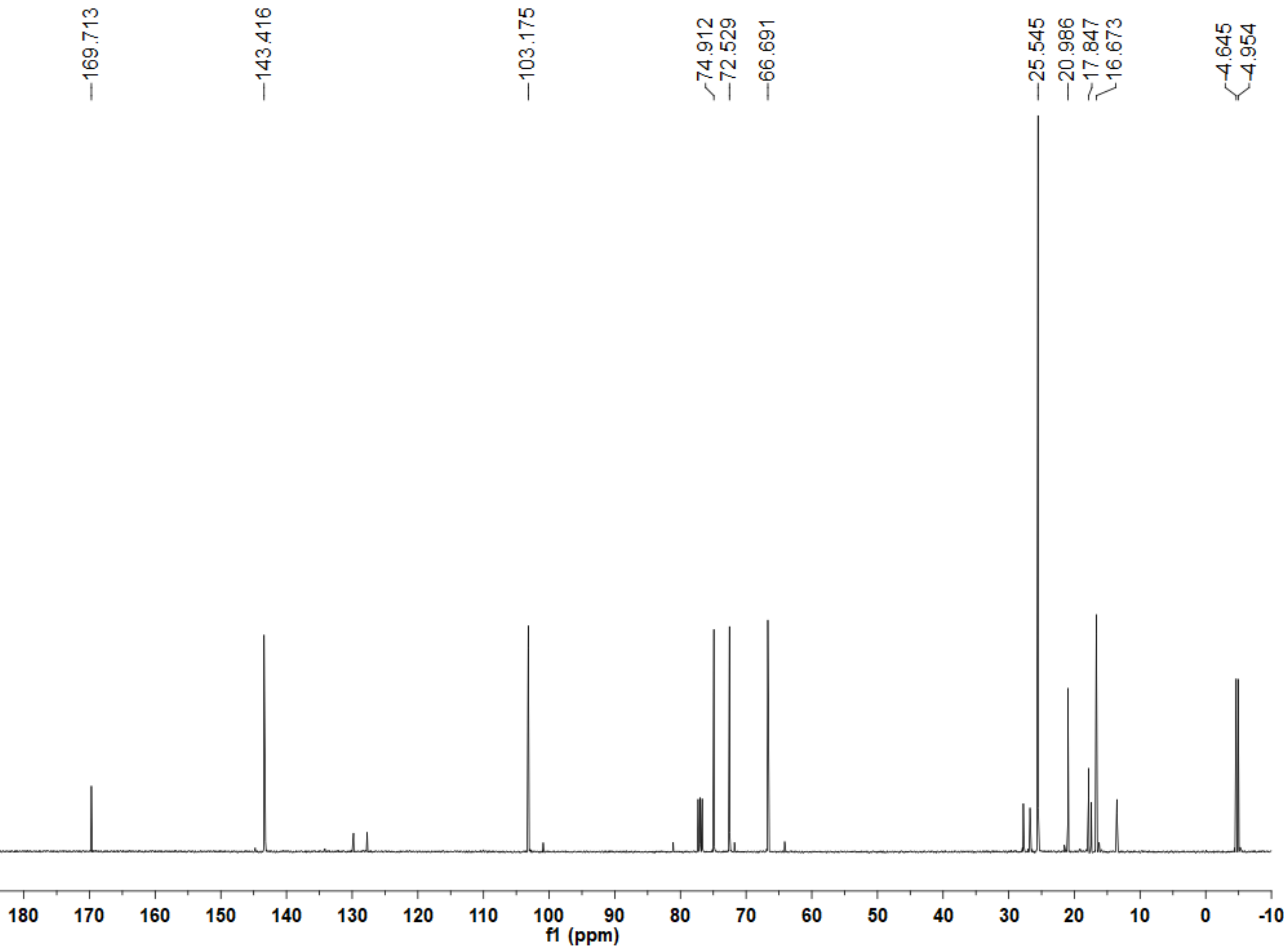

COSY NMR spectrum of 4-*O*-Acetyl-3-*O*-*tert*-butyldimethylsilyl-6-deoxy-D-glucal **23C**

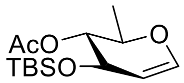

23C

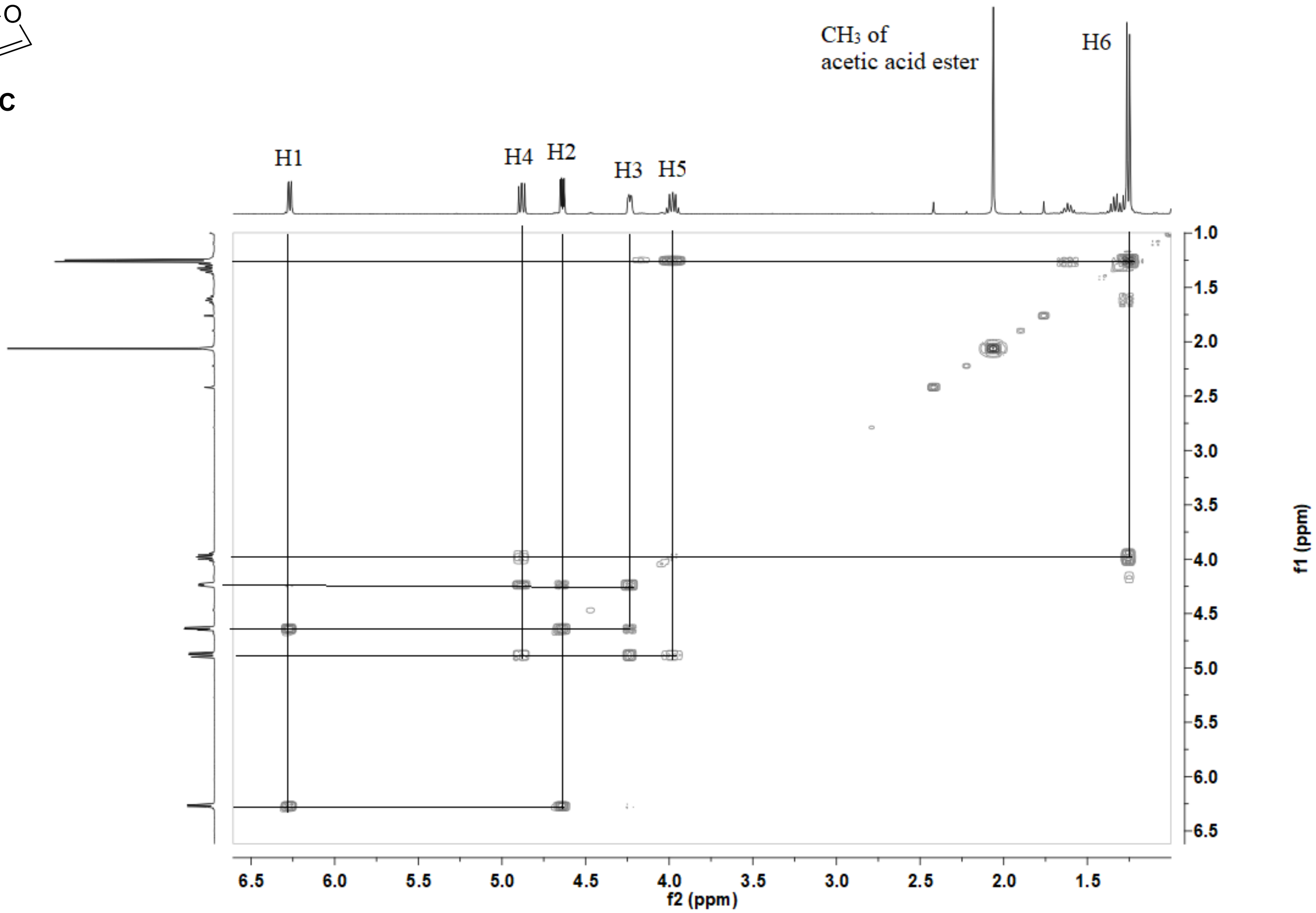

HSQC NMR spectrum of 4-*O*-Acetyl-3-*O*-*tert*-butyldimethylsilyl-6-deoxy-D-glucal **23C**

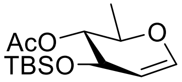

23C

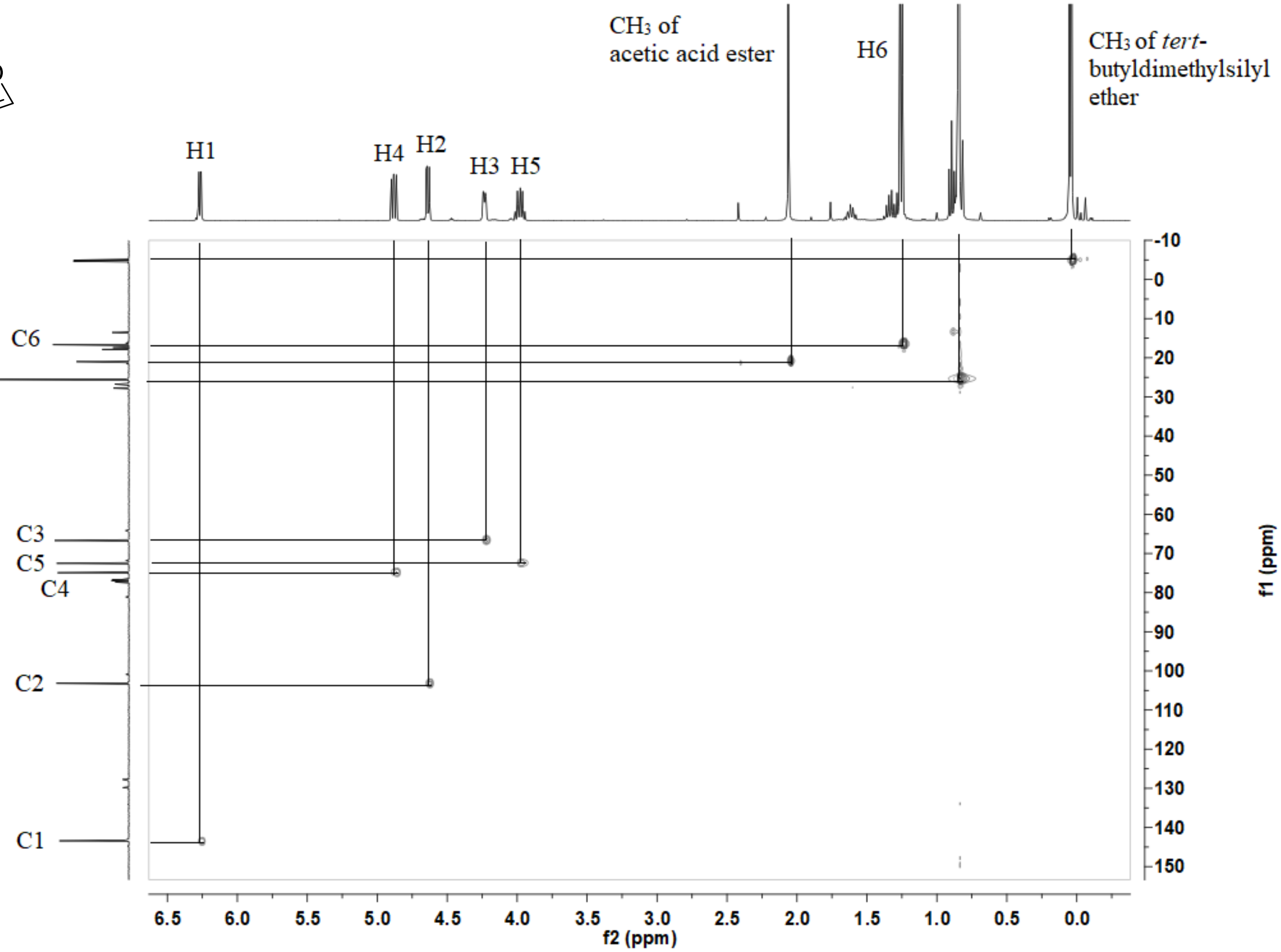

^{1}H NMR spectrum of 1,4-Di-*O*-acetyl--3-*O*-*tert*-butyldimethylsilyl-2,6-dideoxy-2-iodo-*β*-D-glucopyranoside **22**

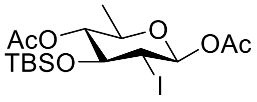

22

(contain some **23D**)

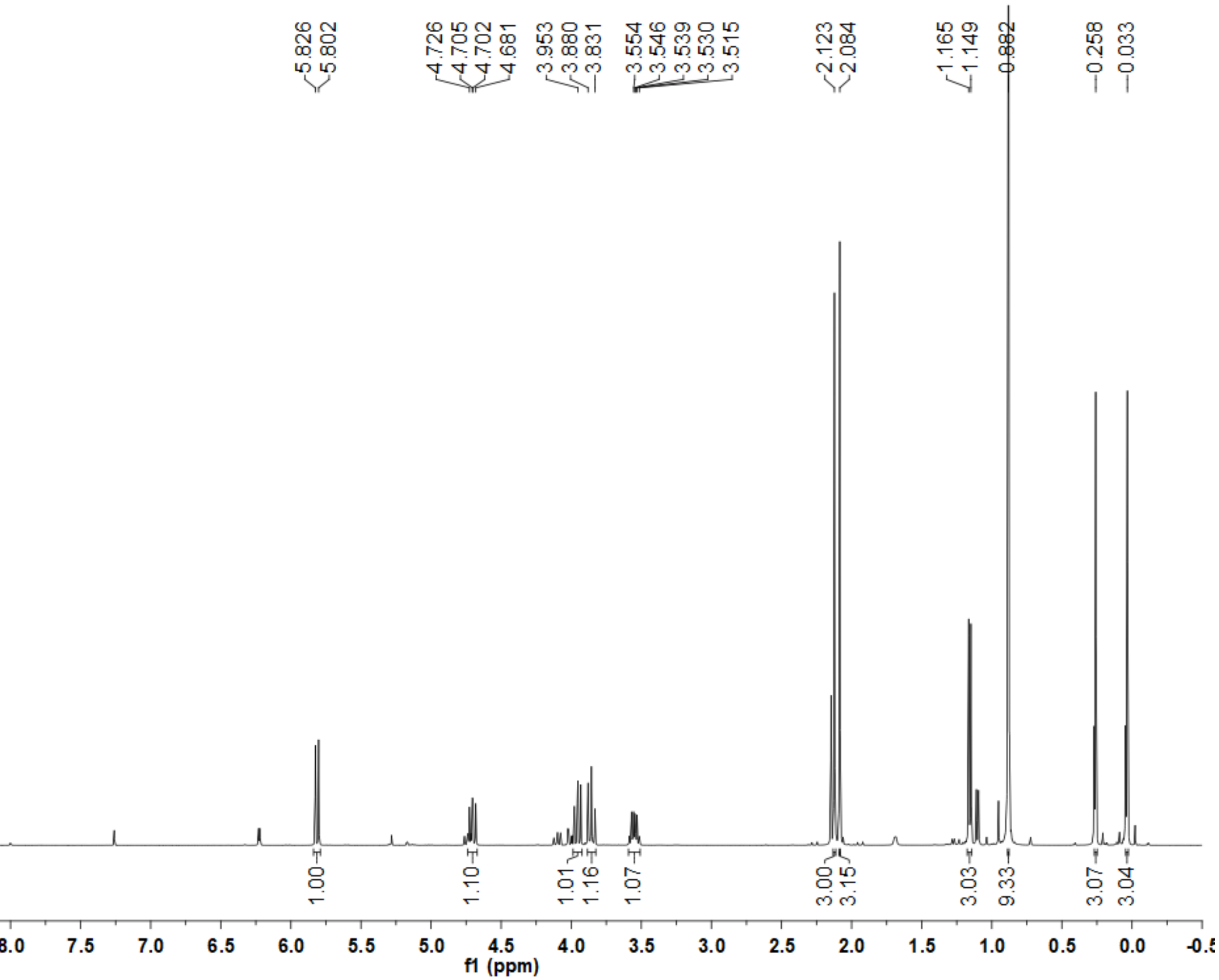

^{13}C NMR spectrum of 1,4-Di-*O*-acetyl--3-*O*-*tert*-butyldimethylsilyl-2,6-dideoxy-2-iodo-*β*-D-glucopyranoside **22**

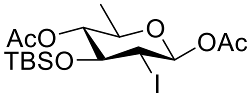

22

(contain some **23D**)

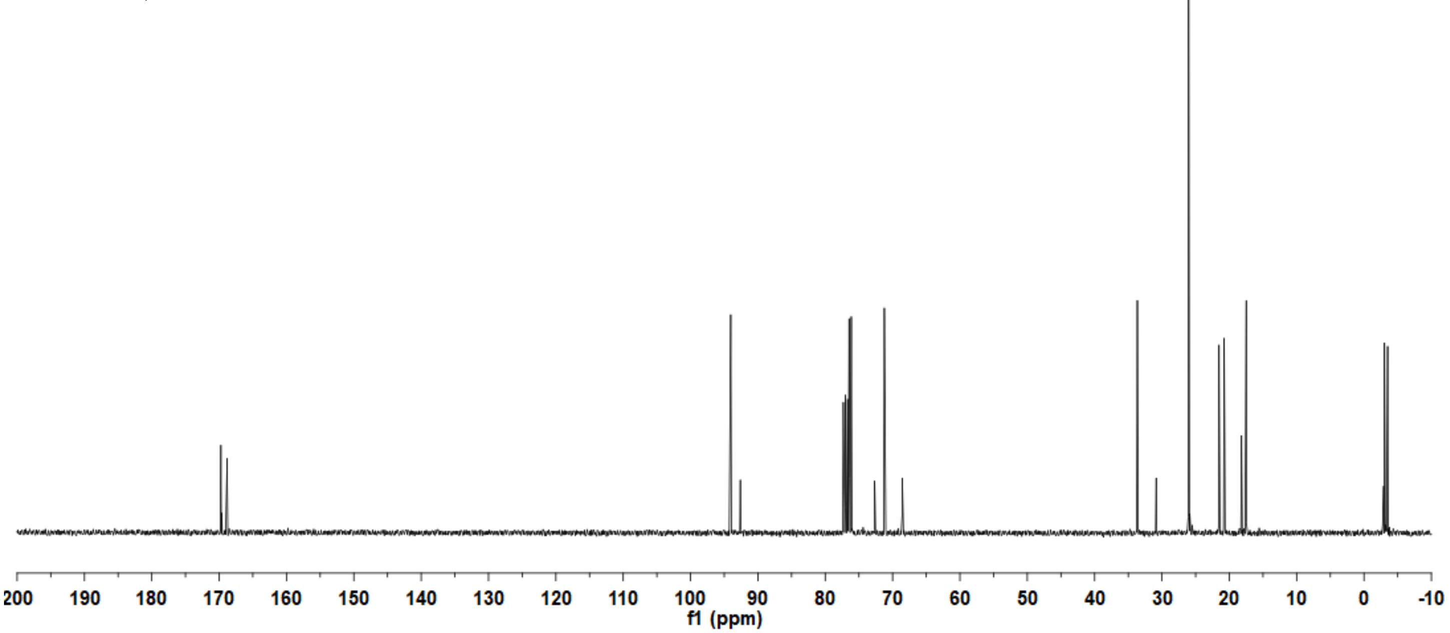

COSY NMR spectrum of 1,4-Di-*O*-acetyl--3-*O*-*tert*-butyldimethylsilyl-2,6-dideoxy-2-iodo-β-D-glucopyranoside **22**

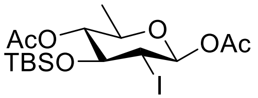

22

(contain some **23**D

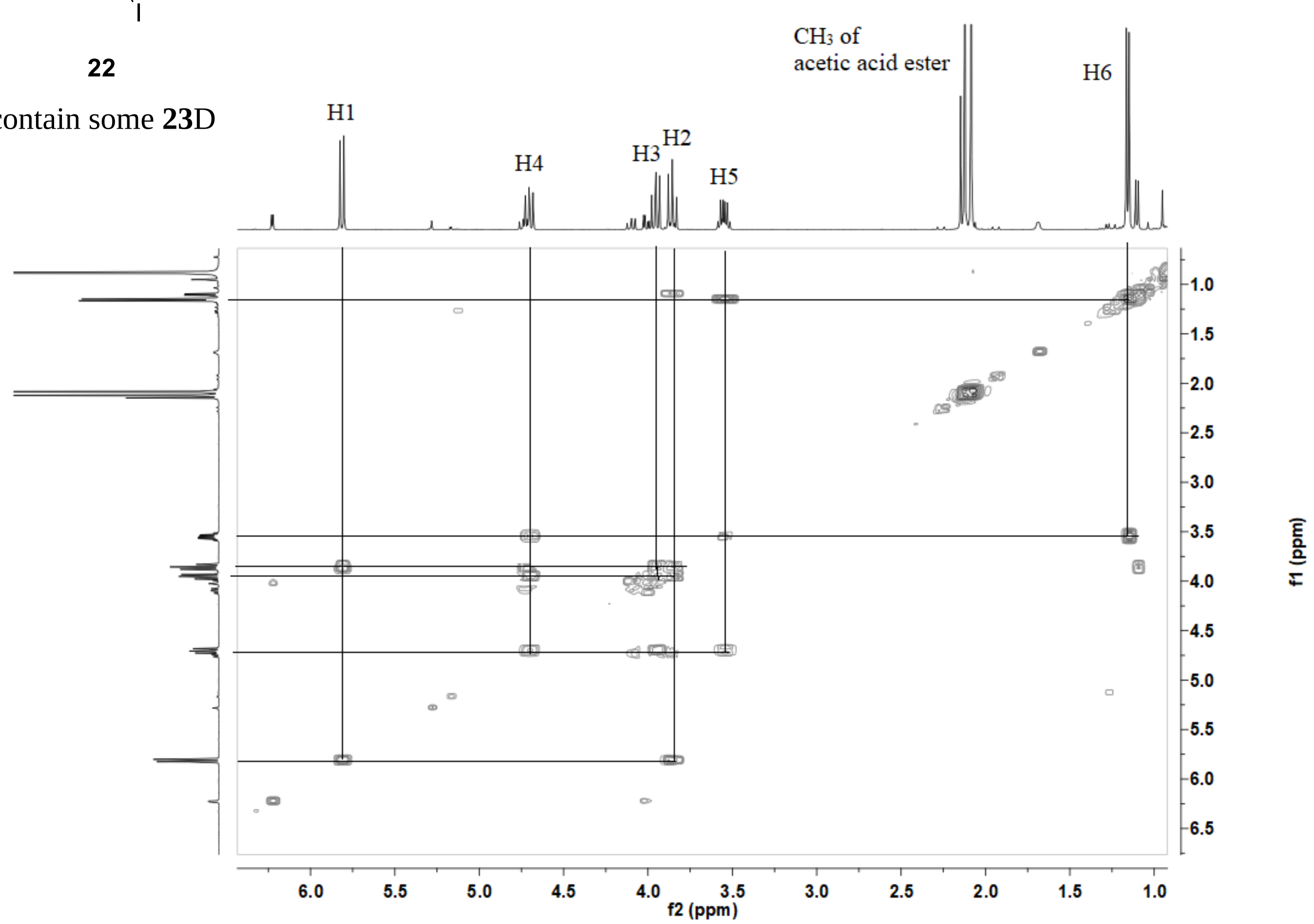

^{1}H NMR spectrum of 1,4-Di-*O*-acetyl--3-*O*-*tert*-butyldimethylsilyl-2,6-dideoxy-2-iodo-α-D-mannopyranoside **23D**

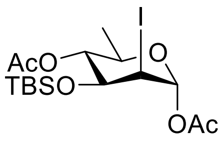

23D

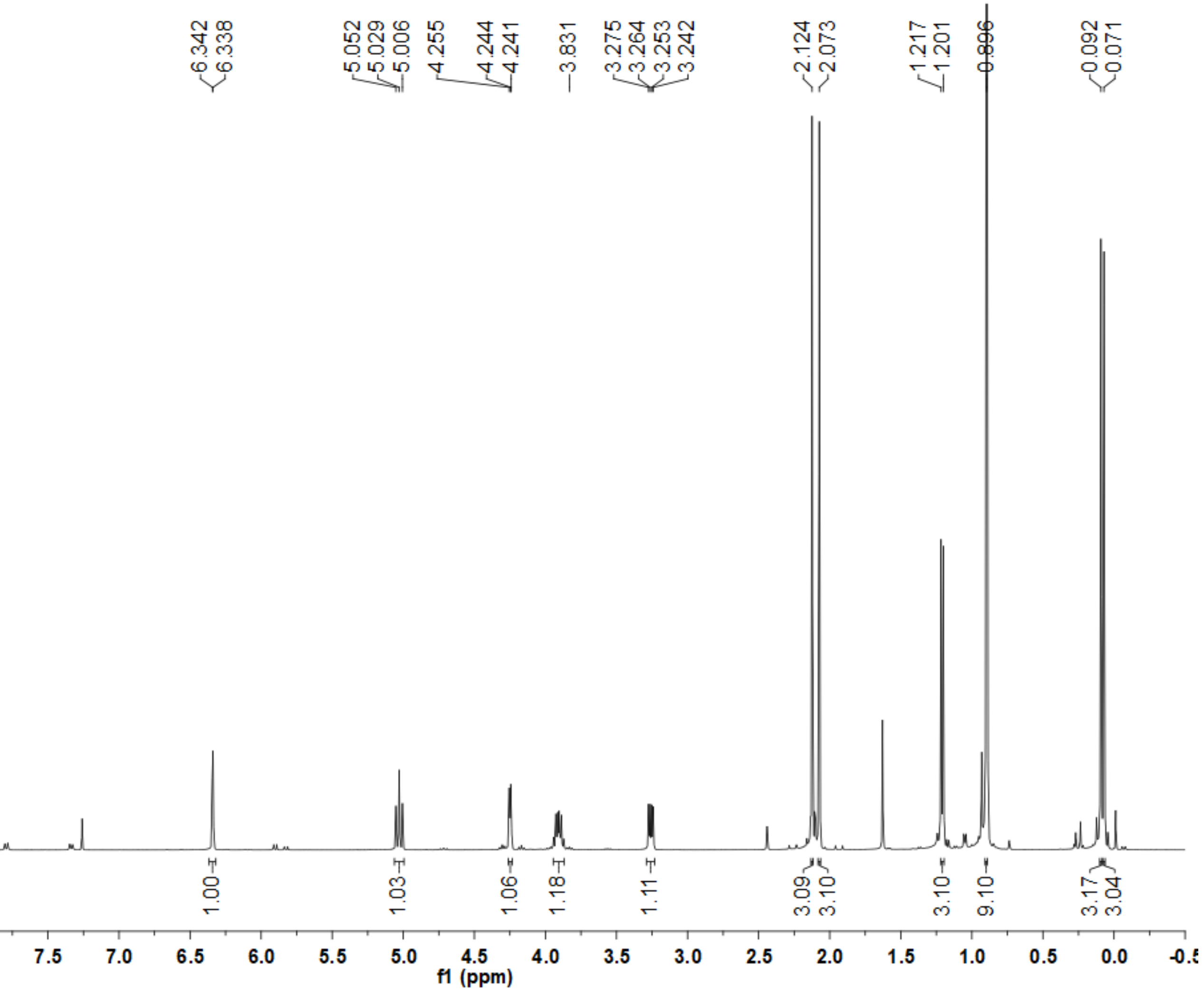

^{1}H NMR spectrum of 1,4-Di-*O*-acetyl-2,6-dideoxy-2-iodo-β-D-glucopyranoside **20**

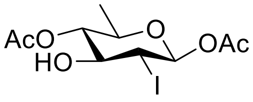

20

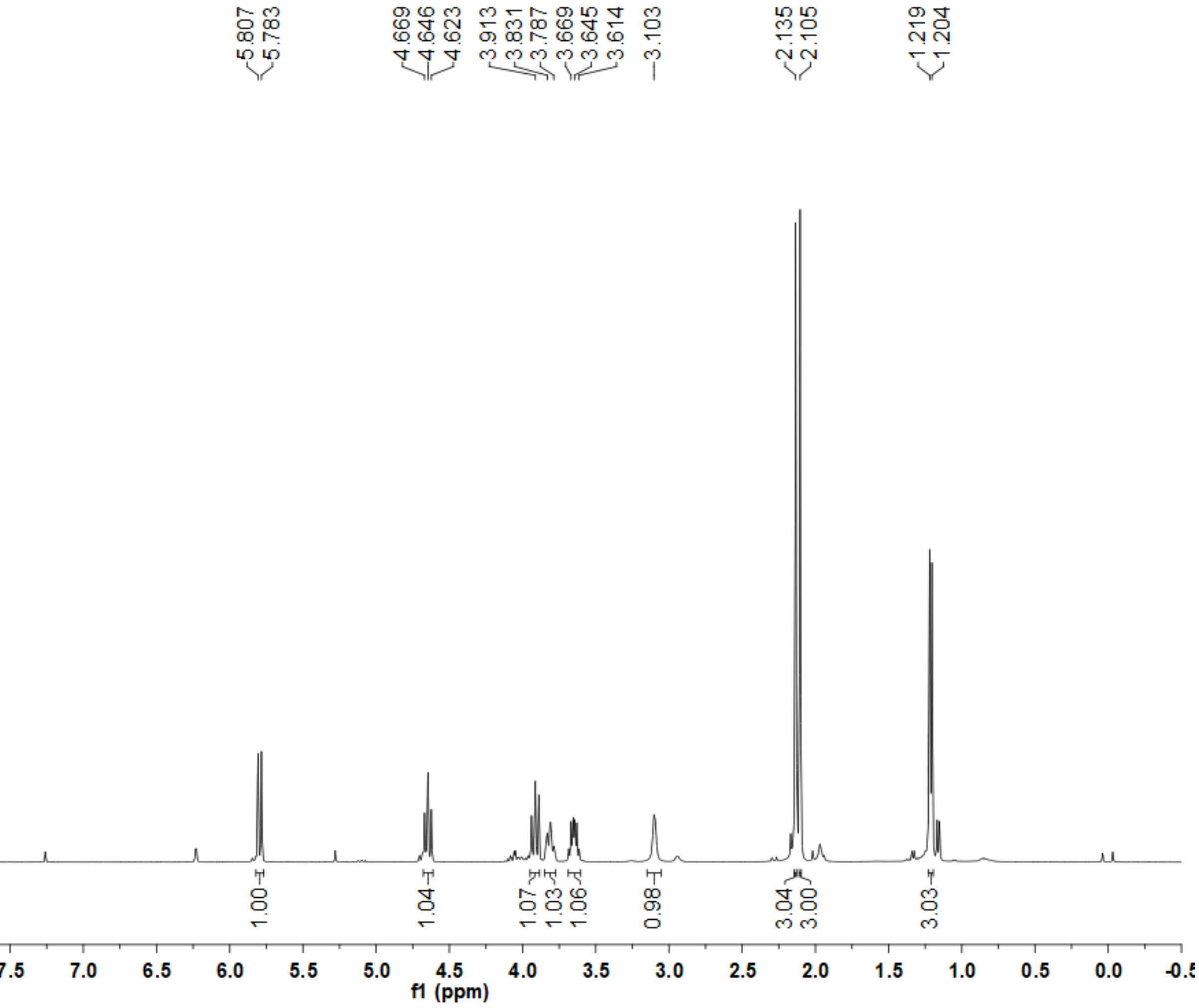

^{13}C NMR spectrum of 1,4-Di-*O*-acetyl-2,6-dideoxy-2-iodo-β-D-glucopyranoside **20**

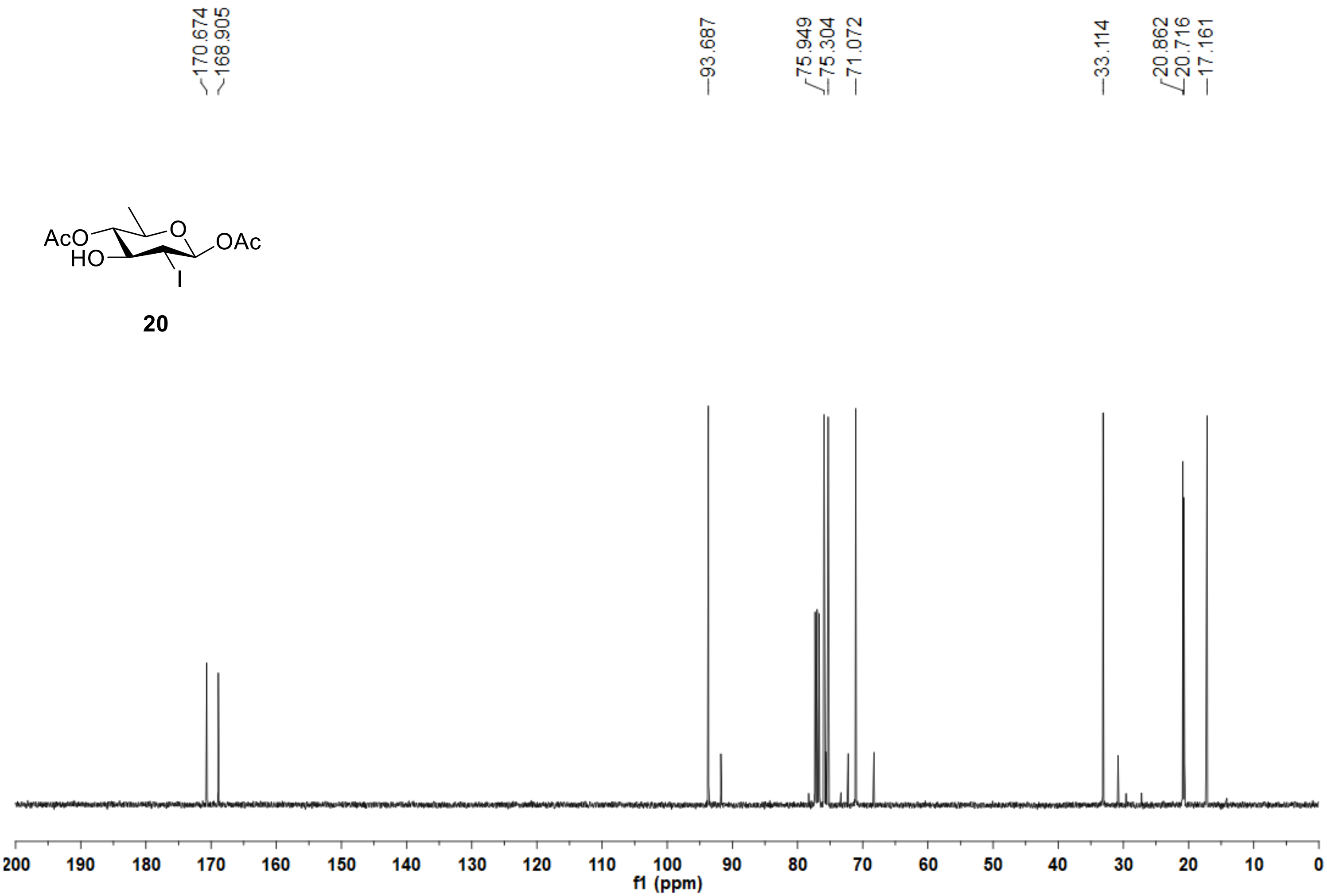

COSY NMR spectrum of 1,4-Di-*O*-acetyl-2,6-dideoxy-2-iodo-β-D-glucopyranoside **20**

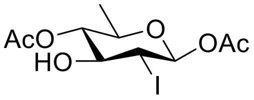

20

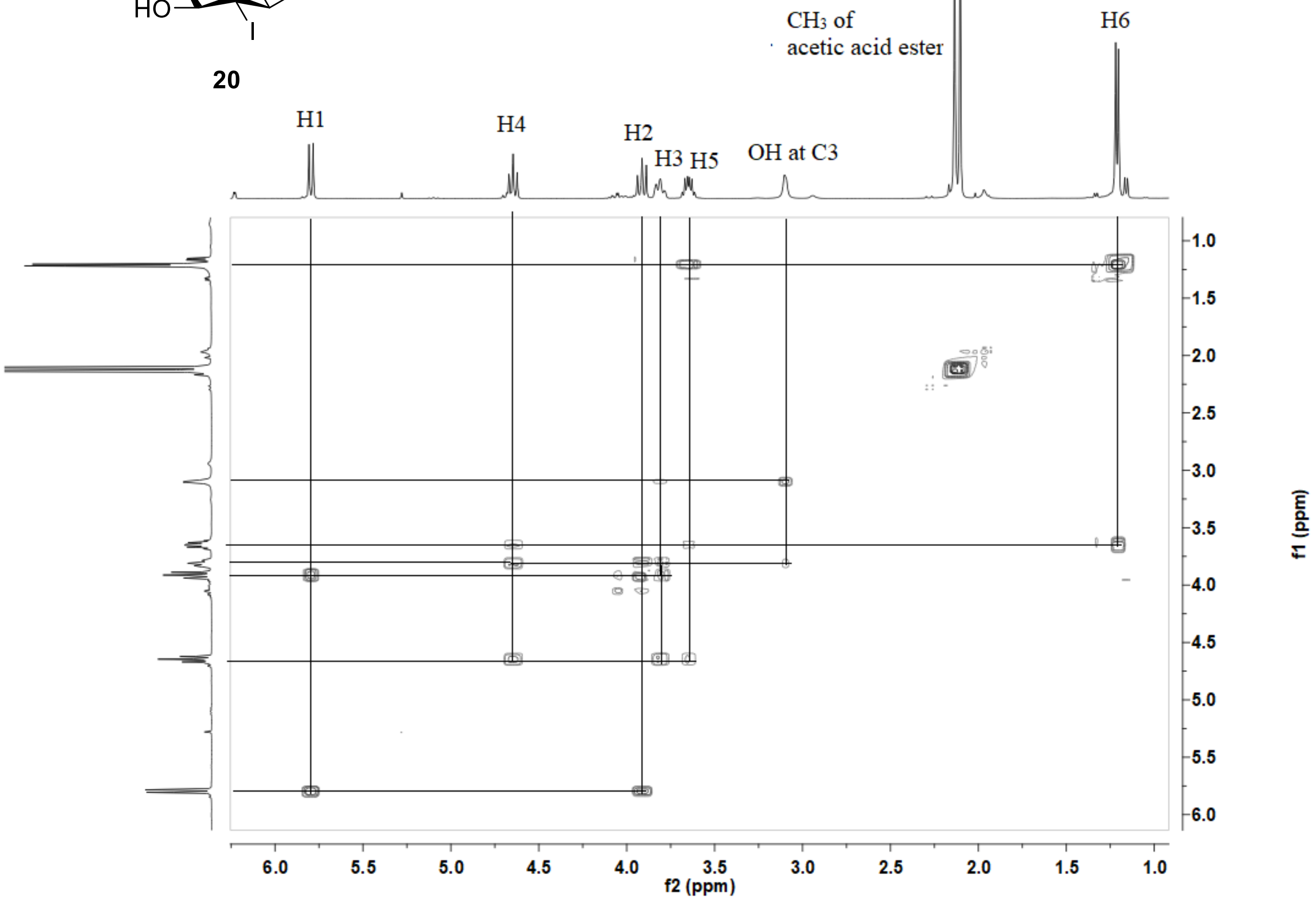

HSQC NMR spectrum of 1,4-Di-*O*-acetyl-2,6-dideoxy-2-iodo-β-D-glucopyranoside **20**

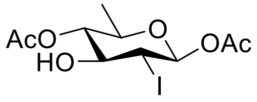

20

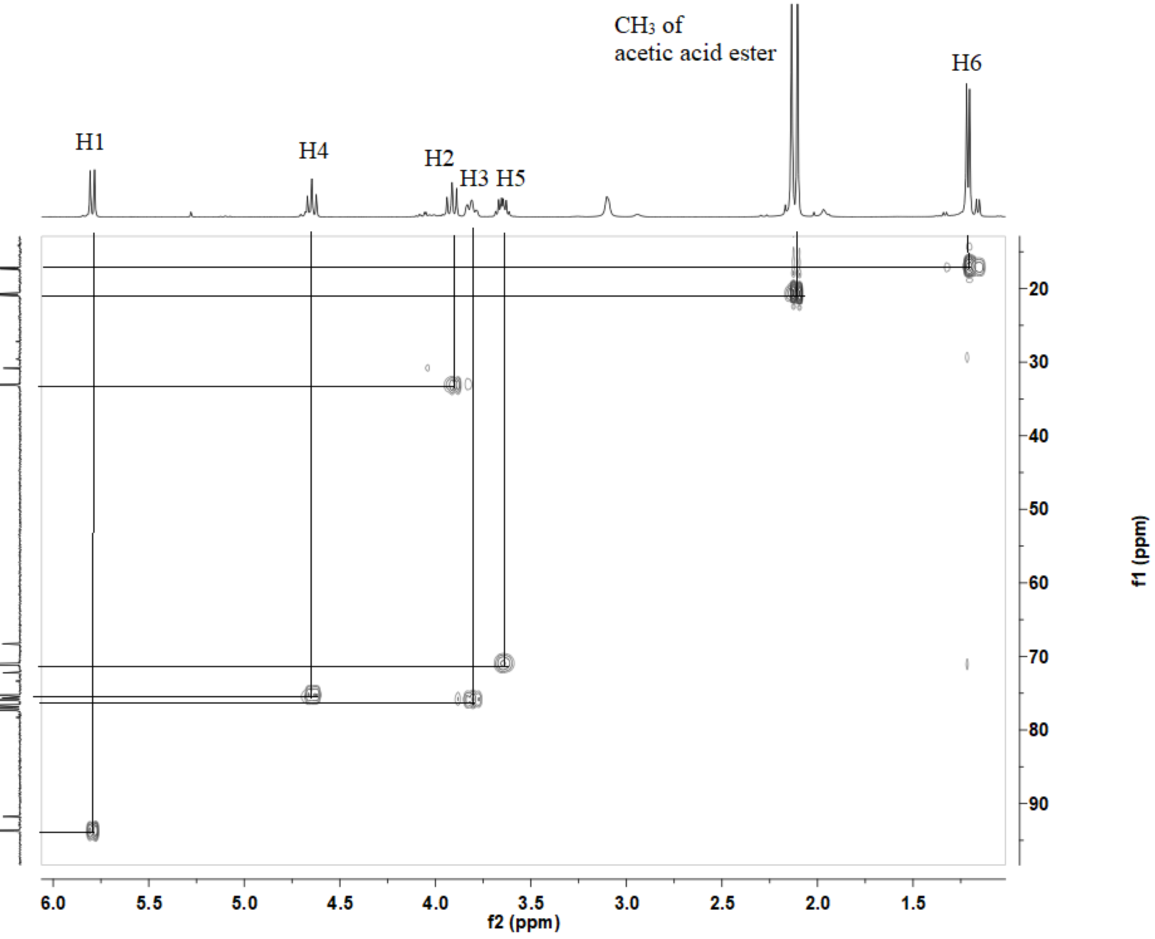

^{1}H NMR spectrum of 3-*O*-Acetyl-6-iodo-D-glucal **25**

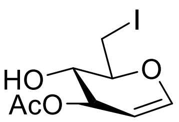

25

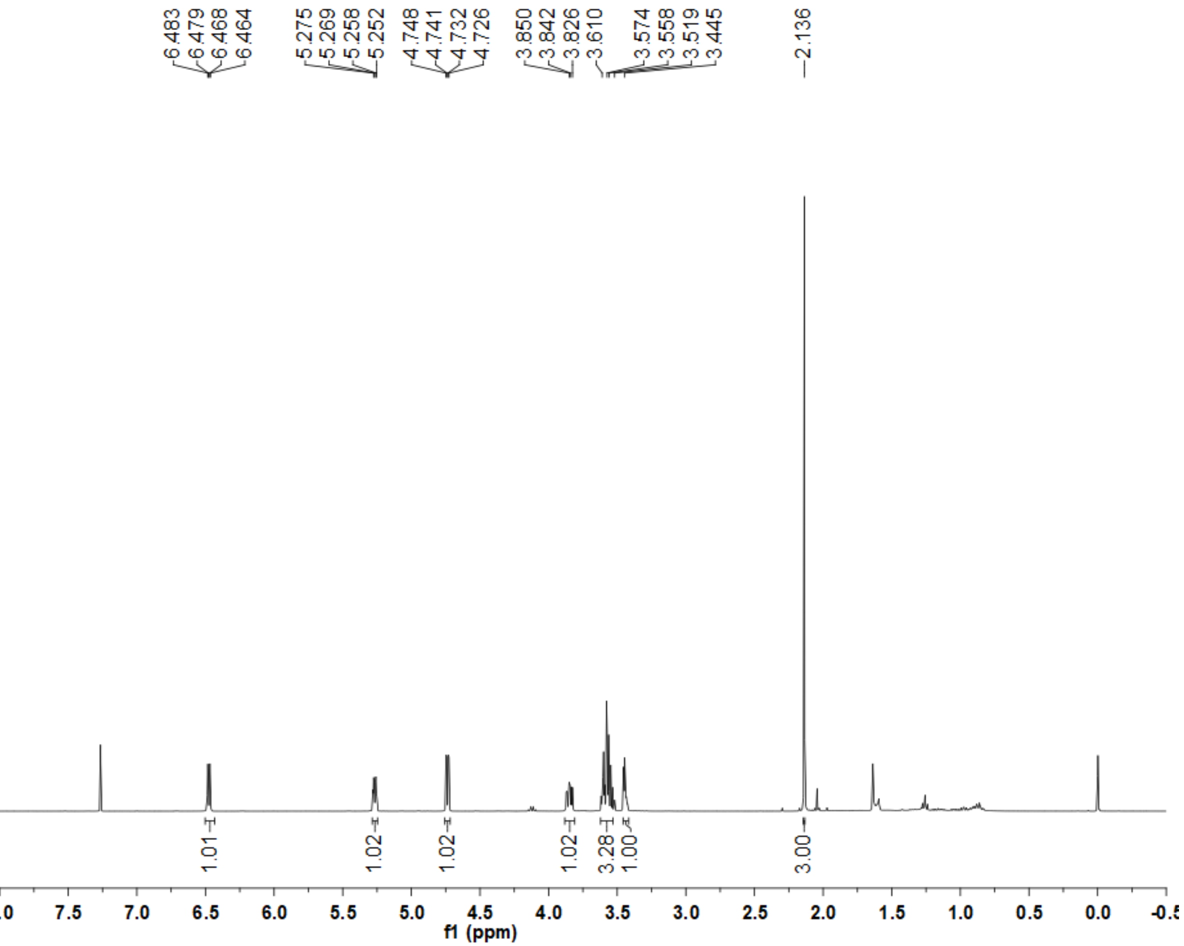

^{13}C NMR spectrum of 3-*O*-Acetyl-6-iodo-D-glucal **25**

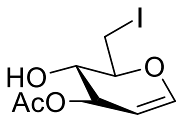

25

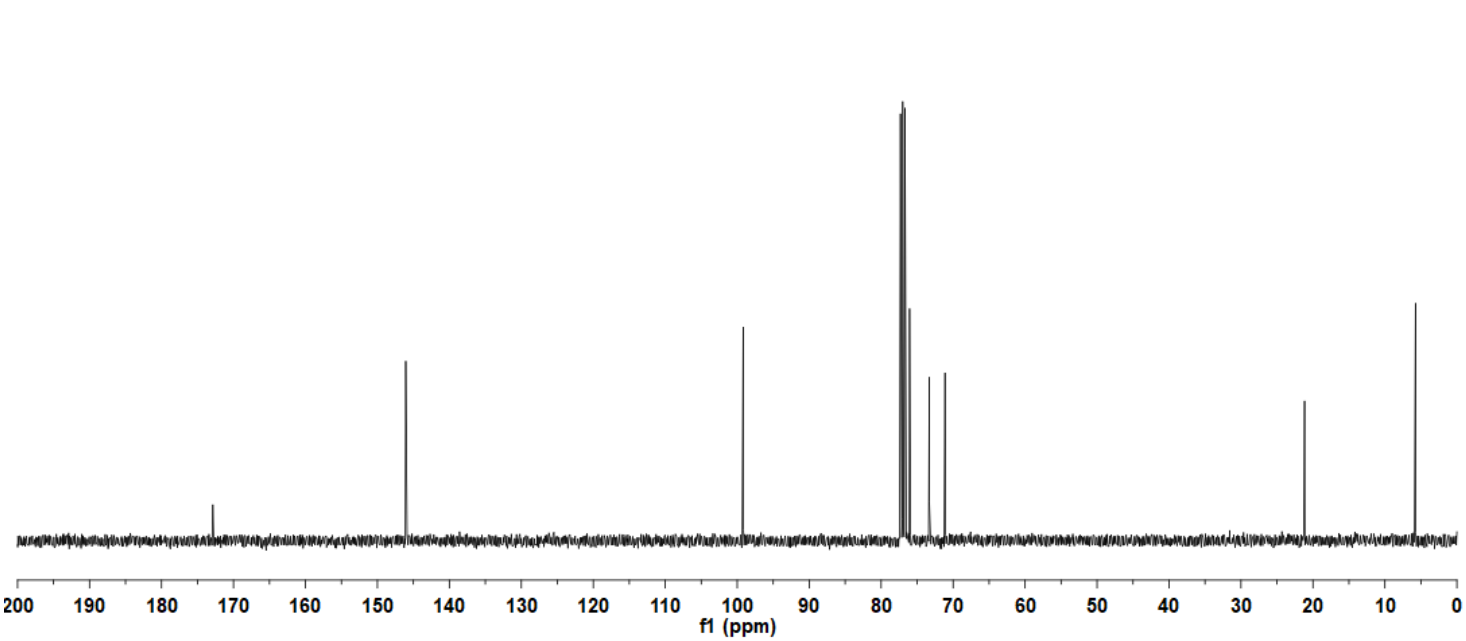

COSY NMR spectrum of 3-*O*-Acetyl-6-iodo-D-glucal **25**

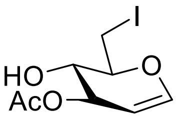

25

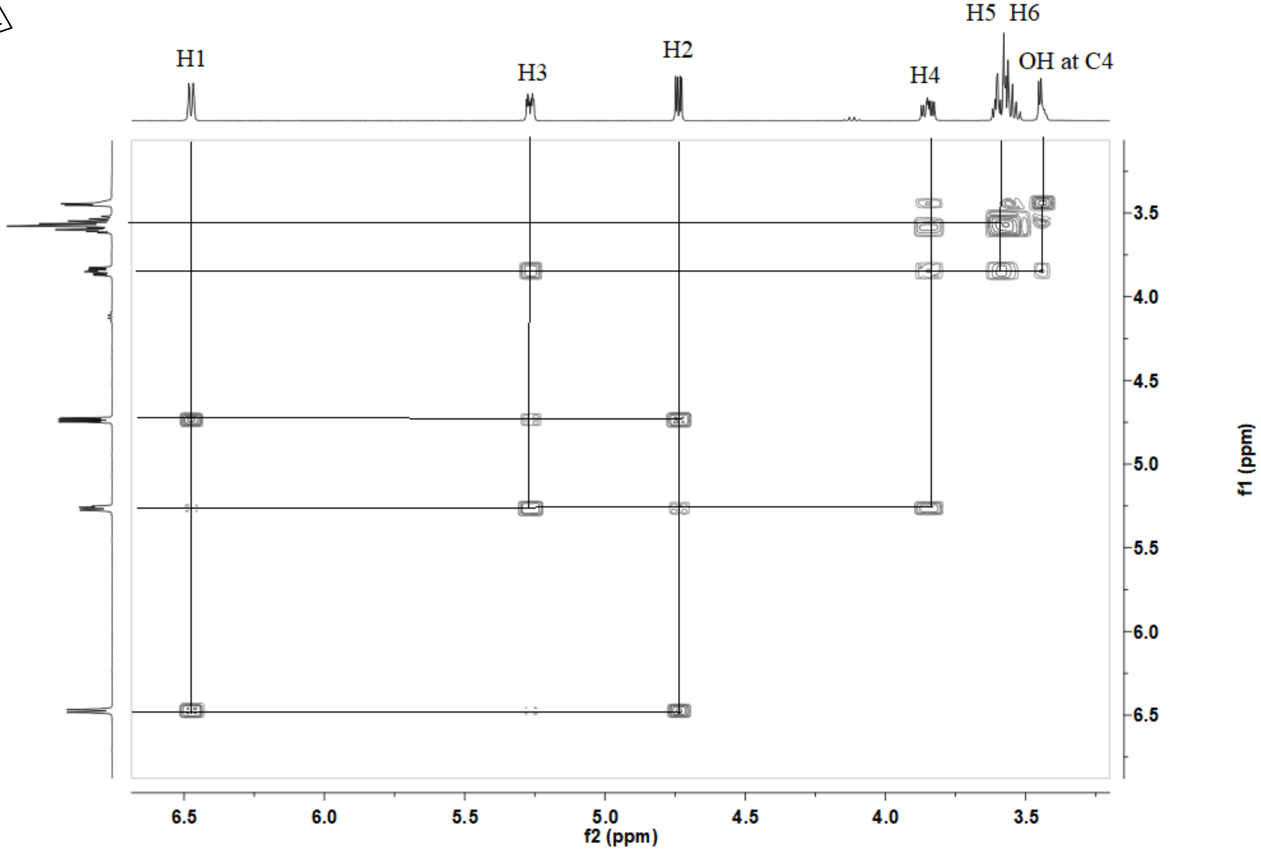

^{1}H NMR spectrum of 3-*O*-Acetyl-6-iodo-4-*O*-*tert*-butyldimethylsilyl-D-glucal **25A**

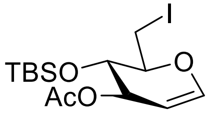

25A

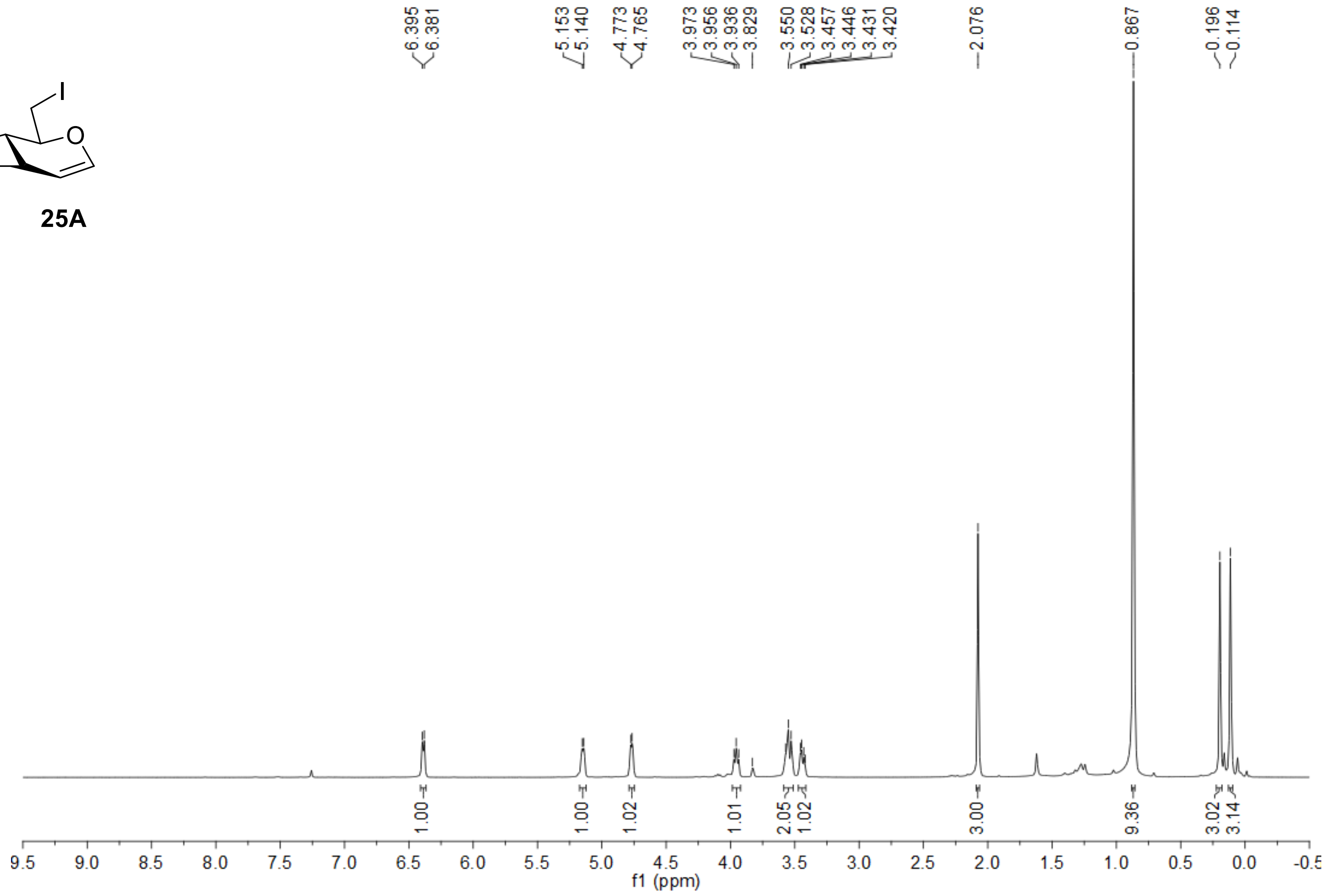

^{13}C NMR spectrum of 3-*O*-Acetyl-6-iodo-4-*O*-*tert*-butyldimethylsilyl-D-glucal **25A**

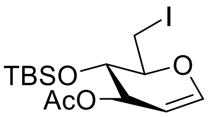

25A

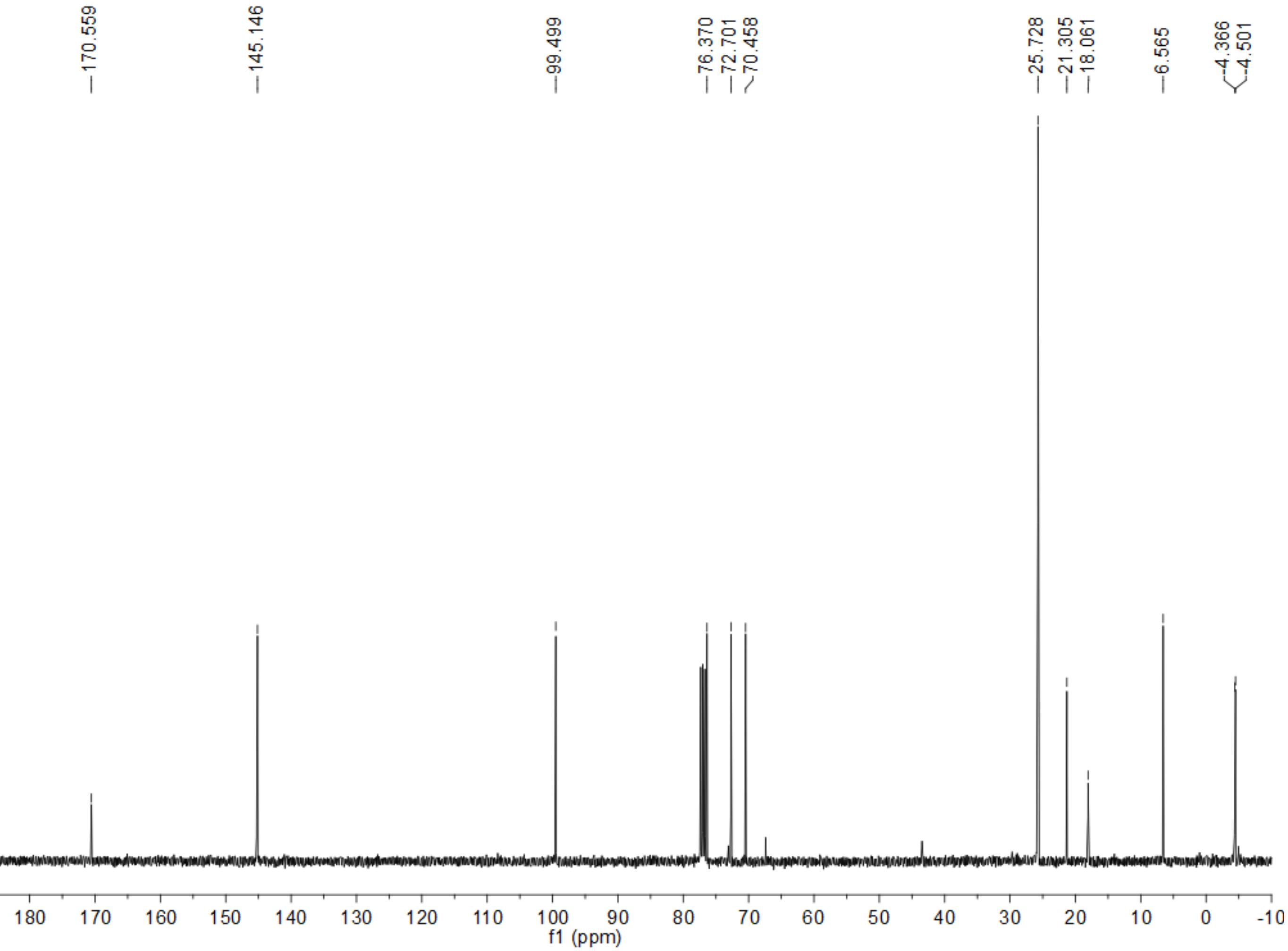

^{1}H NMR spectrum of 3-*O*-Acetyl-6-iodo-4-*O*-*tert*-butyldimethylsilyl-2,6-dideoxy-D-glucopyranosyl acetate **21**

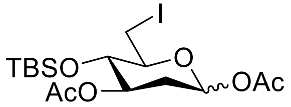

21

(α:β = 3:1)

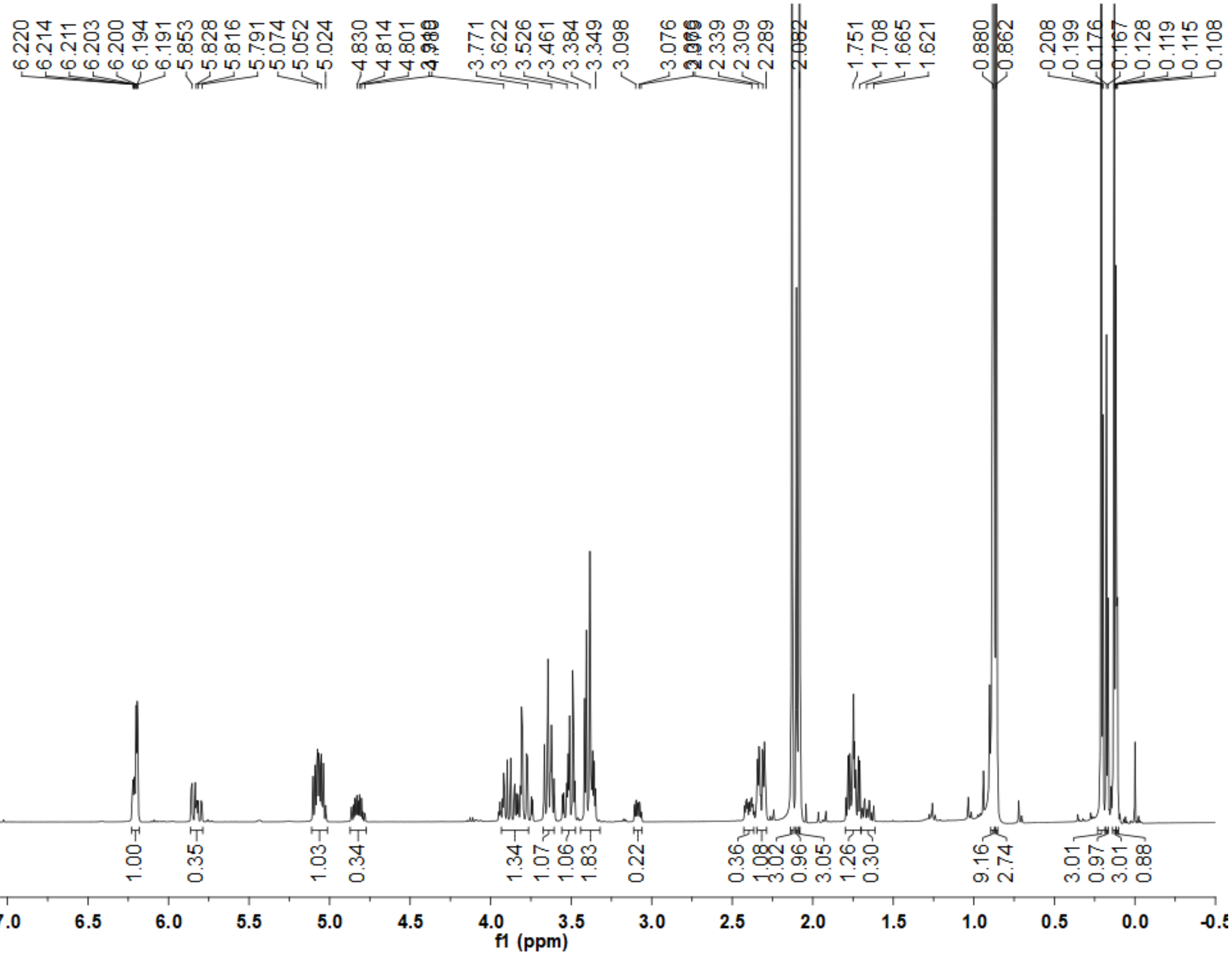

^{13}C NMR spectrum of 3-*O*-Acetyl-6-iodo-4-*O*-*tert*-butyldimethylsilyl-2,6-dideoxy-D-glucopyranosyl Acetate **21**

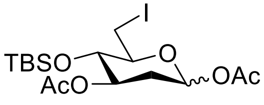

21

(α:β = 3:1)

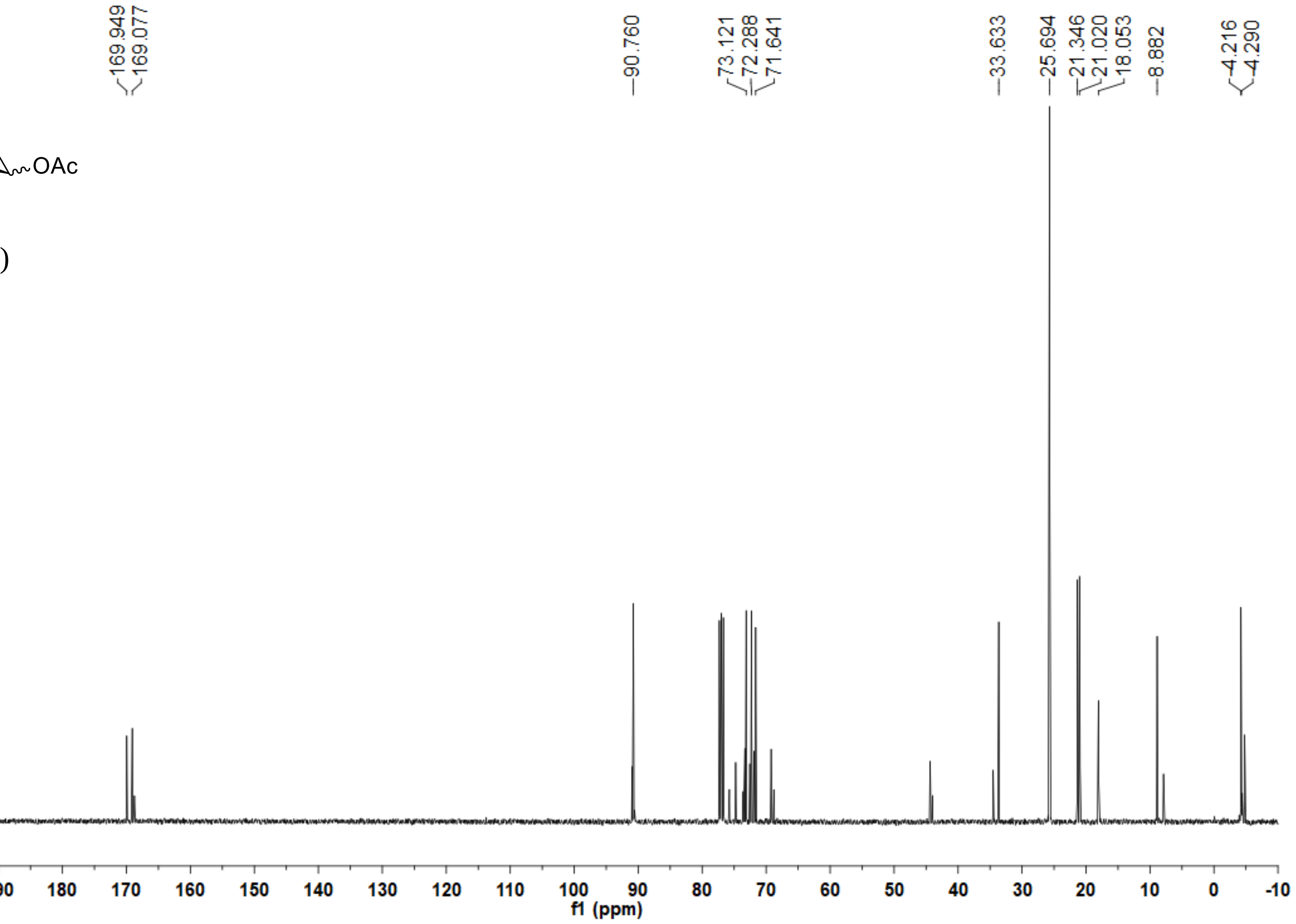

COSY NMR spectrum of 3-*O*-Acetyl-6-iodo-4-*O*-*tert*-butyldimethylsilyl-2,6-dideoxy-D-glucopyranosyl Acetate **21**

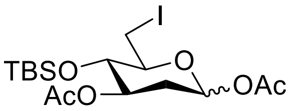

21

(α:β = 3:1)

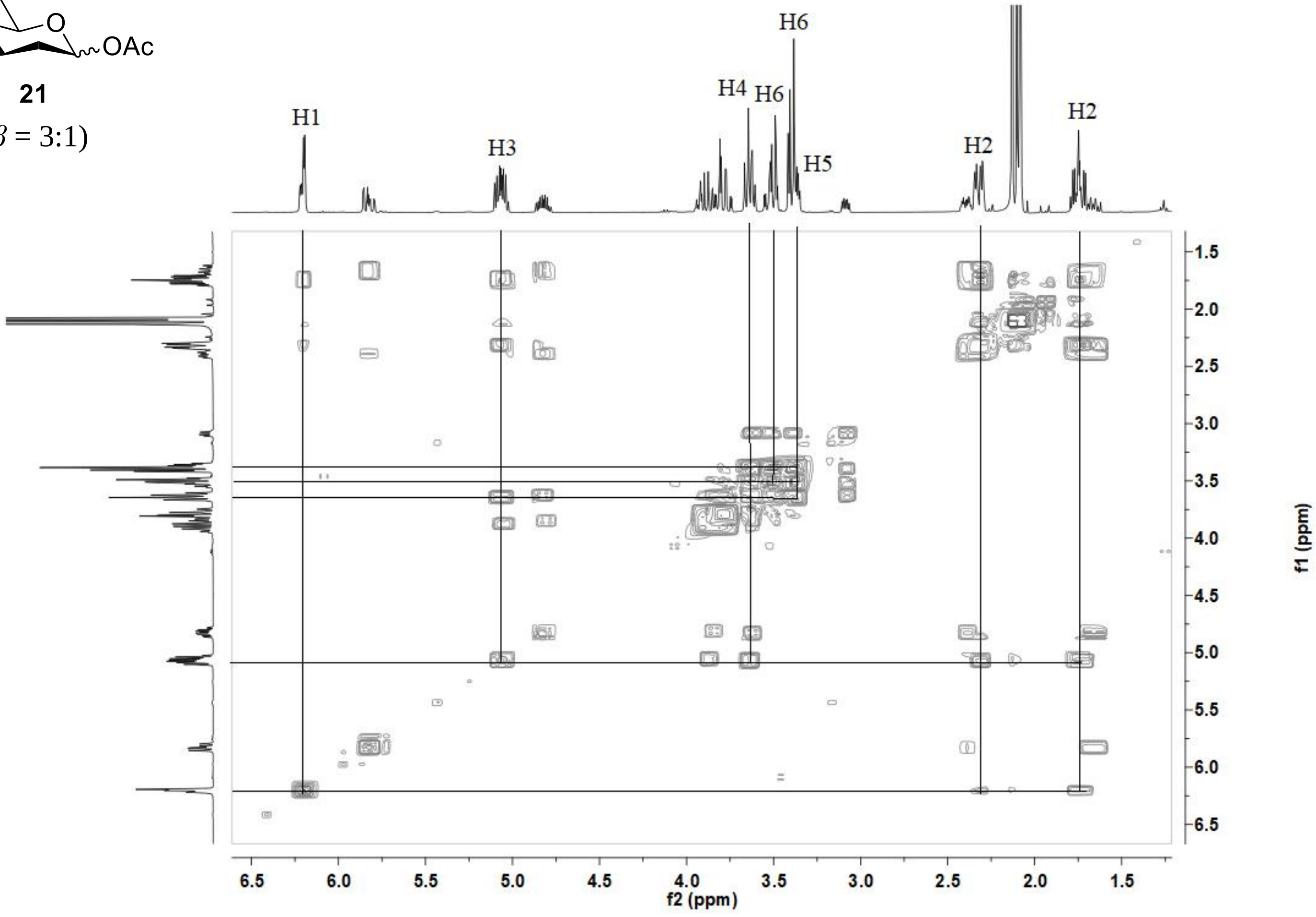

HSQC NMR spectrum of 3-*O*-Acetyl-6-iodo-4-*O*-*tert*-butyldimethylsilyl-2,6-dideoxy-D-glucopyranosyl Acetate **21**

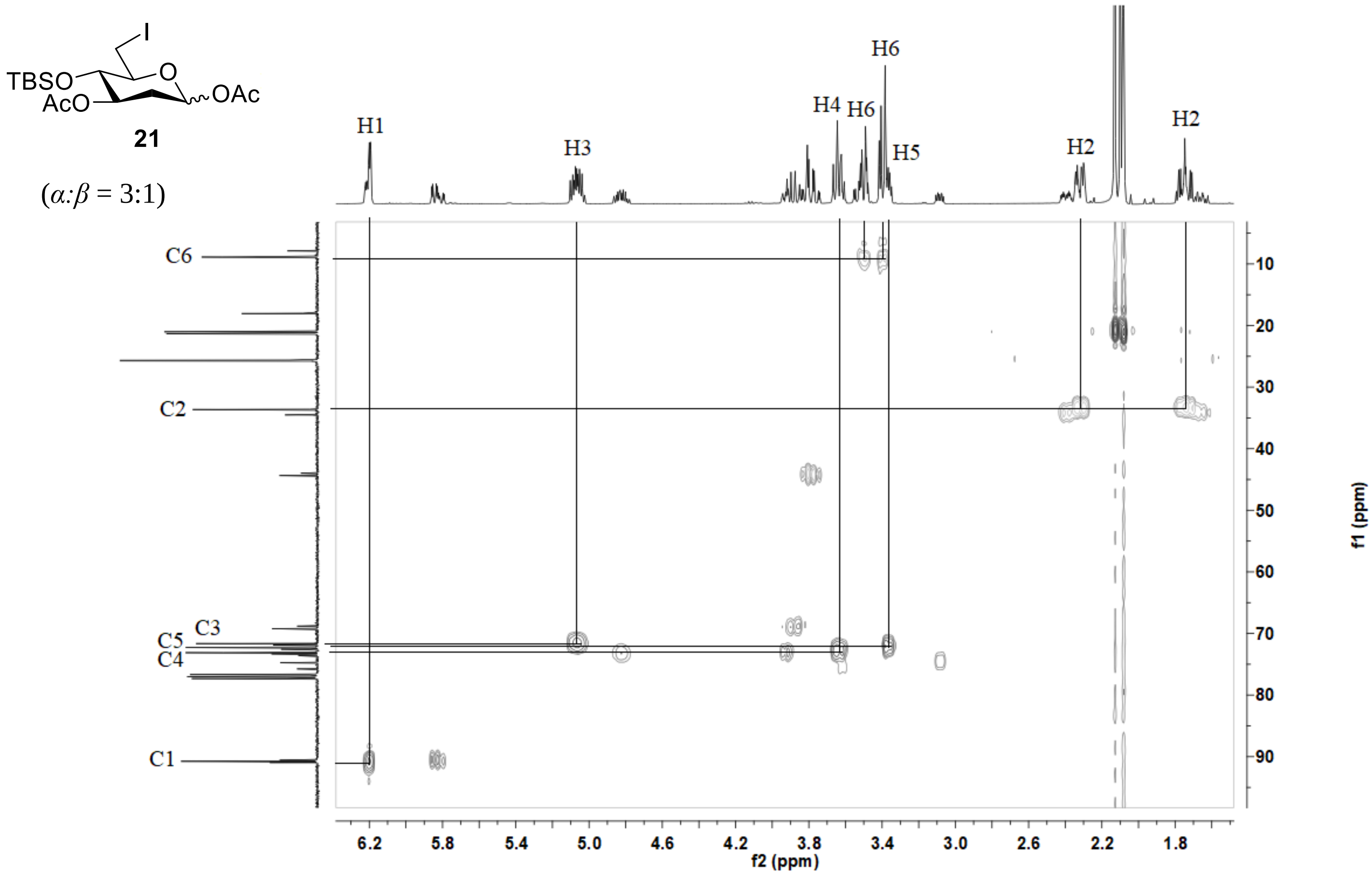

^{1}H NMR spectrum of Anhydrolandomycinonyl *β*-Olivoside **26**

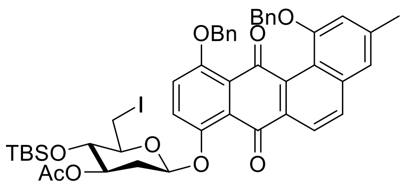

26

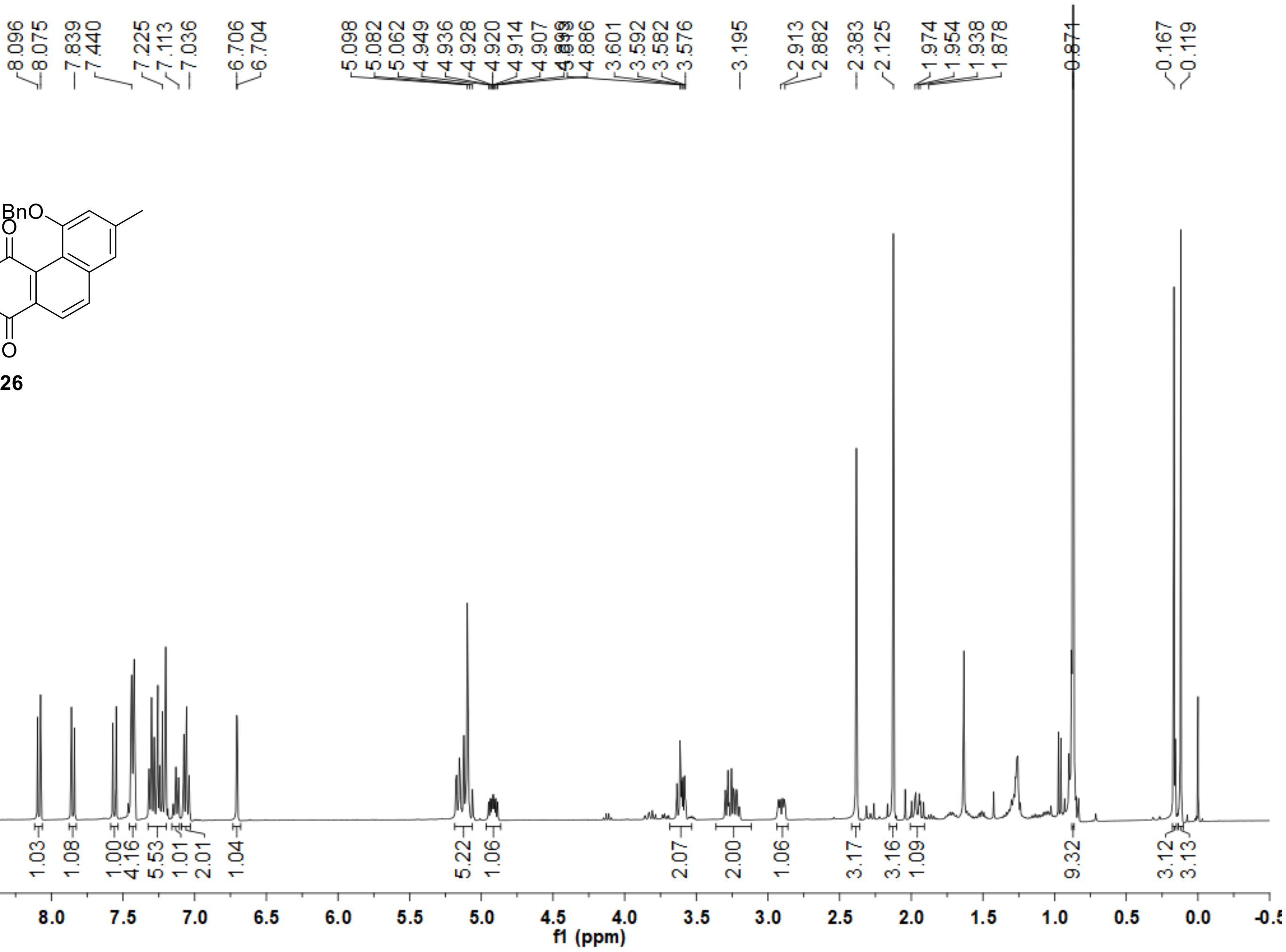

^{13}C NMR spectrum of Anhydrolandomycinonyl β-Olivoside **26**

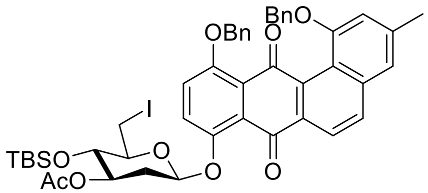

26

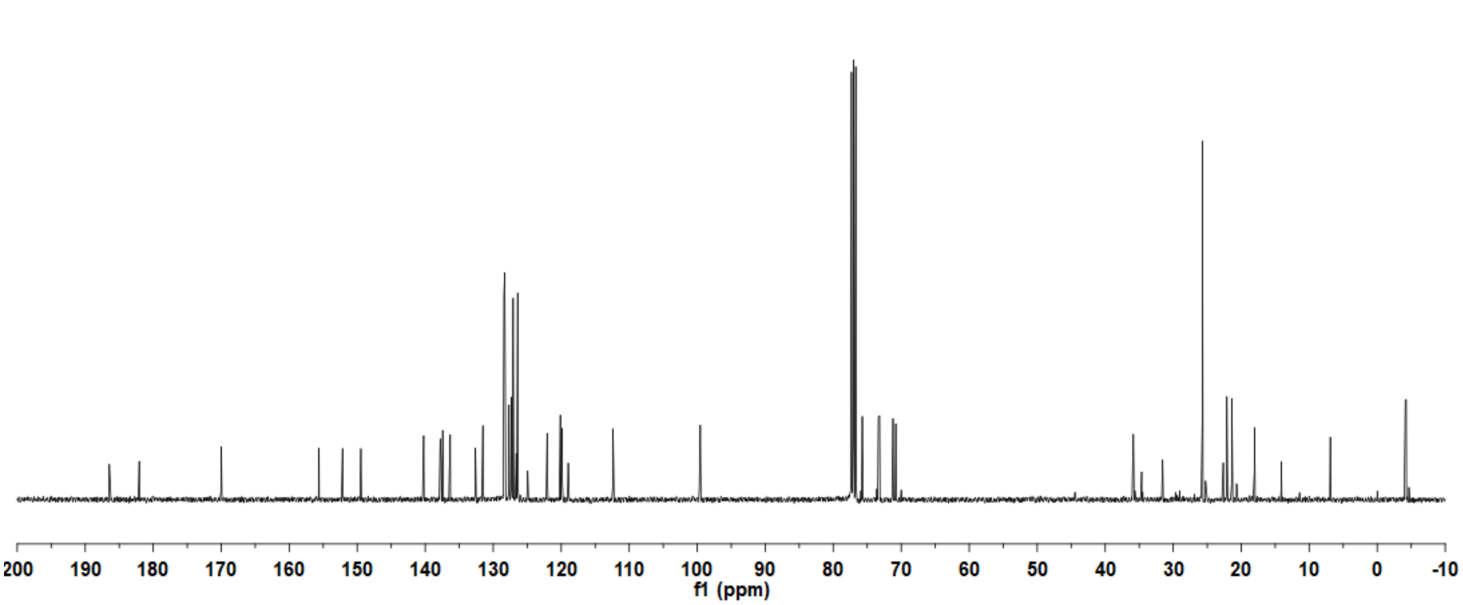

COSY NMR spectrum of Anhydrolandomycinonyl β-Olivoside **26**

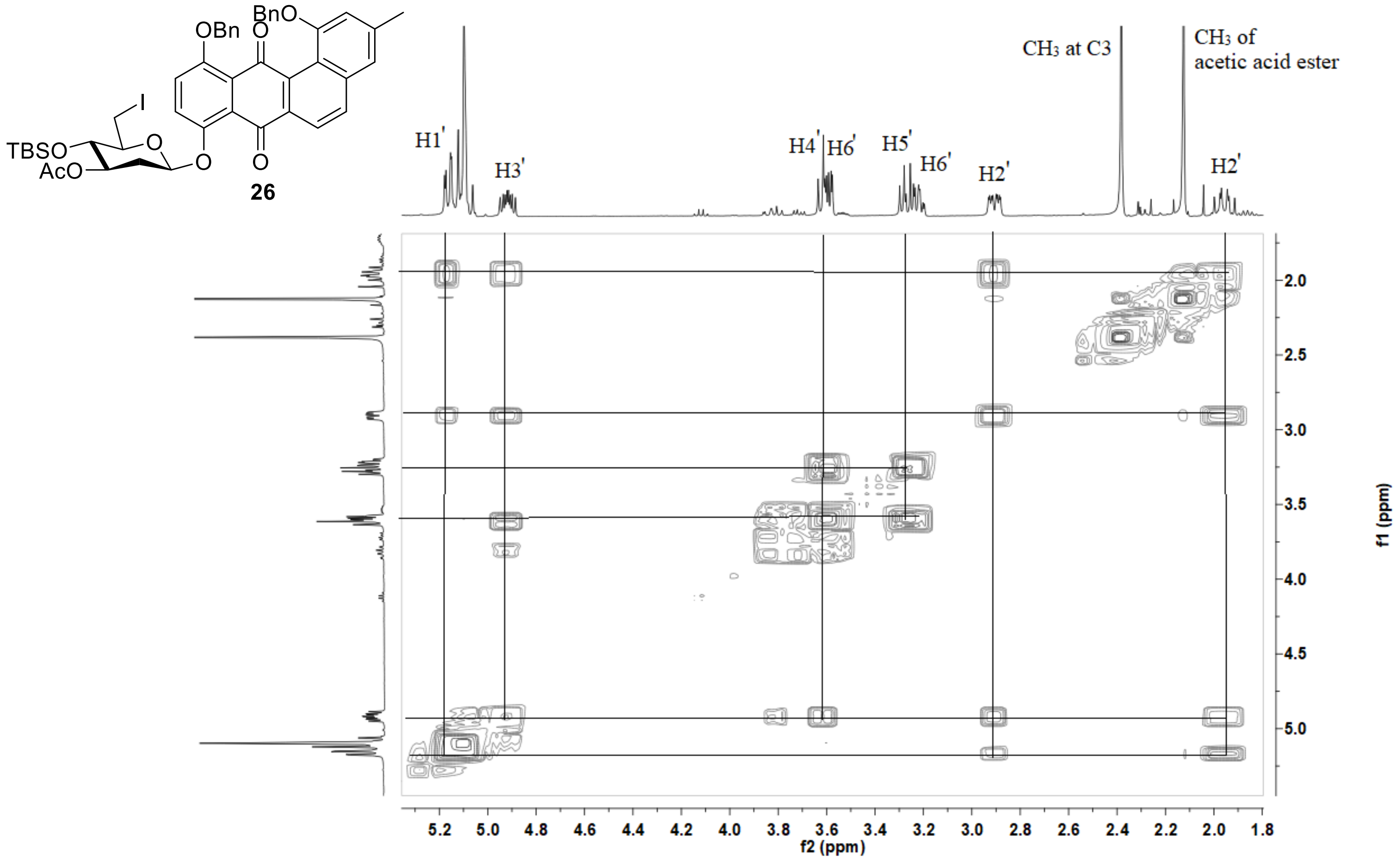

HSQC NMR spectrum of Anhydrolandomycinonyl *β*-Olivoside **26**

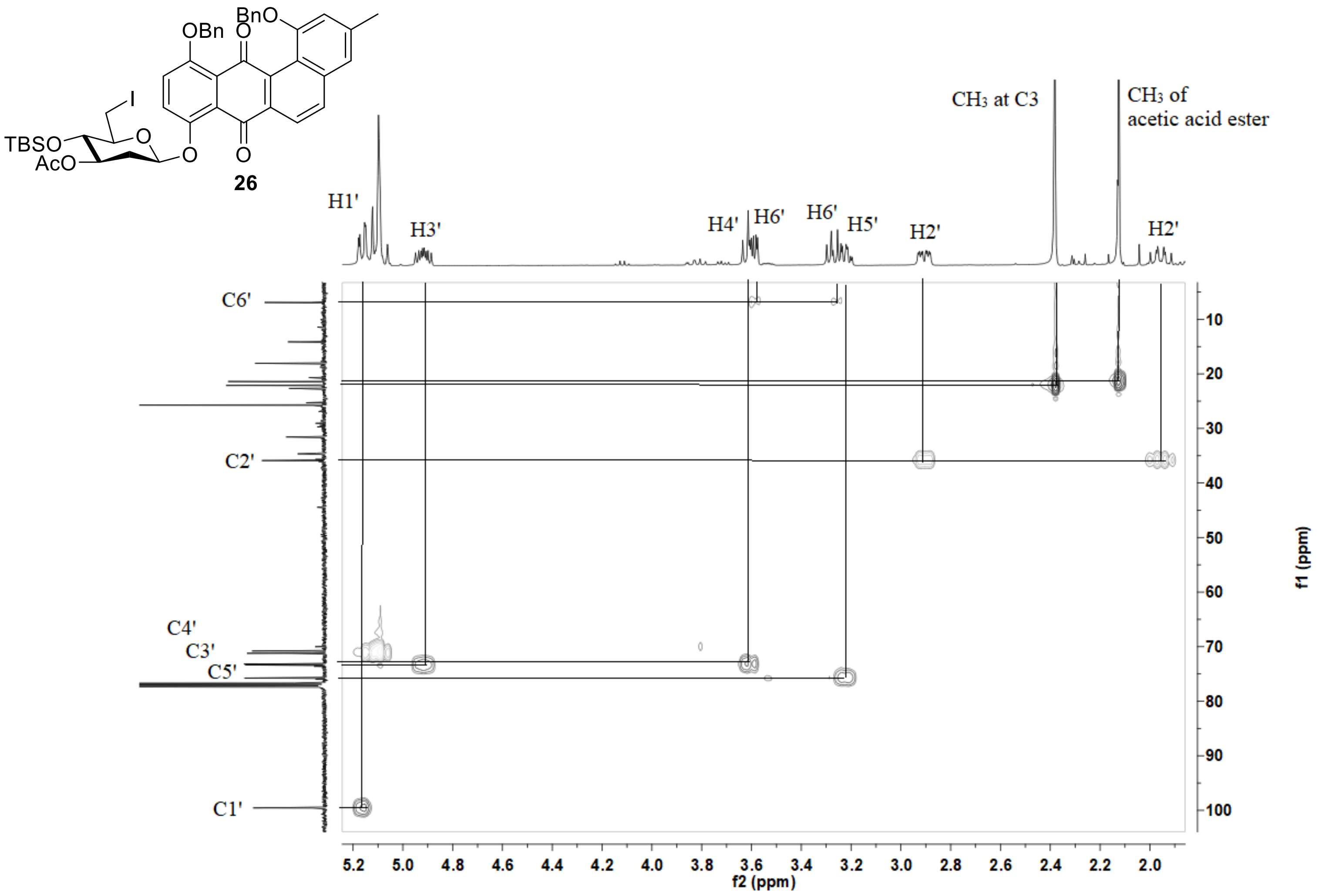

^{1}H NMR spectrum of Anhydrolandomycinonyl *β*-Olivoside Acceptor **27**

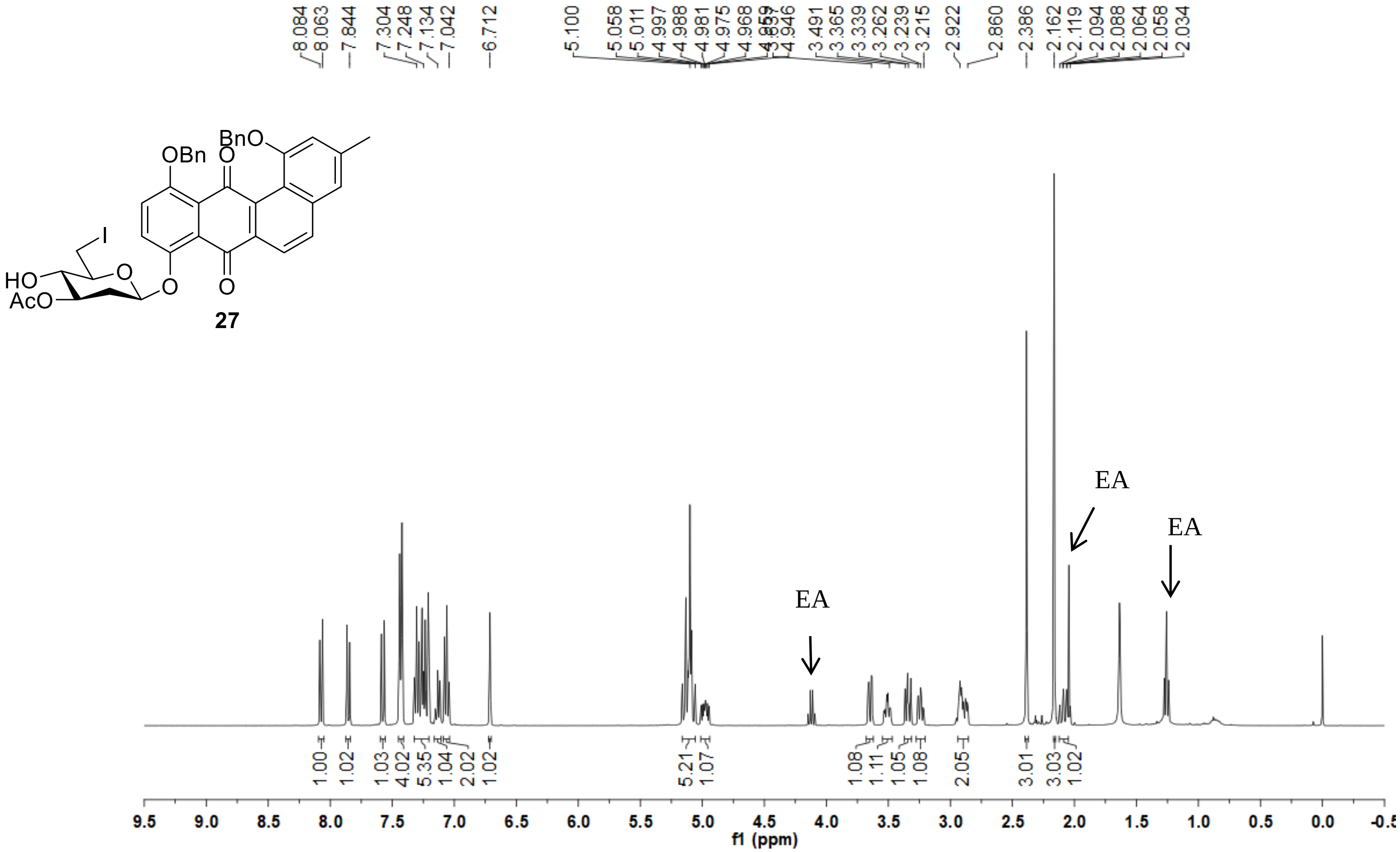

^{13}C NMR spectrum of Anhydrolandomycinonyl *β*-Olivoside Acceptor **27**

27

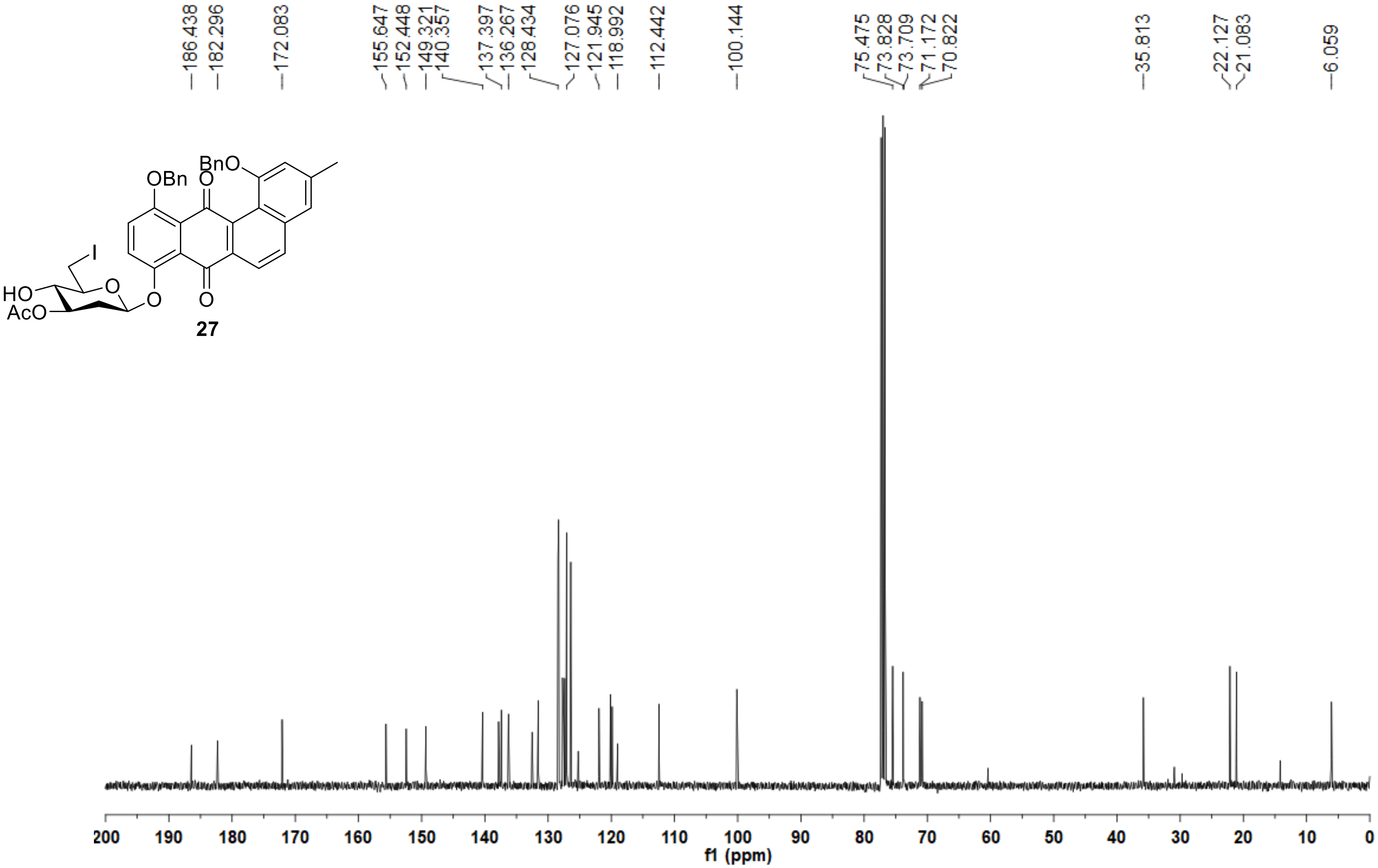

COSY NMR spectrum of Anhydrolandomycinonyl β-Olivoside Acceptor **27**

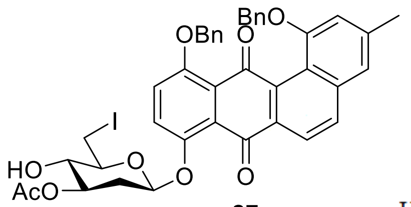

27

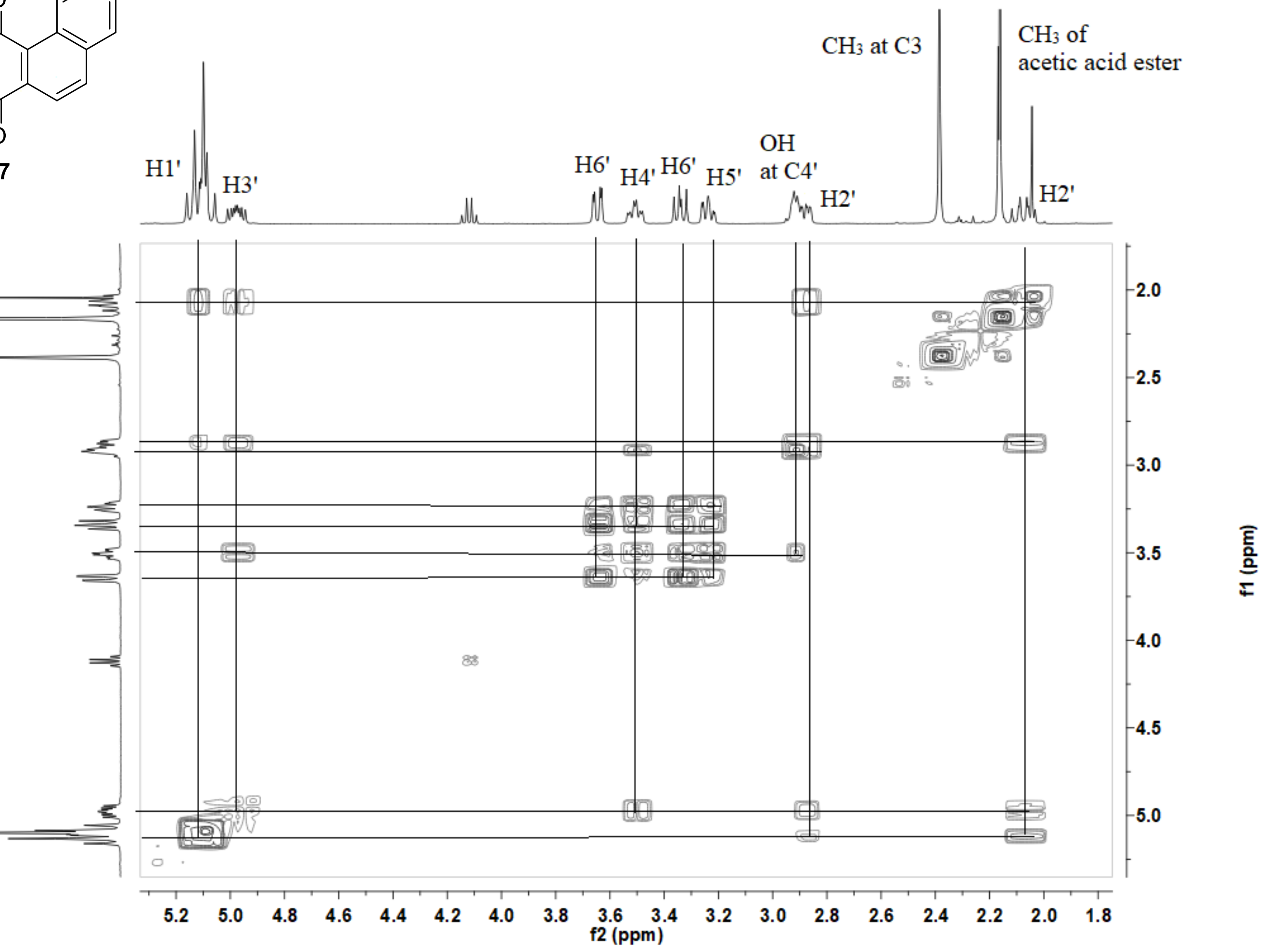

HSQC NMR spectrum of Anhydrolandomycinonyl β-Olivoside Acceptor **27**

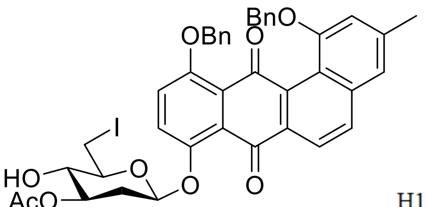

27

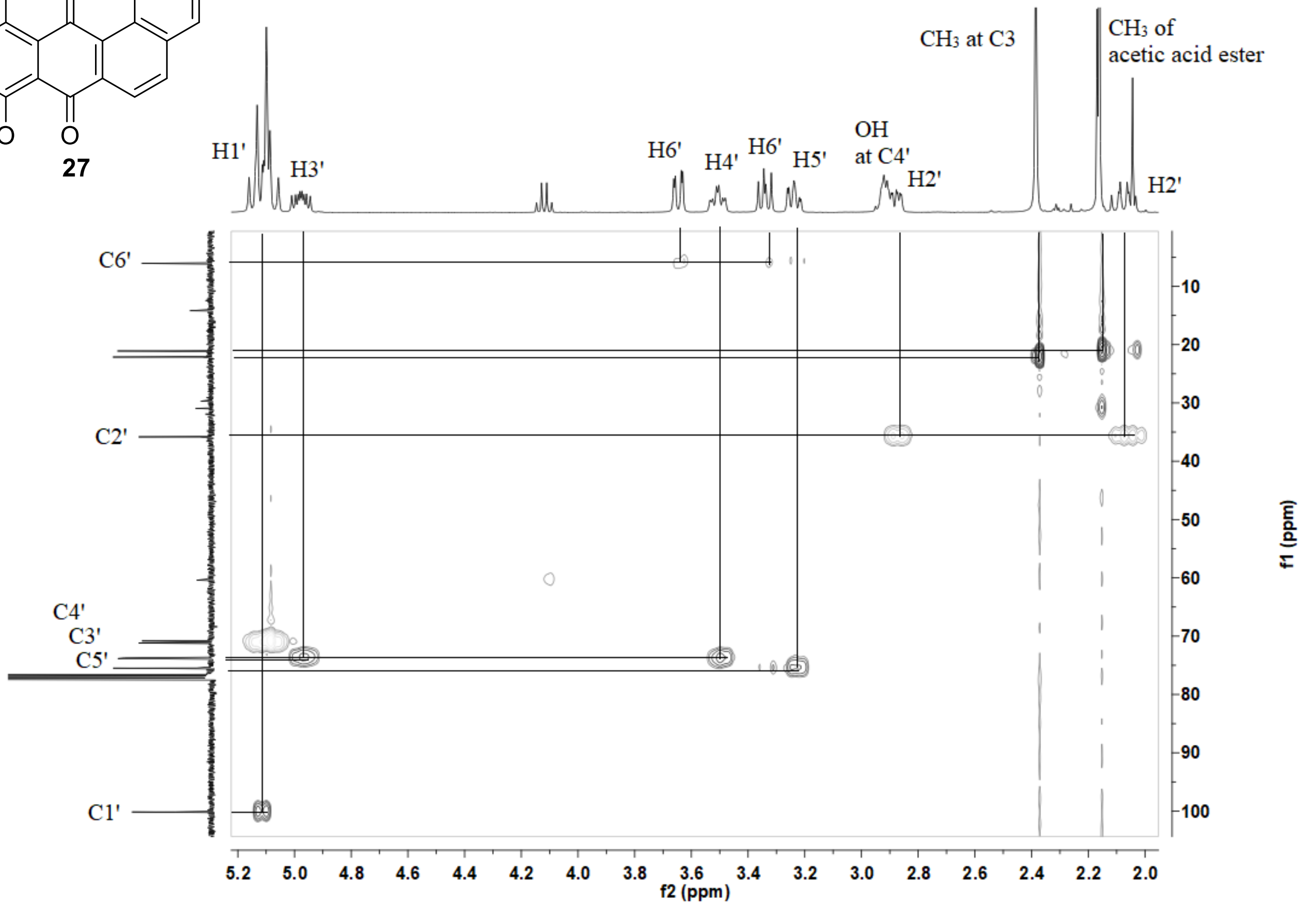

^{1}H NMR spectrum of α-Rhodinosyl-(1,3)-Olivosyl Acetate **28**

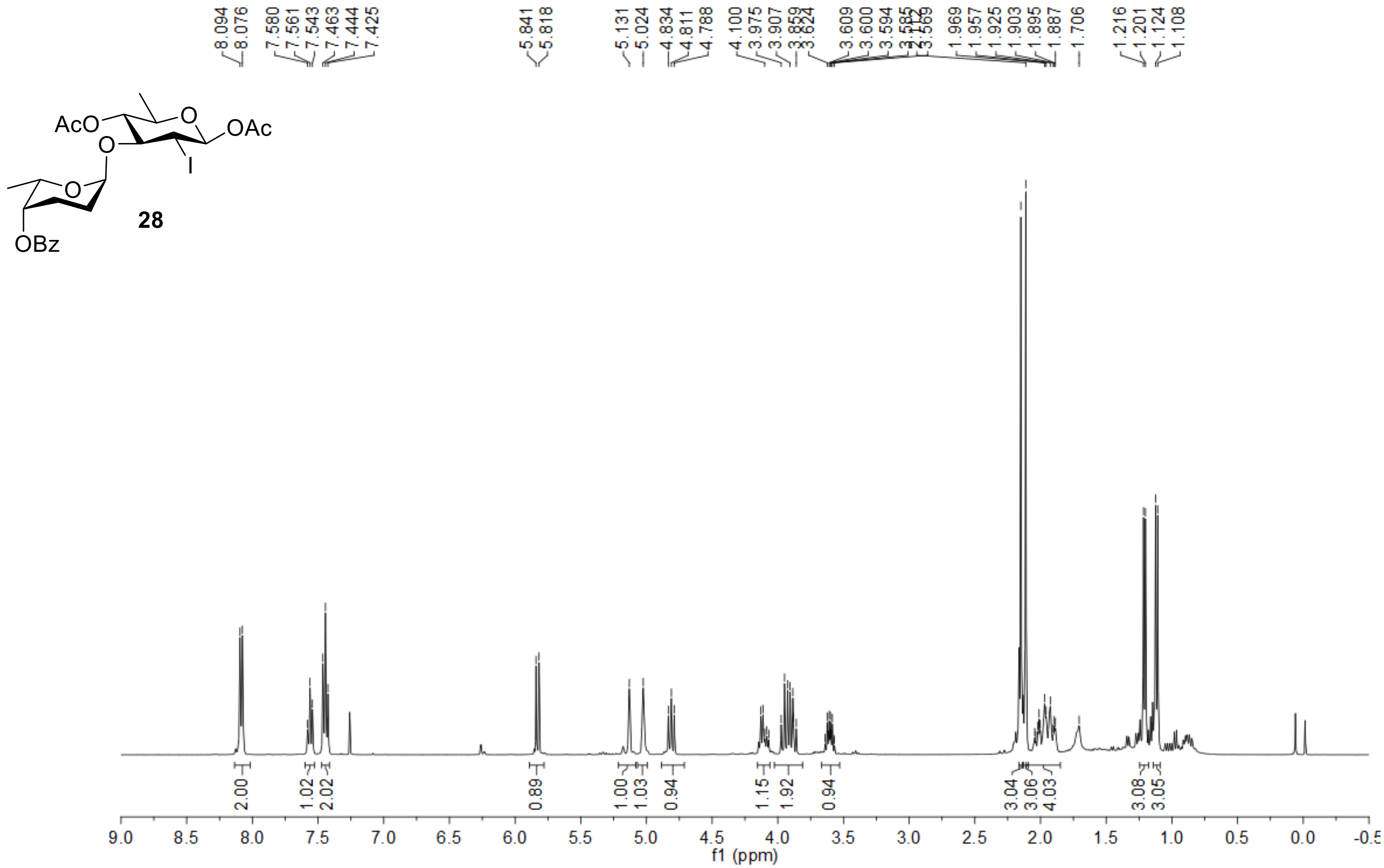

^{13}C NMR spectrum of *α*-Rhodinosyl-(1,3)-Olivosyl Acetate **28**

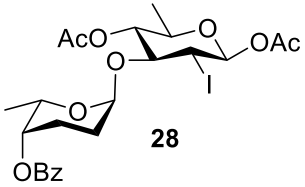

28

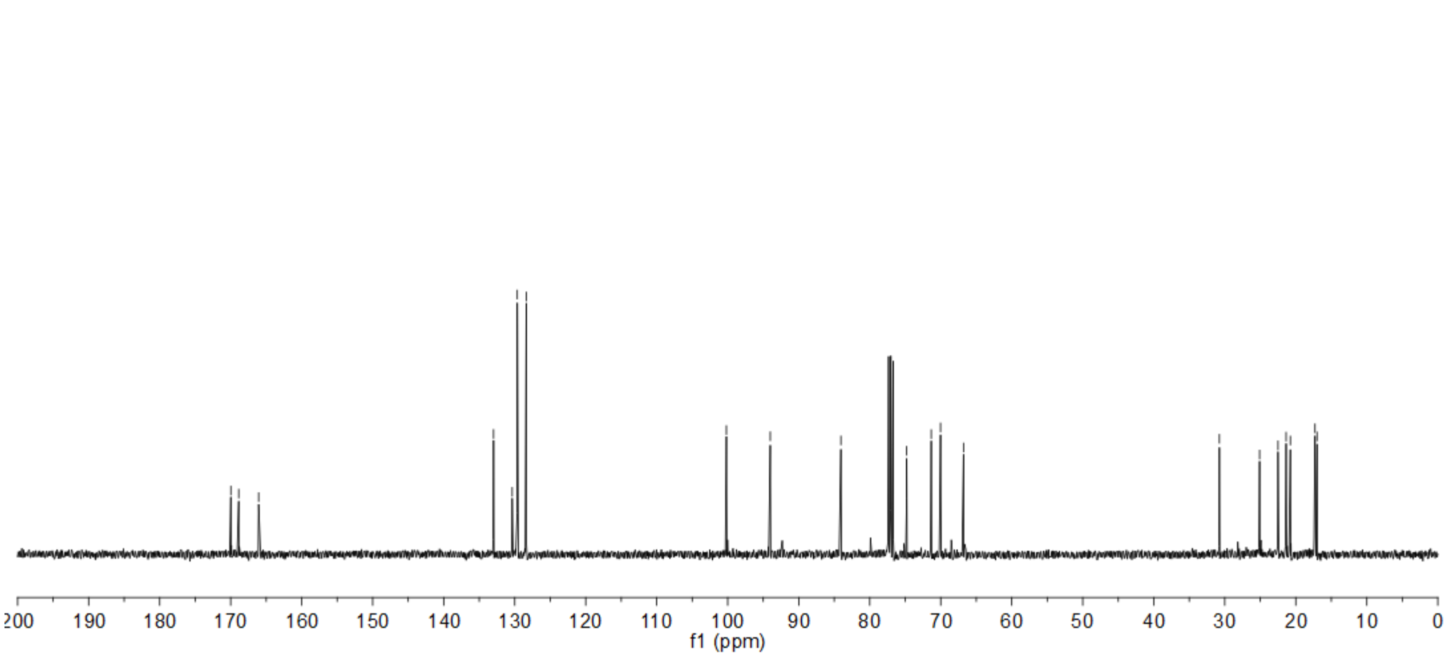

COSY NMR spectrum of α-Rhodinosyl-(1,3)-Olivosyl Acetate **28**

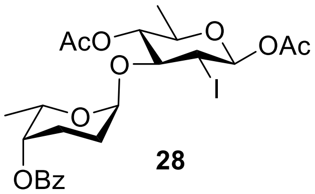

28

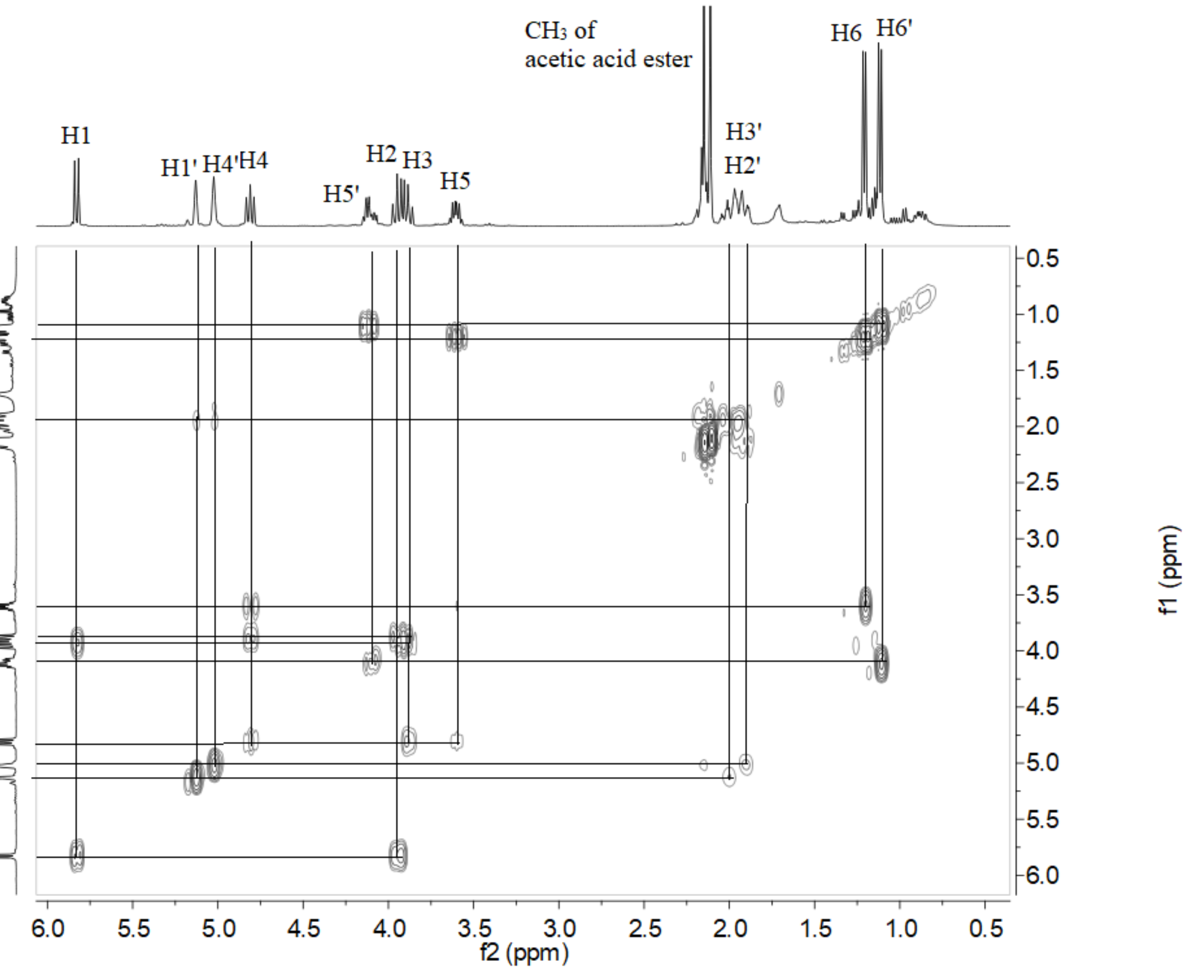

HSQC NMR spectrum of α-Rhodinosyl-(1,3)-Olivosyl Acetate **28**

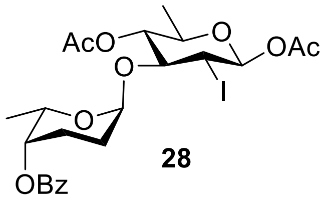

28

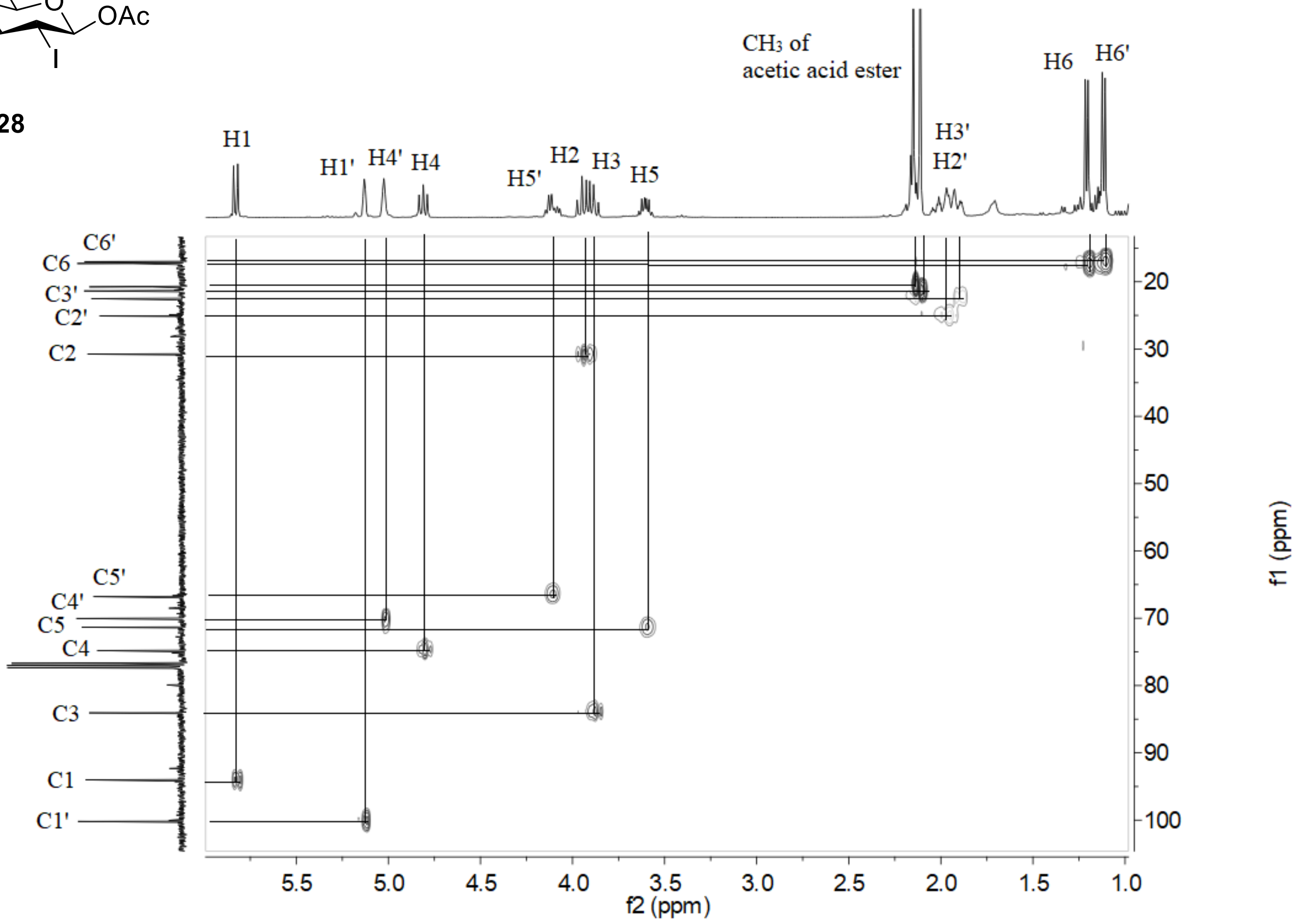

^{1}H NMR spectrum of protected LA Q **30**

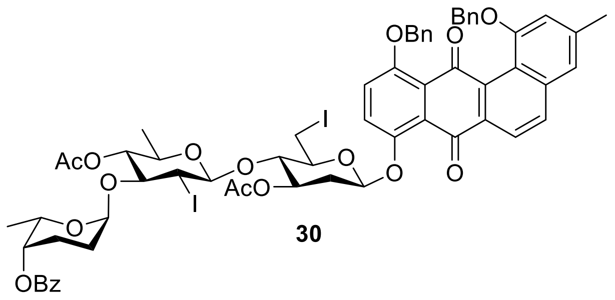

30

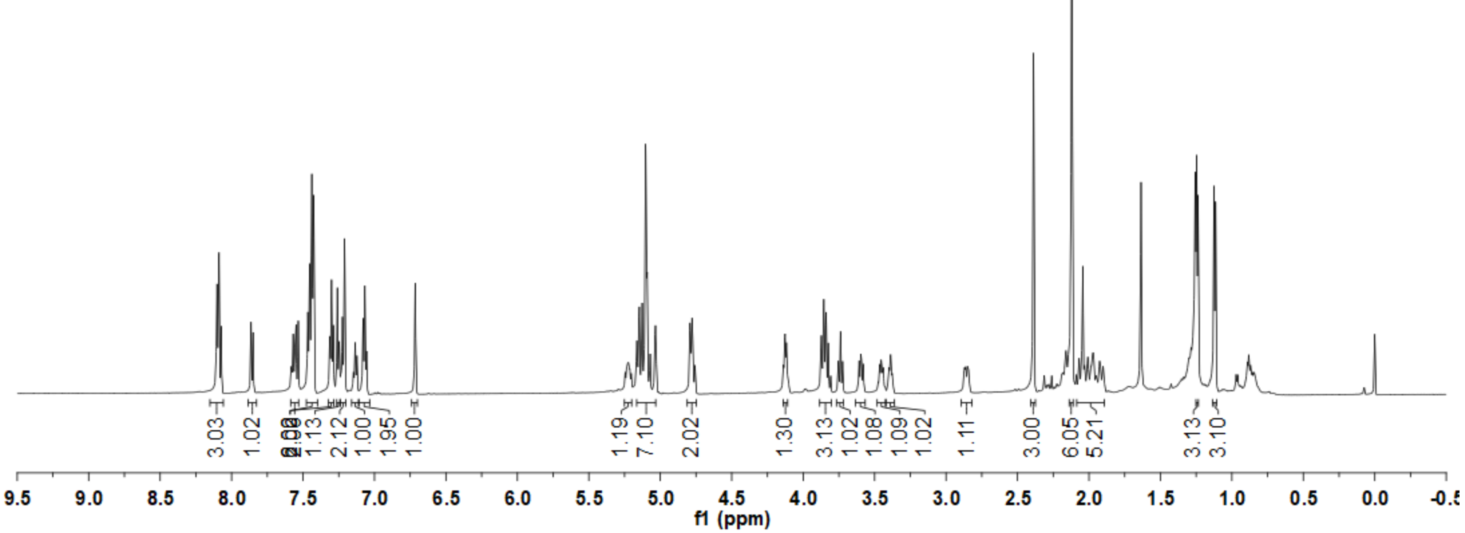

^{13}C NMR spectrum of protected LA Q **30**

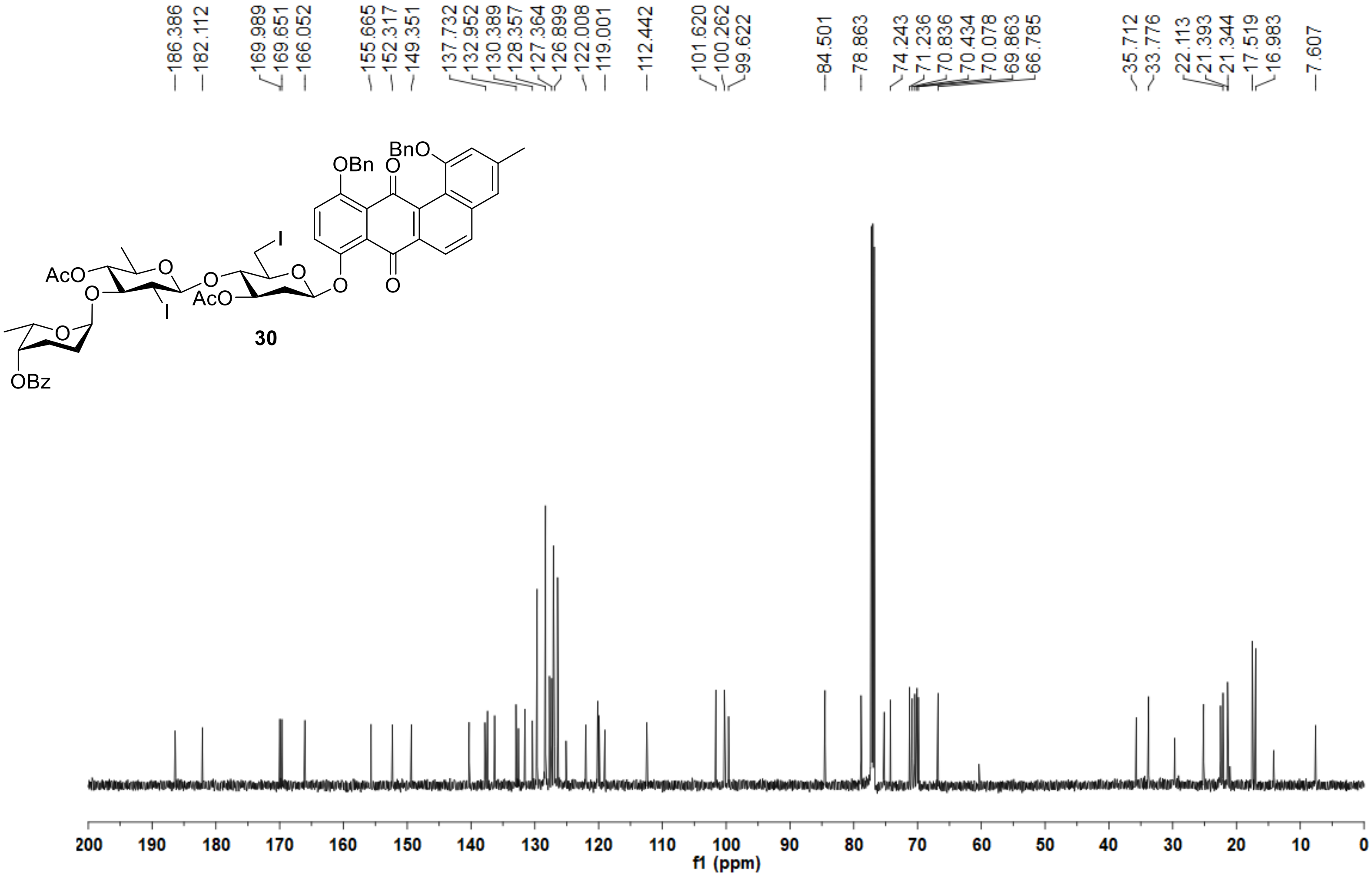

COSY NMR spectrum of protected LA Q **30**

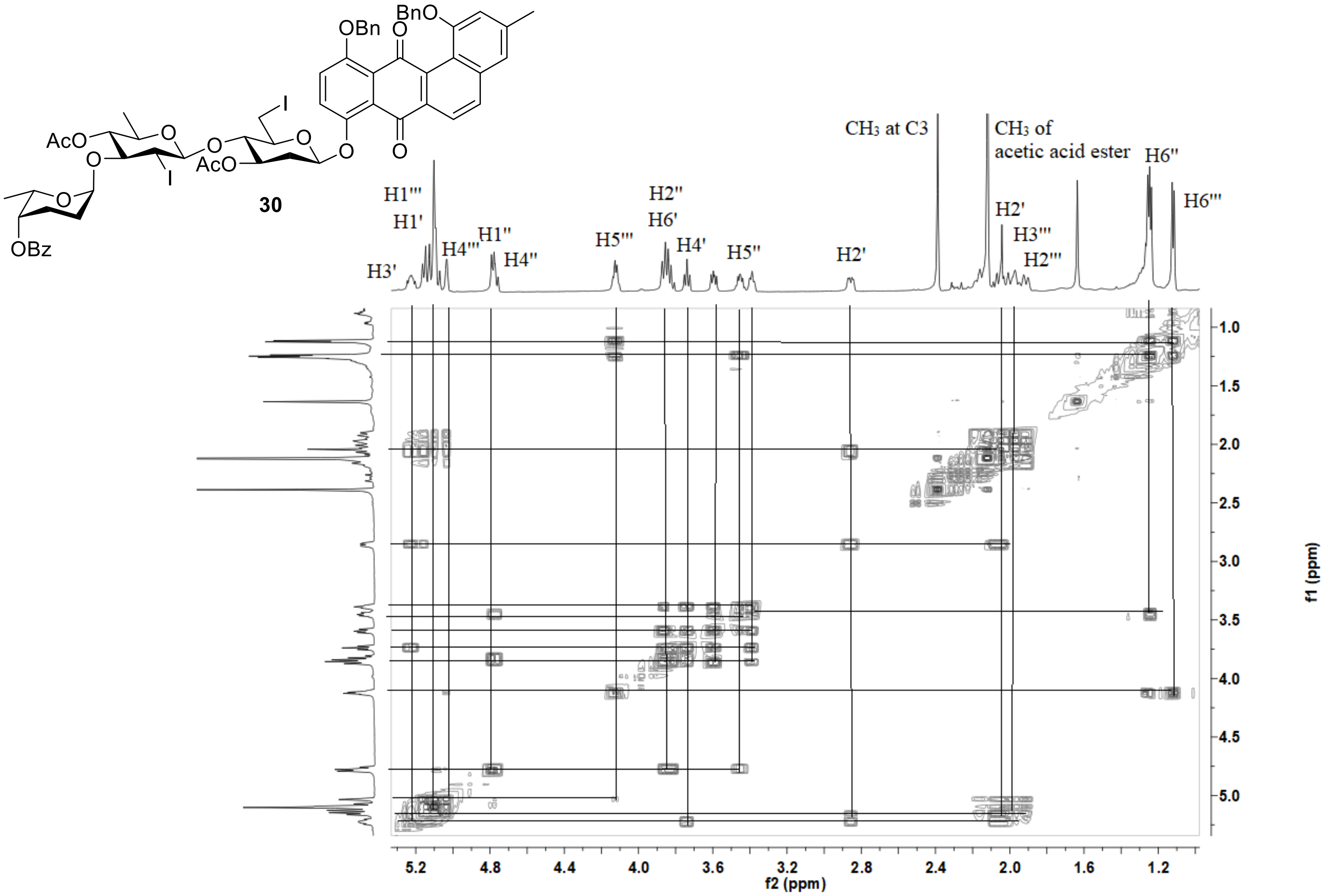

HSQC NMR spectrum of protected LA Q **30**

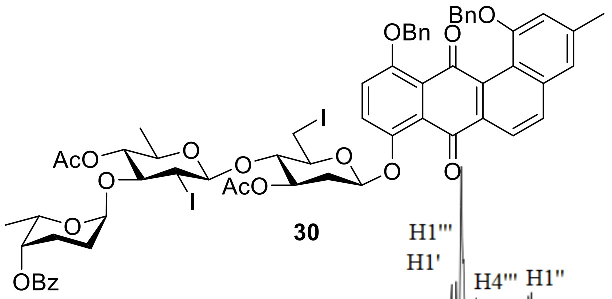

30

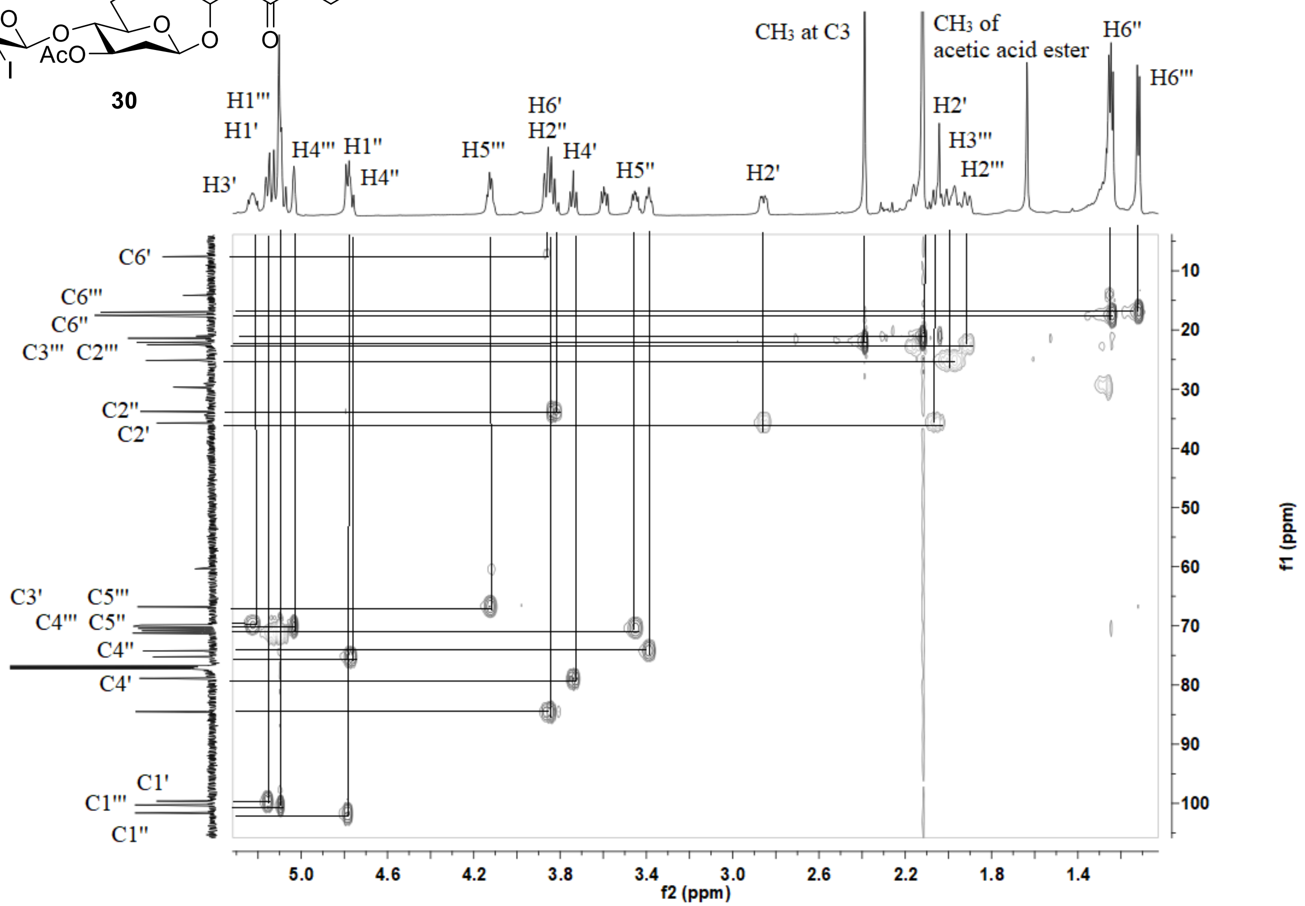

^{1}H NMR spectrum of landomycin Q (**3**)

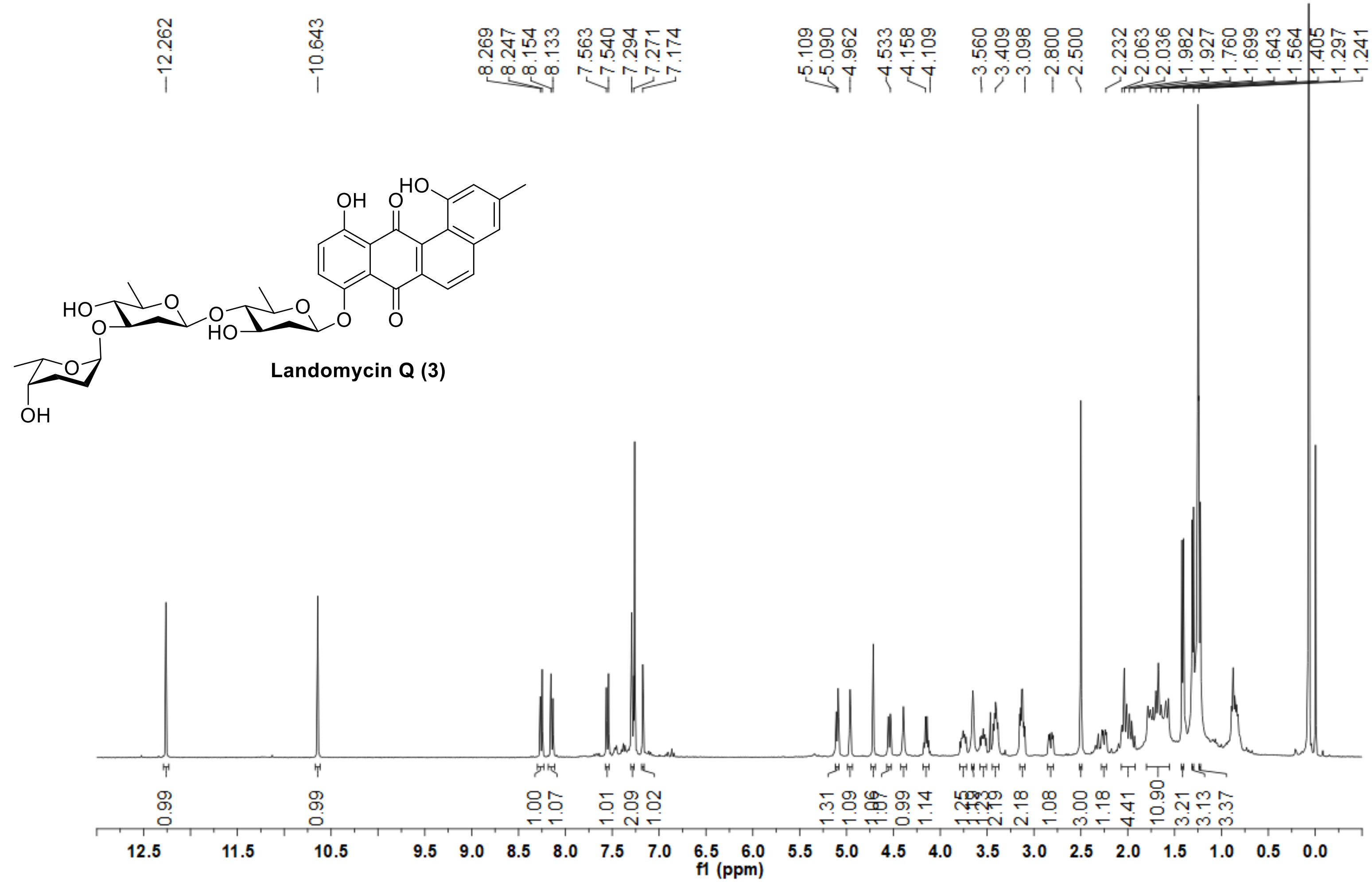

^{13}C NMR spectrum of landomycin Q (**3**)

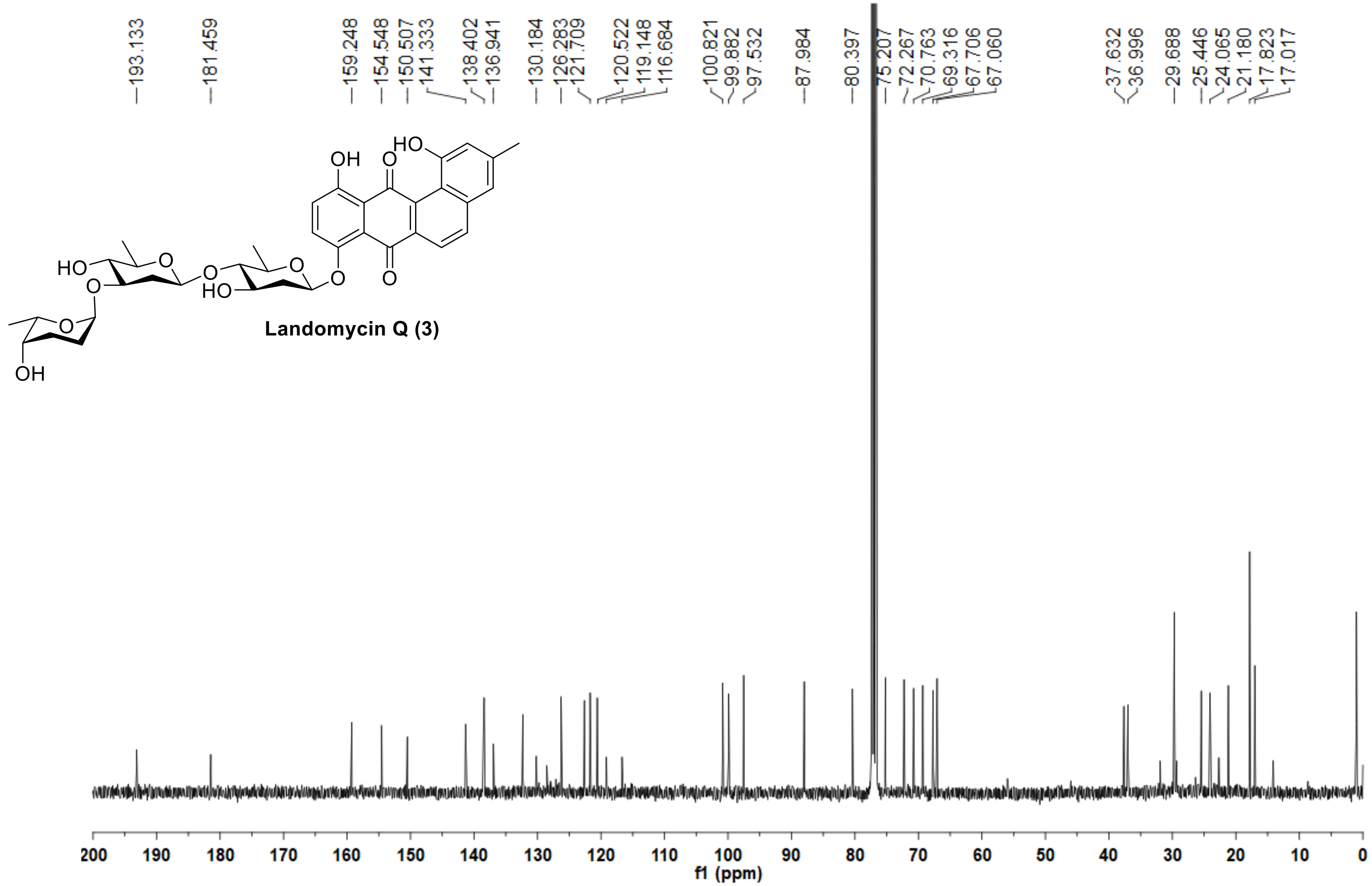

COSY NMR spectrum of landomycin Q (**3**)

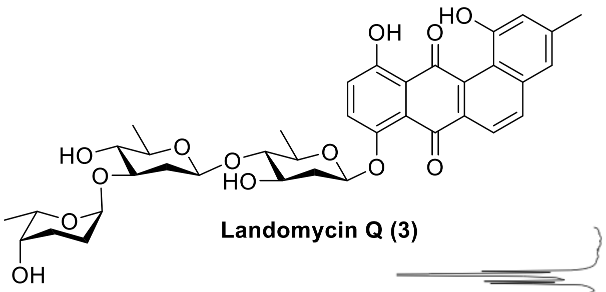

Landomycin Q (3)

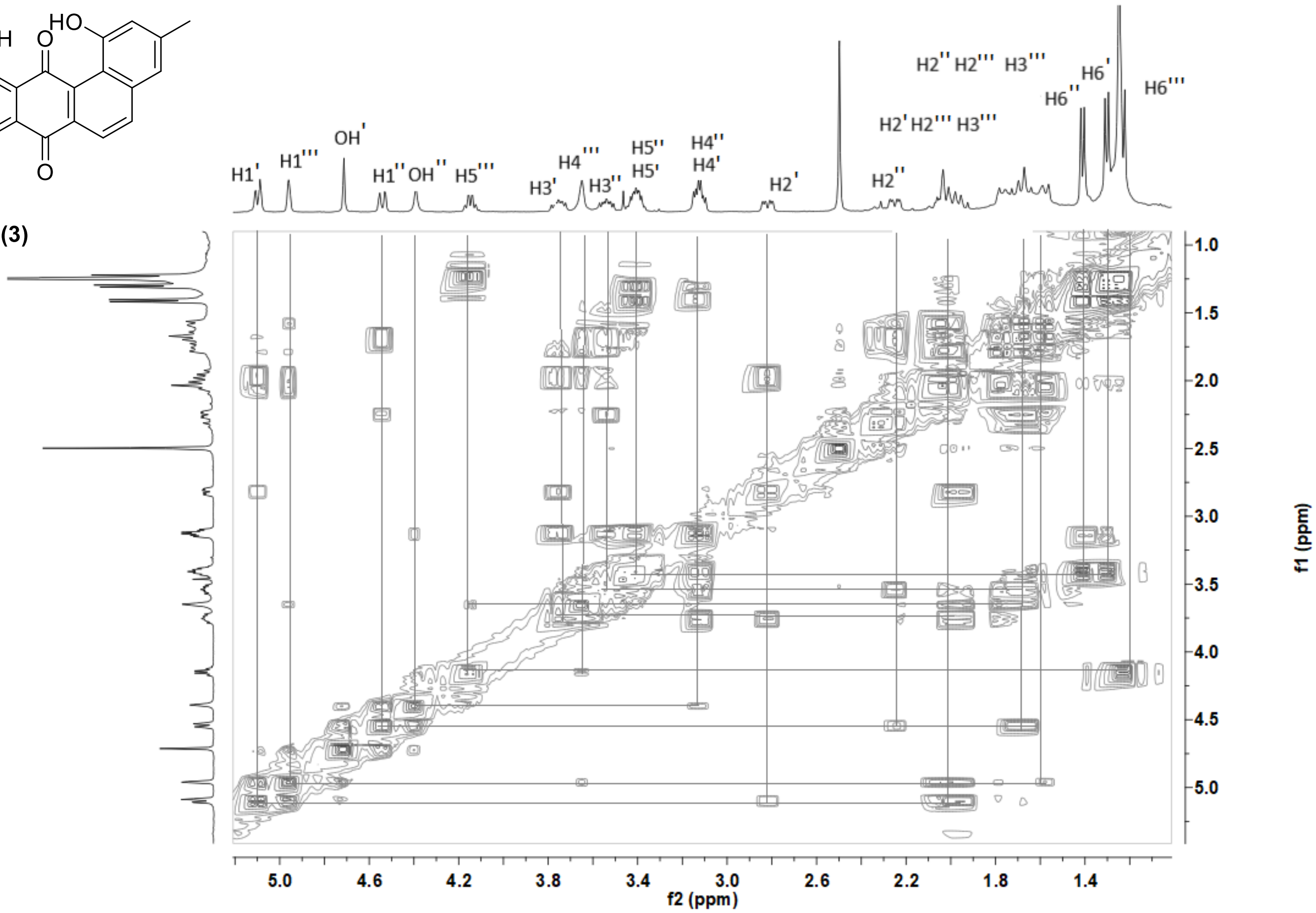

HSQC NMR spectrum of landomycin Q (**3**)

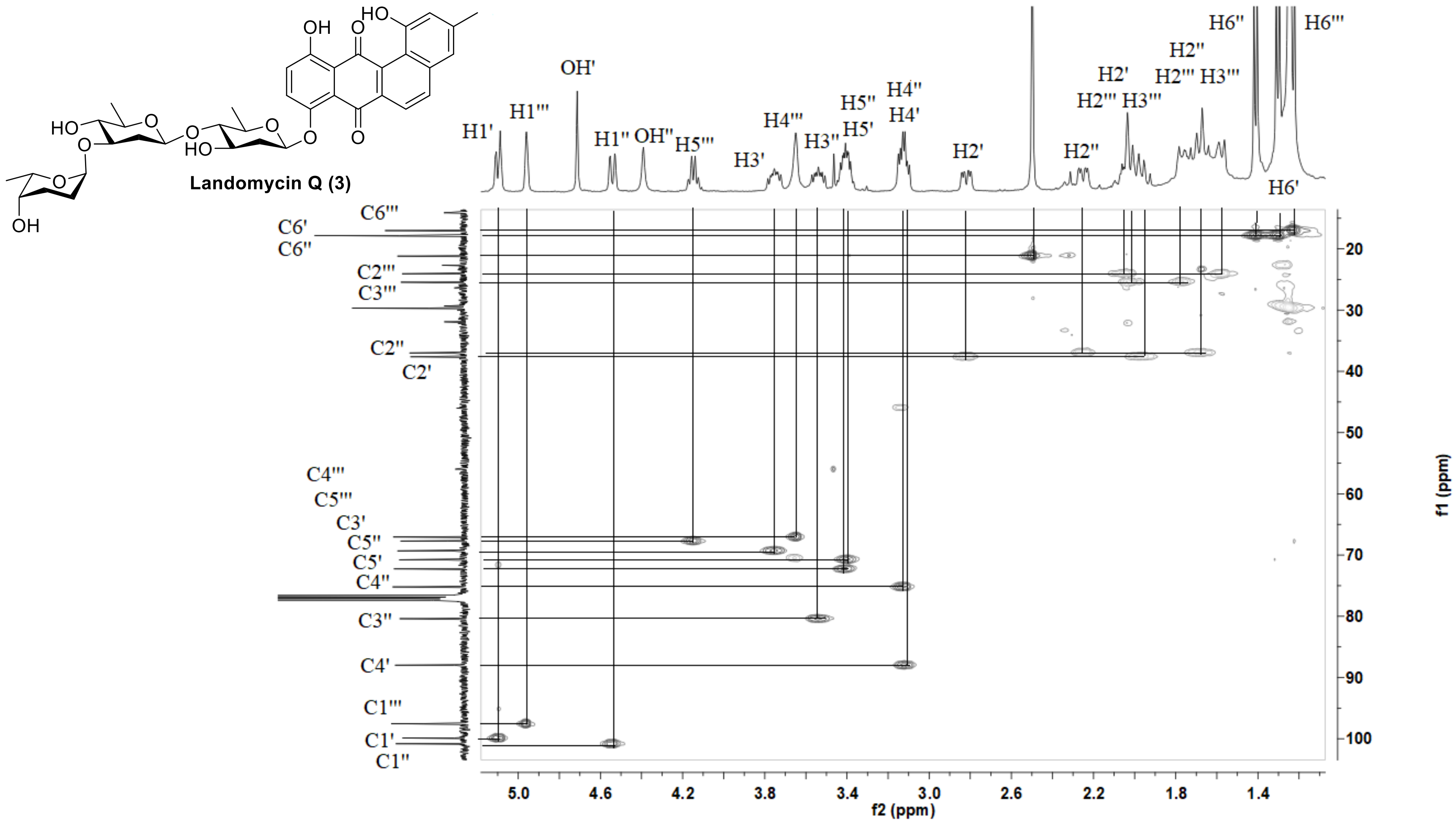

^{1}H NMR spectrum of 5,6-Dihydrotetraphene-7,12-dione *β*-olivoside **31**

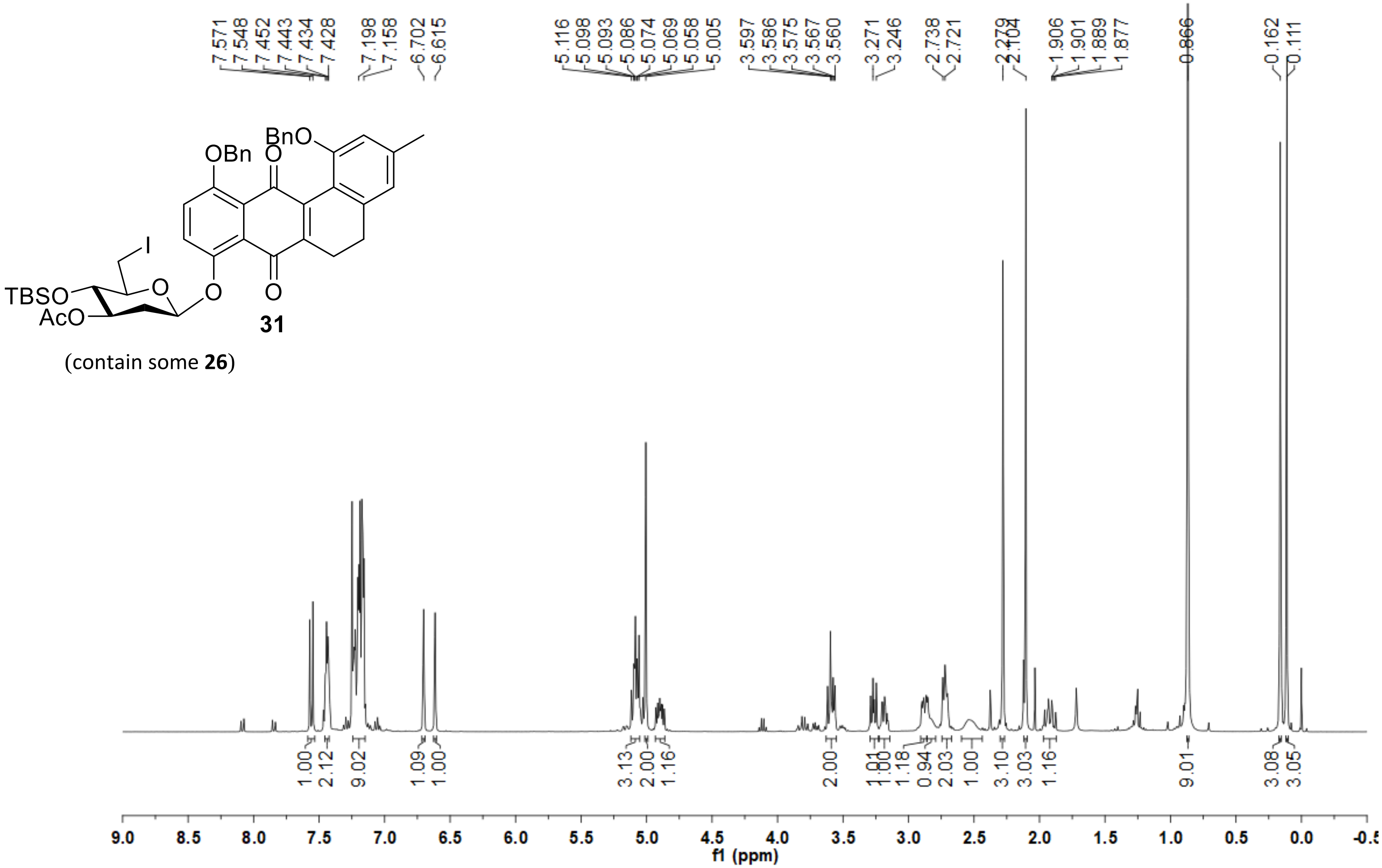

^{13}C NMR spectrum of 5,6-Dihydrotetraphene-7,12-dione *β*-Olivoside **31**

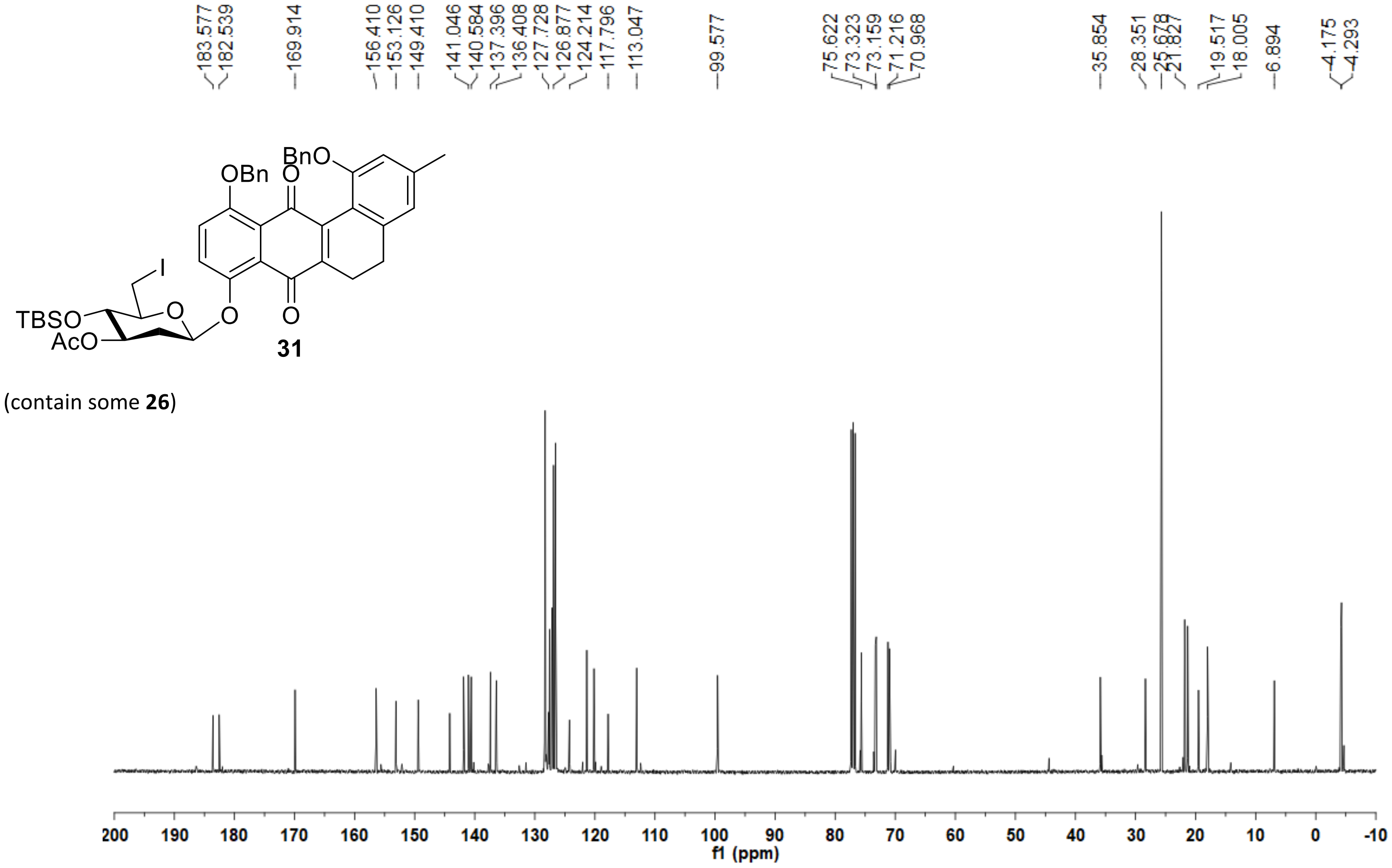

COSY NMR spectrum of 5,6-Dihydrotetraphene-7,12-dione β-Olivoside **31**

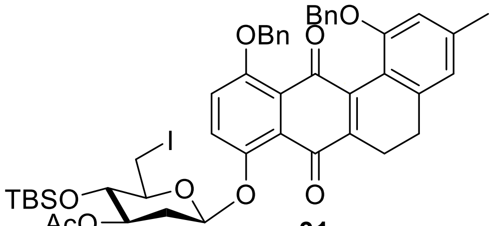

31

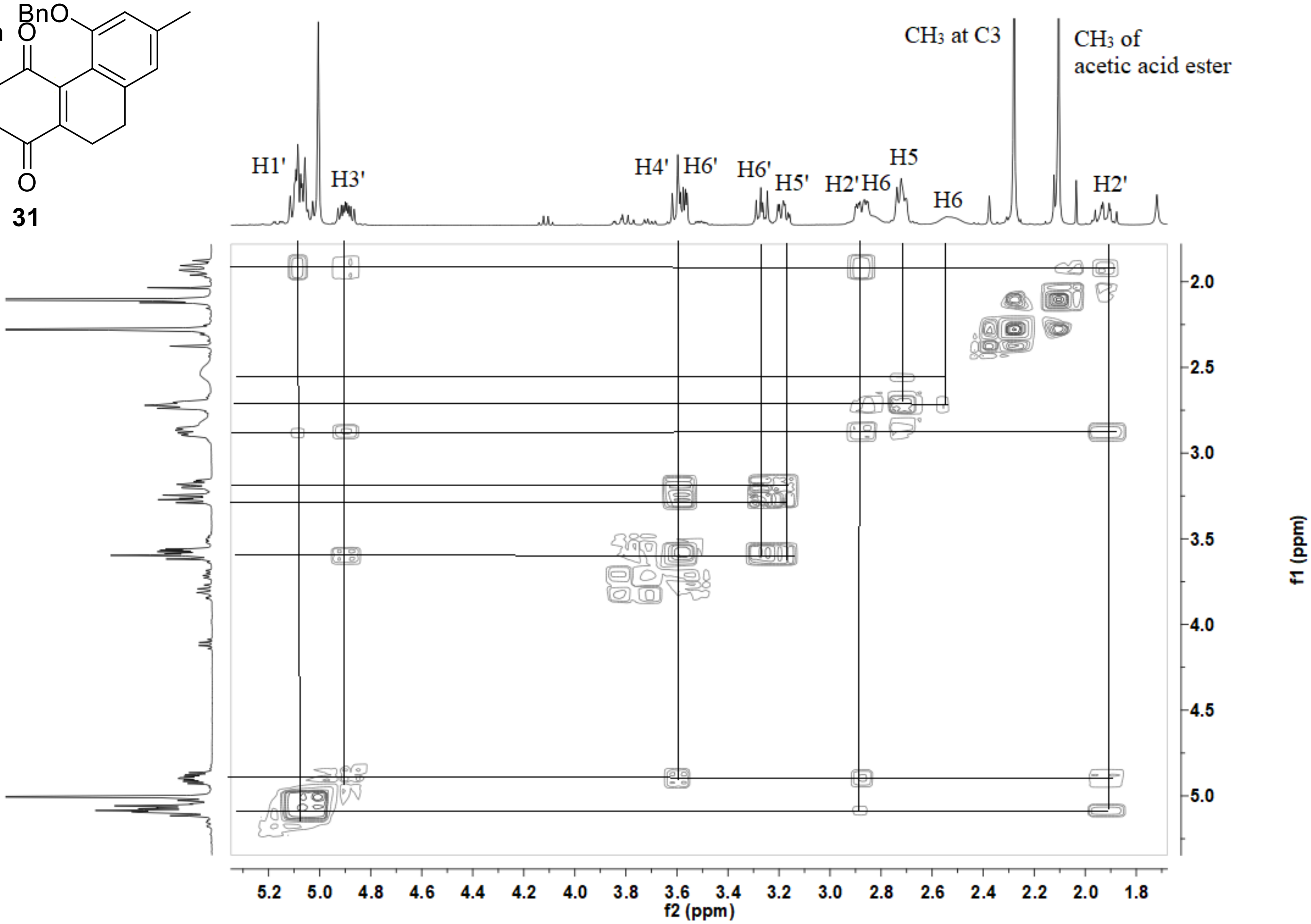

HSQC NMR spectrum of 5,6-Dihydrotetraphene-7,12-dione *β*-Olivoside **31**

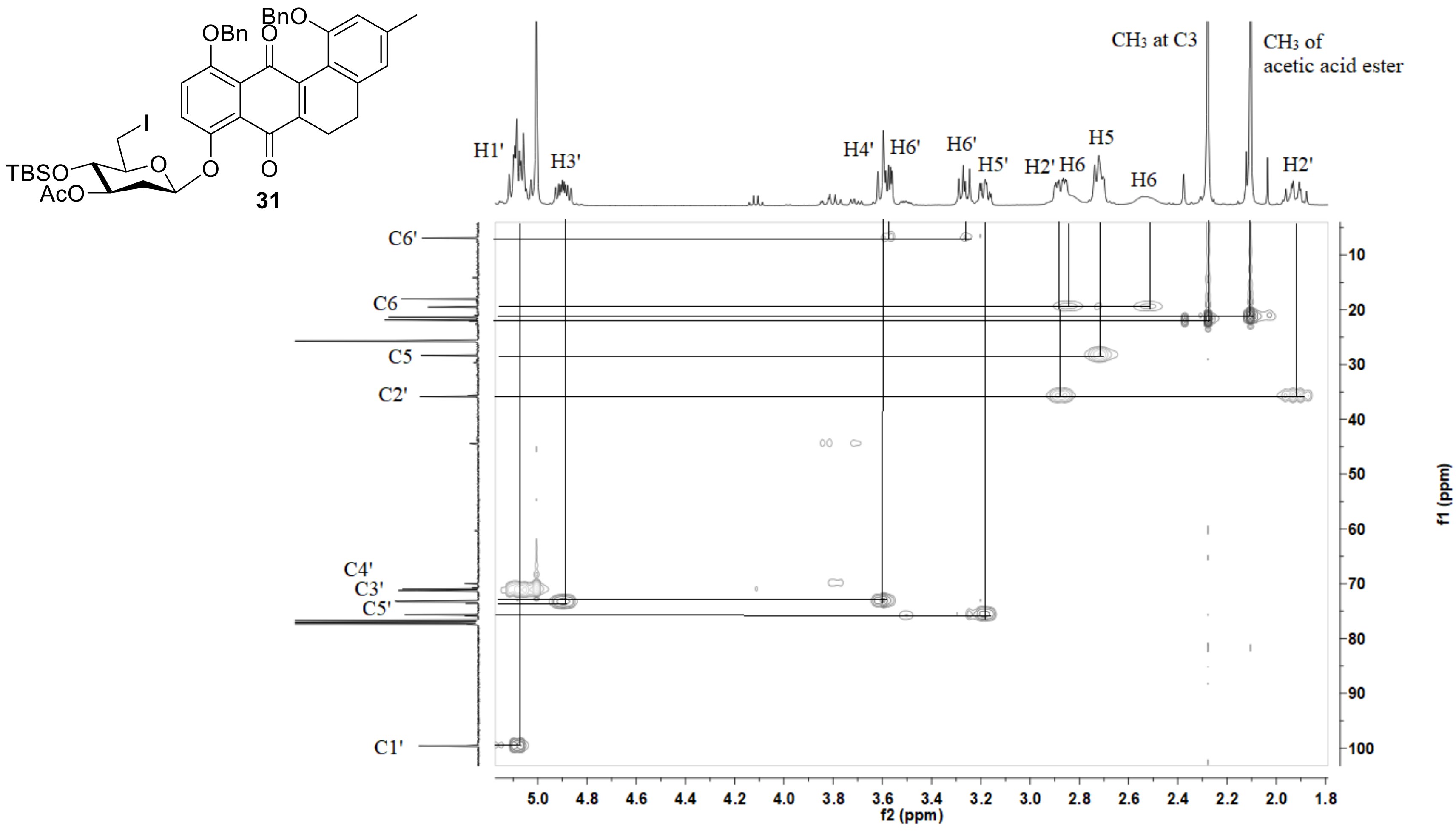

^{1}H NMR spectrum of 5,6-Dihydrotetraphene-7,12-dione *β*-Olivoside Acceptor **32**

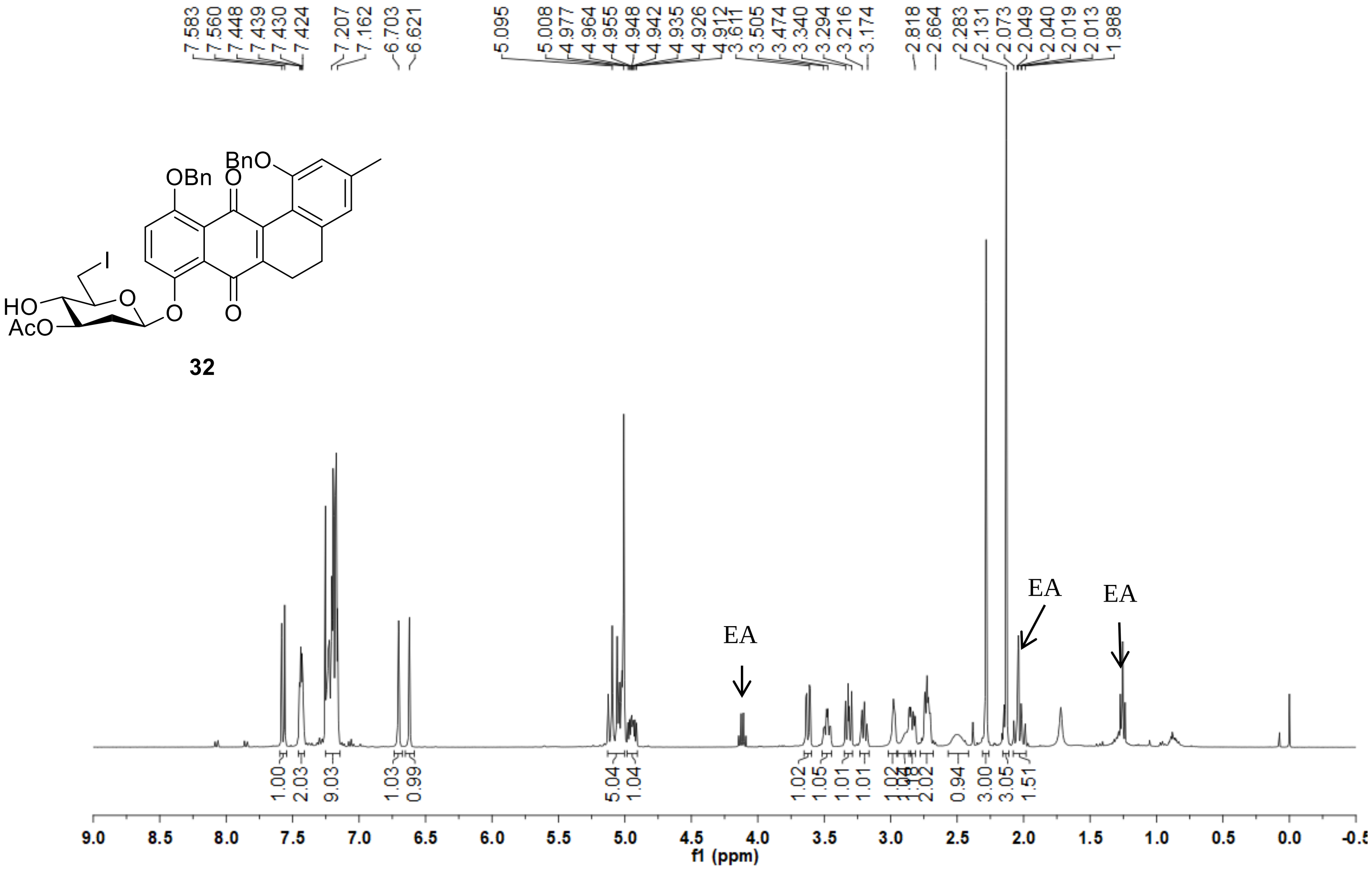

32

^{13}C NMR spectrum of 5,6-Dihydrotetraphene-7,12-dione β-Olivoside Acceptor **32**

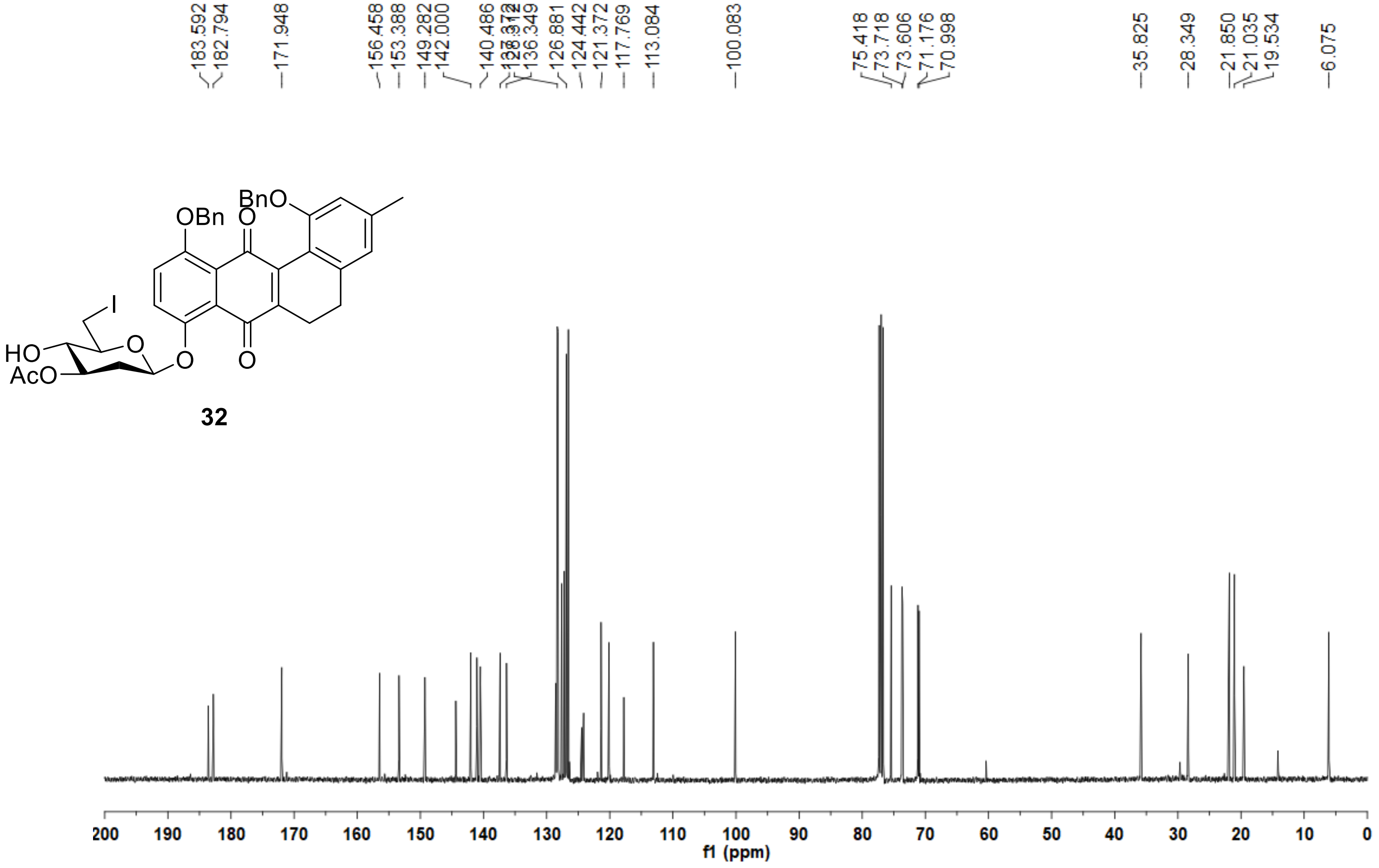

32

COSY NMR spectrum of 5,6-Dihydrotetraphene-7,12-dione β-Olivoside Acceptor **32**

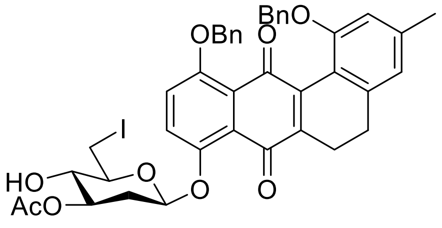

32

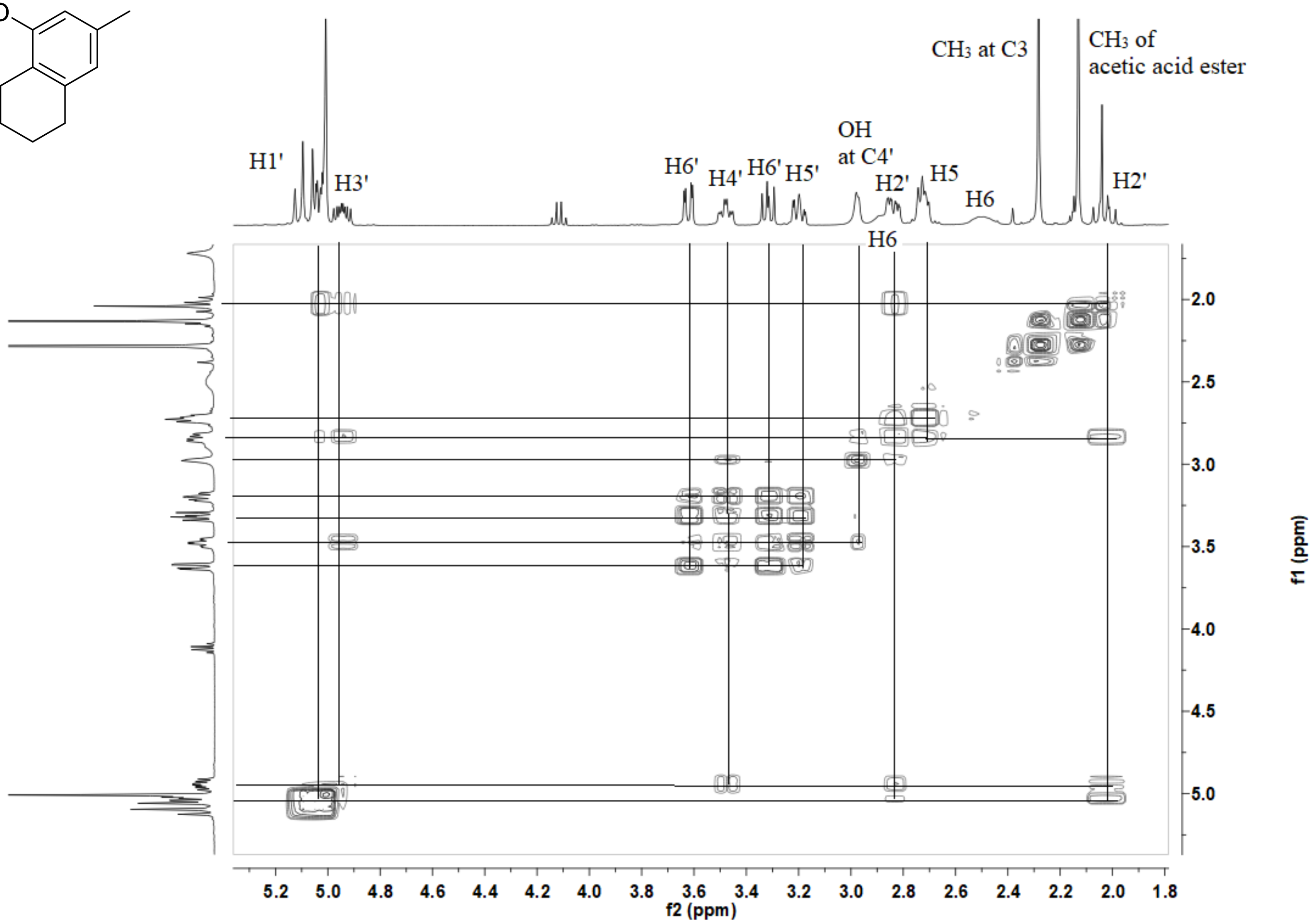

HSQC NMR spectrum of 5,6-Dihydrotetraphene-7,12-dione β-Olivoside Acceptor **32**

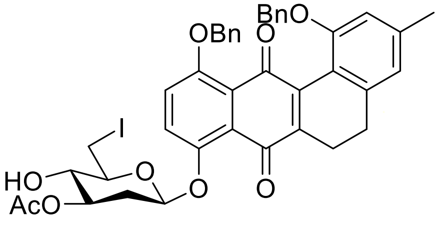

32

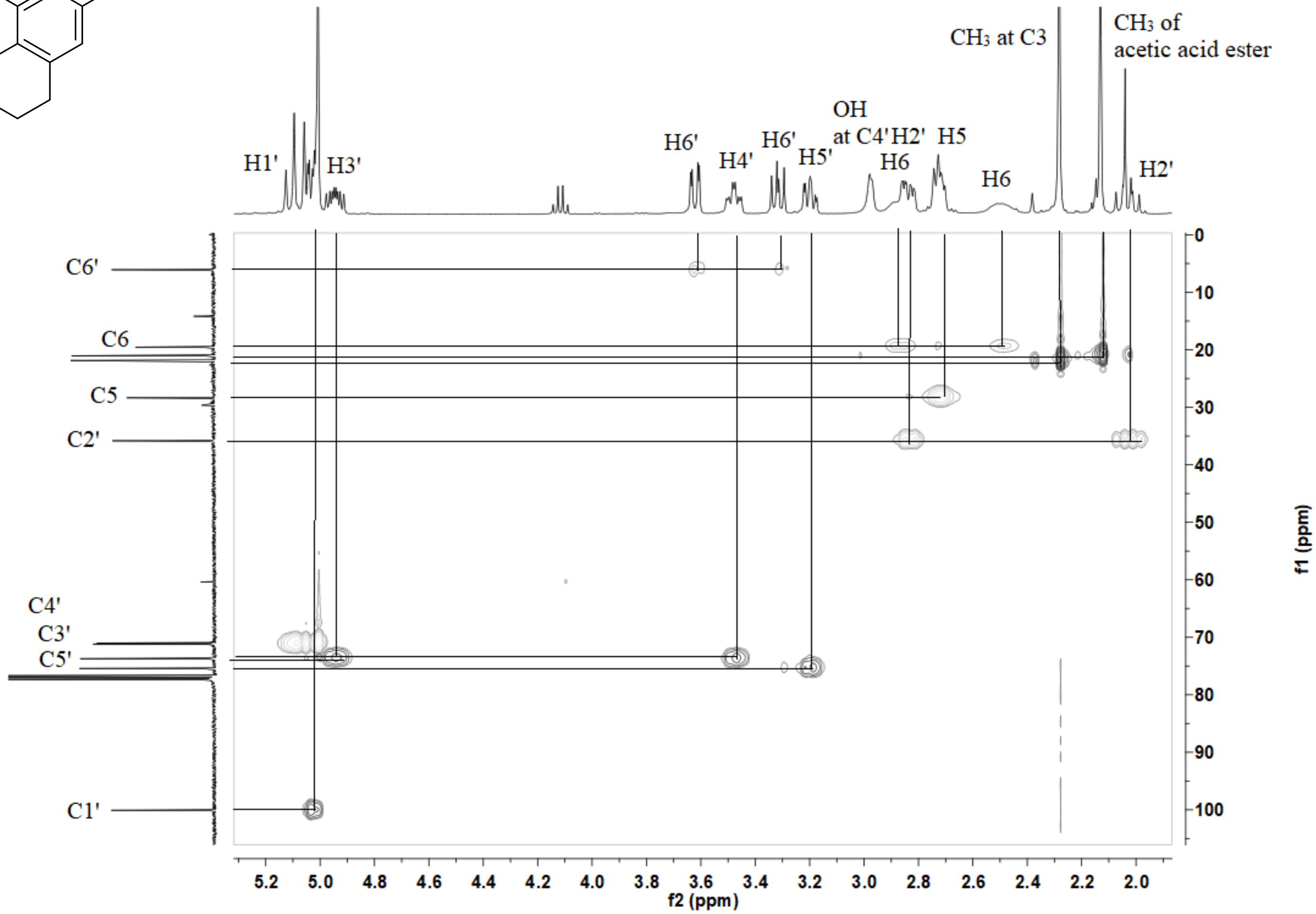

^{1}H NMR spectrum of Protected 8-Disaccharidyl-5,6-dihydrotetraphene-7,12-dione **34**

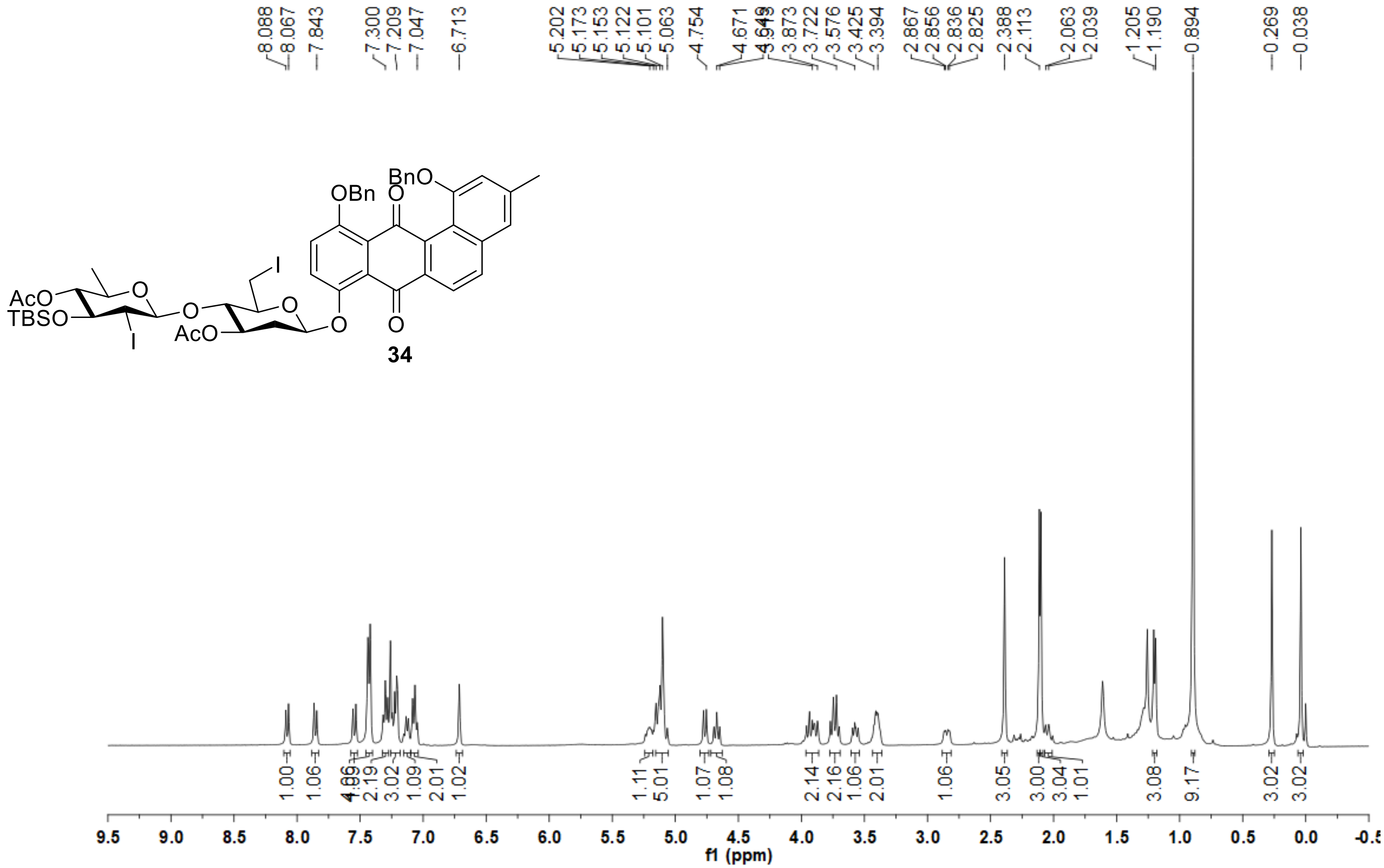

^{13}C NMR spectrum of Protected 8-Disaccharidyl-5,6-dihydrotetraphene-7,12-dione **34**

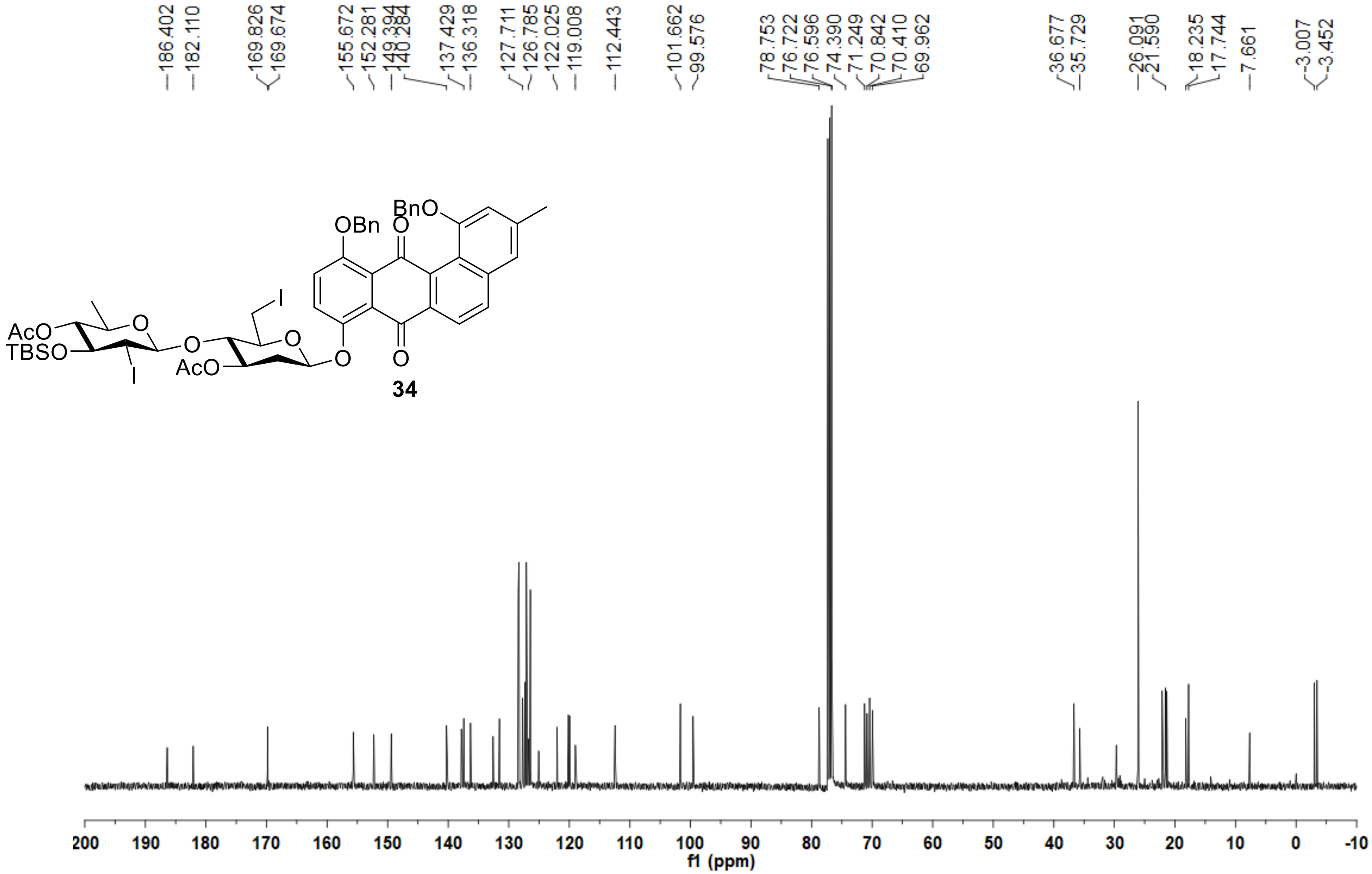

COSY NMR spectrum of Protected 8-Disaccharidyl-5,6-dihydrotetraphene-7,12-dione **34**

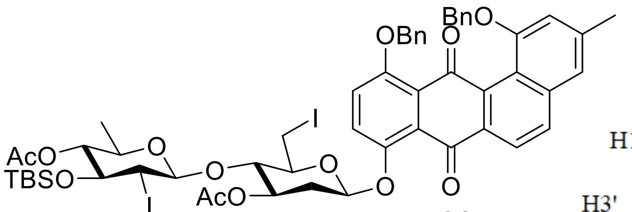

34

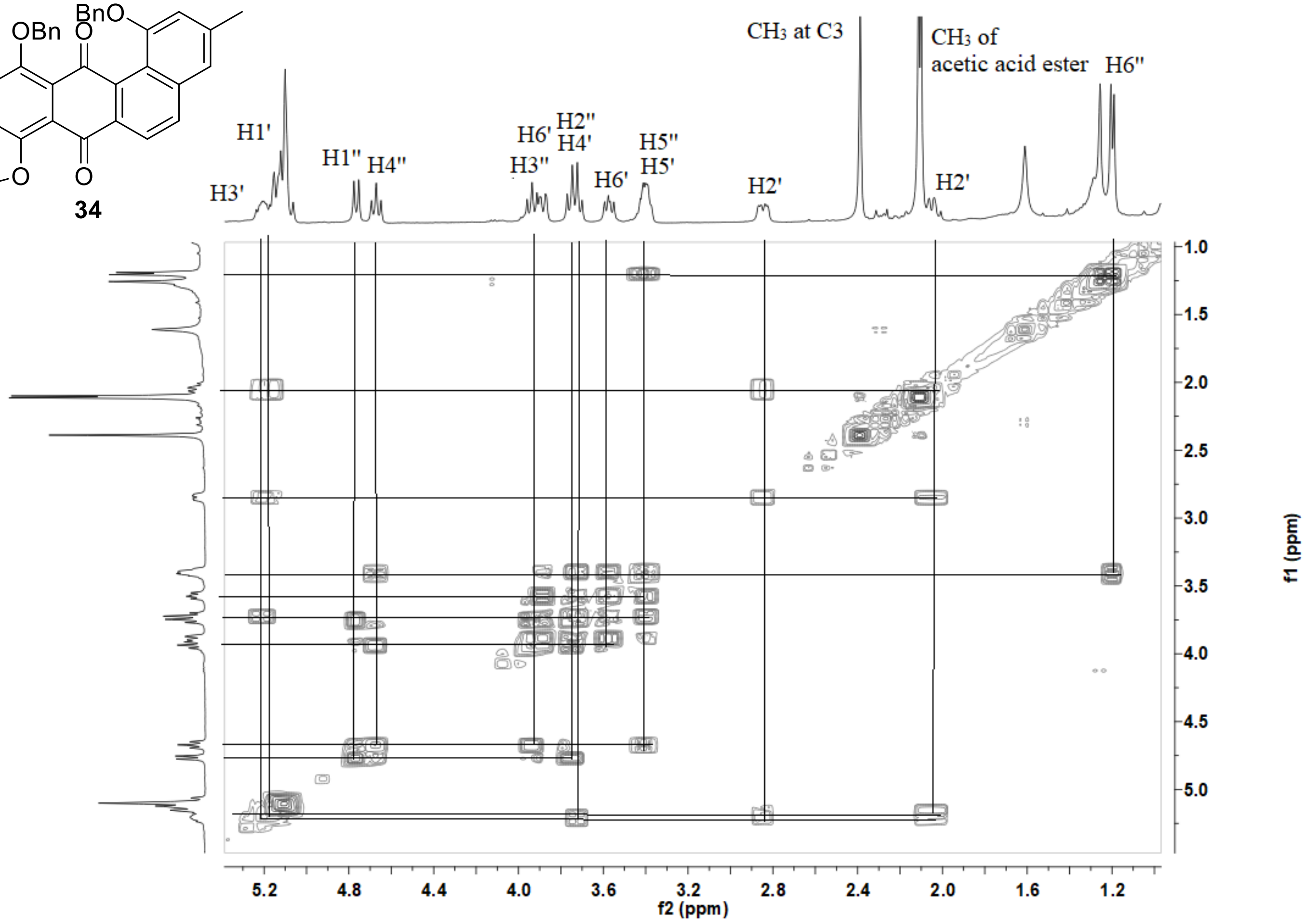

HSQC NMR spectrum of Protected 8-Disaccharidyl-5,6-dihydrotetraphene-7,12-dione **34**

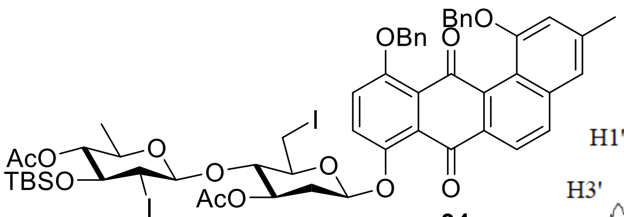

34

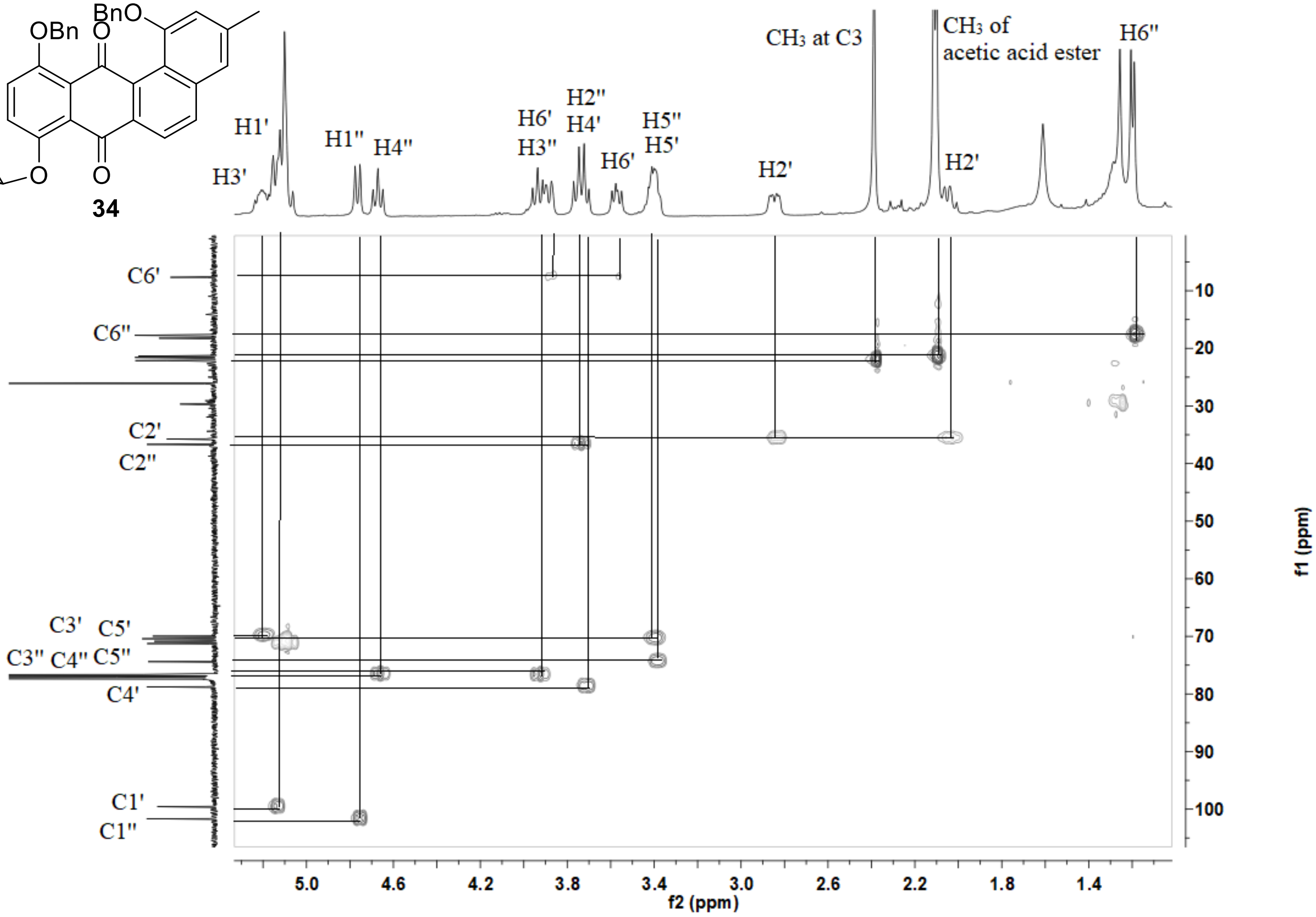

^{1}H NMR spectrum of Partially Deprotected LA R intermediate **34A**

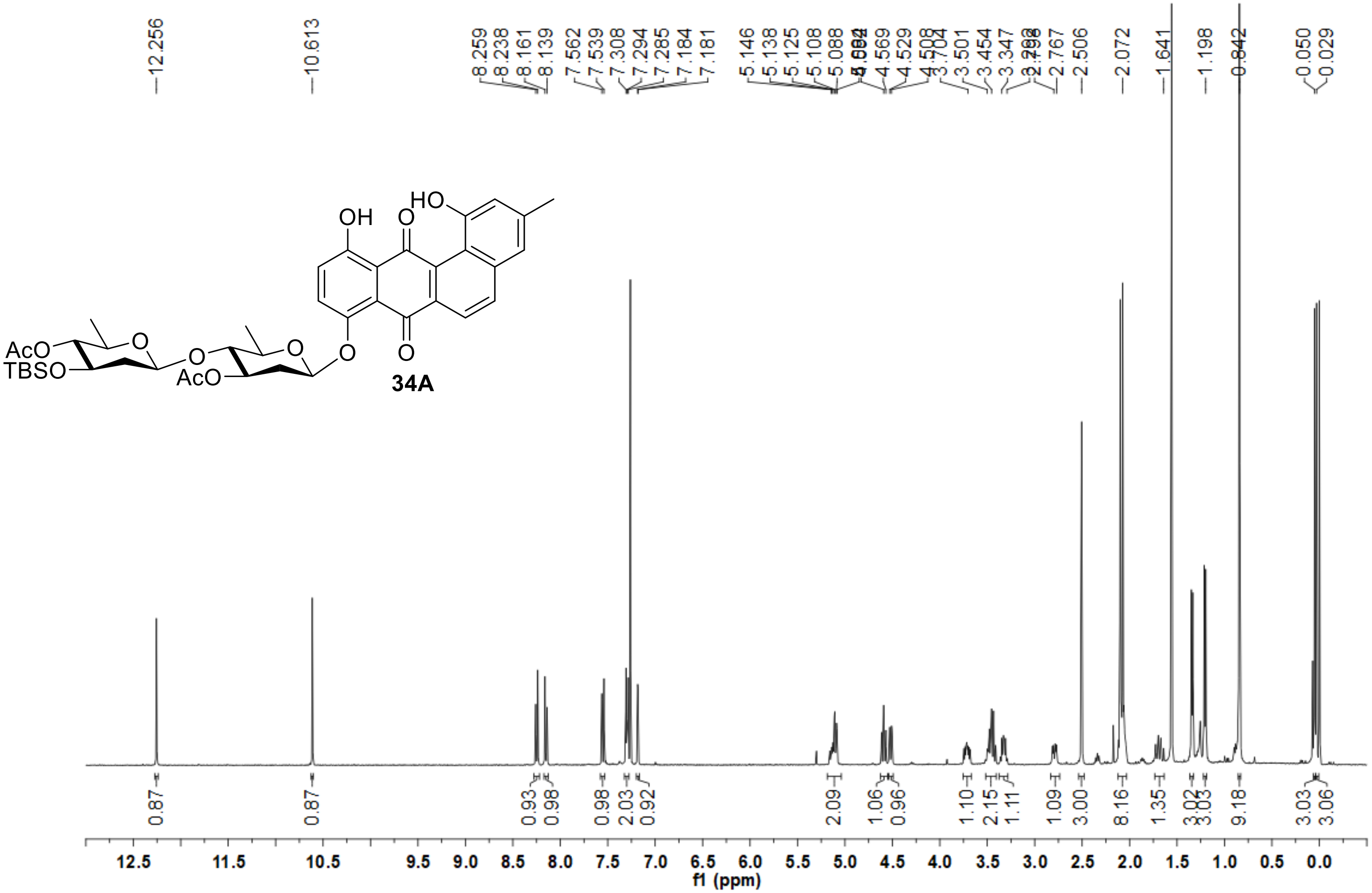

^{1}H NMR spectrum of Landomycin R (**4**)

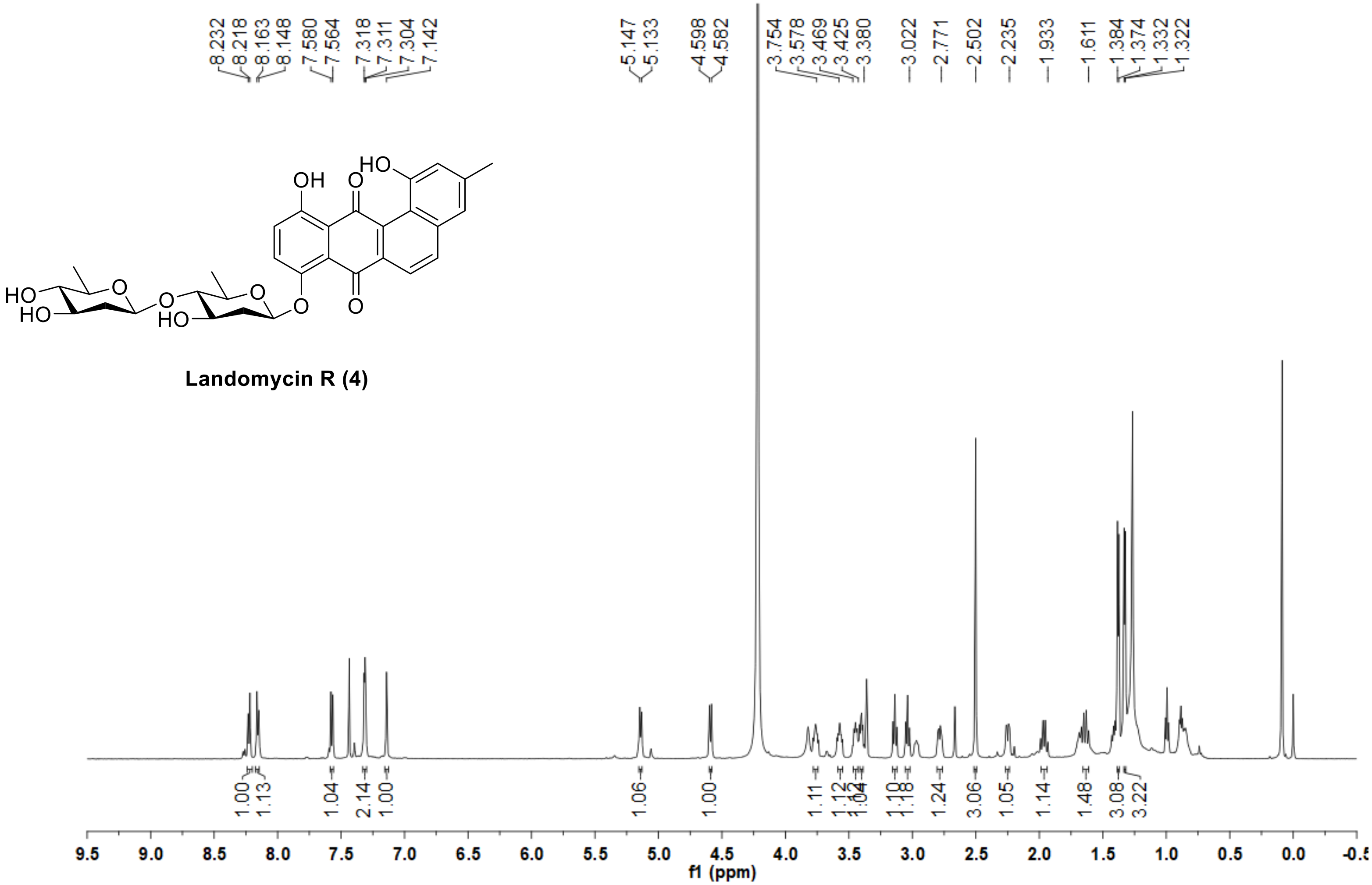

^{13}C NMR spectrum of Landomycin R (**4**)

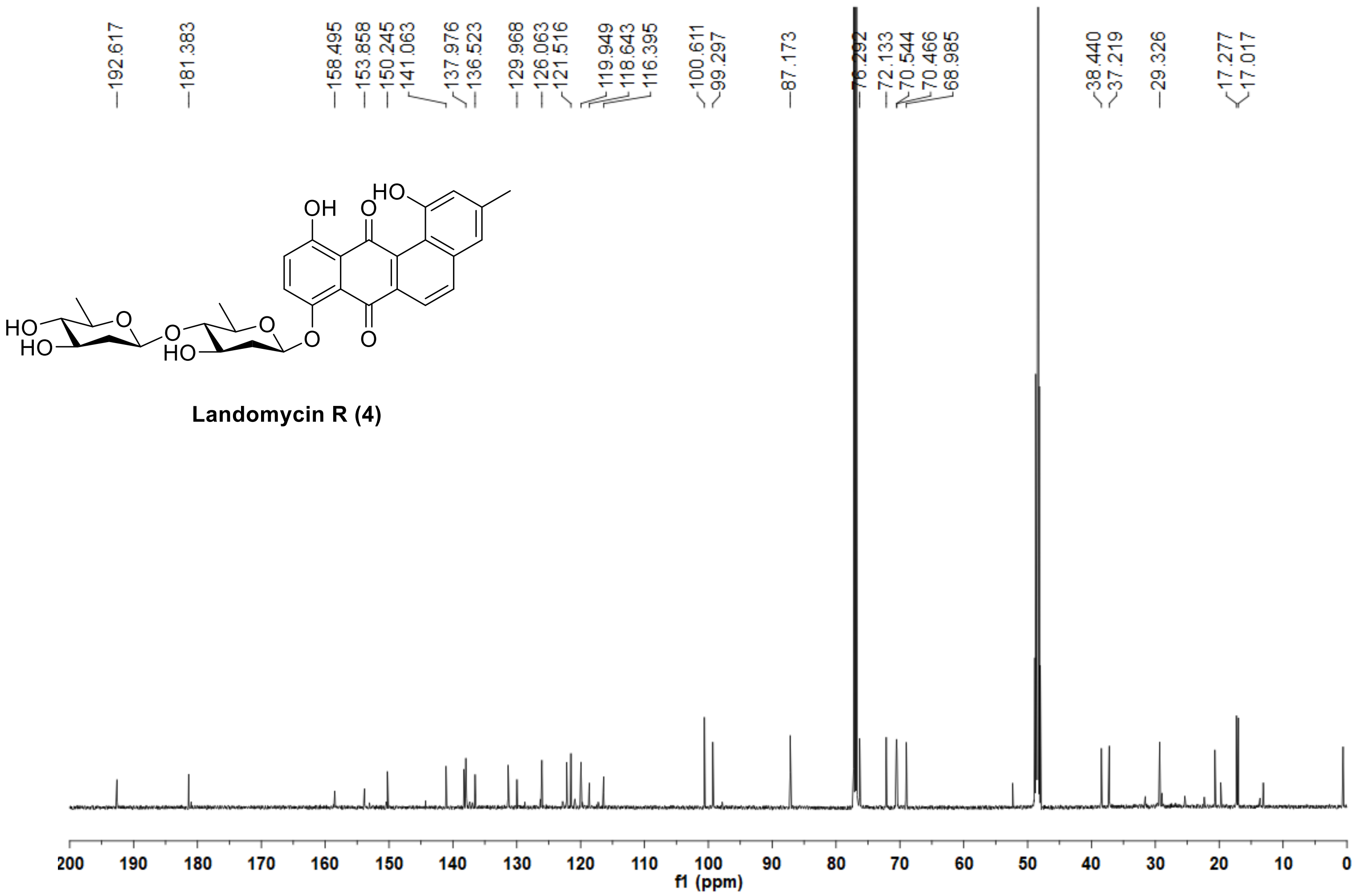

COSY NMR spectrum of Landomycin R (**4**)

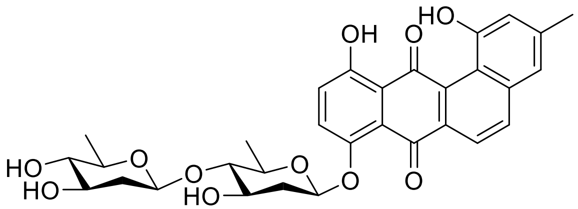

Landomycin R (4)

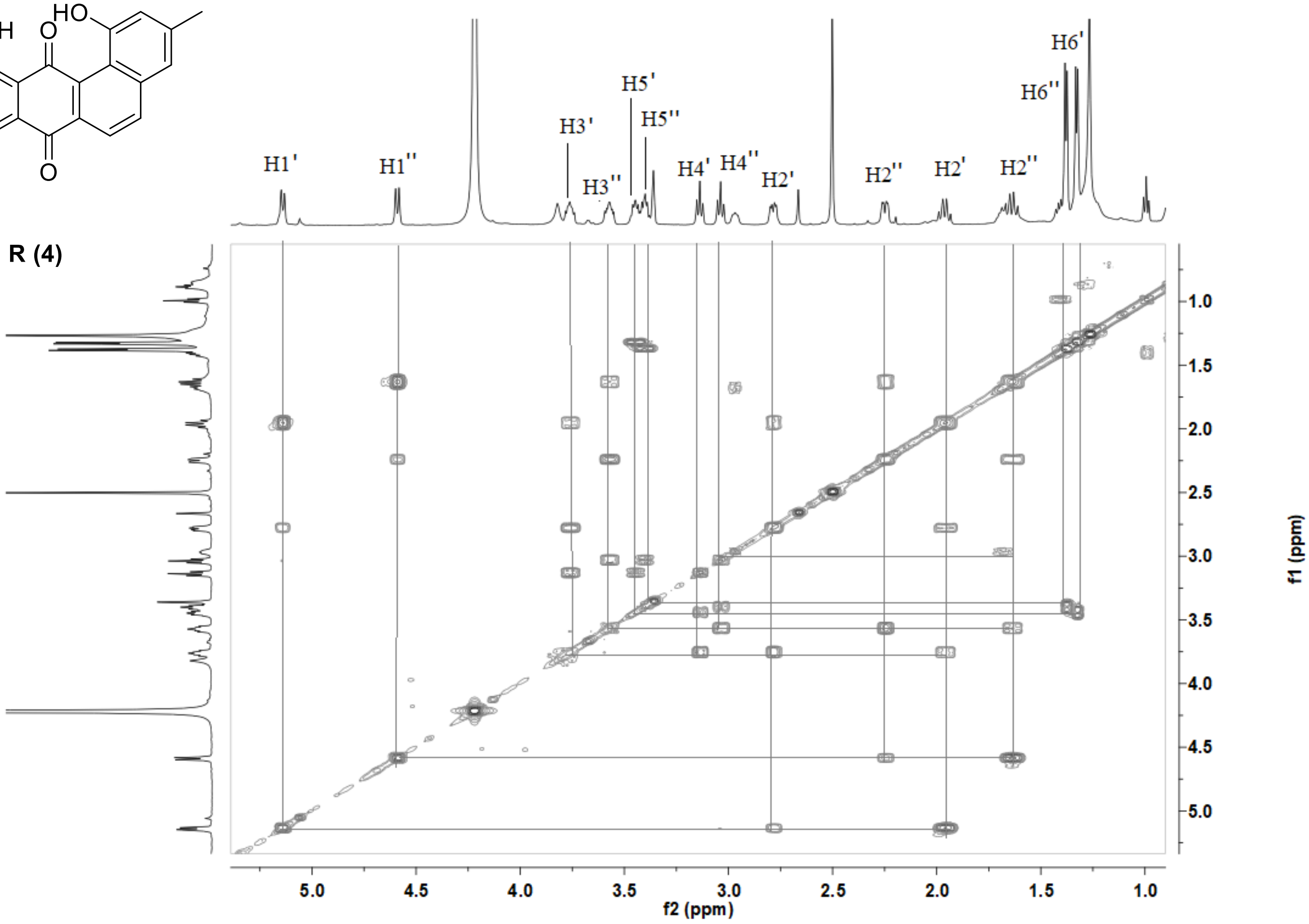

HSQC NMR spectrum of Landomycin R (**4**)

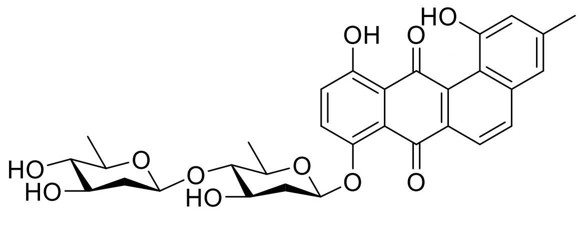

Landomycin R (4)

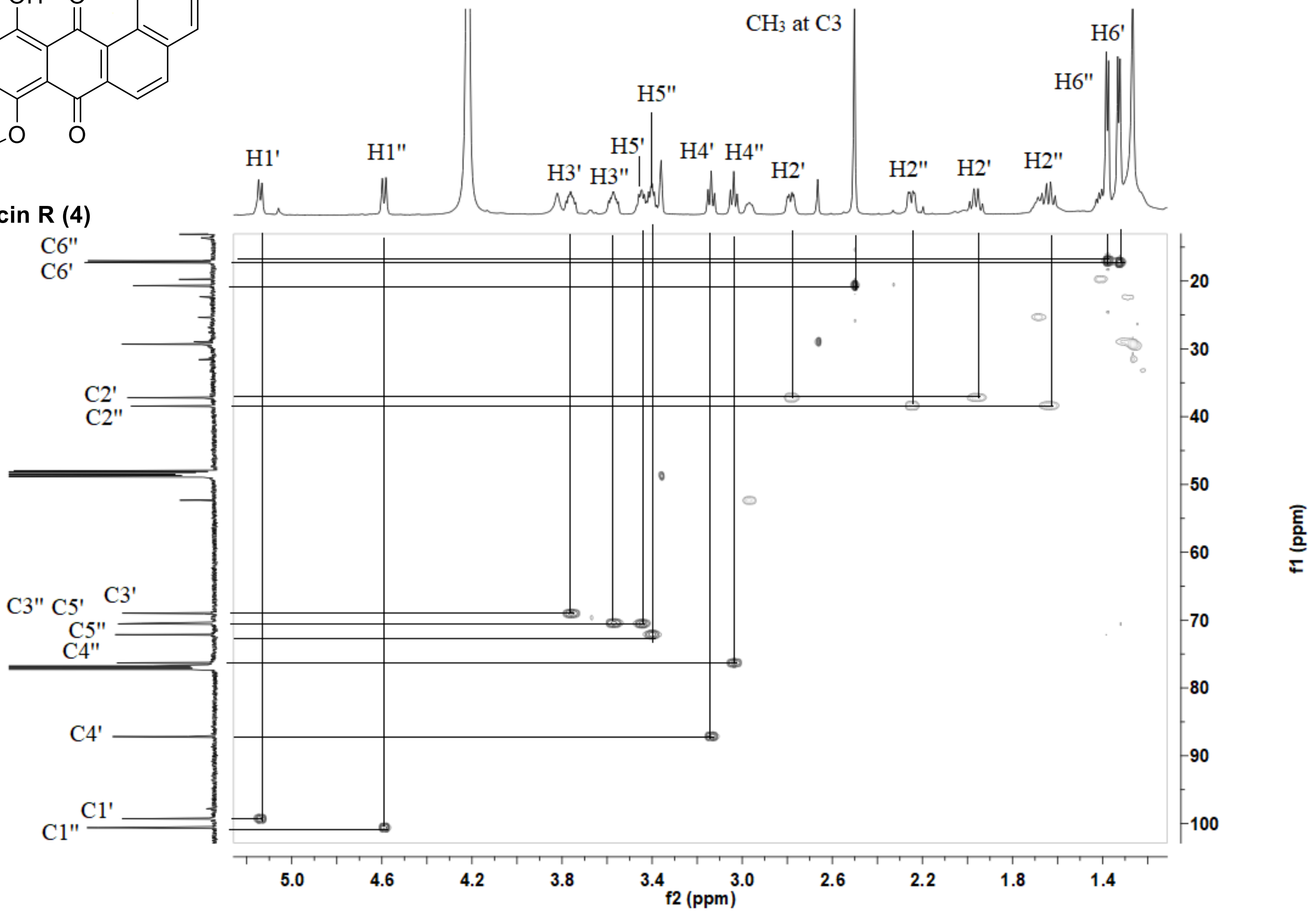

Supplement: RA-011-D1RA01088C-s001 [file RA-011-D1RA01088C-s001.pdf]
